# A Pareto approach to resolve the conflict between information gain and experimental costs: Multiple-criteria design of carbon labeling experiments

**DOI:** 10.1371/journal.pcbi.1006533

**Published:** 2018-10-31

**Authors:** Katharina Nöh, Sebastian Niedenführ, Martin Beyß, Wolfgang Wiechert

**Affiliations:** 1 Institute of Bio- and Geosciences, IBG-1: Biotechnology, Forschungszentrum Jülich GmbH, Jülich, Germany; 2 Computational Systems Biotechnology, RWTH Aachen University, Aachen, Germany; Ecole Polytechnique Fédérale de Lausanne, SWITZERLAND

## Abstract

Science revolves around the best way of conducting an experiment to obtain insightful results. Experiments with maximal information content can be found by computational experimental design (ED) strategies that identify optimal conditions under which to perform the experiment. Several criteria have been proposed to measure the information content, each emphasizing different aspects of the design goal, i.e., reduction of uncertainty. Where experiments are complex or expensive, second sight is at the budget governing the achievable amount of information. In this context, the design objectives *cost* and *information gain* are often incommensurable, though dependent. By casting the ED task into a multiple-criteria optimization problem, a set of trade-off designs is derived that approximates the Pareto-frontier which is instrumental for exploring preferable designs. In this work, we present a computational methodology for multiple-criteria ED of information-rich experiments that accounts for virtually any set of design criteria. The methodology is implemented for the case of ^13^C metabolic flux analysis (MFA), which is arguably the most expensive type among the ‘omics’ technologies, featuring dozens of design parameters (tracer composition, analytical platform, measurement selection etc.). Supported by an innovative visualization scheme, we demonstrate with two realistic showcases that the use of multiple criteria reveals deep insights into the conflicting interplay between information carriers and cost factors that are not amendable to single-objective ED. For instance, tandem mass spectrometry turns out as best-in-class with respect to information gain, while it delivers this information quality cheaper than the other, routinely applied analytical technologies. Therewith, our Pareto approach to ED offers the investigator great flexibilities in the conception phase of a study to balance costs and benefits.

## Author summary

Designing experiments is obligatory in the biosciences to valorize their scientific outcome. When the experiments are expensive, unfortunately, in practice often the costs emerge to be showstoppers. In this situation the question arises: How to get the most out of the experiment for your invest in terms of time and money? We approach this question by formulating the design task as a multiple-criteria optimization problem. Its solution produces a set of Pareto-optimal design proposals that feature the trade-off between information gain, as measured by different metrics, and the costs. Then, exploration of the design proposals allows us to make the best decision on information-economic experiments under given circumstances. Implemented in the field of isotope-based metabolic flux analysis, practical application of the Pareto approach provides detailed insight into the tight interplay of plenty of information carriers and cost factors. Supported by an innovative tailored visual representation scheme, the investigator is enabled to explore the options before conducting the experiment. With a practical showcase at hand, our computational study highlights the benefits of incorporating multiple information criteria apart from the costs, balancing the shortcomings of conventional single-objective experimental design strategies.

## Introduction

The successful design of tailor-made cell factories in the biotechnological and pharmaceutical industries needs firm understanding of the cellular functions and their underlying molecular mechanisms [1–3]. The key to get the most insight from an experiment is a careful experimental design (ED), precisely, the selection of experimental settings and measurements that harvest a maximum of information about the quantities of interest. In this context, there is growing interest in computer-aided modeling to guide the experimental choices [4–8]. Existing design techniques can be broadly divided into statistical approaches that strive to maximize the statistical confidence of inferring model parameters and information-theoretic approaches identifying informative designs to tackle the principal identifiability problem [9–11]. These techniques have been applied in various studies to deduce information-optimal settings to tackle the following questions:

- Which experimental-analytical settings are particularly informative? Which combinations are not worthy to be tried?
- How are design parameters related?
- How beneficial is the incorporation of additional data?

For quantify the information gain, several optimality criteria (or precision scores) have been suggested, all approximating the average statistical confidence of parameter estimates [12,13]. Typically, the information criterion to be used for the ED is decided *ad hoc*, since the most “suited” one is not known in advance. Favoring a single criterion in the planning phase, however, may well lead to improvements in that criterion at the expense of a decline of others, taking the risk to under-explore the design space and, eventually, deriving misleading design decisions [14]. To remedy this limitation, several information criteria could be simultaneously taken into account.

Although information remains a key criterion for science, it comes at a cost. In practice, resource-oriented considerations shape ED strategies, especially when experiments are extensive, time-consuming and labor-intense. For example when organisms exhibit slow growth rates, complicated experimental and sample preparation protocols are involved, or a large number of data has to be analyzed semi-manually. Consequently, from an economic point of view questions on the design of experiments are:

- What are major cost contributors? How are the total costs allocated?
- How broad are the expected information ranges?
- What information level can be achieved at a certain expense?

These questions motivate to explore the experimental settings to select those that are informative and offer this information in a cost-efficient manner. Clearly, such information-economic considerations need cost models that contemplate all major factors (equipment, replicates, analysis time, etc.) and relate them to the information carriers. For instance, increasing the number of samples positively affects the information gain while, at the same time, it raises the costs, implying that here the goals “maximize information” and “minimize cost” are incommensurable.

### Pareto-optimality and decision making

**Fig 1:**
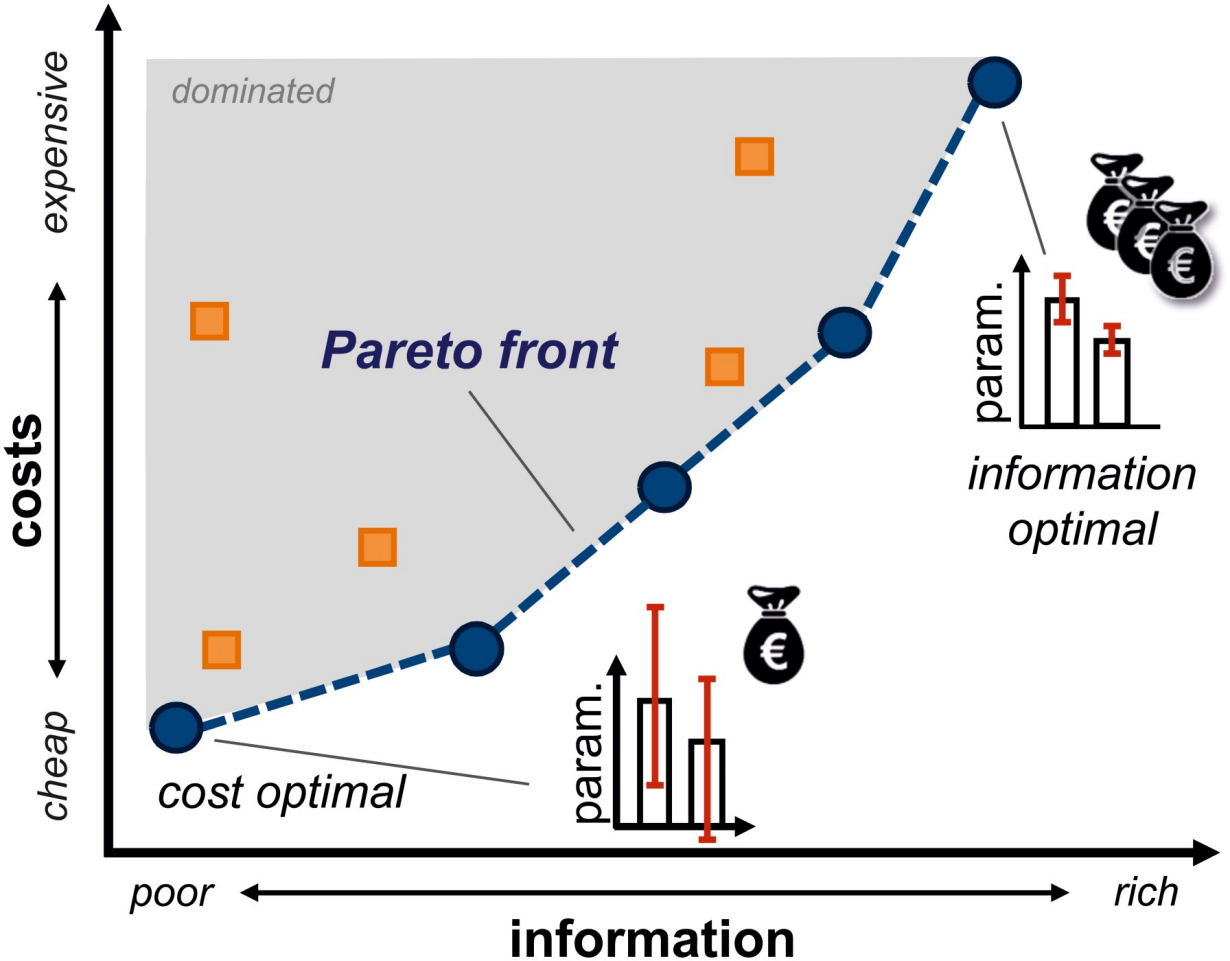
Schematic diagram of information-economic experimental design. The task is to determine trade-off designs that constitute the Pareto front (blue cycles, dashed blue line) in the objective space. The Pareto front trades-off information-rich, economic designs from sub-optimal, dominated solutions (orange squares, gray area). All solutions located on the Pareto front are considered to be equally good solutions of the MO-ED task.

That said, finding an informative, yet cost-efficient experimental setting out of the space of alternate designs is a nontrivial task: First, the space of options may be extensively large and secondly, several related, but potentially conflicting design objectives need to be optimized simultaneously. Here, a common solution concept is to optimize a weighted sum of the single criteria [6,15]. However, in real-world scenarios the objectives are hardly expressible in the same “currency” and appropriate weights to translate between them are not known before the experiment. Consequently, in scenarios where the ability to explore the whole space of design alternatives should be maintained, a fixed-weight solution cannot be utilized [16]. To overcome the limitations of weighted-sum single-objective approaches, the ED task can be casted into a multi-objective optimization (MOO) formulation [17]. Multi-objective (MO) ED comes with an important conceptual difference, compared to single-objective ED: When objectives are conflicting, instead of one specific solution, a whole set of—in terms of the objectives—equally good, compromise EDs is obtained where none of the designs is better than the others in terms of *all* criteria. These compromise EDs, denoted *Pareto-optimal* EDs, determine the *Pareto front* in the objective space (Fig 1). When the objectives are not in competition, a characteristic that cannot be known for real-world problems *a priori*, the MO-ED task degenerates to an ordinary ED problem.

The trade-off decision on the experiment is then made after examining the Pareto front and inspecting the related Pareto-optimal designs where (expert or newly available) information or preferences can be considered in addition. However, to keep track of more than a few relations is not only intrinsically challenging, it also calls for domain-specific solutions to interrogate the high-dimensional Pareto-optimal results and to support exploration and interpretation processes.

### Focus of this work

We present a universal computational methodology for the design of informative, yet cost-effective experiments. Our approach simultaneously optimizes many, potentially contradicting information and cost metrics rather than a single one, therewith generalizing traditional ED frameworks basing on the optimization of a single information criterion. To provide a visual means for result exploration of Pareto-optimal EDs in potentially high-dimensional design and objective spaces, we suggest a flexible solution using chord diagrams.

To exemplify our information-economic Pareto approach, the MO-ED framework is implemented for ^13^C metabolic flux analysis (^13^C MFA), which provides a computationally challenging test bed owing to its enormous design space and diverse cost factors. Equipped with the computational tools, the questions raised above were addressed by a comprehensive investigation featuring the fungus *Penicillium chrysogenum*. In particular, two different scenarios were studied. First, all analytical platforms commonly applied for ^13^C MFA were profiled with respect to their information-economic characteristics, using a single information criterion. The study revealed that the specific measurement information delivered by tandem mass spectrometry (MS/MS) cannot only increase flux information, but also enabled cost savings by the choice of cheaper tracers, emphasizing the potential of our approach. In the second scenario we investigated whether including more than one information criterion could provide a benefit for the decision process. Indeed, for the *P*. *chrysogenum* showcase a variety of additional Pareto-optimal designs were offered, unlocking informed decision making. In particular for, but not limited to, the domain of ^13^C MFA our findings show that the use of several criteria balances shortcomings of conventional ED strategies and offers additional flexibilities for the experimenter, thus providing a methodology of direct practical relevance.

## Methods and models

### General framework for multi-objective experimental design

Planning cost-efficient, informative experiments requires finding the “best” experimental-analytical trade-offs that, on the one hand, maximize the information gain, possibly in view of different information facets, while, on the other hand, keep the associated costs to a minimum. Consequently, two formal ingredients are needed:

1. Information quantifiers which measure the (un)certainty of the unknown parameters of system model under study (here the system model is a mimic of the real experiment). Several criteria have been suggested which are based on the variance of the unknown model parameters. *Information gain* then refers to the improvement in these criteria values by making a different, “better” choice of the experimental settings (i.e., the inputs of the system model).
2. A cost model of the thought experiment, which collects all factors that contribute to its overall expenses.

Employing these criteria in the selection procedure of the ED formally amounts to a multi-objective optimization (MOO) problem:

$$\begin{array}{l} \text{max}{\;}_{\alpha \in \Omega }\;\Phi \left(\alpha ,\theta \right)\\ \;s.t.\;g\left(\alpha ,\theta \right)\geq 0\\ \;h\left(\alpha ,\theta \right)=0\\ \;l\leq \alpha \leq u \end{array}$$ (1)

where the objective vector **Φ** is composed of a set of information and (negated) cost criteria. The objective vector is a function of the design variables ***α***, selected from the space **Ω** of feasible designs. Remaining design parameters, which are constant, are collected in the vector ***θ***. Furthermore, the bounded design variables may be subject to inequality and equality constraints.

Solving the MOO problem (1) means to find the set of all trade-off design solutions α* that minimize the objectives in **Φ** without being dominated by another solution [18]. Here, a specific design ***α***_1_ dominates another one ***α***_2_, if (and only if) ***α***_1_ is at least as good as ***α***_2_ in all objectives and better with respect to at least one, formally expressed by Φ_*i*_ (***α***_1_) ≤ Φ_*i*_ (***α***_2_), ∀*i* and ∃*j*: Φ_*j*_ (***α***_1_) < Φ_*j*_ (***α***_2_) (gray shaded area in Fig 1). The set of all non-dominated solutions is referred to as *Pareto-optimal design set*, and the corresponding achievable objective values are called *Pareto front*.

Clearly, the concrete formulation of the MOO problem depends on the particular application case, namely the underlying system model and the peculiarities of the experimental settings. In this work, we selected a use-case from the domain of ^13^C metabolic flux analysis (MFA), which is arguably the most expensive type of ‘omics’ technology, featuring dozens of design variables. Before introducing the information and intricate cost models as well as the analysis of Pareto-designs in high dimensions, the essential background to the application field is provided, in particular the formulation of the system model.

### ^13^C metabolic flux analysis

Intracellular reaction rates (fluxes) describe the trafficking of metabolites which emerges as the final outcome of all catalytic and regulatory processes acting within living cells [19]. Here, the reactions within a biochemical network are characterized by a pair of flux values, net and exchange fluxes [20], to express the respective proportions of material transported between the reaction’s educts and products. At steady-state, the in- and outflows of each intermediate metabolite are assumed to be constant and mass balanced, yielding the stoichiometric equation system for the flux vector **v**:

$$S\cdot v=b,\;C_{ieq}\cdot v\leq c_{ieq}$$ (2)

with the stoichiometric matrix **S** and the vector **b** containing the extracellular rates (substrate uptake, product formation or effluxes leading to biomass accumulation), accessible through extracellular concentration profiles and biomass quantification. In addition, the fluxes may be constrained in their allowable value range owing to physiological knowledge.

Since metabolic networks contain parallel paths and cycles, fluxes are not uniquely determined by Eq (2), at least not without additional assumptions. The indeterminacy implies that the flux vector **v** can be parametrized through a certain (non-unique) sub-set of fluxes, the so called *free fluxes* **v**^*free*^ [20]. The dimensionality of the vector **v**^*free*^, i.e., dim(**v**) − rank(**S**), is referred to as *degrees of freedom* (DoF). To resolve the DoFs, carbon labeling experiments (CLEs) are conducted. In a CLE, isotopically labeled carbon sources, like [1-^13^C] glucose enriched with a ^13^C isotope at the first position of the carbon backbone, are fed to the cells. The labeled substrate is taken up by the cells and distributed through the metabolic pathways to all intracellular metabolites, where it gives rise to characteristic labeling enrichment patterns. Thus, the labeling patterns are the convoluted result of the routes, the ^13^C labeled substrate takes, as well as the underlying metabolic fluxes. In isotopic steady-state ^13^C MFA, as used in this work, intracellular free fluxes are inferred from the equilibrated labeling patterns and external rate measurements by means of a computational flux fitting procedure that minimizes the least-squares error between observed measurements and those that are simulated by a computational network model [21].

For the model, carbon atom transitions have to be specified for each reaction step describing the fate of each carbon atom from the reactions’ educt to its corresponding product. Mass balancing of the intracellular isotopic forms then yields a high-dimensional nonlinear algebraic equation system that relates the steady-state labeling state **x**, the administered labeled tracer mixture **x**_*inp*_, and the free fluxes **v**^*free*^ [20]. Given **v**^*free*^ and **x**_*inp*_, the vector of steady-state labeling states **x** (represented as isotopomers, cumomers, EMUs, or similar [20,22,23]) is uniquely determined by [24]:

$$x=x\left(v^{free},x_{inp}\right)$$ (3)

Note that CLEs that only differ in the tracer mixture are covered by the same formalism through duplication of the network model and equating the free fluxes.

**Fig 2:**
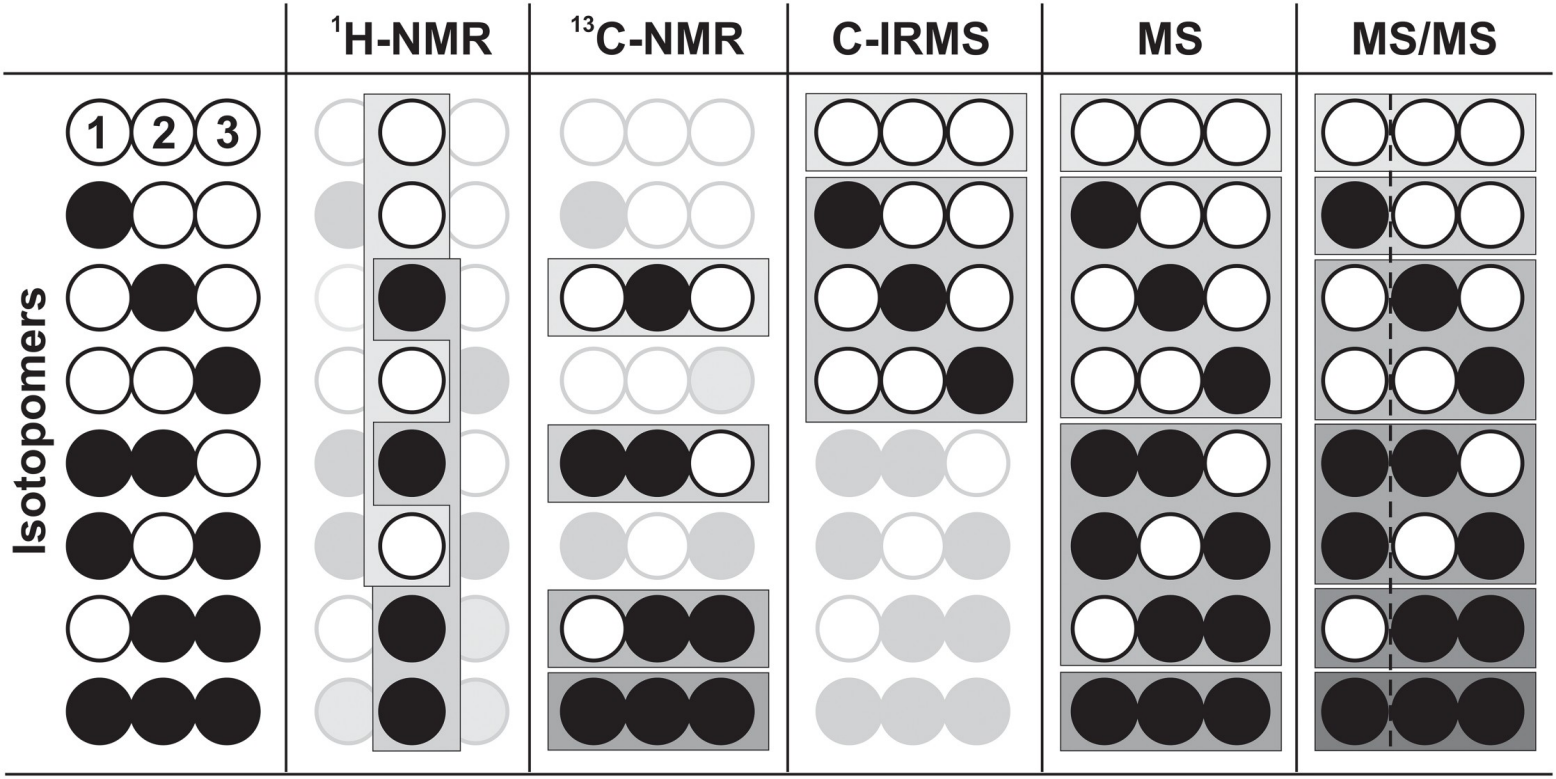
Characteristic measurement information of analytical platforms for a C3 metabolite (C_1_-C_2_-C_3_). The techniques yield specific measurement groups composed of sub-sets or linear combinations of isotopomers as indicated by gray boxes. As an example, ^1^H-NMR and ^13^C-NMR measurements for C_2_, C-IRMS for the total fraction of unlabeled and one-labeled carbon content, MS for the intact precursor ion and MS/MS measurement for the combination of complete precursor and C_2_-C_3_ fragment ion, delivering in effect positional information, are shown. For ^13^C MFA only the carbon backbone of the metabolites and metabolite fractions are relevant. Further details are found in S1 Text.

The full system-wide labeling state **x** is not accessible by any current measurement technology. What can be observed are linear combinations of (relative) abundances for some of the intracellular metabolites, such as mass isotopomer distributions or positional enrichments. Fig 2 shows characteristic sets of observations, henceforth denoted *measurement groups*, for the analytical platforms employed in the field of ^13^C MFA.

All measurement groups available for an analytical device are organized in the measurement matrix $M_{meas}^{dev}$ that, owing to Eq (3), allows to simulate the measurement vector **η**:

$$\eta =M_{meas}^{dev}\cdot x\left(v^{free},x_{inp}\right)$$ (4)

which mimics the real measurements up to normalization to percentage scale [25]. Examples for measurement matrices are given in S1 Text. Real measurements are unavoidably affected by noise. In the context of ^13^C MFA, measurement noise is assumed to be independent, unbiased, additive, and normally distributed with expectation **0** and standard deviation $\sigma_{meas}^{dev}$, as represented by the measurement covariance matrix $\Sigma_{meas}^{dev}$ [26]:

$$\Sigma_{meas}^{dev}=\text{diag}\left(\sigma_{meas}^{dev}\right)$$ (5)

Since in the CLE’s planning phase real measurements are absent, from which measurement standard deviations $\sigma_{meas}^{dev}$ can be derived, measurement error models need to be formulated, relating the measurements with their associated errors. For labeling measurements empirical rule-of-thumb approximations of the measurement precision have been derived for specific analytical setups. For instance, Crown et al. propose a precision of 0.4 mol% for their GC-MS setup targeting proteinogenic amino acids [27]. In general, labeling errors depend on the measurement technique, the instrument, the analytic protocols, they can vary between organisms, analytes and the degree of label incorporation [28]. To arrive at realistic error approximations that allow for a fair comparison of the analytical platforms, measurements and their standard deviations were collected from published studies featuring different organisms, platforms and various labeling contents. In total, more than 900 data points for six analytical platforms, namely GC-MS, LC-MS, LC-MS/MS, ^13^C-NMR, ^1^H-NMR, and GC-C-IRMS were extracted (S1 Text). For all analytical platforms, similar to the approach by Dauner et al. for ^13^C-NMR [29], a regression line was fitted to the respective data set, yielding device-specific linear measurement error models. These analytics-related error models provide empirical standard deviations $\sigma_{meas}^{dev}$ for any given measured vector **η**:

$$\sigma_{meas}^{dev}\left(n_{rep,meas}^{dev}\right)=a\left(n_{rep,meas}^{dev}\right)\cdot \left(b_{1}^{dev}\cdot \eta +b_{2}^{dev}\right)$$ (6)

where $b_{1}^{dev},b_{2}^{dev}$ are the device-specific regression coefficients (S1 Text). Generally, by increasing the number of repetitions $n_{rep,meas}^{dev}$ (i.e., technical replicates), the error estimates are believed to become more reliable. This is accounted for in the error models (6) by a scaling factor (*a*) which tends to 1 for the case of many repetitions (see S2 Appendix for details).

### Covariance-based information measures

Several statistical approaches have been developed to predict the approximate amount of information to be derived from the planned CLE or CLE series. When some pre-knowledge on the expected flux map ${\hat{v}}^{free}$ is available (which we assume in this work), a widely adopted local information measure is the Fisher information matrix (FIM) [9,13,26]:

$$FIM={\left({\frac{\partial \eta }{\partial v^{free}}|}_{{\hat{v}}^{free}}\right)}^{T}\cdot \Sigma_{meas}^{dev}\cdot {\frac{\partial \eta }{\partial v^{free}}|}_{{\hat{v}}^{free}}$$ (7)

whose inversion yields the flux covariance matrix:

$$Cov\left({\hat{v}}^{free},\alpha \right)=FIM^{-1}$$ (8)

which depends on the design point (${\hat{v}}^{free}$) and the design parameters (***α***). As a precondition for stable numeric calculation of the flux covariance matrix, the FIM needs to fulfill two conditions [30]: First, its minimal singular value λ_min_ (**FIM**) needs to be larger than a threshold:

$$\lambda_{\text{min}}\left(FIM\right)>\tau_{1}>0$$ (9)

and secondly, its condition number has to be bounded:

$$\text{cond}\left(FIM\right)<\tau_{2}<\infty$$ (10)

The fulfillment of the conditions (9) and (10) implies that the standard deviations of the free fluxes—as represented by the main diagonal of the covariance matrix—remain bounded and, thus, the flux vector is said to be *statistically identifiable*. First, it should be remarked, that this is a slightly stronger variant of practical identifiability as defined by Raue et al. in [31] and secondly, statistically identifiable fluxes are *per se* structurally identifiable [32]. If either one of the conditions (9) and (10) is violated, fluxes causing the violation have to be excluded from the FIM. Eventually, this leads to models that vary in terms of their DoFs, a fact which needs careful treatment when comparing different experimental setups with respect to their information content.

**Fig 3:**
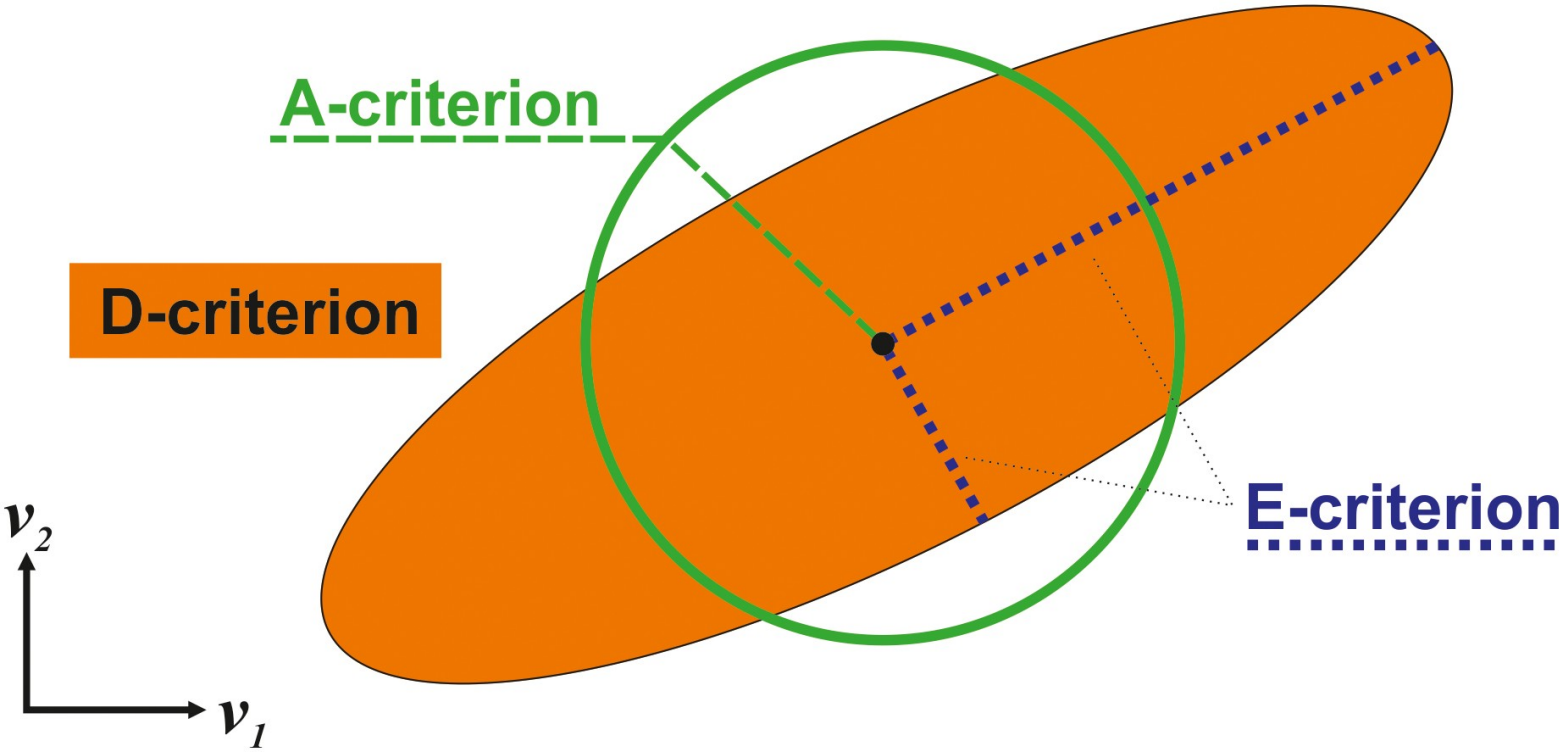
Geometrical interpretation of covariance based information criteria.

For quantifying the information content of a CLE several information quality criteria have been proposed that aggregate the covariance matrix to a single number [9,12,13]. The most prominent ones are the determinant (D), the average-variance (A), and eigenvalue (E) criteria. Ultimately, all these criteria provide a means for the shape of the confidence ellipsoid in the vicinity of a given design point (${\hat{v}}^{free}$ in our case), each emphasizing particular geometrical aspects [12] (Fig 3).

For example, the D-criterion strives to minimize the volume of the confidence ellipsoid (or the geometric mean of the flux confidence intervals):

$$\Phi_{D,p}=\sqrt[{2\cdot p}]{\text{det}\left(Cov\right)}$$ (11)

with *p* the dimension of **Cov** (with arguments omitted for brevity) while the A-criterion aims to minimize the diagonal of the smallest bounding box that contains the confidence ellipsoid (or the arithmetic mean of the flux confidence intervals):

$$\Phi_{A,p}=\text{trace}\left(Cov\right)/p$$ (12)

Hence, the A-criterion is expected to provide designs that are more robust against flux correlations than those based on the D-criterion. Notice that the explicit consideration of the dimension *p* of the covariance matrix in the formulation of criteria (11) and (12) intends to make the criterion values comparable for models differing in the number of free fluxes. In contrast, the E-criterion:

$$\Phi_{E}=\lambda_{\text{max}}\left(Cov\right)/\lambda_{\text{min}}\left(Cov\right)$$ (13)

constitutes a dimension independent measure that strives to improve worst case designs by preventing the Fisher matrix from becoming singular. Besides these quantitative information measures, an obvious quality criterion is the number of free fluxes that can be statistically identified by the ED setting, expressed by:

$$\Phi_{DoF}=\text{dim}\left(Cov\right)$$ (14)

With these information measures at hand, the information gain of a ^13^C MFA study can be influenced by the targeted selection of the input mixture compositions (**x**_*inp*_), the measured groups observable by the analytical device (${\text{M}}_{meas}^{dev}$), as well as the corresponding measurement errors ($\sigma_{meas}^{dev}\left(n_{rep,meas}^{dev}\right)$), i.e., the interval in which the true measurements are believed to lie in to a certain probability, triggered by the number of repeats.

### ED approaches in ^13^C MFA revisited

The choice of isotopically labeled substrate species, either in pure form or in a mixture, dictates the emerging labeling states of the observable metabolites and therefore significantly impacts flux information [25,33]. Several recent field studies yielded information-optimal tracers in a variety of biological systems and give evidence for a high diversity of flux standard deviations depending on the substrate or substrate mixture. For instance, Walther et al. showed that [1,2-^13^C]-labeled glucose and mixtures of [3-^13^C]- and [3,4-^13^C]-glucose increase statistical identifiability when used with fully labeled glutamate for lung cell carcinoma [34]. Crown et al. identified [3,4-^13^C]- and [2,3,4,5,6-^13^C]-labeled glucose to be favorable for elucidating reaction rates in the oxidative pentose phosphate pathway (PPP) and pyruvate carboxylase flux, respectively, based on a small scale network with two free fluxes [35]. Later on, the same group determined [1,2-^13^C]-, [5,6-^13^C]-, and [1,6-^13^C]-labeled glucose as best single tracers for *Escherichia coli* wild type [36]. A study of Metallo et al. suggested [1,2-^13^C]-labeled glucose to be the optimal commercial tracer for most fluxes in the PPP and glycolysis in lung carcinoma cell lines while uniformly labeled glutamine provided optimal results for tricarboxylic acid cycle (TCA) fluxes [37]. In theoretical studies, [3,4,5,6-^13^C]-glucose and [2,3,4,5,6-^13^C]-glucose resulted to have to best information yield in plants and mammalian cells, respectively [38,39]. Araúzo-Bravo et al. calculated mixtures of 70% unlabeled, 10% U-^13^C- and 20% [1,2-^13^C]-labeled glucose to be optimal for flux determination in the cyanobacterium *Synechocystis* sp. PCC6802 [40]. Schellenberger et al. applied a Monte Carlo sampling technique for experimental tracer design to a large-scale *Escherichia coli* network and found positional [1-^13^C] or [6-^13^C] labeled glucoses to be superior over a commonly used mixture of 20% uniform and 80% unlabeled glucose [41]. Here, unusual multi-positional labeling, in particular [5,6-^13^C]-, [1,2,5-^13^C]-, [1,2-^13^C]-, [1,2,3-^13^C]-, and [2,3-^13^C]-glucose, resulted in a higher identifiability than single positional labeling. Nonetheless, no single tracer has been found to outperform all others, an observation which was experimentally confirmed by Crown et al. comparing the outcome of 14 CLEs in *Escherichia coli* [27]. Importantly, the studies also disclosed a high redundancy in the measurement data, meaning that not all observations effectively contribute to the information gain, although they come at a certain cost. One option to raise flux identifiability that recently has become compelling through advances in lab standardization and miniaturization [42], is the conduction of multiple independent, so called *parallel* CLEs under identical conditions, each with a different tracer [43] (and references therein). Concurrent fitting of all labeling patterns with a single model obviously increases the measurement-to-flux ratio but, at the same time, also the measurement redundancies. Still, in these and other theoretical and practical studies a part of the fluxes remained non-identifiable [27,44]. Interestingly, a study of Bouvin et al. [45] exemplified, also using a MO-ED approach, that it is indeed possible to find CLEs with comparable information content, but considerably different tracer costs.

In contrast to the work on tracer design, measurement setups have not yet been the target of ED in the field of ^13^C MFA. The primary analytical methods that are employed are NMR and MS. For both, analytical devices differ not only in the principally observable metabolite/isotopomer spectrum, achievable fragmentation patterns (Fig 2) and the measurement accuracy and sensitivity, but also in terms of analysis speed/throughput, and purchase/maintenance costs (S1 and S2 Text). Since comparative investigations on the inter-platform information content of CLEs for ^13^C MFA are scarce, in essence, it is still an open question which analytical platform delivers maximal flux information and what the information benefit of multiple-device applications is compared to single-device usage.

### A low-level cost model for ^13^C MFA

For considering economic aspects, the cost contribution of the isotopically labeled substrates, the experimental setup and the analytical technologies are to be specified. Additionally, not only the measurement time on the device, but also spectra evaluation and proofreading processes, possibly with the need for manual post-correction, contribute to the workload. Consequently, such direct and hidden factors play a part in the overall CLE costs. Till now, if at all, only ^13^C labeled tracers have been considered in CLE costs examinations while further experimental-analytical efforts were neglected so far (see e.g. [45]), meaning that a fine-grained cost function which relates all cost factors to the design parameters has to be set up. The overall cost function of a ^13^C MFA study is composed of three parts, the experimental, the analytical, and the modeling part. However, the modeling costs such as setting up an adequate model, working through the ^13^C MFA workflow, calculating and interpreting results etc., heavily depend on the use case and are therefore not considered in the following.

#### Costs of the labeling experiment *C*_*CLE*_

Experimental costs of a single CLE are composed of two parts: the costs of the isotopic substrate mixture and the costs for the technical setup and execution of the experiment, while taking culture volume, substrate concentration and labeling duration into account. Costs for all tracers are collected in the substrate cost vector **C**_*inp*_. For a specified mixture composition, represented by the vector of tracer fractions **x**_*inp*_, the total substrate costs can be readily derived.

The technical setup of the experiment *C*_*exp*_, i.e., consumables, media components etc., and the costs for wage payment (*C*_*work*_) contributes with a cost offset. Here, a given working time *t*_*work*,*exp*_ for setting up and controlling the experiment is considered. All in all, the experimental cost contributions of one CLE considered in this work are:

$$C_{CLE}=x_{inp}^{T}\cdot C_{inp}+C_{exp}+t_{work,exp}\cdot C_{work}$$ (15)

where the elements of the tracer fraction vector **x**_*inp*_ fulfill

$$\sum_{i}x_{inp,i}=1.0,\;x_{inp,i}\in \left[0,1\right],\forall i$$ (16)

#### Analytical costs $C_{ANA}^{dev}$

Device-specific analytical costs $C_{ANA}^{dev}$ associated with a CLE depend on the devices’ prices, the acquisition method applied, the number of samples measured and the effort of spectra analysis. In this study, the costs of the instrument are allocated to a time span of five years. With that, the cost per time unit of machine usage is calculated (assuming that the device is operated at full load) providing the basic price for measuring a single sample (*C*_*sample*_). The CLE’s acquisition costs are then derived by multiplying this value with the number of samples *n*_*samples*_ taken in the CLE.

Notice that, of these collected samples, not necessarily all spectra are actually considered in an ED, meaning that single measurement groups may remain unevaluated and, thus, do not contribute to the analytical costs. On the other hand, each measurement group can be evaluated up to *n*_*samples*_ times, which increases the analysis times and the costs proportionally. Here, it is reasonable to assume that the groups consisting of a certain number of peaks, $n_{mgroup,peaks}^{dev}$, are evaluated *en bloc*. In this way, the impact of repeated measurements, collected in the vector $n_{rep,meas}^{dev}$, on the flux standard deviations is coupled with an increase of effort for sample analysis and peak integration. The costs of the peak evaluation then scales linearly with the time needed for a single spectra evaluation, *t*_*work*,*ana*_, and the wage payment. Summarizing, the device-specific analytical costs of one CLE are given by:

$$C_{ANA}^{dev}=\underset{aquisition}{\underset{︸}{n_{rsample}\cdot C_{sample}}}+\underset{peak\;evaluation}{\underset{︸}{\left(\sum_{mgroup=1}^{n_{mgroups}}n_{rep,mgroup}^{dev}\cdot n_{mgroup,peaks}^{dev}\right)\cdot t_{work,ana}\cdot C_{work}}}$$ (17)

#### Device-specific total cost criterion

Finally, the expected total CLE costs **$\Phi_{Costs}^{dev}$** are expressed as a function of the design parameters, namely the ^13^C-labeled substrate mixture and the contributing measurement groups and their biological and technical repeats:

$$\Phi_{Costs}^{dev}=\overset{n_{cle}}{\underset{cle=1}{\sum }}\left(C_{CLE,cle}\left(x_{inp,cle}\right)+C_{ANA,cle}^{dev}\left(M_{meas,cle}^{dev},n_{rep,meas,cle}^{dev}\right)\right)$$ (18)

where the subscript “*cle*” indicates the affiliation of the design parameters to the *cle*^th^ experiment. The design constants, chosen according to the experimental-analytical CLE setup, are omitted for brevity.

### Solution of the MO-ED problem

Together, the information and cost criteria Eqs (11)–(14), (18) make up the set of goal functions out of which the objective vector **Φ** of the MO-ED problem Eq (1) is composed. The design vector ***α*** is subject to inequality and equality constraints such as the invertibility conditions on the Fisher matrix (9) and (10), constraints for weights, as well as constraints imposed by reasonable practical resource considerations, e.g., a maximum number of replicates. Since exact handling of integer-valued replicate numbers would result in NP-complete mixed integer nonlinear optimization problems [46], the optimization problem is relaxed by allowing the replicates to take non-integer values. The solution for the relaxed problem is then “rounded” to integers. The full formulation of the MO-ED problem is given in S2 Text.

**Fig 4:**
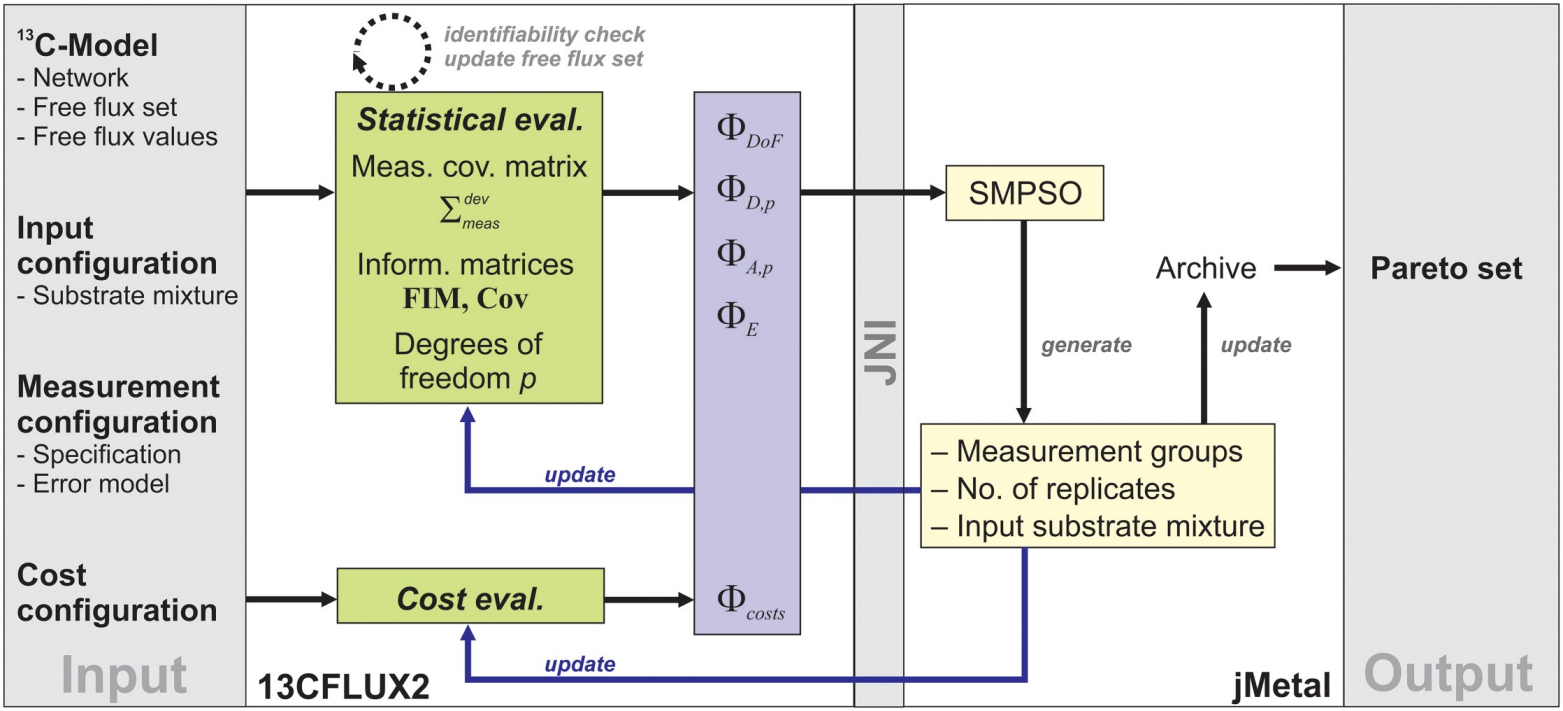
Diagram showing the coupling the 13CFLUX2 simulator and the jMetal library.

Solving Eq (1) means to numerically approximate the (potentially infinite) design set α* by an ensemble of Pareto-optimal results [47,48], optimally uniformly distributed covering the whole Pareto front. Particularly successful among these algorithms with respect to convergence and extensity of Pareto front approximation are those based on Particle Swarm Optimization (PSO) with update mechanisms to ensure that the solution ensemble is well-dispersed over the front [49]. For this work, the jMetal (Metaheuristic Algorithms in Java) library, a suite of state-of-the-art MO algorithms is utilized [50]. jMetal is linked to the high-performance ^13^C MFA simulator 13CFLUX2 [51] via a Java Native Interface (JNI) that enables jMetal to call 13CFLUX2 methods. While 13CFLUX2 is used to evaluate the objectives and takes care of the feasibility of the design parameters, the solution of the MO problem is steered by jMetal routines (Fig 4).

Initially, all experimental, analytical and simulation settings as well as the network model (incl. free flux set and flux values), measurement error models and input species with their respective costs are specified. Depending on the measurement selection proposed by jMetal, the measurement error model is evaluated for the suggested mixture composition in 13CFLUX2 while also taking the number of replicates into account. In turn, statistical flux identifiability is tested and, if one of the invertibility criteria fails, the free flux set is adapted in an iterative procedure: Non-identifiable fluxes are eliminated one-at-a-time by constraining them to their nominal values beginning with the worst determined one, eventually providing the effective number of statistically identifiable fluxes, i.e, *p*. From the resulting covariance matrix the local information measures *Φ*_*D*,*p*_ etc. are calculated. Furthermore, the expected CLE costs $\Phi_{Costs}^{dev}$ are evaluated according to the cost model, given the experimental specification. The objective values are then passed to jMetal, calling the SMPSO algorithm (the rationale for the choice of SMPSO and its parameters is given in S2 Text).

Starting with an initial population created randomly, the swarm is evolved driven by polynomial mutation rules that trigger the choice of the design parameters. In this way, new swarm candidates are proposed out of which Pareto-optimal solutions are selected. The best Pareto solutions are stored in an archive where for each iteration the crowding distance is used to decide which swarm individuals remain in the archive to achieve maximal coverage of the designs. For the newly generated swarm members, measurement values are predicted *in silico* according to the ^13^C MFA model using 13CFLUX2 and the corresponding standard deviations are derived from the associated error models. This process cycle is restarted with the next generation of particles until the stopping criterion (i.e., maximum number of generations) is reached. Finally, the archive containing the (best known) Pareto-optimal ensemble is returned and subjected to visual analysis.

### A domain-specific visualization model for inspecting Pareto-optimal designs

Having the Pareto front approximation at hand, the final step of ED involves decision making on the next experiment. In the context of ^13^C MFA, decision making means to find the most suited experimental-analytical setup out of the range of analytical platforms, input mixture compositions, sets of observable metabolites and replicate numbers. These quantities have different contextual meanings, scales and importance, in the sense of affecting the objective values. Hence, the visual interpretation of MO-ED results faces two challenges:

1. The curvature of the Pareto front (approximation) in the objective space, i.e., its spread, diversity, shape, location, and distribution, needs to be accessible, both to verify solution quality as well as for the purpose of trade-off analysis. Because there is no general Pareto-dominance preserving mapping from a higher-dimensional space to a lower-dimensional one [52], the task of visualizing Pareto fronts in more than three dimensions is a MO problem in its own right [53].
2. The Pareto front in the objective space is to be linked with the Pareto-optimal designs in the design space which is made up by all possible substrate mixtures and measurement configurations. Since, however, the Pareto-optimal solutions are spread within the high-dimensional space, these sets need to be adequately compressed before visual exploration.

**Fig 5:**
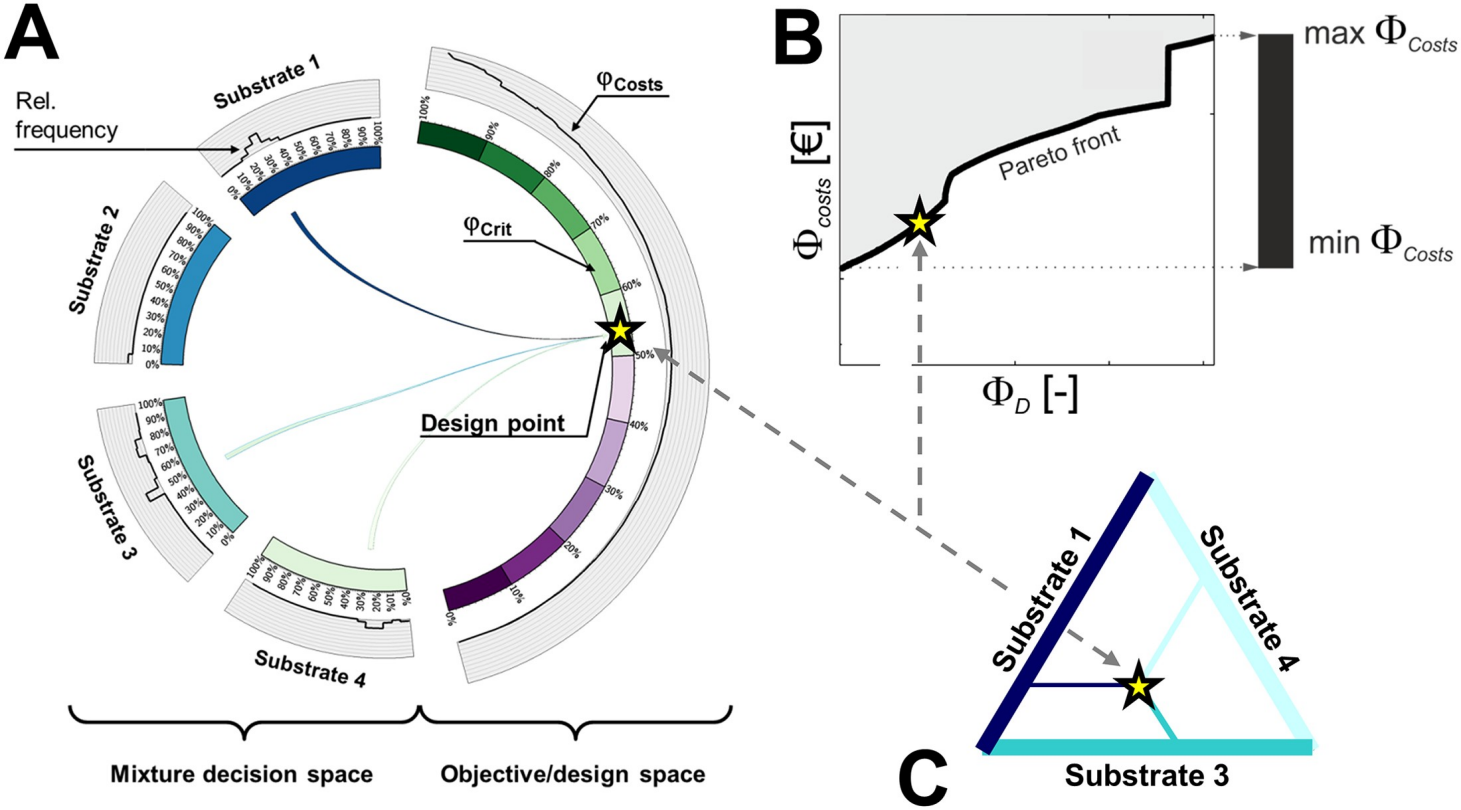
Visual elements for the interpretation of MO-ED results in the context of ^13^C MFA. A: Chord diagram linking designs and objective (circular node elements) by inlying chords, here for the case of two objectives $\Phi_{D,p}^{},\Phi_{Costs}^{dev}$ (right segment) and four input substrate species (left segments). An example is given with three substrates contributing to a design, roughly 25% Substrate1, 0% Substrate2, 50% Substrate3, and 25% Substrate4. The proportions in which the substrate species contribute is indicated by percentages. In addition, the (relative) frequency with which a certain proportion of a substrate species is proposed among the Pareto-optimal solutions is displayed by histograms located at the left outer bands. Information and cost values are scaled to the range of 0–100%. The graphic is created with Circos [54] (www.circos.ca). B: 2D scatter diagram representing the Pareto front with the dominated objective region being grayed-out. The slope of the Pareto front reflects the progressive increase in cost per information gain. The region of the Pareto front in the vicinity of a jump (green arrow) reveals that a higher information value requires the addition of at least one costly input substrate or measurement group which leads to a large cost increase. To the contrary, densely populated flat Pareto fronts indicate that CLE costs can be tuned well. The black bar on the right indicates the overall cost spread. C: Ternary triangles are commonly used in ^13^C MFA to represent mixture designs with three tracer species. The dashed lines relate the design point (yellow star) on the CD and the Pareto frontier with the mixture composition.

To tackle these challenges a tailor-made visual interpretation workflow was created (Fig 5). The workflow is composed of three modules, applying different information visualization techniques that (a) allow for visual assessment of the Pareto front, (b) relate the objective with the most important elements of the design space, and (c) compress presentation of the less important design elements.

#### Pareto front visualization with scatter plots

For the visualization of Pareto frontiers, scatter plots are arguably the most common scheme, although they do not scale well with higher dimensions. In moderate dimensions, as it is the case in the context of this work, 2D scatter plots are still capable of showing the distribution of the objective values and conveying the shape of the Pareto front (Fig 5B).

#### Linking Pareto front and design space by chord diagrams

Keeping the interrelation between Pareto fronts and sets is central for decision making. Here, the challenge is to provide a high-level visual result summary that informs about common features and differences when comparing MO-ED results determined for different analytical platforms. For this purpose chord diagrams (CD) are used. CDs are circular graphs consisting of segmented nodes and inlying “chords” relating entities located within the node segments by arcs. The CD in Fig 5A shows an example how a specific tracer composition is related to the information and cost criterion values through arc connections. One color is assigned to each tracer. The proportion with which the tracer contributes to the mixture determines the origin of the arc while its end is given by the associated criterion value. Furthermore, the circular node segments are decorated with additional information supporting result interpretation.

Substrate mixtures are of predominant interest in ^13^C MFA and central for design decisions. Therefore, input species were selected for the node segments arranged on the left hemicycle while the Pareto-optimal criteria values are summarized by node segments located on the right hemicycle. Here, the criteria node segments are arranged as nested bands, depending on the number of objectives (Fig 5A for the case of two objectives and four input species). Note that while in the case of two objectives related objective values can be neatly aligned, this is no longer to be the case for three and more objectives that contradict each other (S5 Text). The chords connecting left and right hemicycles represent an overlay of all substrate mixture compositions that are proposed as trade-off designs. The relative frequencies with which a specific input species proportion are proposed among all Pareto solutions is reported as histograms located at the outer band of the respective substrate.

#### Compressing input and measurement designs

CDs yield a visual summary, overviewing the range of Pareto-optimal mixtures and their relation to the Pareto front. When it comes to the step of decision making, the practitioner is interested in representative classes of the mixture compositions. Particularly, in cases where the proposed designs are spread over almost all possible mixtures it is important to guide the experimenter’s choice. Therefore, the Pareto-optimal mixtures are pruned and scrutinized by grouping them in line with their similarity. Precisely, hierarchical clustering with the Euclidean distance metric is applied accounting for the mixture compositions, the information value, and the costs. This yields a sequence of nested tracer sets, henceforth denoted substrate clusters which are displayed by dendrograms. Furthermore, in the special case of a three-component substrate cluster, the mixture can be conveniently represented by a ternary triangle (Fig 5C) [25]. Finally, for each emerging substrate cluster replicate numbers for each measurement group are presented by histograms (S4 and S5 Text).

## Results

We developed a framework for information-economic design of CLEs, which we now put into practice.

### Case study: Design of a CLE with *P*. *chrysogenum*

*P*. *chrysogenum* is the primary microbial cell factory for the production of penicillin G and V. Although metabolic engineering strategies have led to strongly improved production efficiencies, the yields of *P*. *chrysogenum* are still far below the theoretical maximum [55]. In this situation, ^13^C MFA is a powerful technique to detect pathway bottlenecks and to guide metabolic engineering efforts. Therefore, this case study explores the Pareto-optimal experimental design spaces in an industrially relevant setting.

#### Experimental setup

A scale-down chemostat setup with glucose as sole carbon source was assumed: Bioreactor working volume of 250 mL, 20 g/L glucose, and a conservative labeling period of six residence times [56]. Sample volumes were sufficient to generate up to a maximum of ten technical replicates per sample. The CLE was conducted once.

#### Tracers

Sales prices of ten commercially available glucose species (cf. Table 1) were inquired with a purity of 99%, except for unlabeled [^12^C]-glucose that has a purity of 98%. Prices range from 0.30 EUR/g for unlabeled glucose to 1293.00 EUR/g for [5-^13^C]-labeled glucose.

**Table 1:** Design parameters for MO-ED case study.

**Tracers:**
[1-^13^C]-, [2-^13^C]-, [3-^13^C]-, [4-^13^C]-, [5-^13^C]-, [6-^13^C]-, [1,2-^13^C]-, [1,6-^13^C]-, [U-^13^C]-glucose
**Labeling measurement configurations** (#metabolites, # meas. groups, #peaks): GC-MS (amino acids): 14, 32, 169 LC-MS (central carbon intermediates and amino acids): 34, 34, 197 LC-MS/MS (central carbon intermediates and amino acids): 34, 34, 287 ^13^C-NMR (amino acids): 17, 50, 156

#### Measurement configuration

Measurement setups were collected from the literature and used for the generation of the measurement error models (Table 1).

#### Cost function

Platform-specific cost functions were compiled for all analytical platforms as described before. Details on the parameters of the cost functions are documented in S2 Text.

#### ^13^C MFA network model

A metabolic network model of *P*. *chrysogenum* was set up based on existing knowledge [57–59] such that the measurement configurations of the analytic platforms under consideration were covered. The model comprises the central metabolic pathways, namely glycolysis/ gluconeogenesis, the pentose phosphate pathway (PPP), the TCA, anaplerotic reactions, amino acid biosynthesis as well as the penicillin synthesis pathway. It is composed of 73 metabolites and 124 reactions, 25 thereof reversible. Reactions were allocated to four compartments (extracellular, cytosolic, mitochondrial, peroxisomal). In total 117 reactions were supplemented with carbon atom mappings. The model has 34 free fluxes (12 net, 22 exchange [20]). A reference flux distribution was chosen after calibrating the model with in-house data. The specification of the *P*. *chrysogenum* network model including carbon atom mappings, reaction directionalities, flux values and constraints, as well as extracellular rate measurements used in this work is given in the supplementary information along with a graphical representation of the network (S3 Text).

Using a typical reference tracer mixture, 60% [1-^13^C]-, 20% [U-^13^C]-, and 20% [^12^C]-glucose, the set of statistically identifiable fluxes was determined for each analytical platform: LC-MS and LC-MS/MS measurements statistically identified 26 of the free fluxes (76%). GC-MS and ^13^C-NMR were capable to statistically determine 24 and 22 free fluxes, respectively (71 and 65%) whereas the majority of free fluxes remained non-identifiable for GC-C-IRMS and ^1^H-NMR. These platforms were only capable to determine 32 and 15% of the free fluxes, respectively. Statistically (non-)identifiable fluxes are documented in S3 Text. Given the large number of measured metabolites and isotopic patterns present in the MS/MS mode, it is not surprising that LC-MS/MS performed best in the category “number of statistically identifiable fluxes”, interestingly sharing the first position with LC-MS. Due to the much lower number of statistically identifiable fluxes as compared to the other four platforms, ^1^H-NMR and GC-C-IRMS were not further examined in this work.

To make an analytical platform comparison between GC-MS, LC-MS, LC-MS/MS, and ^13^C-NMR as fair as possible, the metabolic model was pruned: Those fluxes that are statistically non-identifiable for any of the four platforms were constrained to their reference values, thereby eliminating their contribution to the information matrices. This step reduced the degrees of freedom of the *P*. *chrysogenum* model from 34 to 21 (10 net, 11 exchange free fluxes). The resulting reduced model constituted the basis for the MO-ED studies. Notice, that although the reduced model is statistically identifiable within the reference setup, this does not guarantee full statistical flux identifiability for other design constellations.

#### A note on model dimensionality

Despite D- and A-information criteria (11) and (12), respectively, correct for the effect of differing dimensionality of the covariance matrix, we found in initial test runs that models with a lower number of free fluxes tended to be favored over models with full dimension *p* = 21. Since we are typically interested in determining as many fluxes as possible, the dimensionality criterion *Φ*_*DoF*_ has been added as obligate optimality criteria to the objective vector, which effectively diminished the trend to freeze free fluxes in favor of better D-, A-, or E-criteria values. Nevertheless, models with lower dimensionality still contributed to the Pareto-optimal solutions. Owing to the iterative constraining procedure to assure statistical flux identifiability, these lower dimensional models potentially differ in terms of free flux sets. However, to not over-complicate result interpretation, results with the full reference model dimensionality, *p* = 21, were filtered and only these are discussed in the following. For clarity, the models’ dimensionality is explicitly indicated by subscripts.

### Profiling analytical platforms: Information and cost trade-offs

With the ^13^C MFA *P*. *chrysogenum* model of at hand, two scenarios differing in the composition of the design objectives were studied. The goal of this first scenario was to profile the analytical platforms according to their information-cost trade-offs and to explore the underlying Pareto-optimal designs. To this end, three objectives were considered, two information criteria and the cost criterion:

- maximize the degrees of freedom *p*
- maximize flux confidence, as measured by the D-criterion
- minimize the costs of the CLE

Thus, the objective vector is represented by:

$$\Phi ={\left(\begin{array}{ccc} \Phi_{DoF} & \Phi_{D,p} & -\Phi_{Costs}^{dev} \end{array}\right)}^{T}$$ (19)

Due to the number of objectives involved, the MO-ED problem (1) with (19) is hitherto denoted *3D-MO-ED task*. Pareto-optimal solutions were calculated and objective values were recorded along with the identifiers of the statistically (non-)identifiable fluxes as well as the number of replicates for each single measurement group. Solutions obtained with models of maximal dimension, *p* = 21, are discussed in the following (the complete sets of Pareto sets and fronts are provided in S1 Data).

#### Comparing Pareto-optimal solutions: Potentials and risks

**Fig 6:**
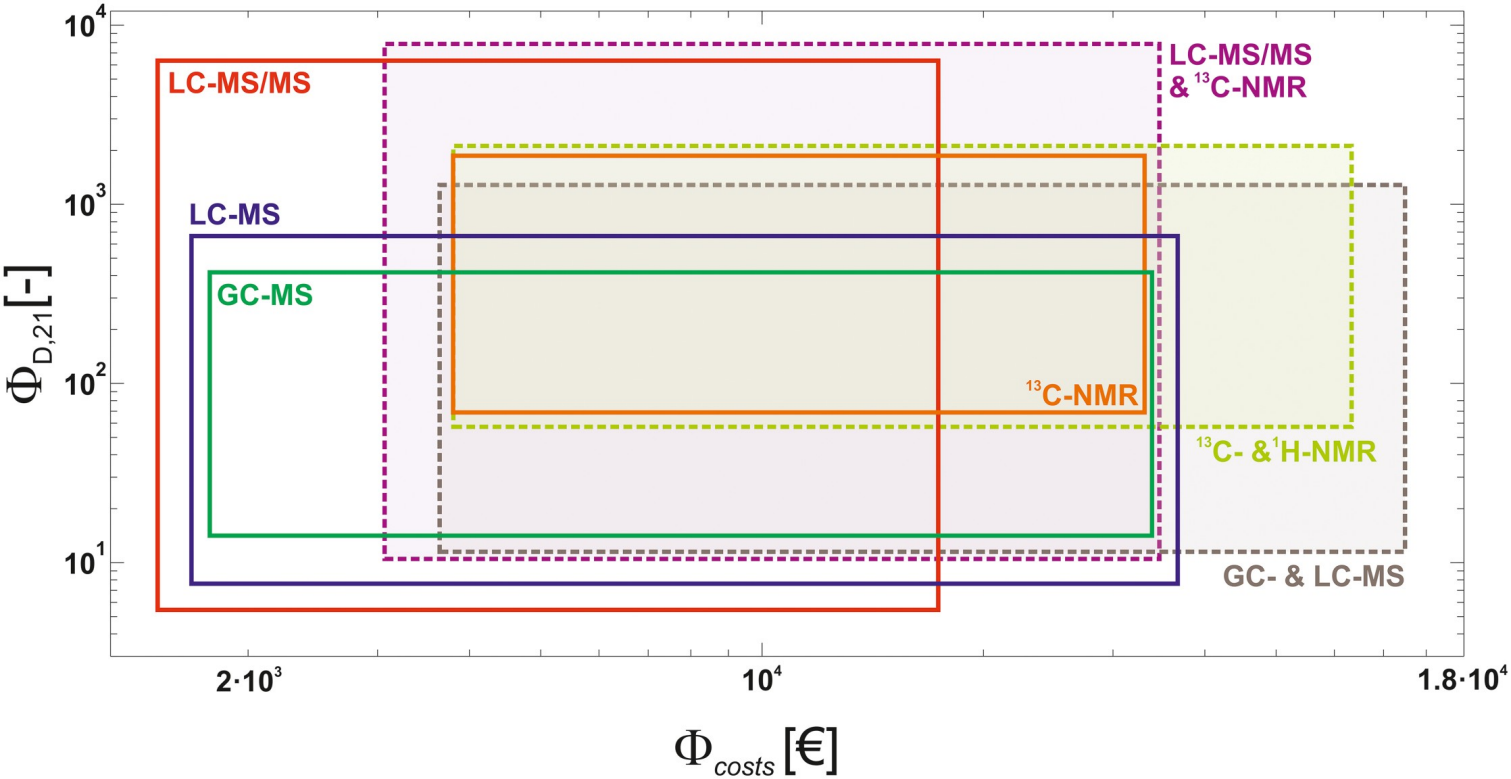
Ranges of Pareto-optimal D-criterion values *Φ*_*D*,*21*_ versus costs *Φ*_*Costs*_ of the 3D-MO-ED problem for different analytical platforms and platform combinations filtered for solutions with full model dimensionality (*p* = 21). Pareto frontiers connect lower left and upper right corners of the boxes (see also the Pareto fronts in Fig 7). Axes are log scaled. See supplementary information for results for *p* < 21 (S4 Text).

In order to access the principal possibilities of the analytical platforms with respect to the objective values, first their spread was compared as indicator of the variance of the Pareto designs (Fig 6).

With regard to maximal information, as measured by the D-criterion, LC-MS/MS ($\Phi_{D,21,max}^{LC-MSMS}=6,333$) was followed by ^13^C-NMR (ΦD,21,maxC13−NMR=1,867), LC-MS ($\Phi_{D,21,max}^{LC-MS}=665$), and GC-MS ($\Phi_{D,21,max}^{GC-MS}=417$). On the other hand, the minimal D-criterion value was found for a LC-MS/MS design followed by LC- and GC-MS. Interestingly, the spread was smallest for ^13^C-NMR and largest for LC-MS/MS, implying that 3D-MO-EDs for LC-MS/MS need to be designed carefully to get the most of a CLE. In contrast, the information content by ^13^C-NMR was fairly high, even in an (information) worst case scenario. Concerning the costs, designs expenses varied over at least one order of magnitude for all platforms ($1.5\leq \Phi_{Costs,21}^{LC-MSMS}\leq 17.4$, $1.7\leq \Phi_{Costs,21}^{LC-MS}\leq 36.7$, $1.8\leq \Phi_{Costs,21}^{GC-MS}\leq 33.9$, 3.8≤ΦCosts,21C13−NMR≤33.1 k€), thus, the overall cheapest designs were possible with LC-MS/MS.

#### How superior are cross-platform applications?

Intuitively, using more than one analytical device could boost both, information and cost measures. To investigate this, the 3D-MO-ED study was repeated for selected platform combinations, in particular:

- *GC-MS + LC-MS* being the nowadays most frequently used techniques;
- ^*1*^*H-NMR +* ^*13*^*C-NMR* providing absolute positional labeling enrichments and labeling information about neighboring labeling patterns where the analysis can be performed on the same device (with slight modifications);
- ^*13*^*C-NMR + LC-MS/MS* delivering complementary, highly-resolved labeling information.

Increases in costs for the multi-platform applications remained in ranges that could be expected. With respect to information, a combination of GC-MS and LC-MS increased the D-criterion value considerably ($\Phi_{D,21,max}^{GC/LC-MS}=1,282$, Fig 6). This value was exceeded by a factor of 1.65 by the combination of the two NMR techniques (ΦD,21,maxH1/C13−NMR=2,119). Finally, the information obtained with a combination of LC-MS/MS and ^13^C-NMR amounted to the overall highest information value (ΦD,21,maxLC−MSMS/C13−NMR=7,858), a plus of 24% compared to LC-MS/MS alone which was, however, bought by an up to 100% increase in costs.

Integrating GC-MS and LC-MS data showed the highest increase in terms of overall statistical flux information gain. We explain this by the orthogonal information supplied by the two platforms: Roughly, fragmented amino acids on the one hand and non-fragmented central carbon intermediates on the other. All in all, in this case study, LC-MS/MS together with ^13^C-NMR outperformed all other scenarios studied. Still, LC-MS/MS alone delivers a five-fold higher information value than a combination of GC- and LC-MS.

#### Exploring Pareto-optimal designs

**Fig 7:**
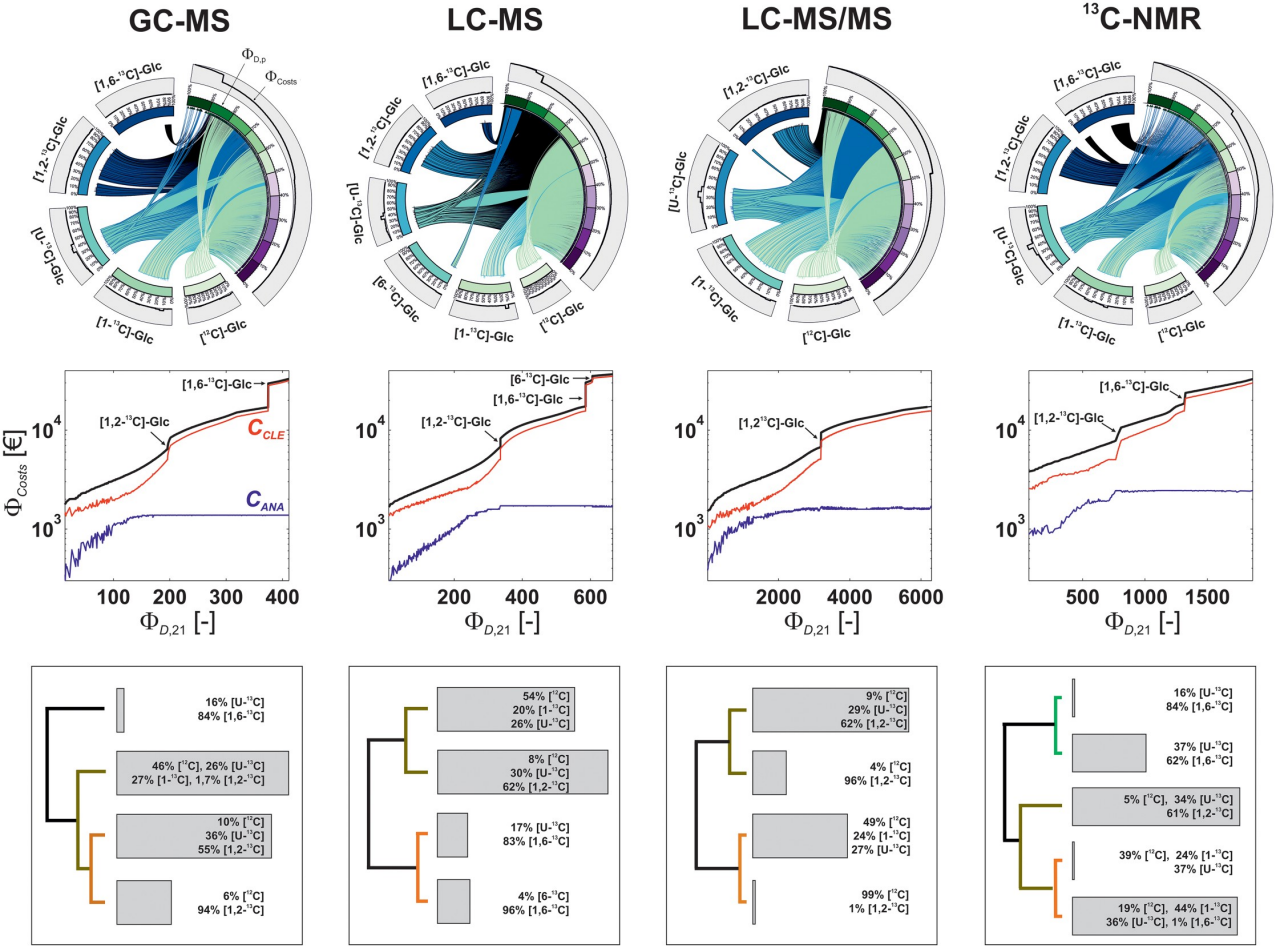
3D-MO-ED results for GC-MS, LC-MS, LC-MS/MS, and ^13^C-NMR filtered for models with maximal degree of freedom (*p* = 21). Top: Chord diagrams relate tracer compositions with D-criterion values and costs by showing an overlay of all Pareto-optimal tracer designs. Substrate species that contributed less than 1% to a mixture are omitted for clarity. Mid: Scatter plots showing Pareto fronts. Overall costs (black) are itemized into experimental (red) and analytical (blue) parts. Step increases of the fronts in costs are attributed to the addition of isotopically labeled substrate species as indicated. In regions of low D-criterion values, the wriggled characteristics of the graphs originates from frequent switching between input substrates and measurement replicates. Cost axes are log-scaled. Bottom: Substrate clusters. D-criterion, costs and input species hierarchically clustered by minimal Euclidean distance. For the compositions average values are given and values below 1% are omitted for clarity. Gray bars scale with the frequency of the clusters. The length of the edges (distance) represents the dissimilarities of the mixtures. Enlarged versions of the chord diagrams are provided in S4 Text.

Aiming at an in-depth platform comparison, the Pareto-optimal CLE designs were examined further. In Fig 7, input mixture designs are related with the objective values utilizing CDs. Furthermore, scatter plots and dendrograms show the Pareto fronts and emerging substrate clusters, respectively.

Apart from low-information CLE designs, Pareto fronts of all platforms are well-populated and show clear profiles with only very few discontinuities (the jiggling fronts for low-information CLEs point to a broad variety of CLE settings contributing to these designs). These step increases were attributed to the addition of costly substrates, mostly [1,2-^13^C]- and [1,6-^13^C]-glucose. Apart from these rare cases, the scatter plots convey that slight changes in the mixture do not have a harsh effect on the D-criterion value, as indicated by the stable course of the curves. On the other hand, the CDs reveal how specific input species are linked with information and costs. For instance, cheap unlabeled glucose contributes to less informative mixtures in case of LC-MS and ^13^C-NMR while it contributes to highly informative mixtures for LC-MS/MS. Thus, CDs deliver characteristic, platform-specific footprints of the Pareto optima. Beyond the labeled species, dendrograms show commonalities and differences in substrate mixture clusters, e.g., a ~20/80 mixture of [U-^13^C]- and [1,6-^13^C]-glucose was suggested for GC-MS, LC-MS, and ^13^C-NMR but not for LC-MS/MS and at least one cluster with high [1,2-^13^C]-glucose content was found across all platforms.

While LC-MS/MS performs best with respect to the D-criterion value, taking the economic perspective, it also shows the least costs of all platforms studied. This seems puzzling since the high number of manual peak evaluations could be expected to increase the overall costs significantly. However, Fig 7 reveals that the effective cost advantage of LC-MS/MS originates from the fact that, except for [1,2-^13^C]-glucose, exclusively comparably cheap substrates were selected. For all other platforms, [1,6-^13^C]-glucose participates in the most informative substrate mixtures which, in turn, leads to a considerable increase in overall costs. Not surprising, ^13^C-NMR shows the highest costs among all analytical devices, due to purchasing costs as well as long analytical acquisition times which are both up to ten times higher as compared to the other devices. Generally, the predominant part of the costs is constituted by the substrate prices (*C*_*CLE*_), while the analytical costs ($C_{ANA}^{dev}$) contribute with an offset.

Regarding the mixture design, six out of the ten available substrate species contribute to Pareto-optimal sets, namely [^12^C]-, [1-^13^C]-, [6-^13^C]-, [1,2-^13^C]-, [1,6-^13^C]-, and [U-^13^C]-glucose, while high-cost singly labeled [2-^13^C]-, [3-^13^C]-, [4-^13^C]-, [5-^13^C]-glucoses were not selected. Interestingly, expensive [1,2-^13^C]-glucose and [1,6-^13^C]-glucose species contribute to information-high mixtures for GC-MS, LC-MS, and ^13^C-NMR. Remarkably, these substrates can increase the statistical identifiability by ~50%. In the LC-MS setting, also [6-^13^C]-glucose raises the D-criterion value. On the other hand, cheap unlabeled glucose contributes primarily to mixtures with lower information criterion values while uniformly labeled glucose is used in a wide variety of compositions whose information values are spread over the complete range. Overall, for the different platforms a medium number of clusters was determined, among which also commonly used mixtures like [^12^C]-, [1-^13^C]-, and [U-^13^C]-glucose occurred.

To examine whether principally non-informative measurement groups exist, Pareto-optimal measurement groups and replicate numbers were extracted for each substrate mixture cluster (detailed results are provided in S4 Text and S3 File). Overall, analysis of the results shows a clear tendency to use most measurements groups with a high number of analytical replicates. This is explained by minimal extra costs of an additional replicate, especially when compared to those of the substrates. In case of LS-MS/MS, only few measurements were less frequently selected, e.g., 2,3-phosphoglycerate, glyceraldehyde-3-phosphate, or malate, arguably because these measurement groups provided only minor extra information with respect to flux confidence. For ^13^C-NMR either integration of the measurement group at maximal replicate numbers or the omission of the complete group was proposed while in case of LC-MS replicate numbers were indeed selected gradually starting with five replicates upwards. Expectedly, cost-beneficial designs delivered more diverse replicate patterns than cost-intensive ones. In particular, this holds for the in each case cheapest mixture clusters for GC-MS, LC-MS, LC-MS/MS, and ^13^C-NMR (S4 Text and S2 File). In any of those cases the proposed measurements strongly varied with respect to measurement groups and replicate numbers. Contrary, visual analysis reveals furthermore that optimal substrate species are conserved for platform combinations. The tight interplay between Pareto-optimal tracers, measurements and flux information manifested in complex device-specific correlation patterns (S4 Text and S1 File).

### Multiple information criteria

The previous study revealed detailed insights into trade-off CLE designs for *P*. *chrysogenum* that relied on the commonly used D-criterion as quantitative information measure. With our second scenario we aimed to study the impact of including additional information criteria on the MO-EDs. To this end, the objective vector is extended by A- and E-information criteria:

$$\Phi ={\left(\begin{array}{ccccc} \Phi_{DoF} & \Phi_{D,p} & \Phi_{A,p} & \Phi_{E} & -\Phi_{Costs}^{dev} \end{array}\right)}^{T}$$ (20)

The MO-ED scenario (1) with (20), henceforth referred to as *5D-MO-ED*, was performed along the same lines as the 3D-MO-ED study. In the following, selected results are presented and related to the outcomes of the previous ED results. Detailed results are given in the S5 Text.

#### An investigative analysis of multi-criteria CLE designs

5D Pareto-optima obtained, again after filtering solutions for full-dimensional models (*p* = 21), unravel highly complex relationships between the different information objectives and the costs. This becomes quite obvious from the 5D-MO-ED CDs provided in S5 Text and S1 File. In particular, the density of edge crossings between the information criterion values (which cannot be disentangled by rearranging the order of the arcs) evidences the mutual incompatibility of the objectives, in particular for LC-based platforms. The CD footprints clearly demonstrate that, compared to the 3D study, 5D Pareto-optimal designs show a higher degree of variation in the selection of substrate species. In each case, at least eight of the ten available tracers contribute to Pareto-optimal designs. Interestingly, one of the most costly substrates, [1,6-^13^C]-glucose contributes to a variety of designs across all platforms. Also [^12^C]-, [1-^13^C]-, [6-^13^C]-, [U-^13^C]-, [1,2-^13^C]-glucoses participate in a broad range of mixtures, while costly positionally labeled [2-^13^C]-, [3-^13^C]-, [4-^13^C]-, [5-^13^C]-species remain under-represented.

**Fig 8:**
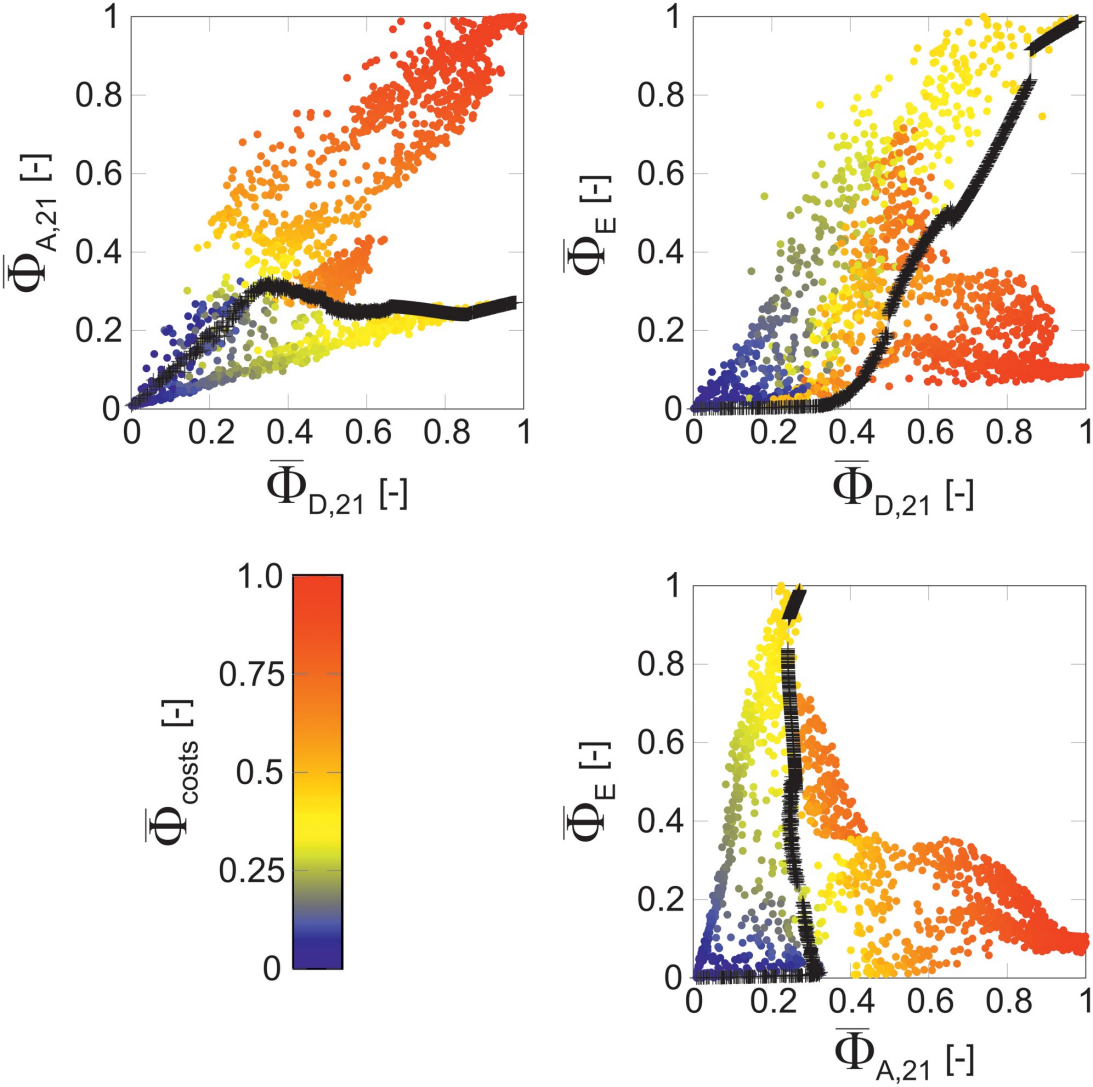
Pareto fronts obtained for LC-MS/MS filtered for solutions with full model dimensionality (*p* = 21). Colored scatter plots represent 2D projections of the Pareto front approximation for the 5D-MO-ED scenario, the 3D-MO-ED Pareto front is shown in black. Design costs are color-coded. For comparability, all criterion values are normalized to [0,1]. Complete Pareto fronts are provided in S1 Data.

For a closer inspection of the relations between the objectives, 5D-MO-ED Pareto fronts were projected to 2D scatter plots. To compare the results with those derived in the first scenario, 3D-MO-ED Pareto-optimal criterion values were incorporated into the plots. Results for LC-MS/MS are shown in Fig 8, while the corresponding outcomes for the other platforms are found in S5 Text, S1 and S2 Files.

In any case, the emerging point clouds exhibit complex, highly curved shapes. Connected regions in the objective space indicate a flexible choice of trade-off designs while concisely shaped correlations between two objectives reflect a lack of alternative designs, at least for solutions considering full model dimensionality.

First and as expected, 5D-MO-ED solutions for LC-MS/MS in Fig 8 show the same or lower Pareto-optimal D-criterion values than those of 3D-MO-EDs. Since 3D-MO-ED is a special case of 5D-MO-ED, this is regarded as cross-check that the MO framework produces reliable results. The lower D-criterion values in the 5D-MO-ED scenario are attributed to increases in other objectives, especially the A-criterion. Strikingly, 3D-MO-ED Pareto-optimal designs have only low A-objective values while 5D-MO-EDs with high D- and A-criterion values exist. On the other hand, 3D-MO-EDs with high D-criterion values have also high E-criterion values. While 5D-MO-ED designs with high D- and A- as well as D- and E-criterion values exist, this is not the case for E- and A-optimal designs, clearly demonstrating that A- and E-criteria are incommensurable. Further findings for LC-MS/MS are:

- Compared to 3D-MO-ED designs, top-priced 5D-MO-ED designs are more than twice as expensive.
- Highly A-informative designs mandate for costly CLEs whereas a wide variety of moderately costly D- and E-informative designs is proposed.

This explains that the preference of the 3D-MO-ED solution towards high E-criteria values occurred not by chance, but triggered by cost considerations.

Studying 5D-MO-ED solution scatter plots for the remaining analytical platforms reveals some quite different characteristics:

- *LC-MS*: designs with high D-/A-criterion values are possible, but not those with high D-/E- and A-/E-criterion values.
- *GC-MS*: joint D-/E- and A-/D-optimal designs exist, while designs with high A-/E-criterion values are not found.
- ^*13*^*C-NMR*: A-/D-/E-objectives are coherent, meaning that designs are available that deliver high values for all three criteria with only minor conflicts.
- Costs of 3D-MO-ED and 5D-MO-ED Pareto designs are comparably similar for LC-MS, GC-MS, and ^13^C-NMR.

Despite these differences, A-informative designs are the most expensive ones across all investigated platforms. To conclude, in both scenarios a wide variety of designs was observed, where 5D-MO-ED results provide a super-set of the 3D-MO-EDs as enabled by the consideration of two additional objectives. Thus, EDs with many objectives unlock multifaceted additional insights into alternative experimental settings, as demonstrated here for the ^13^C MFA case study with *P*. *chrysogenum*.

## Discussion

Intracellular fluxes are of special importance, as they describe the trafficking of metabolites which emerges as the final outcome of all catalytic and regulatory processes acting within living cells. Model-based ^13^C MFA is the gold standard for the quantification of intracellular metabolic fluxes. A smart combination of tracers and measured labeling patterns, i.e., the tracer composition, measurement groups, number of replicate measurements, are the key to accurate flux determination. Since ^13^C MFA studies remain complicated and costly, experimental design can safeguard against sub-optimal resource utilization. Using the concept of single-objective ED previous studies provided valuable indications for informative tracer mixtures. These studies have been performed for specific measurement setups and without considering economic aspects in experiment and analytics. To exploit the full power of ED in ^13^C MFA, here we generalized existing work by simultaneously taking several information quality measures as well as several experimental-analytical cost contributors into account. With a large-scale ^13^C MFA model and realistic measurement setups at hand, widely used analytical platforms were compared with respect to information-economic design options. With that, tracer and measurement design was performed simultaneously rather than independently. The MO-ED technique for designing informative, yet economic experiments, as showcased with the *P*. *chrysogenum* application study, is transferable to virtually any model-based approach and experimental-analytical setup, e.g., to plan parallel CLEs.

### Opportunities, challenges, and new insights

MO-ED enables the determination of design ensembles that seek to balance mutually exclusive information- and cost-objectives. Understanding the characteristics of the Pareto sets and the relationships between the different objectives is invaluable to guide the decision process on how to perform the next experiment. However, the sheer size of the design space and the many and various properties of the design parameters pose new challenges for the exploration procedure. First, searching for Pareto-optimal sets exhaustively over the whole, high-dimensional design space is compute-intensive and requires the efficient evaluation of the system model. Here, this challenge was tackled by connecting the high-performance simulator 13CFLUX2 with the optimization library jMetal. Second, a tailored visual analysis workflow was invented that tracks down Pareto-optimal designs thereby relating tracers, measurement groups, replicate numbers, costs, and information measures by means of graphical representations, starting from most relevant (input species) to less informative features (replicates). This workflow aids the scientist to weigh the insights against the costs and, thus, guides decision making.

### Deviations from the reference design point, a caveat?

ED studies were performed for a reference flux distribution representing prior information about the expected fluxes. It is, however, likely that the actual flux distribution under which a CLE is conducted differs from the assumed one. Because the information criteria used in this work rely on local statistical measures, actual Pareto-optimal designs may be widely different from the suggested ones. To investigate the robustness of the 3D-MO-ED Pareto designs in terms of information gain with respect to deviations from the reference flux values, for each platform 10,000 flux distributions were randomly sampled in the bounding box of the corresponding confidence ellipsoids. For the in each case most informative 3D-MO design setting, the D-information criteria values were calculated (for instance in case of LC-MS/MS for pure [1,2-^13^C]-glucose). In all cases, the average information value of Pareto-optimal results remained in the upper third suggesting that the determined MO-ED designs are reasonably robust (S4 Text and S3 File).

### Pareto-optimal input substrates

The set of Pareto-optimal labeled tracers for CLEs was found to be remarkably similar across all investigated platforms and platform combinations, e.g., [3-^13^C]-, [4-^13^C]-, and [5-^13^C]-glucose rarely contribute to the designs. However, the quantitative composition of the Pareto-optimal tracers varies widely. Often used, inexpensive substrate mixtures consisting of [1-^13^C]-, [U-^13^C]-, and [^12^C]-glucoses provide moderate statistical identifiability for LC-MS/MS. Several former single-objective ED studies found [1,2-^13^C]-glucose to be particularly informative (cf. Sec ED approaches in ^13^C MFA revisited). Although this tracer is more expensive than standard mixtures, our results show that [1,2-^13^C]-glucose is beneficial to achieve a higher degree of flux confidence across all studied platforms. Our study also reveals that the use of other, more expensive substrate species such as [1,6-^13^C]-glucose, which seldom have been suggested by conventional ED studies before, is mandatory when a high degree of flux confidence is needed (as measured by the D-criterion), especially for GC-MS, LC-MS, ^13^C-NMR, GC-MS/LC-MS, ^1^H-NMR/^13^C-NMR. These findings yield a generalized view on existing work that focuses on single objective ED aspects.

### Cost breakdowns

The study delivers detailed experimental and analytical cost reports for all analytical platforms. 3D-MO-ED results demonstrate, not surprisingly, that the substrate species of choice are the main contributor of the costs. On the other hand, often almost the complete available measurement spectrum, including the maximal number of replicates, contributed to the Pareto-optimal designs arguably because, compared to the substrates, additional measurements come almost for free while they always increase the statistical information gain of the CLE. Only for inexpensive substrate mixtures some measurement groups did not contribute to the designs, most likely due to their redundancy. Hence, savings in analytical costs are possible but only achievable to a lesser extent. Interestingly, robust A-optimal designs emerge to be the most expensive ones across all investigated analytical platforms.

### The benefit of considering multiple information criteria

Traditional 1D ^13^C MFA experimental planning techniques as first proposed by Möllney et al. [25] capture the “value” of a CLE in a single scalar measure of information content which is of limited value. By generalizing the 1D formulation to nD, strikingly, our study demonstrated that the latter gives a much more comprehensive view on Pareto-optimal designs, therewith opening up new possibilities for the experimenter in the planning phase of an experiment. The competition between single criteria is reflected in diverse, partly orthogonal designs. For instance, A-/D- and E-/D-optimal designs but no A/E-optimal designs co-exist for LC-MS/MS. The ability to account for a range of information criteria allows to pro-actively countering undesired side effects caused by (a priori unknown) flux correlations and, thus, could increase the design’s reliability. Importantly, these results were found to be specific to the analytical platform under consideration. Clearly, this wealth of additional insights comes at a computational cost. Here, the generalized ED framework has taken advantage of recent algorithmic advances in ^13^C MFA [23,51], which paved the way for complex field studies such as reported in this work.

### Analytical platforms for ^13^C MFA

Statistical flux identifiability with a comprehensive metabolic network of *P*. *chrysogenum* varies strongly among the measurement techniques. Even acknowledging long analysis times and high equipment costs, LC-MS/MS provides EDs with 50% less costs than other devices due to the use of cheaper input substrates. Simultaneously, LC-MS/MS yields up to ~300% higher information values as compared to the other techniques. Remarkably, the first scenario showed that the spread of Pareto-optimal designs has the highest coverage for LC-MS/MS, thus offering more options to the investigator than GC-MS, LC-MS, and ^13^C-NMR.

### ^13^C MFA design *à la carte*

Eventually, the goal of ^13^C MFA is to measure metabolic fluxes with the highest possible precision. Hence, the question arises whether the extra effort of MO-ED pays off in practice. A use case scenario may be as follows: An ED is desired with overall equally well determined fluxes and as little flux correlations as possible. Analyzing the 5D-MO-ED results with a high E-criterion value, corresponding designs may yield large flux confidence regions. In contrast, A-optimal designs indeed deliver superior designs in the sense of overall flux precision. However, by inspecting the costs associated with A-optimal designs, it becomes apparent that CLEs with A-criterion values overrun the budget. In this situation, alternate A-optimal designs satisfying certain cost constraints can be readily identified and even further ranked by their E- and/or D-criteria values. Having localized the desired Pareto-set(s), the associated designs can be further explored in depth providing detailed specifications of substrate composition and the measurement setup. Operated in that way, we believe MO-ED to become a useful new tool for prospective and rational planning of experiments under full cost control.

Besides deploying the framework to further application fields, there are several options to follow up this work. Technically, dependencies of the MO-EDs on the local design points should be diminished, e.g., by incorporation of global sensitivity analysis [60] or other more advanced design techniques [61] into the framework, to handle scenarios when pre-knowledge on the model parameters is absent. Practically, introducing interactive features to the visual analysis such as browsing, querying, filtering, or sorting could boost the quick understanding relationships within and in-between Pareto sets.

## Supporting information

S1 Text Measurement models. Details on the derivation of measurement error models for the analytical platforms GC-MS, LC-MS, LC-MS/MS, ^13^C-NMR, ^1^H-NMR, and GC-C-IRMS.
(PDF)

S2 Text Multi-objective ^13^C MFA: Formulation and setup. Mathematical formulation of the multi-objective optimization task for ^13^C MFA, details about multi-objective optimizers, documentation of the cost factors and the experimental scenario.
(PDF)

S3 Text ^13^C MFA network model of *P*. *chrysogenum*. Description of the network model for *P*. *chrysogenum* including carbon atom transitions, constraints, and flux distribution.
(PDF)

S4 Text Multi-objective experimental design: Results for the 3D scenario. Documentation of results for the analytical platforms GC-MS, LC-MS, LC-MS/MS, ^13^C-NMR and platform combinations.
(PDF)

S5 Text Multi-objective experimental design: Results for the 5D scenario. Documentation of results for the analytical platforms GC-MS, LC-MS, LC-MS/MS, ^13^C-NMR.
(PDF)

S1 Data Pareto set archives. ZIP archive containing the Pareto archives; from the Pareto archives all results discussed in the paper are derived.
(ZIP)

S1 File Pareto scatterplots—Matlab scripts and figures. ZIP archive containing Pareto scatterplots for 3D- and 5D-MO-ED scenarios and scripts to reproduce Figs 7 and 8.
(ZIP)

S2 File Substrate cluster and replicate evaluation. ZIP archive containing Matlab scripts to produce the substrate clusters and replicate evaluations (S4 and S5 Text).
(ZIP)

S3 File Sensitivity evaluation. ZIP archive containing information criteria for flux space samples and Matlab scripts to evaluate variations.
(ZIP)
