# Supplementary material for: A Pareto approach to resolve the conflict between information gain and experimental costs: Multiple-criteria design of carbon labeling experiments
Source: PLoS Comput Biol. 2018 Oct 31;14(10):e1006533. doi: 10.1371/journal.pcbi.1006533 (PMC6209137; doi:10.1371/journal.pcbi.1006533)
Supplement: S1 Text — Details on the derivation of measurement error models for the analytical platforms GC-MS, LC-MS, LC-MS/MS, 13C-NMR, 1H-NMR, and GC-C-IRMS. (PDF) [file pcbi.1006533.s001.pdf]

Supplementary Appendix 1 for

A Pareto approach to resolve the conflict between information gain and experimental costs: Multiple-criteria design of carbon labeling experiments

Measurement Models

Katharina Nöh, Sebastian Niedenführ, Martin Beyß, Wolfgang Wiechert

k.noeh@fz-juelich.de

Contents

1. Analytical platforms – overview ... 3
2. Compilation of calibrated device-specific measurement error models ... 5
3. Gas Chromatography-Mass Spectrometry (GC-MS) ... 7
   3.1 Measurement specification ... 7
   3.2 Measurement model ... 11
   3.3 Measurement error model ... 11
4. Liquid Chromatography-Mass Spectrometry (LC-MS) ... 12
   4.1 Measurement specification ... 12
   4.2 Measurement model ... 15
   4.3 Measurement error model ... 15
5. Liquid Chromatography-Tandem Mass Spectrometry (LC-MS/MS) ... 16
   5.1 Measurement specification ... 16
   5.2 Measurement model ... 20
   5.3 Measurement error model ... 20

6. ^{13}C-Nuclear Magnetic Resonance Spectrometry (^{13}C-NMR) 21
6.1 Measurement specification 21
6.2 Measurement model 25
6.3 Measurement error model 25
7. ^{1}H-Nuclear Magnetic Resonance Spectrometry (^{1}H-NMR) 26
7.1 Measurement specification 26
7.2 Measurement model 28
7.3 Measurement error model 28
8. Gas Chromatography-Combustion-Isotope Ratio Mass Spectrometry (GC-C-IRMS) 29
8.1 Measurement specification 29
8.2 Measurement model 31
8.3 Measurement error model 31
9. References 32

1. Analytical platforms – overview

This section briefly introduces relevant terminology used throughout, while the interested reader is referred to extensive expert reviews and textbooks for more details [1,2].

NMR is a noninvasive technology that distinguishes isotopes by their magnetic properties. The separation relies on frequency shifts induced by protons (^{1}H-NMR), carbon nuclei (^{13}C-NMR) separately or in combination (heteronuclear NMR). ^{1}H-NMR measures the positional fractional enrichment in a single carbon position and therewith provides specific relative label information [3]. ^{13}C-NMR quantifies local patterns of neighboring labeled carbon atoms [4,5]. NMR based technologies deliver measurement information with high chemical specificity, but devices are high-priced and suffer from low sensitivity implying the need for large sample sizes and comparably long acquisition times.

Complementary to NMR, (tandem) MS instruments measure sums of isotopomers or isotopomer fragments sharing identical masses ("cumulative enrichments") [6]. In particular, in the collision-induced fragmentation step within tandem MS procedures, molecules (precursor ions) are cleaved into smaller parts (product ions) which can deliver positional labeling information [7–9]. Typically, MS(/MS) is coupled with a preceding chromatographic separation step, such as gas and liquid chromatography (GC/LC) to increase measurement selectivity. In the field of ^{13}C MFA, GC-MS is the predominant platform applied. GC-combustion-isotope ratio mass spectrometry (GC-C-IRMS) is a highly specialized technique to quantify the total fraction of labeled and unlabeled carbon content per molecule [10–12], with detection levels two orders of magnitude lower than GC-MS while requiring only very small sample sizes [13]. Besides these hyphenated techniques, also "direct" matrix-assisted laser desorption/ionization time-of-flight (MALDI-TOF) MS and Fourier transform-ion cyclotron resonance mass spectrometry (FT-ICR-MS) have been applied in ^{13}C MFA, although use cases are rare [14,15].

NMR and MS based analytical platforms differ in their ability to resolve certain chemical classes of metabolites, detectable analyte concentrations and achievable fragmentation patterns (S1 Table A).

Table A. Analytical platforms typically used in ^{13}C MFA studies along with the reported target analyte classes.

	**Analyte spectrum**			
	**Organic acids**	**Sugars**	**Amino acids**	**Comment**
^{1}H-NMR	-	-	[3]	Fine structures; mostly from hydrolyzed cellular proteins due to limited sensitivity (> 1-2 nmol); non-destructive technology; costly instruments; long acquisition times and complex analysis
^{13}C-NMR	-	-	[4,16–20]	Fine structures; mostly from hydrolyzed cellular proteins due to low sensitivity; nucleosides; non-destructive technology; costly instruments; long acquisition times and complex analysis

GC-MS	[21,22]	[23]	[17,23–31]	Mass isotopmomers; derivatization and bias correction step for natural abundant isotopes mandatory; indirect fragment labeling information; very robust and reproducible; easy analysis; most abundant technique for ^{13}C MFA; good analytical cost-benefit ratio
GC-C-IRMS	-	-	[10,32]	Fractional enrichment; especially suited for low labeling content; extensive isolation; small metabolite spectrum
LC-MS	[33–35]	[34,35]	[7,34,36]	Mass isotopomers; non-trivial data analysis; measures against ion suppression required; high sensitivity and selectivity; direct labeling information
LC-MS/MS	[7,37]	[34,35,37]	[7,37]	Tandem mass isotopomers; non-trivial and time-consuming data analysis; measures against ion suppression required; highest sensitivity and selectivity; direct fragment labeling information
MALDI-TOF-MS	-	[14]	[14]	Crude extracts; direct introduction method; more tolerant to higher salt content samples than electrospray ionization methods; new MALDI matrices minimize the issue of matrix inference; fast analysis
FT-ICR-MS	-	[38]	[15,39]	Ultra-high resolution and mass accuracies better than 0.2 ppm; fast analysis < 5 min per sample

With respect to their use in the context of ^{13}C MFA, comparative investigations on the inter-platform information content of CLEs for ^{13}C MFA are scarce. Jeffrey et al. compared ^{13}C-NMR, GC-MS, and GC-MS/MS for measuring the ^{13}C-fractional enrichment of glutamate resulting in the statement that flux results benefit from the increased number of MS/MS measurement as compared to those obtained by ^{13}C-NMR and GC-MS [40]. Single and tandem LC- and GC-MS, respectively, have been applied in a network-wide manner in [7,41] and it was shown that tandem MS indeed gives a better overall flux determinacy compared to single MS. On the other hand, different analytical techniques have been combined to increase the coverage of the metabolite and isotopomer spectrum. For instance, GC-MS, ^{1}H- and ^{13}C-NMR by McKinlay et al. for the capnophilic bacterium *Actinobacillus succinogenes* [42], GC-MS, LC-MS and ^{13}C-NMR for ^{13}C MFA in *Saccharomyces cerevisiae* [35], and LC-MS with ^{13}C-NMR in *Penicillium chrysogenum* [43]. Recently, GC- and LC-MS derived labeling data were jointly used to resolve metabolic fluxes in *Pseudomonas fluorescens* [44].

2. Compilation of calibrated device-specific measurement error models

To arrive at realistic error approximations for the measurement covariance matrix, data from studies featuring different organisms, platforms and various labeling contents are collected [3,10,19,20,23,25,27,34,36,37,42,45–49]. For six analytical platforms, namely GC-MS, LC-MS, LC-MS/MS, ^{13}C-NMR, ^{1}H-NMR, and GC-C-IRMS, published measurements and their corresponding standard deviations were extracted (cf. Sec. 3-8). The measurements are assumed to be corrected for natural abundance. Each measurement group was considered only once, also if it is acquired by different analytical methods. In order to prevent over-optimistic predictions, we used conservative, i.e., on average higher error estimates than reported in, e.g., [15,45]. For the same reason, very specialized setups are not considered in the survey (e.g.[38]).

Not unexpectedly, the reported data showed large variances. Similar to the approach in Dauner et al. for ^{13}C-NMR [18], we proposed a linear regression between the measurements' standard deviation and the observed signal:

$$\boldsymbol{\sigma}_{meas}^{dev,lit} = b_1^{dev} \cdot \boldsymbol{\eta} + b_2^{dev}$$

where the device-specific regression coefficients b_1^{dev}, b_2^{dev} are calibrated with all data collected for the device, i.e., across all measurement groups. For ^{1}H-NMR and GC-C-IRMS only few labeling data sets are publicly available, all with only low label incorporation levels questioning the validity of the error models for these two platforms for higher levels of fractional enrichments.

Results of the linear regressions are shown in S1 Fig A, including the values for the coefficient pairs. In summary, the regression lines of the different platforms show only minor differences in their slopes. Roughly, in regions with low label enrichment GC-C-IRMS and LC-MS/MS errors are modeled to be most accurate while for ^{13}C-NMR the error remains nearly constant over the whole possible labeling range. With these measurement error models at hand, errors become predictable in dependence of the analytes' labeling states. As a consequence, the variances in the main diagonal of the measurement covariance matrix depend on the labeling fraction and are, thus, heteroscedastic.

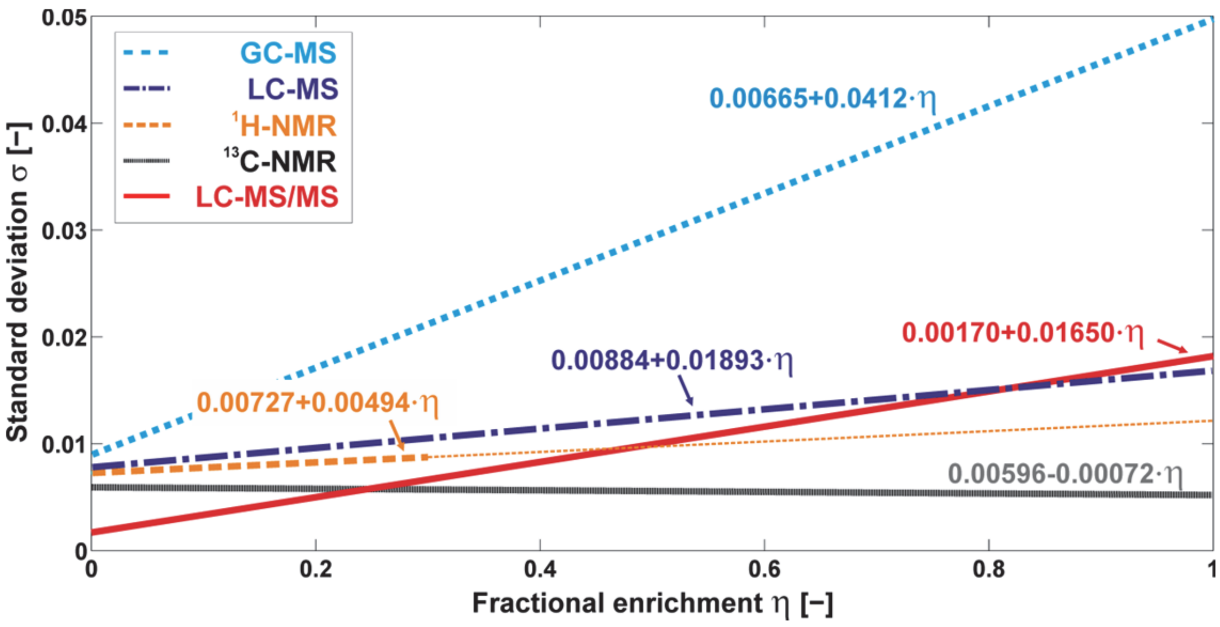

Fig A. Comparison of device-specific error models for fractional labeling measurements. Underlying data are collected from studies utilizing different organisms and labeling contents (see S1 Sec. 3-8 for the specifications). Standard deviations are determined by linear regression, i.e., by fitting a linear error model $\sigma = a \cdot \eta + b$ with slope (*a*) and y-axis intercept (*b*). Here, *b* represents the baseline error. For ^{1}H-NMR only data sets with a low abundance of ^{13}C labeling content are available. Therefore, the error model is considered realistic only for labeling fractions of less than 30% ^{13}C incorporation, while errors of measurements with higher labeling content are linearly extrapolated, as indicated by the change in line thickness.

3. Gas Chromatography-Mass Spectrometry (GC-MS)

3.1 Measurement specification

Table B. GC-MS measurement group specification.

Metabolite		# Carbons	Measurement specification	Fragment and/or mass	References (exemplary)
Alanine	ALA	3	ALA#M0,1,2,3	M-57, 260	[23], [45], [27], [25], [46], [42], [47]
			ALA[2-3]#M0,1,2	M-85, 232	[23], [45], [27], [25], [46], [42], [47]
				M-159	[25]
Arginine	ARG*	6	ARG#M0,1,2,3,4,5	442	[23]
Aspartate	ASP	4	ASP#M0,1,2,3,4	M-57, 418	[23], [45], [27], [46], [47]
				M-15, 460	[27]
			ASP[2-4]#M0,1,2,3	M-85, 390	[23], [45], [46], [47]
				M-159, 316	[23], [27]
			ASP[1-2]#M0,1,2	f302, 302	[45], [46], [47]
Aspartate/ Asparagine	ASX*	4	ASX#M0,1,2,3,4	M-57, 418	[25], [42]
			ASX[2-4]#M0,1,2,3	M-85, 390	[25], [42]
				M-159, 316	[25], [42]
			ASX[1-2]#M0,1,2	f302, 302	[25], [42]
Fumarate	FUM*	4	FUM#M0,1,2,3,4	M-57	[42]
Glycine	GLY	2	GLY#M0,1,2	M-57, 246	[23], [45], [27], [25], [46], [42], [47]
			GLY[2]#M0,1*	M-85, 218	[23], [45], [27], [25], [46], [42], [47]
Glutamate	GLU	5	GLU#M0,1,2,3,4,5	M-57, 432	[23], [45], [27] [46], [47]
			GLU[2-5]#M0,1,2,3,4	M-85, 404	[45], [27], [46], [47]
				M-159, 330	[23], [27], [46]
Glutamate/ Glutamine	GLX*	5	GLX#M0,1,2,3,4,5	M-57	[25], [42]
			GLX [2-5]#M0,1,2,3,4	M-85	[25], [42]
				M-159	[25], [42]
			GLX[1-2]#M0,1,2	f302, 302	[25]

Glutamine	GLN*	5	GLN#M0,1,2,3,4,5	M-57, 431	[46]
Histidine	HIS*	6	HIS[2-6]#M0,1,2,3,4,5	n.a.	[45]
Isoleucine	ILE	6	ILE#M0,1,2,3,4,5,6	n.a.	[45], [47]
			ILE[2-6]#M0,1,2,3,4,5	M-85, 274	[45], [27], [25], [46], [42], [47]
				M-159, 200	[27], [25], [46], [42]
Leucine	LEU	6	LEU#M0,1,2,3,4,5,6		[47]
			LEU[2-6]#M0,1,2,3,4,5	M-85, 274	[45], [27], [25], [46], [42], [47]
				M-159, 200	[27], [25], [42]
Lysine	LYS	6	LYS#M0,1,2,3,4,5,6	M-57	[25], [47]
			LYS[2-6]#M0,1,2,3,4,5	M-159, 329	[27], [25], [47]
			LYS[1-2]#M0,1	f302, 302	[25]
Methionine	MET	5	MET#M0,1,2,3,4,5	M-57, 320	[27], [25], [46], [42]
			MET[2-5]#M0,1,2,3,4	M-85, 292	[27], [25], [46], [42]
				M-159, 218	[27], [25], [46]
Phenylalanine	PHE	9	PHE#M0,1,2,3,4,5,6,7,8,9	M-57, 336	[23], [45], [27], [46], [42], [47]
			PHE[2-9] #M0,1,2,3,4,5,6,7,8	M-85, 308	[45], [27], [25], [46], [42], [47]
				M-159, 234	[23], [27], [25], [46]
			PHE[3-9] #M0,1,2,3,4,5,6,7	sc	[25]
			PHE[1-2]#M0,1,2	f302, 302	[23], [45], [25], [46], [42], [47]
Proline	PRO	5	PRO#M0,1,2,3,4,5	M-57	[45], [25], [47]
			PRO[2-5]#M0,1,2,3,4	M-85	[45], [25], [47]
				M-159, 184	[27], [25], [42]
Serine	SER	3	SER#M0,1,2,3	M-57, 390	[23], [45], [27], [25], [46], [42], [47]
			SER[2-3]#M0,1,2	M-85, 362	[23], [45], [27], [46], [42], [47]
				M-159,	[27], [25],

				288	[46], [42], [23]
			SER[1-2]#M0,1,2	f302, 302	[45], [25], [42], [47]
Succinate	SUC*	4	SUC#M0,1,2,3,4	M-15	[42]
				M-57	[42]
Threonine	THR	4	THR#M0,1,2,3,4	M-57, 404	[23], [45], [27], [46], [42], [47]
				M-57, 290	[27]
			THR[2-4]#M0,1,2,3	M-85, 376	[23], [45], [27], [46], [42], [47]
			THR[3-4]#M0,1,2*		[45]
Tyrosine	TYR	9	TYR#M0,1,2,3,4,5,6,7,8,9*		[47]
			TYR[2-9] #M0,1,2,3,4,5,6,7,8	M-85	[23], [25], [47]
				M-159	[25]
			TYR[1-2]#M0,1,2	f302, 302	[23], [45], [25], [46], [47]
Valine	VAL	5	VAL#M0,1,2,3,4,5	M-57, 288	[23], [45], [27], [25], [46], [42], [47]
			VAL[2-5]#M0,1,2,3,4	M-85, 260	[23], [45], [27], [25], [46], [47]
				M-159, 186	[23], [27], [42]
			VAL[1-2]#M0,1,2*	f302, 302	[45], [47]

*: metabolite not present in the reaction network or not used in the study
n.a.: information not available

The MS measurement specification METAB#M0,1,2,3 describes a MS measurement of the full molecule of the metabolite pool METAB having three carbon atoms. If only a certain molecule fragment is observed, the carbon-range is specified that contains the labeling positions, e.g., METAB[2-3]#M0,1,2.

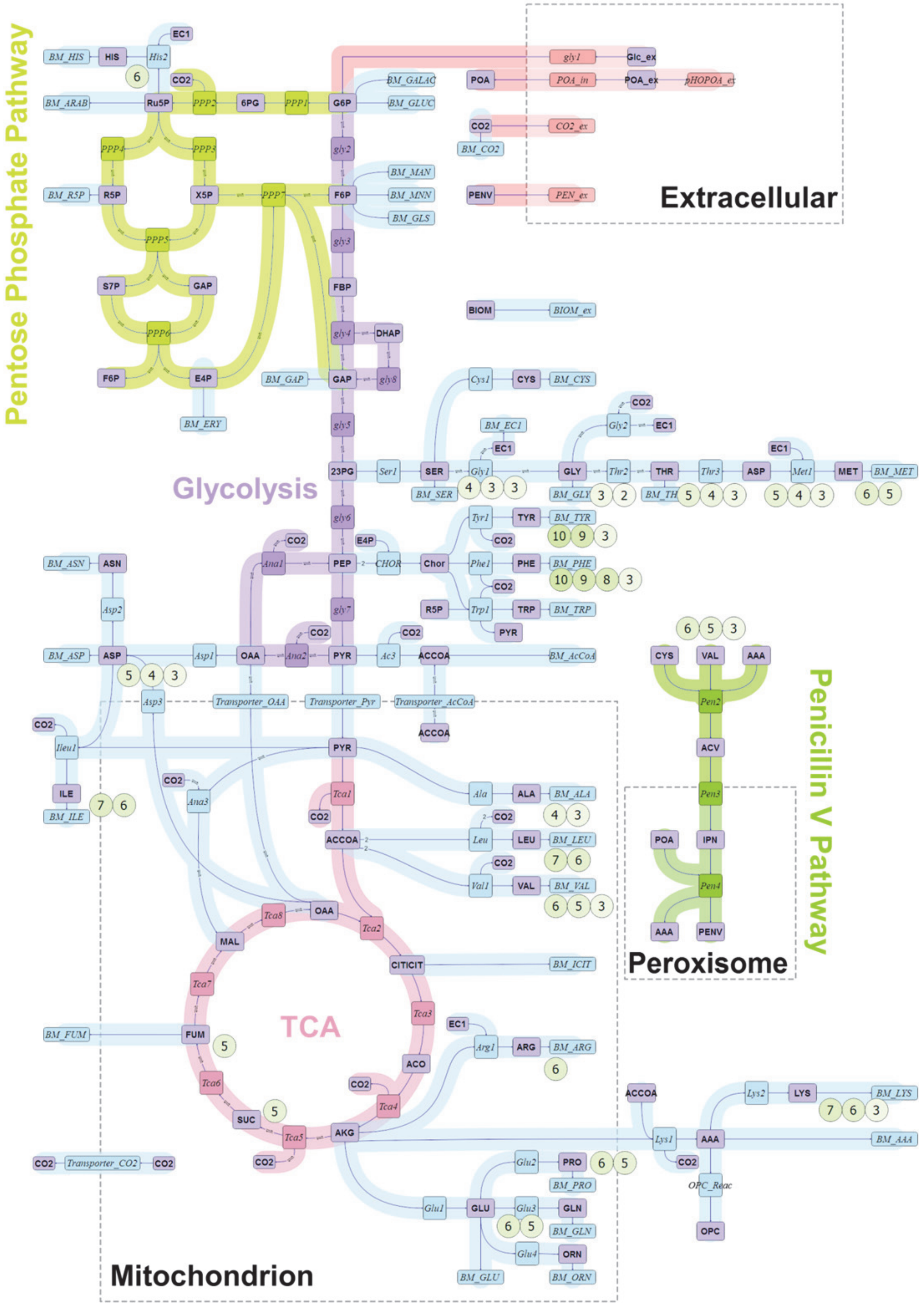

Fig B. Network and GC-MS labeling measurements. Each measurement group is represented by a circle giving the number of measured fractions. All metabolic network diagrams were drawn with the software OMIX [50]. Visualization of data was done with tailored OVL scripts.

3.2 Measurement model

Mass isotopomers can be expressed – up to a normalization factor – as a linear combination of isotopomer fractions [51], as shown here for the C3 metabolite pyruvate as an example:

$$\begin{pmatrix} PYR\#M0 \\ PYR\#M1 \\ PYR\#M2 \\ PYR\#M3 \end{pmatrix} = \begin{pmatrix} 1 & 0 & 0 & 0 & 0 & 0 & 0 & 0 \\ 0 & 1 & 1 & 0 & 1 & 0 & 0 & 0 \\ 0 & 0 & 0 & 1 & 0 & 1 & 1 & 0 \\ 0 & 0 & 0 & 0 & 0 & 0 & 0 & 1 \end{pmatrix} \cdot \begin{pmatrix} x_{\circ\circ\circ} \\ x_{\circ\circ\bullet} \\ x_{\circ\bullet\circ} \\ x_{\circ\bullet\bullet} \\ x_{\bullet\circ\circ} \\ x_{\bullet\circ\bullet} \\ x_{\bullet\bullet\circ} \\ x_{\bullet\bullet\bullet} \end{pmatrix} = \mathbf{M}_{PYR,MS} \cdot \mathbf{x}_{PYR}$$

where $\mathbf{x}_{PYR}$ denotes the vector of isotopomer fractions of the metabolite PYR.

3.3 Measurement error model

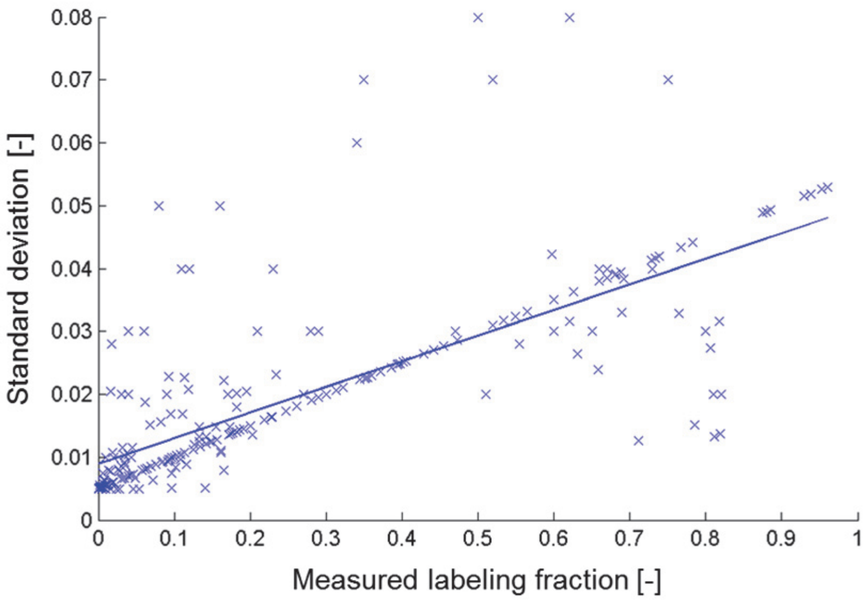

Fig C. Error model for GC-MS based labeling measurements compiled from published data sets (S1 Table B). Standard deviations were determined by linear regression, i.e., by fitting the linear error model $\sigma = b + m \cdot \eta$ with slope (m) and y-axis intercept (b) to the data resulting in $\sigma = 0.006653 + 0.04119 \cdot \eta$.

4. Liquid Chromatography-Mass Spectrometry (LC-MS)

4.1 Measurement specification

Table C: LC-MS measurement group specification.

Metabolite		# Carbons	Measurement specification	References (exemplary)
2-P-Glycerate + 3-P-Glycerate	23PG*	3	23PG#M0,1,2,3	[47], [49], [34]
6-Phosphogluconate	6PG*	6	6PG#M0,1,2,3,4,5,6	[47], [49], [34]
2-oxoglutarate	AKG	5	AKG# M0,1,2,3,4,5	[47]
Alanine	ALA	3	ALA#M0,1,2,3	[46], [36]
Arginine	ARG	6	ARG#M0,1,2,3,4,5,6	[52]
Asparagine	ASN	4	ASN#M0,1,2,3,4	[46], [36]
Aspartate	ASP	4	ASP#M0,1,2,3,4	[46]
Cysteine	CYS	3	CYS#M0,1,2,3	[37]
Dihydroxyacetone-phosphate	DHAP	3	DHAP#M0,1,2,3	[37]
Erythrose-4-phosphate	E4P	4	E4P#M0,1,2,3,4	[34]
Fructose-6-phosphate	F6P	6	F6P#M0,1,2,3,4,5,6	[47], [34]
Fructose-1,6-phosphate	FBP	6	FBP#M0,1,2,3,4,5,6	[47], [49], [34]
Fumarate	FUM	4	FUM# M0,1,2,3,4	[47]
Glucose-1-phosphate	G1P*	6	G1P#M0,1,2,3,4,5,6	[34]
Glucose-6-phosphate	G6P	6	G6P#M0,1,2,3,4,5,6	[47], [34]
Glutamine	GLN	5	GLN#M0,1,2,3,4,5	[36]
Glutamate	GLU	5	GLU#M0,1,2,3,4,5	[46], [36]
Glyceraldehyde-3-phosphate	GAP	3	GAP#M0,1,2,3	[37]
Glycine	GLY	2	GLY#M0,1,2	[46], [36]
Histidine	HIS	6	HIS#M0,1,2,3,4,5,6	[37]
Isoleucine	ILE	6	ILE#M0,1,2,3,4,5,6	[37]
Leucine	LEU	6	LEU#M0,1,2,3,4,5,6	[37]
Lysine	LYS	6	LYS#M0,1,2,3,4,5,6	[37]
Malate	MAL	4	MAL# M0,1,2,3,4	[47]
Methionine	MET	5	MET#M0,1,2,3,4,5	[46]
Oxaloacetate	OAA	4	OAA#M0,1,2,3,4	[37]
Ribose-5-phosphate + Ribulose-5-phosphate + Xylulose-5-phosphate	RU5P	5	RU5P#M0,1,2,3,4,5	[47], [49], [34]
Phosphoenol-pyruvate	PEP	3	PEP# M0,1,2,3	[47]
Phenylalanine	PHE	9	PHE# M0,1,2,3,4,5,6,7,8,9	[46], [36]
Proline	PRO	5	PRO#M0,1,2,3,4,5	[37]
Pyruvate	PYR	3	PYR# M0,1,2,3	[37]

Sedoheptulose-7-phosphate	S7P	7	S7P#M0,1,2,3,4,5,6,7	[47], [34]
Serine	SER	3	SER#M0,1,2,3	[46], [36]
Succinate	SUC	4	SUC# M0,1,2,3,4	[47]
Threonine	THR	4	THR# M0,1,2,3,4	[46], [36]
Tryptophane	TRP*	11	TRP# M0,1,2,3,4,5,6,7,8,9,10,11	[37]
Tyrosine	TYR	9	TYR# M0,1,2,3,4,5,6,7,8,9	[36]
Valine	VAL	5	VAL# M0,1,2,3,4,5	[46]

*: metabolite not present in the reaction network or not used in the study

The MS measurement specification METAB#M0,1,2,3 describes a MS measurement of the full molecule of the metabolite pool METAB having three carbon atoms.

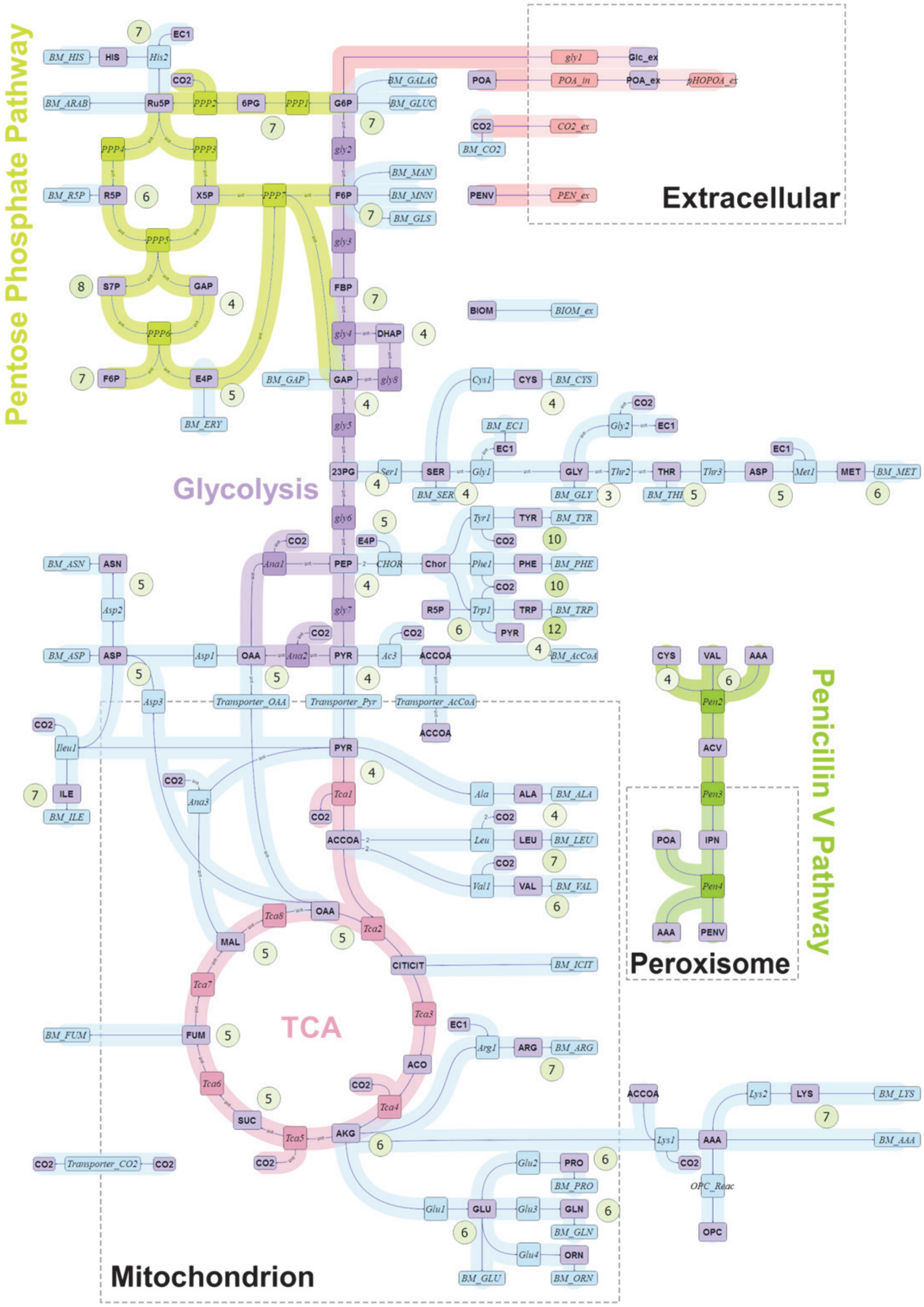

Fig D. Network and LC-MS labeling measurements. Each measurement group is represented by a circle giving the number of measured fractions.

4.2 Measurement model

same as in S1 Sec 3.2.

4.3 Measurement error model

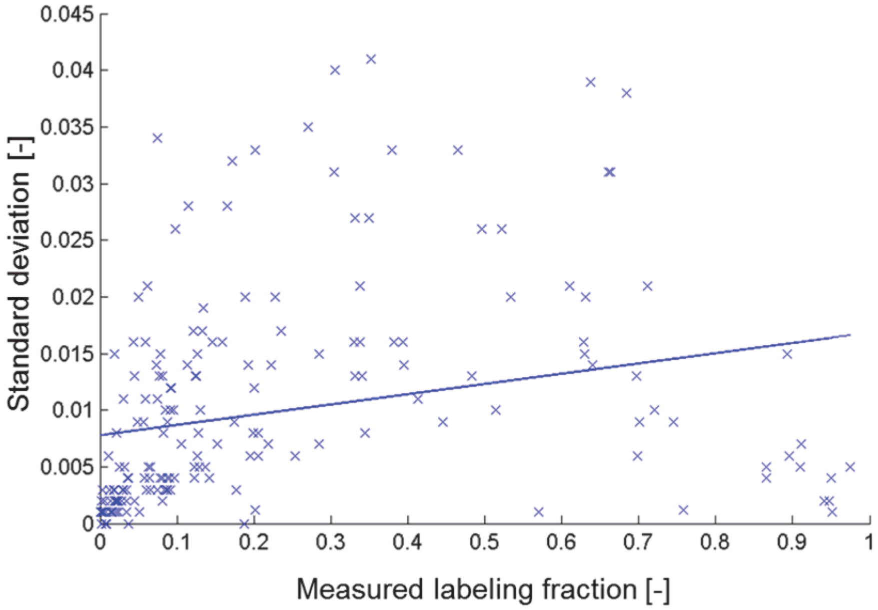

Fig E. Error model for LC-MS based labeling measurements compiled from published data sets (S1 Table C). Standard deviations were determined by linear regression, i.e., by fitting the linear error model $\sigma = b + m \cdot \eta$ with slope (m) and y-axis intercept (b) to the data resulting in $\sigma = 0.008839+0.018934 \cdot \eta$.

5. Liquid Chromatography-Tandem Mass Spectrometry (LC-MS/MS)

5.1 Measurement specification

Table D. LC-MS/MS measurement group specification.

**Metabolite**		**# Carbons**	**Measurement specification**	**Reference (exemplary)**
2-phosphate-glycerate + 3-phosphate-glycerate	23PG *	3	23PG[1-3:1-3]#M(0,0)(1,1)(2,2)(3,3)	[37]
6-phospho-gluconate	6PG*	6	6PG[1:6:1:6] #M(0,0)(1,1)(2,2)(3,3)(4,4)(5,5)(6,6)	[37]
2-oxoglutarate	AKG	5	AKG[1-5:1-4] #M(0,0)(1,0)(1,1)(2,1)(2,2)(3,2)(3,3)(4,3)(4,4)(5,4)	[37]
Alanine	ALA	3	ALA[1-3:2-3] #M(0,0)(1,0)(1,1)(2,1)(2,2)(3,2)	[37]
Arginine	ARG	6	ARG[1-6:1-5] #M(0,0)(1,0)(1,1)(2,1)(2,2)(3,2)(3,3)(4,3)(4,4)(5,4)(5,5)(6,5)	[37]
Asparagine	ASN	4	ASN[1-4:2-4] #M(0,0)(1,0)(1,1)(2,1)(2,2)(3,2)(3,3)(4,3)	[37]
Aspartate	ASP	4	ASP[1-4:1-2] #M(0,0)(1,0)(1,1)(2,0)(2,1)(2,2)(3,1)(3,2)(4,2)	[37]
Citrate + Isocitrate	CIT*	6	CIT[1-6:2-6] #M(0,0)(1,0)(1,1)(2,1)(2,2)(3,2)(3,3)(4,3)(4,4)(5,4) (5,5)(6,5)	[37]
Cysteine	CYS	3	CYS[1-3:2-3] #M(0,0)(1,0)(1,1)(2,1)(2,2)(3,2)	[37]
Dihydroxyacetone-phosphate	DHAP	3	DHAP[1-3:1-3]#M(0,0)(1,1)(2,2)(3,3)	[37]
Erythrose-4-phosphate	E4P	4	E4P[1-4:1-4]#M(0,0)(1,1)(2,2)(3,3)(4,4)	[37]
Fumarate	FUM	4	FUM[1-4:1-3] #M(0,0)(1,0)(1,1)(2,1)(2,2) (3,2)(3,3)(4,3) [1-3]=[2-4]	[37]
Fructose-6-phosphate	F6P	6	F6P[1-6:1-6] #M(0,0)(1,1)(2,2)(3,3)(4,4)(5,5)(6,6)	[37]
Fructose-1,6-phosphate	FBP	6	FBP[1-6:1-6] #M(0,0)(1,1)(2,2)(3,3)(4,4)(5,5)(6,6)	[37]
Glucose-6-phosphate	G6P	6	G6P[1-6:1-6] #M(0,0)(1,1)(2,2)(3,3)(4,4)(5,5)(6,6)	[37]
Glycer-aldehyde-3-phosphate	GAP	3	GAP[1-3:1-3]#M(0,0)(1,1)(2,2)(3,3)	[37]

Glutamine	GLN	5	GLN[1-5:2-5] #M(0,0)(1,0)(1,1)(2,1)(2,2)(3,2)(3,3)(4,3)(4,4)(5,4)	[37]
Glutamate	GLU	5	GLU[1-5:2-5] #M(0,0)(1,0)(1,1)(2,1)(2,2)(3,2)(3,3)(4,3)(4,4)(5,4)	[37]
Glycine	GLY	2	GLY[1-2:1]#M(0,0)(1,0)(1,1)(2,1)	[37]
Histidine	HIS	6	HIS[1-6:2-6] #M(0,0)(1,0)(1,1)(2,1)(2,2)(3,2)(3,3)(4,3)(4,4)(5,4)(5,5)(6,5)	[37]
Isoleucine	LEU	6	LEU[1-6:2-6] #M(0,0)(1,0)(1,1)(2,1)(2,2)(3,2)(3,3)(4,3)(4,4)(5,4)(5,5)(6,5)	[37]
Leucine	ILEU	6	ILEU[1-6:2-6] #M(0,0)(1,0)(1,1)(2,1)(2,2)(3,2)(3,3)(4,3)(4,4)(5,4)(5,5)(6,5)	[37]
Lysine	LYS	6	LYS[1-6:1-6] #M(0,0)(1,1)(2,2)(3,3)(4,4)(5,5)(6,6)	[37]
Malate	MAL	4	MAL[1-4:1-4]#M(0,0)(1,1)(2,2)(3,3)(4,4)	[37]
Methionine	MET	5	MET[1-5:2-5] #M(0,0)(1,0)(1,1)(2,1)(2,2)(3,2)(3,3)(4,3)(4,4)(5,4)	[37]
Oxaloacetate	OAA	4	OAA[1-4:1-3] #M(0,0)(1,0)(1,1)(2,1)(2,2)(3,2)(3,3)(4,3)	[37]
Phosphoenol-pyruvate	PEP	3	PEP[1-3:1-3]#M(0,0)(1,1)(2,2)(3,3)	[37]
Phenylalanine	PHE	9	PHE[1-9:2-9] #M(0,0)(1,0)(1,1)(2,1)(2,2)(3,2)(3,3)(4,3)(4,4)(5,4)(5,5)(6,5)(6,6)(7,6)(7,7)(8,7)(8,8)(9,8)	[37]
Proline	PRO	5	PRO[1-5:2-5] #M(0,0)(1,0)(1,1)(2,1)(2,2)(3,2)(3,3)(4,3)(4,4)(5,4)	[37]
Pyruvate	PYR	3	PYR[1-3:2-3] #M(0,0)(1,0)(1,1)(2,1)(2,2)(3,2)	[37]
Ribose-5-P	R5P*	5	R5P[1-5:1-5] #M(0,0)(1,1)(2,2)(3,3)(4,4)(5,5)	[37]
Ribulose-5-P + Xylulose-5-P	RU5P	5	RU5P[1-5:1-5] #M(0,0)(1,1)(2,2)(3,3)(4,4)(5,5)	[37]
Sedoheptulose-7-phosphate	S7P	7	S7P[1-7:1-7] #M(0,0)(1,1)(2,2)(3,3)(4,4)(5,5)(6,6)(7,7)	[37]
Serine	SER	3	SER[1-3:2-3] #M(0,0)(1,0)(1,1)(2,1)(2,2)(3,2)	[37]
Succinate	SUC	4	SUC[1-4:1-3] #M(0,0)(1,0)(1,1)(2,1)(2,2)(3,2)(3,3)(4,3) [1-3]=[2-4]	[37]
Homoserine/ Threonine	THR	4	THR[1-4:2-4] #M(0,0)(1,0)(1,1)(2,1)(2,2)(3,2)(3,3)(4,3)	[37]

Tryptophane	TRP*	11	TRP[1-11:1-11] #M(0,0)(1,1)(2,2)(3,3)(4,4)(5,5)(6,6)(7,7)(8,8)(9,9)(10,10)(11,11)	[37]
Tyrosine	TYR	9	TYR[1-9:2-9] #M(0,0)(1,0)(1,1)(2,1)(2,2)(3,2)(3,3)(4,3)(4,4)(5,4)(5,5)(6,5)(6,6)(7,6)(7,7)(8,7)(8,8)(9,8)	[37]
Valine	VAL	5	VAL[1-5:2-5] #M(0,0)(1,0)(1,1)(2,1)(2,2)(3,2)(3,3)(4,3)(4,4)(5,4)	[37]

*: metabolite not present in the reaction network or not used in the study

The tandem-MS measurement group specification METAB[1-3:2,3]#M0,1,2,3 of a C3 metabolite METAB describes a tandem-MS measurement on the full (mother) molecule METAB and on its (daughter) fragment consisting of the second and third atom position. The list of pairs of mass traces following #M denotes the mass-increments of the mother and daughter fragments.

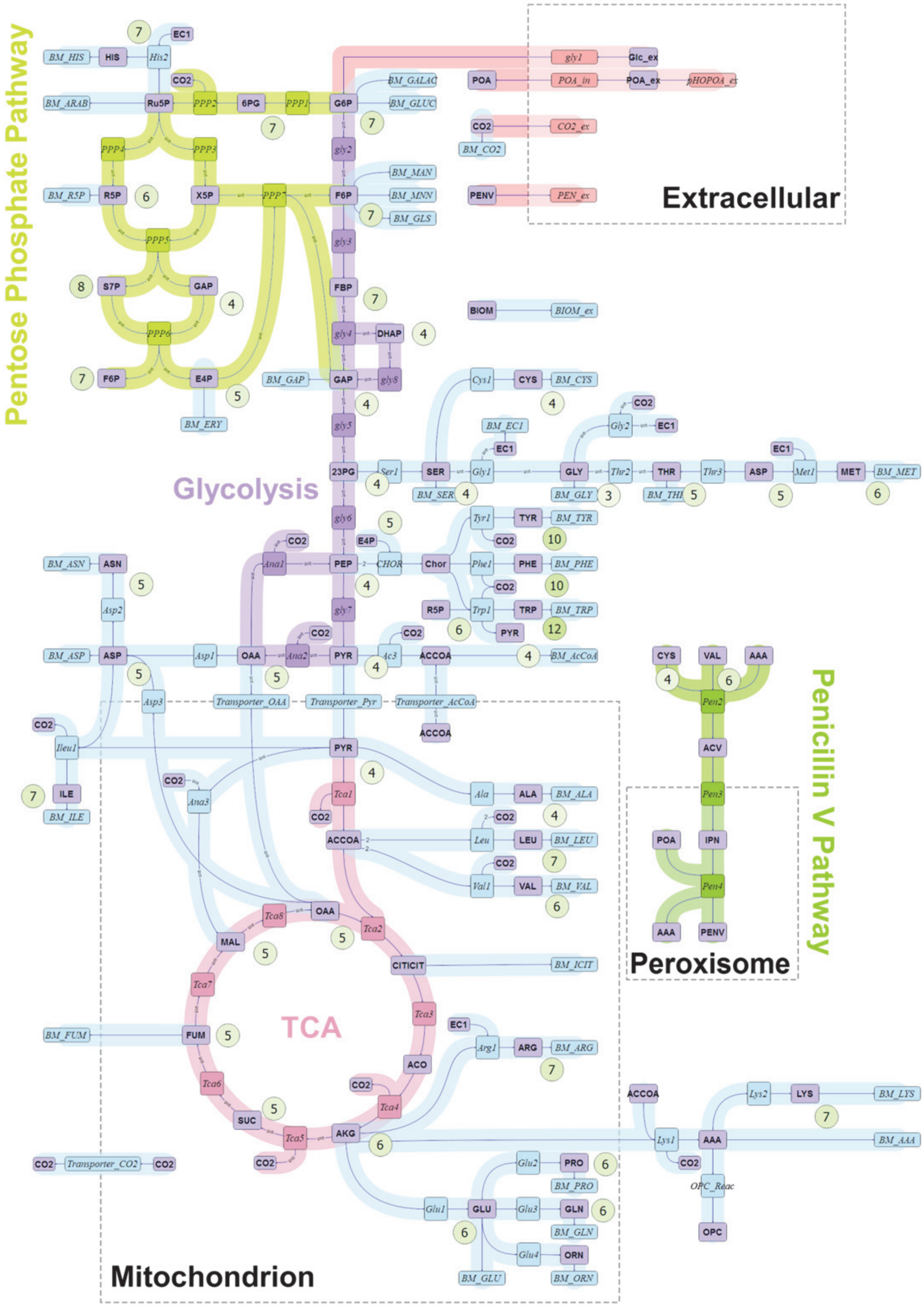

Fig F. Network and LC-MS/MS labeling measurements. Each measurement group is represented by a circle giving the number of measured fractions.

5.2 Measurement model

Tandem mass isotopomers can be expressed – up to a normalization factor – as a linear combination of isotopomer fractions [7], as shown here for the C3 metabolite pyruvate as an example:

$$\begin{pmatrix} PYR[1-3:2-3]\#M(0,0) \\ PYR[1-3:2-3]\#M(1,0) \\ PYR[1-3:2-3]\#M(1,1) \\ PYR[1-3:2-3]\#M(2,1) \\ PYR[1-3:2-3]\#M(2,2) \\ PYR[1-3:2-3]\#M(3,2) \end{pmatrix} = \begin{pmatrix} 1 & 0 & 0 & 0 & 0 & 0 & 0 & 0 \\ 0 & 0 & 0 & 0 & 1 & 0 & 0 & 0 \\ 0 & 1 & 1 & 0 & 0 & 0 & 0 & 0 \\ 0 & 0 & 0 & 0 & 0 & 1 & 1 & 0 \\ 0 & 0 & 0 & 1 & 0 & 0 & 0 & 0 \\ 0 & 0 & 0 & 0 & 0 & 0 & 0 & 1 \end{pmatrix} \cdot \begin{pmatrix} x_{\circ\circ\circ} \\ x_{\circ\circ\bullet} \\ x_{\circ\bullet\circ} \\ x_{\circ\bullet\bullet} \\ x_{\bullet\circ\circ} \\ x_{\bullet\circ\bullet} \\ x_{\bullet\bullet\circ} \\ x_{\bullet\bullet\bullet} \end{pmatrix} = \mathbf{M}_{PYR,MSMS} \cdot \mathbf{x}_{PYR}$$

where $\mathbf{x}_{PYR}$ denotes the vector of isotopomer fractions of the metabolite PYR. In this case C1-3 denotes the mother ion and C2-3 the daughter ion.

5.3 Measurement error model

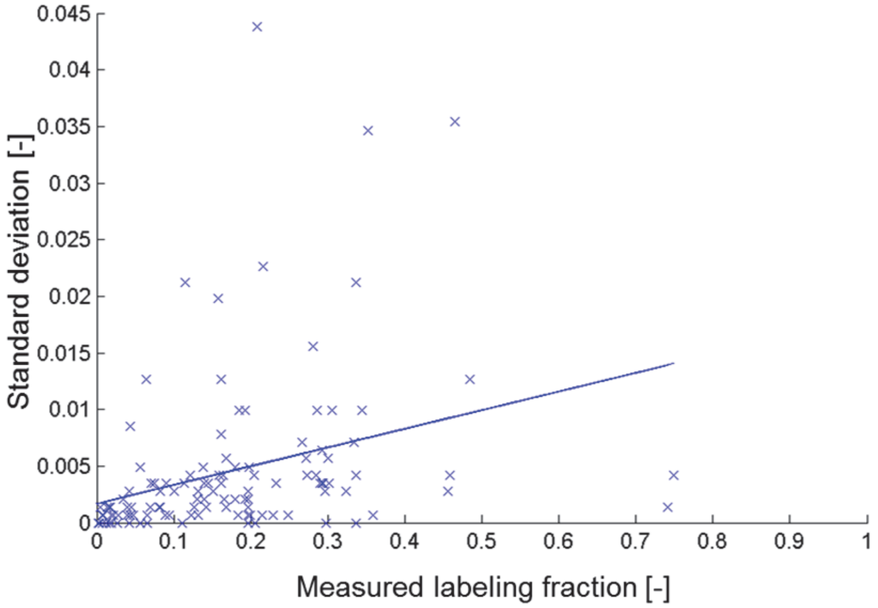

Fig G. Error model for LC-MS/MS based labeling measurements compiled from published data sets (S1 Table D). Standard deviations were determined by linear regression, i.e., by fitting the linear error model $\sigma = b + m\cdot\eta$ with slope (m) and y-axis intercept (b) to the data resulting in $\sigma = 0.001696+0.016496\cdot\eta$.

6. ^{13}C-Nuclear Magnetic Resonance Spectrometry (^{13}C-NMR)

6.1 Measurement specification

Table E. 2D-^{13}C NMR measurement group specification.

**Metabolite**		**Analytical technique**	**Measurement specification**	**References**
Alanine	ALA	^{1}H-^{13}C HSQC	ALA#S2,DL2,DR2,DD2<br>ALA#S3,DL3	[20], [48]
		^{1}H-^{13}C COSY	ALA#S2,DL2,DR2,DD2<br>ALA#S3,DL3	[47], [19]
Arginine	ARG	^{1}H-^{13}C HSQC	ARG#S3,DL3,T3<br>ARG#S5,DL5	[48]
		^{1}H-^{13}C COSY	ARG#S3,DL3,DD3<br>ARG#S4,DL4,DD4<br>ARG#S5,DL5	[19]
			ARG#S3,DL3,T3<br>ARG#S5,DL5	[47]
Aspartate	ASP	^{1}H-^{13}C HSQC	ASP#S2,DL2,DR2,DD2<br>ASP#S3,DL3,DR3,DD3	[20], [48]
		^{1}H-^{13}C COSY	ASP#S2,DL2,DR2,DD2<br>ASP#S3,DL3,DR3,DD3	[47]
Aspartate/<br>Asparagine	ASX*	^{1}H-^{13}C COSY	ASX#S2,DL2,DR2,DD2<br>ASX#S3,DL3,DR3,DD3	[19]
Cysteine	CYS	^{1}H-^{13}C COSY	CYS#S2,DL2,DR2,DD2<br>CYS#S3,DL3	[47]
Glutamate	GLU	^{1}H-^{13}C HSQC	GLU#S2,DL2,DR2,DD2<br>GLU#S3,DL3,DD3<br>GLU#S4,DL4,DR4,DD4	[20]
		^{1}H-^{13}C COSY	GLU#S2,DL2,DR2,DD2<br>GLU#S3,DL3,T3<br>GLU#S4,DL4,DR4,DD4	[47]
Glutamate/<br>Glutamine	GLX*	^{1}H-^{13}C COSY	GLX#S2,DL2,DR2,DD2<br>GLX#S3,DL3,DD3<br>GLX#S4,DL4,DR4,DD4	[19]
Glycine	GLY	^{1}H-^{13}C HSQC	GLY#S2,DL2	[20], [48]
		^{1}H-^{13}C COSY	GLY#S2,DL2	[47], [19]
Histidine	HIS	^{1}H-^{13}C HSQC	HIS#S2,DL2,DR2,DD2<br>HIS#S3,DL3,DR3,DD3<br>HIS#S5,DL5	[20]
			HIS# S3,DL3,DR3,DD3<br>HIS#S5,DL5	[48]
		^{1}H-^{13}C COSY	HIS# S2,DL2,DR2,DD2<br>HIS# S3,DL3,DR3,DD3	[47], [19]
Isoleucine	ILE	^{1}H-^{13}C HSQC	ILE#S2,DL2,DR2,DD2	[20]
			ILE#S2,DL2,DR2,DD2<br>ILE#S4,DL4,T4<br>ILE#S5,DL5<br>ILE#S6,DL6	[48]
		^{1}H-^{13}C COSY	ILE#S2,DL2,DR2,DD2<br>ILE#S4,DL4,DD4<br>ILE#S5,DL5	[19]

			ILE#S6,DL6	
			ILE#S3,DL3,T3<br>ILE#S5,DL5	[47]
Leucine	LEU	^{1}H-^{13}C HSQC	LEU#S2,DL2,DR2,DD2<br>LEU#S3,DL3,DD3	[20]
			LEU#S2,DL2,DR2,DD2<br>LEU#S3,DL3,T3<br>LEU#S5,DL5<br>LEU#S6,DL6	[48]
		^{1}H-^{13}C COSY	LEU#S2,DL2,DR2,DD2<br>LEU#S3,DL3,DD3<br>LEU#S5,DL5<br>LEU#S6,DL6	[19]
			LEU#S2,DL2,DR2,DD2<br>LEU#S3,DL3,T3<br>LEU#S5,DL5<br>LEU#S6,DL6	[47]
Lysine	LYS	^{1}H-^{13}C HSQC	LYS#S3,DL3,T3<br>LYS#S4,DL4,T4<br>LYS#S5,DL5,T5	[48]
		^{1}H-^{13}C COSY	LYS#S3,DL3,DD3<br>LYS#S4,DL4,DD4<br>LYS#S5,DL5,DD5<br>LYS#S6,DL6	[19]
			LYS#S3,DL3,T3<br>LYS#S4,DL4,T4	[47]
Methionine	MET	^{1}H-^{13}C COSY	MET#S2,DL2,DR2,DD2	[47], [19]
Phenylalanine	PHE	^{1}H-^{13}C HSQC	PHE#S2,DL2,DR2,DD2<br>PHE#S3,DL3,DD3	[20]
			PHE#S2,DL2,DR2,DD2	[48]
		^{1}H-^{13}C COSY	PHE#S2,DL2,DR2,DD2<br>PHE#S3,DL3,DD3	[19]
			PHE#S2,DL2,DR2,DD2<br>PHE#S3,DL3,DR3,DD3	[47]
Proline	PRO	^{1}H-^{13}C HSQC	PRO#S2,DL2,DR2,DD2<br>PRO#S5,DL5	[20]
			PRO#S2,DL2,DR2,DD2<br>PRO#S3,DL3,T3	[48]
		^{1}H-^{13}C COSY	PRO#S2,DL2,DR2,DD2<br>PRO#S3,DL3,DD3<br>PRO#S4,DL4,DD4<br>PRO#S5,DL5	[19]
			PRO#S2,DL2,DR2,DD2<br>PRO#S3,DL3	[47]
Serine	SER	^{1}H-^{13}C HSQC	SER#S2,DL2,DR2,DD2<br>SER#S3,DL3	[20],<br>[48]
		^{1}H-^{13}C COSY	SER#S2,DL2,DR2,DD2<br>SER#S3,DL3	[47], [19]
Threonine	THR	^{1}H-^{13}C HSQC	THR#S4,DL4	[20], [48]
		^{1}H-^{13}C COSY	THR#S2,DL2,DR2,DD2<br>THR#S3,DL3,DD3<br>THR#S4,DL4	[19]
			THR#S2,DL2,DR2,DD2<br>THR#S3,DL3,T3	[47]

			THR#S4,DL4	
Tyrosine	TYR	^{1}H-^{13}C HSQC	TYR#S5,DL5,T5	[48]
			TYR#S5,DL5,DD5<br>TYR#S6,DL6,DD6	[20]
		^{1}H-^{13}C COSY	TYR#S2,DL2,DR2,DD2<br>TYR#S3,DL3,DR3,DD3	[47], [19]
Valine	VAL	^{1}H-^{13}C HSQC	VAL#S2,DL2,DR2,DD2<br>VAL#S3,DL3,DD3,DD3	[20]
			VAL#S2,DL2,DR2,DD2<br>VAL#S4,DL4<br>VAL#S5,DL5	[48]
		^{1}H-^{13}C COSY	VAL#S2,DL2,DR2,DD2<br>VAL#S4,DL4<br>VAL#S5,DL5	[19]
			VAL#S2,DL2,DR2,DD2<br>VAL#S4,DL4	[47]

*: metabolite not represented in the reaction network or used in the study

^{1}H-^{13}C HSQC: two-dimensional heteronuclear single-quantum coherence (^{13}C, ^{1}H)-correlation NMR

^{1}H-^{13}C COSY: two dimensional heteronuclear correlation spectroscopy

NMR fine structures are specified by singlet (S), doublets (D), double doublets (DD), and triplets (T), where DL and DR denote the left and right doublet, respectively, followed by the measured position.

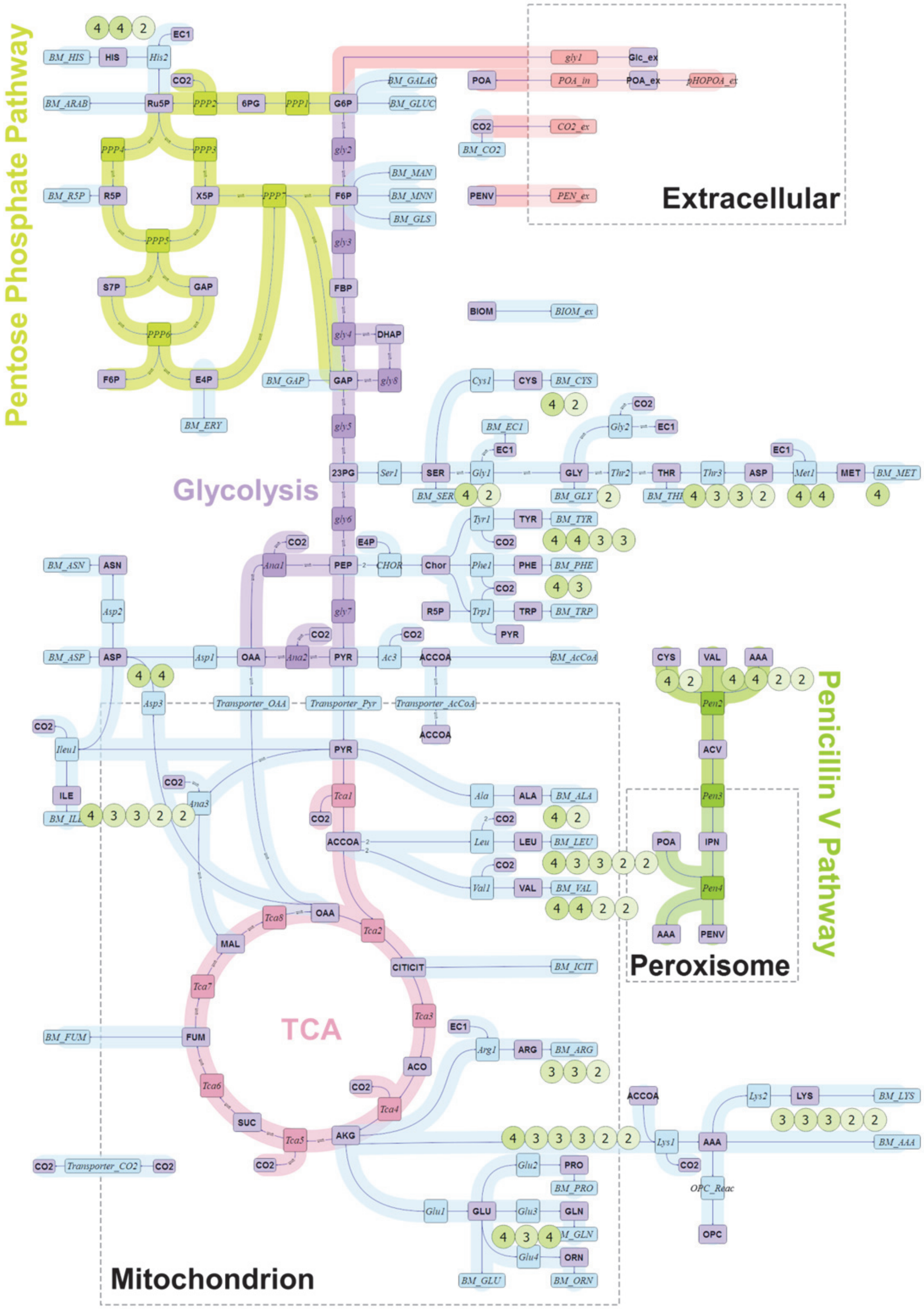

Fig H. Network and ^{13}C-NMR labeling measurements. Each measurement group is represented by a circle giving the number of measured fractions.

6.2 Measurement model

Multiplet resonances can be expressed – up to a normalization factor – as a linear combination of isotopomer fractions [51], as shown here for the C3 metabolite pyruvate as an example:

$$\begin{pmatrix} PYR\#S2 \\ PYR\#DL2 \\ PYR\#DR2 \\ PYR\#DD2 \end{pmatrix} = \begin{pmatrix} 0 & 0 & 1 & 0 & 0 & 0 & 0 & 0 \\ 0 & 0 & 0 & 0 & 0 & 0 & 1 & 0 \\ 0 & 0 & 0 & 1 & 0 & 0 & 0 & 0 \\ 0 & 0 & 0 & 0 & 0 & 0 & 0 & 1 \end{pmatrix} \cdot \begin{pmatrix} x_{\circ\circ\circ} \\ x_{\circ\circ\bullet} \\ x_{\circ\bullet\circ} \\ x_{\circ\bullet\bullet} \\ x_{\bullet\circ\circ} \\ x_{\bullet\circ\bullet} \\ x_{\bullet\bullet\circ} \\ x_{\bullet\bullet\bullet} \end{pmatrix} = \mathbf{M}_{PYR[2],CNMR} \cdot \mathbf{x}_{PYR}$$

where $\mathbf{x}_{PYR}$ denotes the vector of isotopomer fractions of the metabolite PYR. PYR[2] indicates that the second carbon atom is observed.

6.3 Measurement error model

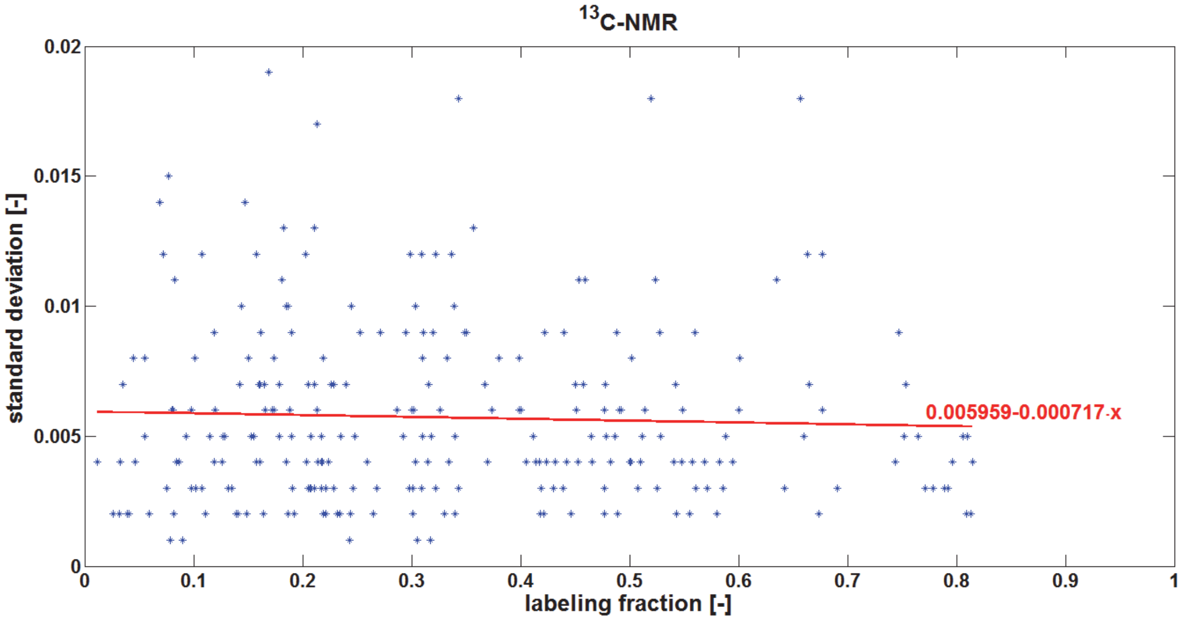

Fig I. Error model for ^{13}C-NMR based labeling measurements compiled from published data sets (S1 Table E). Standard deviations were determined by linear regression, i.e., by fitting the linear error model $\sigma = b + m \cdot \eta$ with slope (m) and y-axis intercept (b) to the data resulting in $\sigma = 0.005959+0.000717 \cdot \eta$.

7. ^{1}H-Nuclear Magnetic Resonance Spectrometry (^{1}H-NMR)

7.1 Measurement specification

Table F. ^{1}H-NMR measurement group specification.

Metabolite		#carbons	Measurement specification	References
Acetate	ACE*	2	ACE#P1,2	[42]
Alanine	ALA	3	ALA#P2,3	[42], [53]
Aspartate	ASP	4	ASP#P2,3	[53]
Glutamate	GLU	6	GLU#P2,3,4	[53]
Glycine	GLY	2	GLY#P1,2	[53]
Isoleucine	ILE	6	ILE#P2,5,6	[53]
Leucine	LEU	6	LEU#P2,3,4,5,6	[53]
Lysine	LYS*	6	LYS#P2,3,4,5,6	[53]
Phenylalanine	PHE	9	PHE#P2,3,4,5,6,7,8,9	[53]
Serine	SER	3	SER#P2,3	[53]
Succinate	SUC*	4	SUC#P1,2; [1]=[4], [2]=[3]	[47]
Threonine	THR	4	THR#P2,3,4	[53]
Valine	VAL	5	VAL#P4,5	[53]

*: metabolite not represented in the reaction network or used in the study

One-dimensional ^{1}H-NMR allows for the measurement of positional ^{13}C-enrichments. The observed positional enrichments are denoted by a leading “P” followed by the position(s). An example for the corresponding measurement model is given in S1 Sec 7.2.

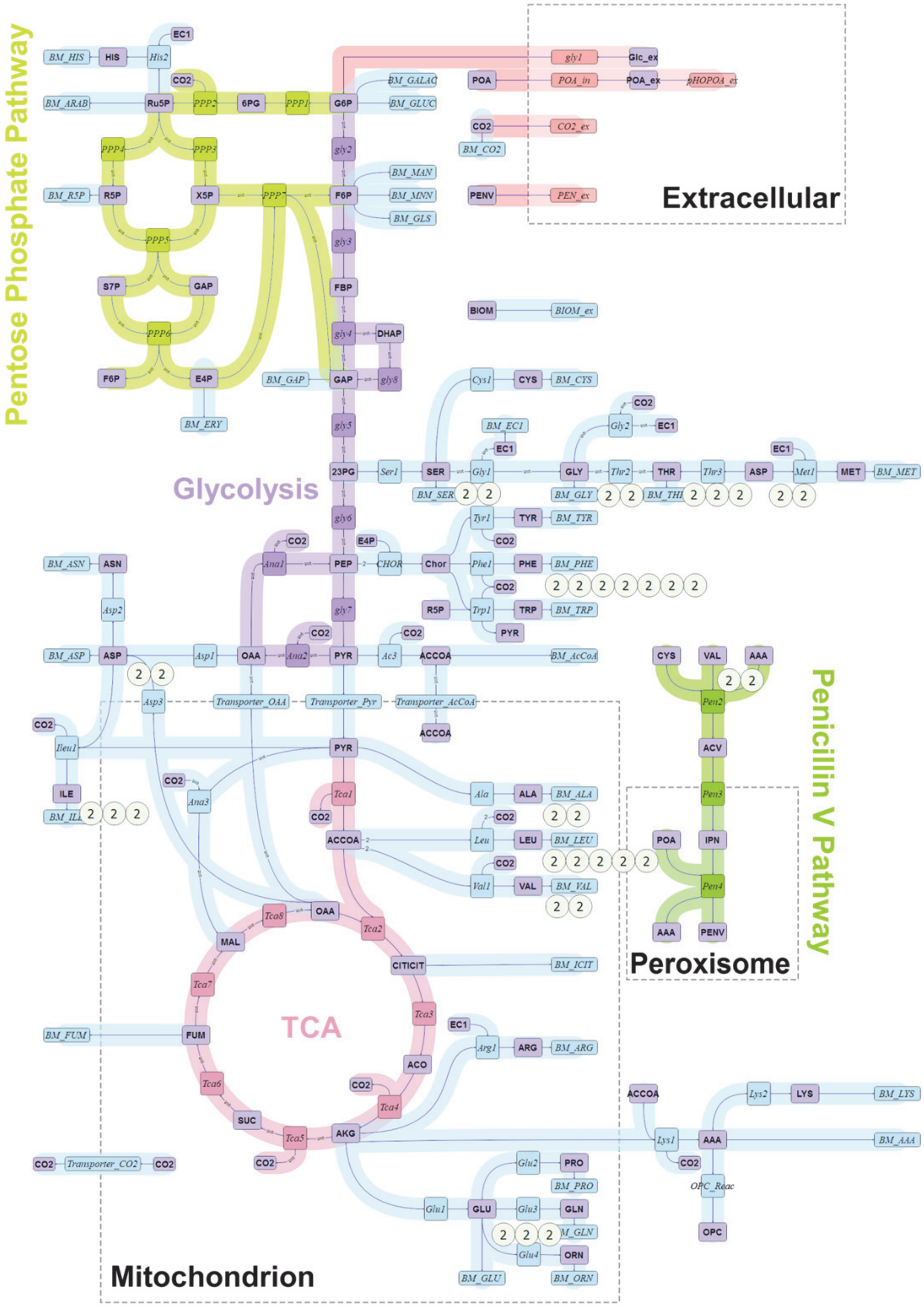

Fig J. Network and ^{1}H-NMR labeling measurements. Each measurement group is represented by a circle giving the number of measured fractions.

7.2 Measurement model

Positional enrichments can be expressed – up to a normalization factor – as a linear combination of isotopomer fractions [51], as shown here for the C3 metabolite pyruvate as an example:

$$PYR\#\mathrm{P1}:\begin{pmatrix} PYR[1]\#0 \\ PYR[1]\#1 \end{pmatrix} = \begin{pmatrix} 1 & 1 & 1 & 1 & 0 & 0 & 0 & 0 \\ 0 & 0 & 0 & 0 & 1 & 1 & 1 & 1 \end{pmatrix} \cdot \begin{pmatrix} x_{\circ\circ\circ} \\ x_{\circ\circ\bullet} \\ x_{\circ\bullet\circ} \\ x_{\circ\bullet\bullet} \\ x_{\bullet\circ\circ} \\ x_{\bullet\circ\bullet} \\ x_{\bullet\bullet\circ} \\ x_{\bullet\bullet\bullet} \end{pmatrix} = \mathbf{M}_{PYR[1],HNMR} \cdot \mathbf{x}_{PYR}$$

where $\mathbf{x}_{PYR}$ denotes the vector of isotopomer fractions of the metabolite PYR. P1 indicates the first C atom position is observed.

7.3 Measurement error model

Up to now, for ^{1}H-NMR only data-points having a low abundance of ^{13}C labeling content have been published. Errors of observables with high labeling content were linearly extrapolated from these data.

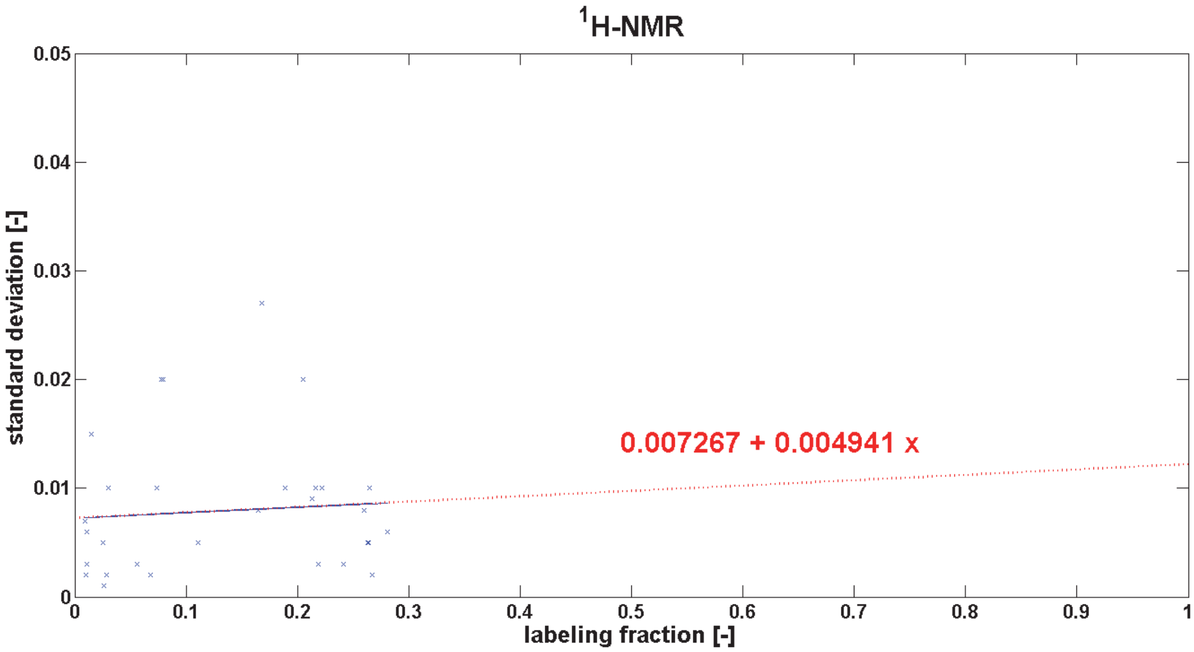

Fig K. Error model for ^{1}H-NMR based labeling measurements compiled from published data sets (S1 Table F). Standard deviations were determined by linear regression, i.e., by fitting the linear error model $\sigma = b + m \cdot \eta$ with slope (m) and y-axis intercept (b) to the data resulting in $\sigma = 0.007267 + 0.004941 \cdot \eta$ (blue line). For data with higher labeling incorporation than 30%, standard deviations were linearly extrapolated (red dotted line).

8. Gas Chromatography-Combustion-Isotope Ratio Mass Spectrometry (GC-C-IRMS)

8.1 Measurement specification

Table G. GC-C-IRMS measurement group specification.

Metabolite		# Carbons	Measurement specification	Reference
Alanine	ALA	3	ALA#M0,1	[10]
Aspartate	ASP	4	ASP#M0,1	[10]
Glutamate	GLU	6	GLU#M0,1	[10]
Glycine	GLY	2	GLY#M0,1	[10]
Histidine	HIS	6	HIS#M0,1	[10]
Isoleucine	ILE	6	ILE#M0,1	[10]
Leucine	LEU	6	LEU#M0,1	[10]
Phenylalanine	PHE	9	PHE#M0,1	[10]
Proline	PRO	5	PRO#M0,1	[10]
Serine	SER	3	SER#M0,1	[10]
Threonine	THR	4	THR#M0,1	[10]
Valine	VAL	5	VAL#M0,1	[10]

GC-C-IRMS permits the measurement of the $^{13}C/^{12}C$ ratio. Typically, measurements are given in $\delta^{13}C$, denoting the ^{13}C content (expressed in per mill) which is converted into ^{13}C enrichment. Thus, METAB#M0,1 denotes the content of ^{12}C to ^{13}C isotope fractions for a metabolite METAB. An example for the corresponding measurement model is given in S1 Sec 8.2.

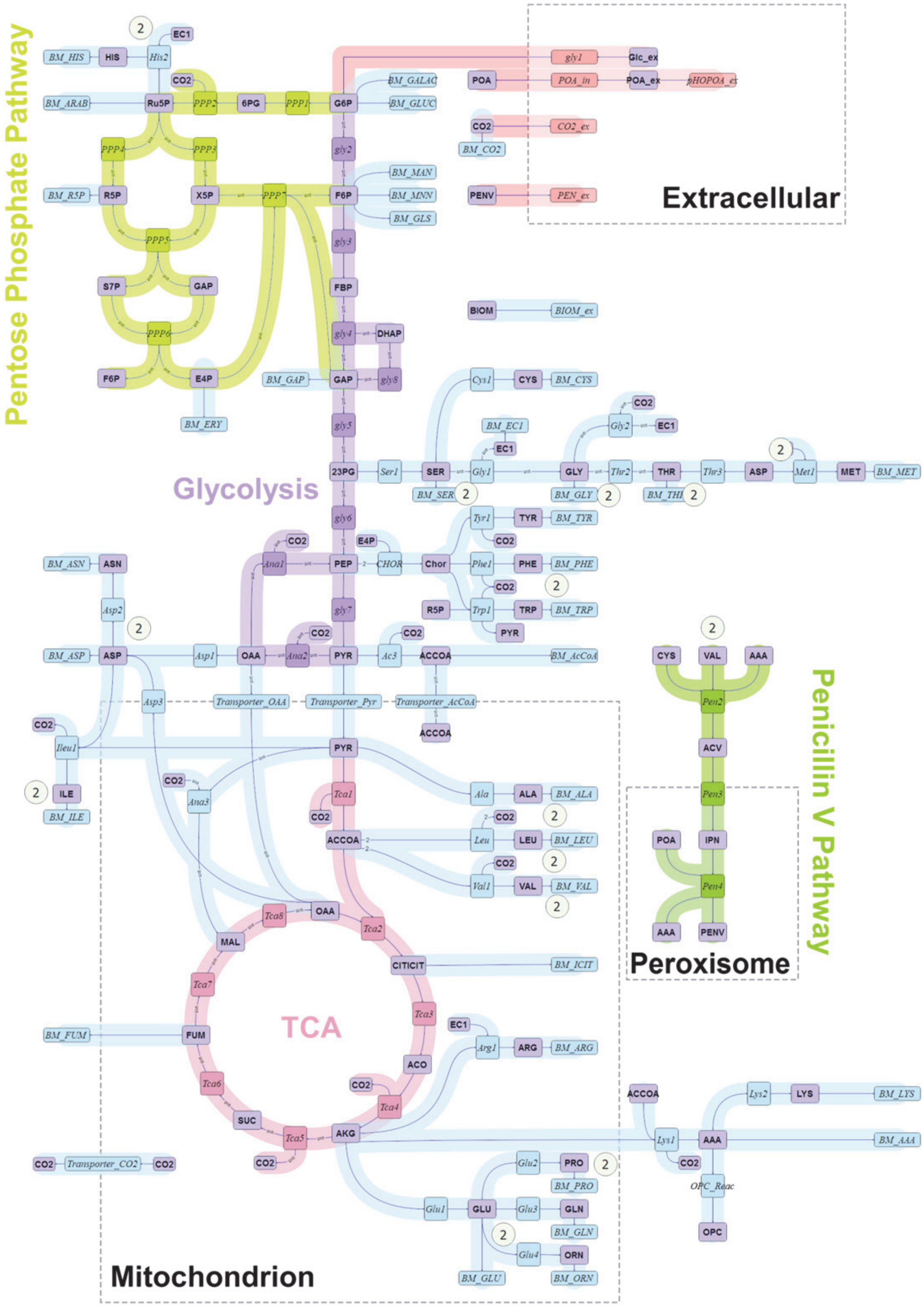

Fig L. Network and GC-C-IRMS labeling measurements. Each measurement group is represented by a circle giving the number of measured fractions.

8.2 Measurement model

GC-C-IRMS enrichments can be expressed as a linear combination of isotopomer fractions weighted by the ratio of (un)labeled carbon atoms and total number of carbon atoms, as shown here for the C3 metabolite pyruvate as an example:

$$\begin{pmatrix} PYR\#0 \\ PYR\#1 \end{pmatrix} = \begin{pmatrix} 1 & 2/3 & 2/3 & 1/3 & 2/3 & 1/3 & 1/3 & 0 \\ 0 & 1/3 & 1/3 & 2/3 & 1/3 & 2/3 & 2/3 & 1 \end{pmatrix} \cdot \begin{pmatrix} x_{\circ\circ\circ} \\ x_{\circ\circ\bullet} \\ x_{\circ\bullet\circ} \\ x_{\circ\bullet\bullet} \\ x_{\bullet\circ\circ} \\ x_{\bullet\circ\bullet} \\ x_{\bullet\bullet\circ} \\ x_{\bullet\bullet\bullet} \end{pmatrix} = \mathbf{M}_{PYR,CIRMS} \cdot \mathbf{x}_{PYR}$$

where $\mathbf{x}_{PYR}$ denotes the vector of isotopomer fractions of the metabolite PYR.

8.3 Measurement error model

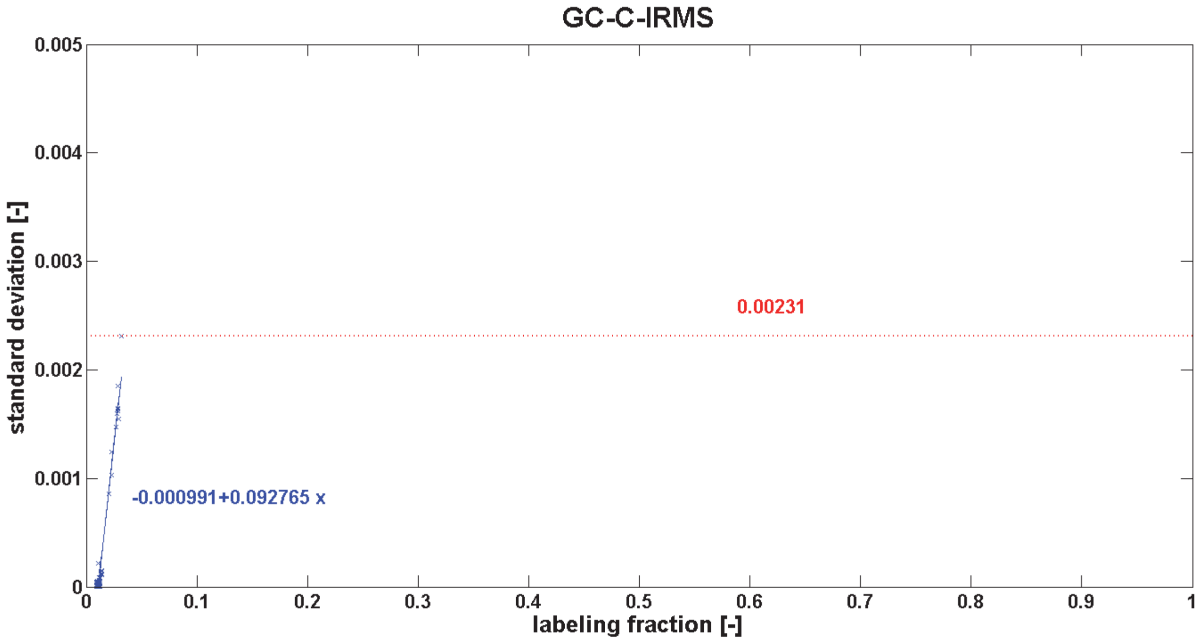

Fig M. Error model for GC-C-IRMS based labeling measurements compiled from published data sets (S1 Table G). Standard deviations were determined by linear regression, i.e., by fitting the linear error model $\sigma = b + m \cdot \eta$ with slope (m) and y-axis intercept (b) to the data resulting in $-0.000991+0.092765 \cdot \eta$. Since only metabolomics data with a low labeling incorporation were available, instead of this error model a defensive constant error model was chosen, determined by the largest error value: $\sigma = 0.0231$.

9. References

1. Watson JT, Sparkman OD. Introduction to Mass Spectrometry. 4th ed. Introduction to Mass Spectrometry: Instrumentation, Applications and Strategies for Data Interpretation: Fourth Edition. Chichester, UK: John Wiley & Sons, Ltd; 2007. doi:10.1002/9780470516898
2. Siuzdak G. The Expanding Role of Mass Spectrometry in Biotechnology. 2nd ed. San Diego, CA: MCC Press; 2006.
3. Marx A, de Graaf AA, Wiechert W, Eggeling L, Sahm H. Determination of the fluxes in the central metabolism of *Corynebacterium glutamicum* by nuclear magnetic resonance spectroscopy combined with metabolite balancing. Biotechnol Bioeng. 1996;49: 111–129. doi:10.1002/(SICI)1097-0290(19960120)49:2<111::AID-BIT1>3.0.CO;2-T
4. Szyperski T. Biosynthetically directed fractional ^{13}C-labeling of proteinogenic amino acids. An efficient analytical tool to investigate intermediary metabolism. Eur J Biochem. 1995;232: 433–448. doi:10.1111/j.1432-1033.1995.433zz.x
5. Massou S, Nicolas C, Letisse F, Portais J-C. NMR-based fluxomics: quantitative 2D NMR methods for isotopomers analysis. Phytochemistry. 2007;68: 2330–2340. doi:10.1016/j.phytochem.2007.03.011
6. Wittmann C, Heinzle E. Mass spectrometry for metabolic flux analysis. Biotechnol Bioeng. 1999;62: 739–750. doi:10.1002/(SICI)1097-0290(19990320)62:6<739::AID-BIT13>3.0.CO;2-E
7. Rühl M, Rupp B, Nöh K, Wiechert W, Sauer U, Zamboni N. Collisional fragmentation of central carbon metabolites in LC-MS/MS increases precision of ^{13}C metabolic flux analysis. Biotechnol Bioeng. 2012;109: 763–771. doi:10.1002/bit.24344
8. Choi J, Antoniewicz MR. Tandem mass spectrometry: a novel approach for metabolic flux analysis. Metab Eng. 2010/12/08. 2011;13: 225–233. doi:10.1016/j.ymben.2010.11.006
9. McCloskey D, Young JD, Xu S, Palsson BO, Feist AM. MID Max: LC-MS/MS method for measuring the precursor and product mass isotopomer distributions of metabolic intermediates and cofactors for metabolic flux analysis applications. Anal Chem. 2016;88: 1362–1370. doi:10.1021/acs.analchem.5b03887
10. Yuan Y, Hoon Yang T, Heinzle E. ^{13}C metabolic flux analysis for larger scale cultivation using gas chromatography-combustion-isotope ratio mass spectrometry. Metab Eng. 2010;12: 392–400. doi:10.1016/j.ymben.2010.02.001
11. Higashi RM, Fan TW, Lorkiewicz PK, Moseley HNB, Lane AN. Stable isotope-labeled tracers for metabolic pathway elucidation by GC-MS and FT-MS. In: Raftery D, editor. Mass Spectrometry in Metabolomics. New York, NY: Springer New York; 2014. pp. 147–167. doi:10.1007/978-1-4939-1258-2
12. Godin JP, Faure M, Breuille D, Hopfgartner G, Fay LB. Determination of ^{13}C isotopic enrichment of valine and threonine by GC-C-IRMS after formation of the N(O,S)-ethoxycarbonyl ethyl ester derivatives of the amino acids. Anal Bioanal Chem. 2007;388: 909–918. doi:10.1007/s00216-007-1275-2
13. Wong WW, Hachey DL, Zhang S, Clarke LL. Accuracy and precision of gas chromatography/combustion isotope ratio mass spectrometry for stable carbon isotope ratio measurements. Rapid Commun Mass Spectrom. 1995;9: 1007–1011. doi:10.1002/rcm.1290091107
14. Wittmann C, Heinzle E. Application of MALDI-TOF MS to lysine-producing *Corynebacterium glutamicum*: a novel approach for metabolic flux analysis. Eur J Biochem. 2001;268: 2441–2455. doi:10.1046/j.1432-1327.2001.02129.x
15. Tang Y, Pingitore F, Mukhopadhyay A, Phan R, Hazen TC, Keasling JD. Pathway confirmation and flux analysis of central metabolic pathways in *Desulfovibrio vulgaris hildenborough* using gas chromatography-mass spectrometry and Fourier transform-ion cyclotron resonance mass spectrometry. J Bacteriol. 2007;189: 940–949. doi:10.1128/JB.00948-06
16. Szyperski T, Glaser RW, Hochuli M, Fiaux J, Sauer U, Bailey JE, et al. Bioreaction

network topology and metabolic flux ratio analysis by biosynthetic fractional ^{13}C labeling and two-dimensional NMR spectroscopy. Metab Eng. 1999;1: 189–197. doi:10.1006/mben.1999.0116
17. Emmerling M, Dauner M, Ponti A, Fiaux J, Hochuli M, Szyperski T, et al. Metabolic flux responses to pyruvate kinase knockout in *Escherichia coli*. J Bacteriol. 2002;184: 152–164. doi:10.1128/JB.184.1.152-164.2002
18. Dauner M, Bailey JE, Sauer U. Metabolic flux analysis with a comprehensive isotopomer model in *Bacillus subtilis*. Biotechnol Bioeng. 2001;76: 144–156. doi:10.1002/bit.1154
19. Schmidt K, Nielsen J, Villadsen J. Quantitative analysis of metabolic fluxes in *Escherichia coli*, using two-dimensional NMR spectroscopy and complete isotopomer models. J Biotechnol. 1999;71: 175–189. doi:10.1016/S0168-1656(99)00021-8
20. Flores S, Gosset G, Flores N, de Graaf AA, Bolívar F. Analysis of carbon metabolism in *Escherichia coli* strains with an inactive phosphotransferase system by ^{13}C labeling and NMR spectroscopy. Metab Eng. 2002;4: 124–137. doi:10.1006/mben.2001.0209
21. Fischer E, Sauer U. Metabolic flux profiling of *Escherichia coli* mutants in central carbon metabolism using GC-MS. Eur J Biochem. 2003;270: 880–891. doi:10.1046/j.1432-1033.2003.03448.x
22. Ahn WS, Antoniewicz MR. Metabolic flux analysis of CHO cells at growth and non-growth phases using isotopic tracers and mass spectrometry. Metab Eng. 2011;13: 598–609. doi:10.1016/j.ymben.2011.07.002
23. Becker J, Klopprogge C, Wittmann C. Metabolic responses to pyruvate kinase deletion in lysine producing *Corynebacterium glutamicum*. Microb Cell Fact. 2008;7: 8. doi:10.1186/1475-2859-7-8
24. Christensen B, Nielsen J. Isotopomer analysis using GC-MS. Metab Eng. 1999;1: 282–290. doi:10.1006/mben.1999.0117
25. Dauner M, Sauer U. GC-MS analysis of amino acids rapidly provides rich information for isotopomer balancing. Biotechnol Prog. 2000;16: 642–649. doi:10.1021/bp000058h
26. Wittmann C. Fluxome analysis using GC-MS. Microb Cell Fact. 2007;6: 6. doi:10.1186/1475-2859-6-6
27. Suthers PF, Burgard AP, Dasika MS, Nowroozi F, van Dien S, Keasling JD, et al. Metabolic flux elucidation for large-scale models using ^{13}C labeled isotopes. Metab Eng. 2007;9: 387–405. doi:10.1016/j.ymben.2007.05.005
28. Antoniewicz MR, Kelleher JK, Stephanopoulos G. Accurate assessment of amino acid mass isotopomer distributions for metabolic flux analysis. Anal Chem. 2007;79: 7554–7559. doi:10.1021/ac0708893
29. Fischer E, Zamboni N, Sauer U. High-throughput metabolic flux analysis based on gas chromatography-mass spectrometry derived ^{13}C constraints. Anal Biochem. 2004;325: 308–316. doi:10.1016/j.ab.2003.10.036
30. Fiaux J, Çakar ZP, Sonderegger M, Wüthrich K, Szyperski T, Sauer U. Metabolic-flux profiling of the yeasts *Saccharomyces cerevisiae* and *Pichia stipitis*. Eukaryot Cell. 2003;2: 170–180. doi:10.1128/EC.2.1.170
31. Crown SB, Indurthi DC, Ahn WS, Choi J, Papoutsakis ET, Antoniewicz MR. Resolving the TCA cycle and pentose-phosphate pathway of *Clostridium acetobutylicum* ATCC 824: Isotopomer analysis, in vitro activities and expression analysis. Biotechnol J. 2011;6. doi:10.1002/biot.201000282
32. Heinzle E, Yuan Y, Kumar S, Wittmann C, Gehre M, Richnow H-H, et al. Analysis of ^{13}C labeling enrichment in microbial culture applying metabolic tracer experiments using gas chromatography-combustion-isotope ratio mass spectrometry. Anal Biochem. 2008;380: 202–210. doi:10.1016/j.ab.2008.05.039
33. Nöh K, Grönke K, Luo B, Takors R, Oldiges M, Wiechert W, et al. Metabolic flux analysis at ultra short time scale: isotopically non-stationary ^{13}C labeling experiments. J Biotechnol. 2007;129: 249–267. doi:10.1016/j.jbiotec.2006.11.015
34. van Winden WA, van Dam JC, Ras C, Kleijn RJ, Vinke JL, van Gulik WM, et al. Metabolic-flux analysis of CEN.PK113-7D based on mass isotopomer measurements of ^{13}C-labeled primary metabolites. FEMS Yeast Res. 2005;5: 559–568.

doi:10.1016/j.femsyr.2004.10.007
35. Kleijn RJ, Geertman J-MA, Nfor BK, Ras C, Schipper D, Pronk JT, et al. Metabolic flux analysis of a glycerol-overproducing *Saccharomyces cerevisiae* strain based on GC-MS, LC-MS and NMR-derived ^{13}C-labelling data. FEMS Yeast Res. 2007;7: 216–231. doi:10.1111/j.1567-1364.2006.00180.x
36. Iwatani S, van Dien S, Shimbo K, Kubota K, Kageyama N, Iwahata D, et al. Determination of metabolic flux changes during fed-batch cultivation from measurements of intracellular amino acids by LC-MS/MS. J Biotechnol. 2007;128: 93–111. doi:10.1016/j.jbiotec.2006.09.004
37. van Ooyen J, Noack S, Bott M, Reth A, Eggeling L. Improved L-lysine production with *Corynebacterium glutamicum* and systemic insight into citrate synthase flux and activity. Biotechnol Bioeng. 2012;109: 2070–2081. doi:10.1002/bit.24486
38. Moseley HNB, Lane AN, Belshoff AC, Higashi RM, Fan TWM. A novel deconvolution method for modeling UDP-N-acetyl-D-glucosamine biosynthetic pathways based on ^{13}C mass isotopologue profiles under non-steady-state conditions. BMC Biol. 2011;9: 37. doi:10.1186/1741-7007-9-37
39. Pingitore F, Tang Y, Kruppa GH, Keasling JD. Analysis of amino acid isotopomers using FT-ICR MS. Anal Chem. 2007;79: 2483–2490. doi:10.1021/ac061906b
40. Jeffrey FMH, Roach JS, Storey CJ, Sherry AD, Malloy CR. ^{13}C isotopomer analysis of glutamate by tandem mass spectrometry. Anal Biochem. 2002;300: 192–205. doi:10.1006/abio.2001.5457
41. Okahashi N, Kawana S, Iida J, Shimizu H, Matsuda F. GC-MS/MS survey of collision-induced dissociation of tert-butyldimethylsilyl-derivatized amino acids and its application to ^{13}C-metabolic flux analysis of *Escherichia coli* central metabolism. Anal Bioanal Chem. Analytical and Bioanalytical Chemistry; 2016;408: 6133–6140. doi:10.1007/s00216-016-9724-4
42. McKinlay JB, Shachar-Hill Y, Zeikus JG, Vieille C. Determining *Actinobacillus succinogenes* metabolic pathways and fluxes by NMR and GC-MS analyses of ^{13}C-labeled metabolic product isotopomers. Metab Eng. 2007;9: 177–192. doi:10.1016/j.ymben.2006.10.006
43. Kleijn RJ, Liu F, van Winden WA, van Gulik WM, Ras C, Heijnen JJ. Cytosolic NADPH metabolism in penicillin-G producing and non-producing chemostat cultures of *Penicillium chrysogenum*. Metab Eng. 2007;9: 112–123. doi:10.1016/j.ymben.2006.08.004
44. Lien SK, Sletta H, Ellingsen TE, Valla S, Correa E, Goodacre R, et al. Investigating alginate production and carbon utilization in *Pseudomonas fluorescens* SBW25 using mass spectrometry-based metabolic profiling. Metabolomics. 2012;9: 403–417. doi:10.1007/s11306-012-0454-0
45. Alonso AP, Val DL, Shachar-Hill Y. Central metabolic fluxes in the endosperm of developing maize seeds and their implications for metabolic engineering. Metab Eng. 2011;13: 96–107. doi:10.1016/j.ymben.2010.10.002
46. Antoniewicz MR, Kelleher JK, Stephanopoulos G. Elementary metabolite units (EMU): a novel framework for modeling isotopic distributions. Metab Eng. 2007;9: 68–86. doi:10.1016/j.ymben.2006.09.001
47. Kleijn RJ. Development and Application of ^{13}C-Labeling Techniques: Analyzing the Pentose Phosphate Pathway of *Penicillium chrysogenum*. Delft University of Technology. 2007.
48. Choudhary MK, Yoon JM, Gonzalez R, Shanks J V. Re-examination of metabolic fluxes in *Escherichia coli* during anaerobic fermentation of glucose using ^{13}C labeling experiments and 2-dimensional nuclear magnetic resonance (NMR) spectroscopy. Biotechnol Bioprocess Eng. 2011;16: 419–437. doi:10.1007/s12257-010-0449-5
49. Schaub J, Mauch K, Reuss M. Metabolic flux analysis in *Escherichia coli* by integrating isotopic dynamic and isotopic stationary ^{13}C labeling data. Biotechnol Bioeng. 2008;99: 1170–1185. doi:10.1002/bit.21675
50. Droste P, Nöh K, Wiechert W. Omix - A visualization tool for metabolic networks with highest usability and customizability in focus. Chemie Ing Tech. 2013;85: 849–862.

doi:10.1002/cite.201200234
51. Möllney M, Wiechert W, Kownatzki D, de Graaf AA. Bidirectional reaction steps in metabolic networks: IV. Optimal design of isotopomer labeling experiments. Biotechnol Bioeng. 1999;66: 86–103. doi:10.1002/(SICI)1097-0290(1999)66:2<86::AID-BIT2>3.0.CO;2-A
52. Toya Y, Ishii N, Nakahigashi K, Hirasawa T, Soga T, Tomita M, et al. ^{13}C-metabolic flux analysis for batch culture of *Escherichia coli* and its pyk and pgi gene knockout mutants based on mass isotopomer distribution of intracellular metabolites. Biotechnol Prog. 2010;26: 975–992. doi:10.1002/btpr.420
53. Marx A, Striegel K, de Graaf AA, Sahm H, Eggeling L. Response of the central metabolism of *Corynebacterium glutamicum* to different flux burdens. Biotechnol Bioeng. 1997;56: 168–180. doi:10.1002/(SICI)1097-0290(19971020)56:2<168::AID-BIT6>3.0.CO;2-N
