# Supplementary material for: A Pareto approach to resolve the conflict between information gain and experimental costs: Multiple-criteria design of carbon labeling experiments
Source: PLoS Comput Biol. 2018 Oct 31;14(10):e1006533. doi: 10.1371/journal.pcbi.1006533 (PMC6209137; doi:10.1371/journal.pcbi.1006533)
Supplement: S2 Text — Mathematical formulation of the multi-objective optimization task for 13C MFA, details about multi-objective optimizers, documentation of the cost factors and the experimental scenario. (PDF) [file pcbi.1006533.s002.pdf]

Supplementary Appendix 2 for

A Pareto approach to resolve the conflict between information gain and experimental costs: Multiple-criteria design of carbon labeling experiments

Multi-objective ^{13}C MFA: Formulation and setup

Katharina Nöh, Sebastian Niedenführ, Martin Beyß, Wolfgang Wiechert

k.noeh@fz-juelich.de

Contents

1. Mathematical formulation of the MO-ED problem 2
2. Empirical computational performance assessment 8
   2.1 Solution quality and speed assessment of NSGA-II, SPEA 2, and SMPSO for MO-ED ^{13}C-MFA 8
3. Experimental scenario 10
4. Cost factors 11
5. References 12

1. Mathematical formulation of the MO-ED problem

In multi-objective ED the task is to find design variables $\boldsymbol{\alpha}$ that simultaneously optimize more than a single objective

$$\max_{\boldsymbol{\alpha}\in\boldsymbol{\Omega}} \; \boldsymbol{\Phi}\left(\boldsymbol{\alpha},\boldsymbol{\theta}\right)$$

subject to

$$\begin{aligned}\mathbf{g}\left(\boldsymbol{\alpha},\boldsymbol{\theta}\right) &\geq \mathbf{0}\\ \mathbf{h}\left(\boldsymbol{\alpha},\boldsymbol{\theta}\right) &= \mathbf{0}\\ \mathbf{l} \leq \boldsymbol{\alpha} &\leq \mathbf{u}\end{aligned}$$

where $\boldsymbol{\Phi}$ is the vector of objective functions to be minimized, $\boldsymbol{\alpha}$ the vector of design variables located in the design space $\boldsymbol{\Omega}$ of the MO-ED problem, and $\boldsymbol{\theta}$ is a vector of further parameters that are chosen with respect to the application scenario and which not change during the study. The functions $\mathbf{g}$ and $\mathbf{h}$ determine inequality and equality constraints, respectively that contain additional requirements for the design parameters to be fulfilled. Solving the MO-ED problem means to approximate the (possibly uncountable) set of best design parameters $\boldsymbol{\alpha}^*$ that then give the Pareto-front $\boldsymbol{\Phi}^*$. For the approximation of the Pareto front, an evolutionary MOO algorithm is used (cf. S2 Sec 2 for further details).

Two scenarios are investigated in the main text, as represented by two different realizations of the objective vector $\boldsymbol{\Phi}$:

3D-MO-ED: $$\boldsymbol{\Phi}\left(\boldsymbol{\alpha},\boldsymbol{\theta}\right)=\begin{pmatrix}\varphi_{D,p}\left(\boldsymbol{\alpha},\boldsymbol{\theta}\right)\\ \varphi_{DoF}\left(\boldsymbol{\alpha},\boldsymbol{\theta}\right)\\ -\varphi_{Costs}\left(\boldsymbol{\alpha},\boldsymbol{\theta}\right)\end{pmatrix}$$

5D-MO-ED: $$\boldsymbol{\Phi}\left(\boldsymbol{\alpha},\boldsymbol{\theta}\right)=\begin{pmatrix}\varphi_{D,p}\left(\boldsymbol{\alpha},\boldsymbol{\theta}\right)\\ \varphi_{A,p}\left(\boldsymbol{\alpha},\boldsymbol{\theta}\right)\\ \varphi_{E}\left(\boldsymbol{\alpha},\boldsymbol{\theta}\right)\\ \varphi_{DoF}\left(\boldsymbol{\alpha},\boldsymbol{\theta}\right)\\ -\varphi_{Costs}\left(\boldsymbol{\alpha},\boldsymbol{\theta}\right)\end{pmatrix}$$

with the objective functions:

- $\boldsymbol{\Phi}_{D,p}$: D-criterion

$$\boldsymbol{\Phi}_{D,p}\left(\boldsymbol{\alpha},\boldsymbol{\theta}\right)=\sqrt[2\cdot p]{\det\left(\mathbf{Cov}\left(\left(\underbrace{\mathbf{w}_{inp},\mathbf{w}_{meas}^{dev}}_{\boldsymbol{\alpha}},\underbrace{\hat{\mathbf{v}}^{free},b_1^{dev},b_2^{dev},\ldots}_{\boldsymbol{\theta}}\right)\right)\right)}$$

with $p=\dim\left(\mathbf{Cov}\right)$

- $\mathbf{\Phi}_{A,p}$: A-criterion

$$\mathbf{\Phi}_{A,p}\left(\boldsymbol{\alpha},\boldsymbol{\theta}\right) = \operatorname{trace}\left(\mathbf{Cov}\left(\underbrace{\mathbf{w}_{inp},\mathbf{w}_{meas}^{dev}}_{\boldsymbol{\alpha}},\boldsymbol{\theta}\right)\right)\Big/ p$$

with $p = \dim\left(\mathbf{Cov}\right)$

- $\mathbf{\Phi}_{E}$: E-criterion

$$\mathbf{\Phi}_{E}\left(\boldsymbol{\alpha},\boldsymbol{\theta}\right) = \frac{\boldsymbol{\lambda}_{\max}\left(\mathbf{Cov}\left(\mathbf{w}_{inp},\mathbf{w}_{meas}^{dev},\boldsymbol{\theta}\right)\right)}{\boldsymbol{\lambda}_{\min}\left(\mathbf{Cov}\left(\underbrace{\mathbf{w}_{inp},\mathbf{w}_{meas}^{dev}}_{\boldsymbol{\alpha}},\boldsymbol{\theta}\right)\right)}$$

with $\lambda_{\min},\lambda_{\max}$ the smallest and largest eigenvalues of $\mathbf{Cov}$

- $\mathbf{\Phi}_{DoF}$: DoF-criterion

$$\mathbf{\Phi}_{DoF}\left(\boldsymbol{\alpha},\boldsymbol{\theta}\right) = \dim\left(\mathbf{Cov}\left(\underbrace{\mathbf{w}_{inp},\mathbf{w}_{meas}^{dev}}_{\boldsymbol{\alpha}},\boldsymbol{\theta}\right)\right)$$

- $\mathbf{\Phi}_{Costs}$: Total cost functional

$$\mathbf{\Phi}_{Costs}\left(\boldsymbol{\alpha},\boldsymbol{\theta}\right) = \sum_{cle=1}^{n_{cle}}\mathbf{\Phi}_{Costs,cle}\left(\boldsymbol{\alpha}_{cle},\boldsymbol{\theta}_{cle}\right)$$

- $\mathbf{\Phi}_{Costs,cle}$: Cost functional of a single CLE

$$\begin{aligned}\mathbf{\Phi}_{Costs,cle}\left(\boldsymbol{\alpha}_{cle},\boldsymbol{\theta}_{cle}\right) = {} & \underbrace{\mathbf{w}_{inp}^{T}\cdot\mathbf{C}_{inp}}_{tracer} + \underbrace{C_{exp} + t_{work,exp}\cdot C_{work}}_{experimental\ setup} \\ & + \underbrace{n_{samples}\cdot C_{sample}}_{aquisition} + \underbrace{\left(\sum_{i=1}^{n_{mgroups}} n_{rep,mgroup_i}^{dev}\cdot n_{mgroup,peaks}^{dev}\right)\cdot t_{work,ana}\cdot C_{work}}_{peak\ evaluation}\end{aligned}$$

For shortness of notation, we do not explicitly indicate the association of the design parameters and constants to the *cle*th experiment.

Design variables α:

- $\mathbf{w}_{inp}$: Design vector of tracer composition fractions

$$\dim\left(\mathbf{w}_{inp}\right) = \dim\left(\mathbf{x}_{inp,all}\right)$$

- $\mathbf{n}_{meas}^{dev}$: Design vector for measurement group selection

$$\dim\left(\mathbf{n}_{meas}^{dev}\right) = n_{mgroups}$$

For each measurement group in $M_{meas,all}^{dev}$, the corresponding entry indicates whether the group is not selected (0) or selected (>1) with a certain number of repeats: $n_{meas,i}^{dev} \in \left\{0,2,\ldots,n_{samples,max}\right\},\ i = 1,\ldots,n_{mgroups}$.

To calculate standard deviations, at least two replicates have to be analyzed.

To circumvent the solution of a mixed-integer nonlinear programming problem, the formulation is relaxed, such that instead of integers in the optimization process weights are allowed to take non-integer values: $w_{meas,i}^{dev} \in \left[0, n_{samples,max}\right], i = 1, \ldots, n_{mgroups}$ and the results are finally rounded to integers.

$$n_{meas,i}^{dev} = \begin{cases} 0, & w_{meas,i}^{dev} < 1.5 \\ \text{round}\left(w_{meas,i}^{dev}\right), & \text{else} \end{cases}, \; i = 1, \ldots, n_{mgroups}$$

Nonlinear inequality constraints:

- $\boldsymbol{\lambda}_{\min}\left(\mathbf{FIM}\right) > 10^{-3}$ with $\lambda_{\min}$ being the smallest eigenvalue of the Fisher information matrix $\mathbf{FIM}$
- $\text{cond}\left(\mathbf{FIM}\right) < 5 \cdot 10^{5}$ with cond denoting the condition number of the Fisher information matrix $\mathbf{FIM}$

Linear equality constraints $\boldsymbol{h}\left(\boldsymbol{\alpha}, \boldsymbol{\theta}\right)$:

- $\sum_i w_{inp,i} = 1.0$

Boxed constraints $\boldsymbol{l}, \boldsymbol{u}$:

- $0 \leq w_{inp,i} \leq 1, i = 1, \ldots, \dim\left(\mathbf{x}_{\boldsymbol{inp,all}}\right)$
- $0 \leq w_{meas,i}^{dev} \leq n_{samples,max}, \; i = 1, \ldots, n_{mgroups}$

Inputs/design constants:

To pose the MO-ED task, the following quantities have to be specified:

- $\hat{\mathbf{v}}^{free}$: Free flux vector at the design point
- n_{cle}: Number of cultivations
- $\mathbf{x}_{inp,all}$: Tracer configuration vector for each CLE
- b_1^{dev}, b_2^{dev}: Coefficients of the linear measurement error models; pairs of regression coefficients are calibrated for the analytical platform
- $n_{samples,max}$: Maximal number of samples that can be withdrawn from the cultivation for each CLE

- $\mathbf{M}_{meas,all}^{dev}$: Measurement configuration matrix translating the labeling state vector into the measured signals for each CLE; the configuration consists $n_{mgroups}^{dev}$ measurement groups; each measurement group consists of a set of $n_{mgroup,peaks}^{dev}$ measurements;

  $$\dim\left(\mathbf{M}_{meas,all}^{dev}\right) = n_{mgroups}^{dev} \cdot n_{mgroup,peaks}^{dev} \times \dim\left(\mathbf{x}\right)$$

- $\mathbf{C}_{inp}$: Tracer cost configuration vector

  $$\dim\left(\mathbf{C}_{inp}\right) = \dim\left(\mathbf{x}_{inp,all}\right)$$

- C_{exp}: Experimental costs (consumables, etc.)
- C_{sample}: Acquisition costs per sample
- C_{work}: Operators wage for work per hour
- $\mathrm{t}_{work,exp}$: Time for the experiment
- $\mathrm{t}_{work,ana}$: Time for the analytics

Further notation:

- $\mathbf{x}^{inp}$: Input substrate mixture of a design

  $$\mathbf{x}^{inp} = \mathbf{w}_{inp}^{T} \cdot \mathbf{x}_{inp,all}$$

- $\mathbf{x}$: Labeling state vector $\mathbf{x} = \mathbf{x}\left(\mathbf{v}^{free}, \mathbf{x}^{inp}\right)$, uniquely derived from the solution of the labeling mass balances $\mathbf{f}\left(\mathbf{x}, \mathbf{x}^{inp}, \mathbf{v}^{free}\right) = \mathbf{0}$
- $\mathbf{M}_{meas}^{dev}$: Measurement matrix containing measurement groups of a design
- $n_{mgroups}^{dev}$: Number of measurement groups contained in $\mathbf{M}_{meas}^{dev}$
- $n_{mgroup,peaks}^{dev}$: Number of peaks contained in the measurement group $mgroup$
- $n_{rep,mgroup}^{dev}$: Number of replicates of a measurement group $mgroup$

  $$n_{rep,mgroup}^{dev} \leq n_{samples,max}, \quad i = 1, \ldots, n_{mgroups}^{dev}$$

- $n_{samples}$: Number of samples that are withdrawn from the bioreactor

  $$n_{samples} = \max_{\forall i} \; n_{rep,mgroup_i}^{dev}$$

Measurement (error) model for technical replicates:

- $\boldsymbol{\sigma}_{meas}^{dev}$: Model-based standard deviation approximation for labeling data

  To establish a conservative dependency between the error and the number of repeated measurements, instead of the value provided by the error models in S1 Fig B, the measurement error of a single

measurement is associated with the upper bound of the confidence interval of the standard deviations [1]:

$$\boldsymbol{\sigma}_{meas}^{dev}\left(\boldsymbol{\alpha},\boldsymbol{\theta}\right)=\sqrt{\frac{\left(\mathbf{n}_{rep,meas}^{dev}-\mathbf{1}\right)}{\chi^2\left(1-\beta/2,\mathbf{n}_{rep,meas}^{dev}-\mathbf{1}\right)}}\cdot\left(b_1^{dev}\cdot\mathbf{M}_{meas}^{dev}\cdot\mathbf{x}\left(\boldsymbol{\alpha},\boldsymbol{\theta}\right)+b_2^{dev}\right)$$

(evaluated component-wise) for a given significance level β.

$$\dim\left(\boldsymbol{\sigma}_{meas}^{dev}\right)=\sum_{i=1}^{n_{mgroups}} n_{mgroup_i,peaks}^{dev}$$

- $\boldsymbol{\Sigma}_{meas}^{dev}$: Measurement covariance matrix

$$\boldsymbol{\Sigma}_{meas}^{dev}\left(\boldsymbol{\alpha},\boldsymbol{\theta}\right)=\mathrm{diag}\left(\boldsymbol{\sigma}_{meas}^{dev}\left(\boldsymbol{\alpha},\boldsymbol{\theta}\right)\right)$$

with

$$\dim\left(\boldsymbol{\Sigma}_{meas}^{dev}\right)=\sum_{i=1}^{n_{mgroups}} n_{mgroup_i,peaks}^{dev}\times\sum_{i=1}^{n_{mgroups}} n_{mgroup_i,peaks}^{dev}$$

Information matrices:

- $\mathbf{FIM}$: Fisher information matrix

$$\mathbf{FIM}\left(\boldsymbol{\alpha},\boldsymbol{\theta}\right)=\left(\left.\frac{\partial\ \mathbf{M}_{meas}^{dev}\cdot\mathbf{x}\left(\mathbf{v}^{free},\mathbf{x}^{inp}\right)}{\partial\,\mathbf{v}^{free}}\right|_{\hat{\mathbf{v}}^{free}}\right)^T\cdot\ \boldsymbol{\Sigma}_{meas}^{dev}\ \cdot\ \left.\frac{\partial\ \mathbf{M}_{meas}^{dev}\cdot\mathbf{x}\left(\mathbf{v}^{free},\mathbf{x}^{inp}\right)}{\partial\,\mathbf{v}^{free}}\right|_{\hat{\mathbf{v}}^{free}}$$

- $\mathbf{Cov}$: Covariance matrix

$$\mathbf{Cov}\left(\boldsymbol{\alpha},\boldsymbol{\theta}\right)=\mathbf{FIM}^{-1}\left(\boldsymbol{\alpha},\boldsymbol{\theta}\right)$$

Calculation of free fluxes:

- $\mathbf{S}$, $\mathbf{b}$: Stoichiometric network model and extracellular rate vector such that $\mathbf{S}\cdot\mathbf{v}=\mathbf{b}$ with $\mathbf{v}$ the vector of all intracellular fluxes
- $\mathbf{C}_{ineq}$, $\mathbf{c}_{ineq}$: Inequality constraints such that $\mathbf{C}_{ineq}\cdot\mathbf{v}\leq\mathbf{c}_{ineq}$
- $\mathbf{v}$: Flux vector, fulfills mass balances induced by a metabolic network model $\mathbf{S}\cdot\mathbf{v}=\mathbf{b}$ and inequality constraints $\mathbf{C}_{ineq}\cdot\mathbf{v}\leq\mathbf{c}_{ineq}$
- $\mathbf{v}^{free}$: Free flux vector derived according to $\mathbf{v}=\mathbf{K}\cdot\begin{pmatrix}\mathbf{1}\\ \mathbf{v}^{free}\end{pmatrix}$ with $\mathbf{K}$ denoting the kernel (nullspace) matrix of $\mathbf{S}$.
Then $\mathbf{v}^{free}$ is subject to inequality constraints

$\mathbf{C}_{ineq}^{free} \cdot \mathbf{v}^{free} \leq \mathbf{c}_{ineq} - \mathbf{c}^{free}$ with $\left(\mathbf{c}^{free} \quad \mathbf{C}_{ineq}^{free}\right) \stackrel{!}{=} \mathbf{C}_{ineq} \cdot \mathbf{K}$ such that the dimension of $\mathbf{C}_{ineq}^{free}$ matches that of $\mathbf{v}^{free}$.

2. Empirical computational performance assessment

The MO-ED formulation arising from ^{13}C MFA applications is non-linear, high-dimensional and constrained. This makes an application of exact techniques infeasible. Instead, the aim is to approximate the true Pareto front as good as possible. Excellent comparative overviews of numerical state-of-the art algorithms like Speed-constrained Multi-objective Particle Swarm Optimization (SMPSO [2]), Strength Pareto Evolutionary Algorithm 2 (SPEA-2) [3] and Non-dominated Sorting Genetic Algorithm-II (NSGAII) [4] are found in the recent literature [5,6]. Evolutionary algorithms and Particle Swarm Optimization (PSO) are examples for approximate meta-heuristics that have been successfully applied to solve MO problems [2,7].

jMetal [8] is a powerful, open source optimization framework implemented in Java that contains the realizations of several advanced meta-heuristics to solve MO problems. Implemented algorithms include for instance, the genetic algorithms NSGA-II (Non-dominated Sorting Genetic Algorithm-II [4]), and SPEA 2 (Strength Pareto Evolutionary Algorithm [3]), and the PSO algorithm SMPSO (Speed constrained Multi-objective PSO [2]). NSGA-II and SPEA 2 are arguably the most often applied multi-objective optimization techniques. The implementation of jMetal ver.1 was used. The calculations were run on a Linux work station with Intel® Xeon® CPU (2.93 GHz) with 16 processors (4 MB cache each, multi-threading enabled) and 132 GB main memory.

2.1 Solution quality and speed assessment of NSGA-II, SPEA 2, and SMPSO for MO-ED ^{13}C-MFA

Performance comparisons report that the performance of MOO methods is heavily influenced by the parameters of the multi-heuristics, such as population/particle size, selection operators, and termination criteria and is specific to the problem under investigation, in particular convexity, discreteness, and multimodality characteristics [6,9]. Therefore, simulation experiments were run for each algorithm to test the used set of MOO parameters. The population (NSGA-II, SPEA 2) and swarm size (SMPSO), respectively, was set to 1,000. The size of the archive was also 1,000 for all algorithms. Simulated binary crossover and polynomial-based mutation (for 15% of the swarm individuals in case of SMPSO) was used. As stopping criterion for the algorithms, a threshold for the maximum number of generations was set (25,000). All remaining parameters were selected as originally proposed in Durillo et al. [9]. These values are approved in several simulation experiments with the *P. chrysogenum* network model (cf. S3 Appendix). No explicit effort was made to further tune the algorithm's parameters for the different MO-ED formulations.

Algorithm's performances were benchmarked with respect to solution quality and speed. Solution quality is determined by the diversity or spread of the best-known Pareto optimal set [9]. In this study, the volume of the objective space dominated by a Pareto front approximation, the so called *hypervolume* quality indicator [10] was used which measures not only the diversity but also the convergence of the solution. Because the true Pareto set is unknown in our case, convergence speed was measured as in Nebro et al. [11]: the algorithms were run with different maximal number of function evaluations. We found that a maximal number of 10^6 evaluations was sufficient for convergence as indicated by a constant hypervolume (hv_{opt}) for iterations > 10^6. For each algorithm the number of iterations was recorded that was

needed to find an approximation of the best-known Pareto set with a hypervolume of 98% of best known value hv_{opt}. If the algorithm was unable to meet this criterion, a failure was recorded for the run.

The number of iterations was measured in several independent runs as follows:

- SMPSO: $4.4 \pm 2.8 \cdot 10^4$ iterations
- NSGA-II: $1.7 \pm 0.9 \cdot 10^5$ iterations

SPEA 2 was often unable to find acceptable, i.e., well-spread solutions. In particular, in our test-runs with this algorithm many times a strong decrease in hypervolume was diagnosed after reaching an optimum.

Summarizing, SMPSO was ~300% faster than NSGA-II. Based on these tests, we decided to use the SMPSO algorithm for our study.

For generating MO-ED results, calculations were performed at least in duplicate and results were pooled to guarantee that the Pareto front approximations contain sufficiently many solutions.

3. Experimental scenario

The to be planned labeling experiment is conducted in a 250 mL bioreactor setting operated in continuous mode (i.e., chemostat) without replication (i.e., $n_{cle} = 1$). We assumed a total of 6 bioreactor volume changes, resulting in a requirement of 1.5 L medium. For the cultivation media with 20 g/L substrate was assumed. Substrate costs are given in S2 Table A. For preparation and observation of the experiment 39 h were assumed. The compensation was set to 20 €/h. A maximal number of 10 samples (i.e., technical replicates) could be withdrawn per experiment. The cost for the bio-analytics in terms of purchase investment, maintenance and related measurement time varies depending on the device used (cf. S2 Table B). It was assumed that each device is in usage for five years operated at full capacity. The resulting costs for acquisition are related to the device usage time. A measurement spectrum is checked, transferred to the model in 0.0055 h (20 s) based on our on-average experiences for mass spectroscopy.

4. Cost factors

Table A. Substrate costs for MO-ED ^{13}C-MFA. Substrates were assumed to have a purity of 99% with exception of unlabeled glucose which possesses a purity of 98%. Prices were collected from Cambridge Isotope Laboratories, Inc. (www.isotope.com).

Substrates	Specification	Price [€/g]
[^{12}C]-glucose	GLC#000000	0.30
[1-^{13}C]-glucose	GLC#100000	147.00
[2-^{13}C]-glucose	GLC#020000	472.00
[3-^{13}C]-glucose	GLC#003000	912.00
[4-^{13}C]-glucose	GLC#000400	1,218.00
[5-^{13}C]-glucose	GLC#000050	1,293.00
[6-^{13}C]-glucose	GLC#000006	532.00
[1,2-^{13}C]-glucose	GLC#110000	494.00
[1,6-^{13}C]-glucose	GLC#100001	1141.00
[U-^{13}C]-glucose	GLC#111111	134.00

Remark: ^{13}C-labeled substrates purchasable from manufacturers are always affected by isotopic impurities that alter the actual labeling pattern of the substrate and therewith also the emerging intracellular labeling patterns. Manufacturers' purity factors are usually specified in atom-%. For example, 99 atom-% pure [U-^{13}C]-glucose means that each carbon position is 99% ^{13}C-labeled resulting in an abundance of 0.94 for the molecule. The bias in labeled substrates needs to be taken into account because it impacts not only the labeling states but also the statistical flux identifiability. Prices were received in 2014 and not corrected for price movements or currency changes.

Table B. Sales prices for bioanalytical devices and measurement time per replicate used for MO-ED ^{13}C-MFA. Prices were received by manufacturer information and by interviewing scientists from analytical labs for their experiences. Prices are inquired in 2014 and not corrected for price movements or currency trends.

Analytical platform	Price [€]	Measurement time [h]
GC-MS	100,000	0.66
LC-MS	180,000	0.66
LC-MS/MS	350,000	0.66
^{13}C-NMR	1,000,000	6.00

5. References

1. Lee I, Choi KK, Noh Y. Comparison study between probabilistic and possibilistic approach for problems with correlated input and lack of input statistical information. Volume 5: 35th Design Automation Conference, Parts A and B. ASME; 2009. pp. 1137–1148. doi:10.1115/DETC2009-86703
2. Nebro AJ, Durillo JJ, Garcia-Nieto J, Coello Coello CA, Luna F, Alba E. SMPSO: A new PSO-based metaheuristic for multi-objective optimization. 2009 IEEE Symposium on Computational Intelligence in Multi-Criteria Decision-Making. 2009. pp. 66–73. doi:10.1109/MCDM.2009.4938830
3. Zitzler E, Laumanns M, Thiele L. SPEA2: Improving the strength pareto evolutionary algorithm. Evolutionary Methods for Design Optimization and Control with Applications to Industrial Problems. Technical Report ETH Zurich, 2001.
4. Deb K, Member A, Pratap A, Agarwal S, Meyarivan T. A fast and elitist multiobjective genetic algorithm: NSGA-II. IEEE Trans Evol Comput. 2002;6: 182–197.
5. Konak A, Coit DW, Smith AE. Multi-objective optimization using genetic algorithms: A tutorial. Reliab Eng Syst Saf. 2006;91: 992–1007. doi:10.1016/j.ress.2005.11.018
6. Zitzler E, Deb K, Thiele L. Comparison of multiobjective evolutionary algorithms: Empirical results. Evol Comput. 2000;8: 173–195. doi:10.1162/106365600568202
7. Zitzler E, Laumanns M, Bleuler S. A tutorial on evolutionary multiobjective optimization. Evol Comput. 2004;535: 3–37. doi:10.1007/978-3-642-17144-4_1
8. Durillo JJ, Nebro AJ. jMetal: A Java framework for multi-objective optimization. Adv Eng Softw. 2011;42: 760–771. doi:10.1016/j.advengsoft.2011.05.014
9. Durillo JJ, Nebro AJ, Luna F, Coello Coello CA, Alba E. Convergence speed in multi-objective metaheuristics: efficiency criteria and empirical study. Int J Numer Methods Eng. 2010;84: 1344–1375. doi:10.1002/nme
10. Zitzler E, Thiele L. Multiobjective evolutionary algorithms: A comparative case study and the strength Pareto approach. Evol Comput IEEE Trans. 1999;3: 257–271. doi:10.1109/4235.797969
11. Nebro AJ, Durillo JJ, Coello Coello CA, Luna F, Alba E. Design issues in a study of convergence speed in multi-objective metaheuristics. In: Rudolph G, Al E, editors. Parallel Problem Solving from Nature - PPSN X. Springer; 2008. pp. 763–772.
