# Supplementary material for: A Pareto approach to resolve the conflict between information gain and experimental costs: Multiple-criteria design of carbon labeling experiments
Source: PLoS Comput Biol. 2018 Oct 31;14(10):e1006533. doi: 10.1371/journal.pcbi.1006533 (PMC6209137; doi:10.1371/journal.pcbi.1006533)
Supplement: S3 Text — Description of the network model for P. chrysogenum including carbon atom transitions, constraints, and flux distribution. (PDF) [file pcbi.1006533.s003.pdf]

Supplementary Appendix 3 for

A Pareto approach to resolve the conflict between information gain and experimental costs: Multiple-criteria design of carbon labeling experiments

^{13}C MFA network model of *P. chrysogenum*

Katharina Nöh, Sebastian Niedenführ, Martin Beyß, Wolfgang Wiechert

k.noeh@fz-juelich.de

Contents

1. Network Model 2

1. Network Model

Table A. Metabolic network model and carbon atom mappings of *P. chrysogenum* used for the multi-objective experimental design study. Reaction arrows indicate uni- and bidirectional reactions, respectively. For effluxes extracellular pools are omitted. Reactions with empty brackets "()" provide stoichiometric relations only. Association of metabolites with compartments other than the cytosol is indicated by brackets "[]" (ex: extracellular, mit: mitochondrial).

Reaction	Stoichiometry and carbon atom mappings
gly1:	GLC[ex](#ABCDEF) → G6P(#ABCDEF)
gly2:	G6P(#ABCDEF) ⇌ F6P(#AFBCDE)
gly3:	F6P(#ABCDEF) → FBP(#ABCDEF)
gly4:	FBP(#ABCDEF) ⇌ DHAP(#EBF) + GAP(#DAC)
gly4b:	DHAP(#ABC) ⇌ GAP(#ABC)
gly5:	GAP(#ABC) ⇌ 23PG(#BCA)
gly6:	23PG(#ABC) ⇌ PEP(#ABC)
gly7:	PEP(#ABC) → PYR(#ABC)
Ppp1:	G6P(#ABCDEF) → 6PG(#DACEBF)
Ppp2:	6PG(#ABCDEF) → Ru5P(#DBAEC) + CO2(#F)
Ppp3:	Ru5P(#ABCDE) ⇌ X5P(#ABCDE)
Ppp4:	Ru5P(#ABCDE) ⇌ R5P(#BDECA)
Ppp5:	X5P(#ABCDE) + R5P(#FGHIJ) ⇌ GAP(#EBD) + S7P(#AFCGJHI)
Ppp6:	GAP(#ABC) + S7P(#DEFGHIJ) ⇌ F6P(#BDCAHF) + E4P(#JEIG)
Ppp7:	E4P(#ABCD) + X5P(#EFGHI) ⇌ F6P(#BEDCAG) + GAP(#IFH)
Help_Ru5P:	Ru5P(#ABCDE) → Ru5P_X5P(#ABCDE)
Help_X5P:	X5P(#ABCDE) → Ru5P_X5P(#ABCDE)
Help_Ru5P_X5P:	Ru5P_X5P(#ABCDE) →
Tca1:	PYR[mit](#ABC) → ACCOA[mit](#AB) + CO2[mit](#C)
Tca2:	OAA[mit](#ABCD) + ACCOA[mit](#EF) → CIT[mit](#AECFDB)
Tca3:	CIT[mit](#ABCDEF) → ACO[mit](#ABFCDE)
Tca4:	ACO[mit](#ABCDEF) → AKG[mit](#CBAED) + CO2[mit](#F)
Tca5a:	AKG[mit](#ABCDE) ⇌ SUC[mit](#BADC) + CO2[mit](#E)
Tca5b:	AKG[mit](#ABCDE) ⇌ SUC[mit](#ABCD) + CO2[mit](#E)
Tca6a:	SUC[mit](#ABCD) ⇌ FUM[mit](#ABCD)
Tca6b:	SUC[mit](#ABCD) ⇌ FUM[mit](#BADC)
Tca7a:	FUM[mit](#ABCD) ⇌ MAL[mit](#BADC)
Tca7b:	FUM[mit](#ABCD) ⇌ MAL[mit](#ABCD)
Tca8:	MAL[mit](#ABCD) ⇌ OAA[mit](#ABCD)
Ana1:	OAA(#ABCD) ⇌ PEP(#ABD) + CO2(#C)
Ana2:	PYR(#ABC) + CO2(#D) ⇌ OAA(#ABDC)
Ana3:	PYR[mit](#ABC) + CO2[mit](#D) ⇌ MAL[mit](#ABDC)
Ac:	PYR(#ABC) → CO2(#C) + ACCOA(#AB)

Reaction	Stoichiometry and carbon atom mappings
Ser1:	23PG(#ABC) → PHP(#ABC)
Ser2:	PHP(#ABC) → PSer(#ABC)
Ser3:	PSer(#ABC) → SER(#ABC)
Ser4:	SER(#ABC) + ACCOA(#DE) → OACSER(#DAEBC)
Gly1:	SER(#ABC) ⇌ EC1(#A) + GLY(#BC)
Gly2:	EC1(#A) + CO2(#B) ⇌ GLY(#AB)
Cys1:	OACSER(#ABCDE) → CYS(#BDE) + ACCOA(#AC)
Cys2:	CYS(#ABC) + CYS(#DEF) → CYST(#DAEBFC)
His:	R5P(#ABCDE) + EC1(#F) → HIS(#CEFDBA)
Chor:	E4P(#ABCD) + PEP(#EFG) + PEP(#HIJ) → CHOR(#EBDHFICAGJ)
Phe:	CHOR(#ABCDEFGHIJ) → PHE(#GCHBDAFEI) + CO2(#J)
Tyr:	CHOR(#ABCDEFGHIJ) → TYR(#DBHCAFGEI) + CO2(#J)
Met:	EC1(#A) + ASP(#BCDE) → MET(#ABDCE)
Trp1:	CHOR(#ABCDEFGHIJ) → PYR(#AEI) + ANT(#CGBHFDJ)
Trp2:	ANT(#ABCDEFG) + R5P(#HIJKL) → ANT2(#ABCDLHEFKIJG)
Trp3:	ANT2(#ABCDEFGHIJKL) → CO2(#L) + ANT3(#ABCDEFGIHJK)
Trp4:	ANT3(#ABCDEFGHIJK) → TRP(#ABCDKEHGJIF)
Ala:	PYR[mit](#ABC) → ALA[mit](#ABC)
Val1:	PYR[mit](#ABC) + PYR[mit](#DEF) → CO2[mit](#C) + DHIV[mit](#ADEFB)
Val2:	DHIV[mit](#ABCDE) → KIV(#ABECD)
Val3:	KIV(#ABCDE) → VAL(#ABCDE)
Leu:	PYR[mit](#ABC) + PYR[mit](#DEF) + ACCOA(#GH) → LEU(#DABEGH) + CO2(#F) + CO2(#C)
Ileu1:	PYR[mit](#ABC) + ASP(#DEFG) → CO2(#C) + KILE(#FADBEG)
Ileu2:	KILE(#ABCDEF) → ILE(#ABCDEF)
Arg:	AKG[mit](#ABCDE) + EC1(#F) → ARG(#BADCEF)
Glu1:	AKG[mit](#ABCDE) → GL[mit](#ABCDE)
Glu2:	GLU[mit](#ABCDE) → PRO[mit](#BADCE)
Glu3:	GLU[mit](#ABCDE) → GLN[mit](#ABCDE)
Glu4:	GLU[mit](#ABCDE) → ORN[mit](#BADCE)
Lys1:	AKG[mit](#ABCDE) + ACCOA(#FG) → CO2(#E) + AAA(#BDAFCG)
Lys2:	AAA(#ABCDEF) → LYS(#ACBEDF)
Asp1:	OAA(#ABCD) → ASP(#ABCD)
Asp1b:	OAA[mit](#ABCD) → ASP(#ABCD)
Asp2:	ASP(#ABCD) → ASN(#ABCD)
Thr1:	ASP(#ABCD) → HSER(#ACBD)
Thr2:	ACCOA(#AB) + GLY(#CD) ⇌ THR(#ABCD)
Thr3:	HSER(#ABCD) → THR(#BACD)
Poa_IN:	POA_IN() → POA()
Pen1:	POA() + IPN(#ABCDEF) → PENV() + AAA(#ABCDEF)

Reaction	Stoichiometry and carbon atom mappings
Pen2:	CV() + AAA(#ABCDEF) → IPN(#ABCDEF)
Pen3:	PENV() → BYPROD()
Pen_Syn_VAL:	VAL(#ABCDE) →
Pen_Syn_CYS:	CYS(#ABC) →
Transporter_PYR:	PYR(#ABC) → PYR[mit](#ABC)
Transporter_OAA:	OAA(#ABCD) ⇌ OAA[mit](#ABCD)
Transporter_ACCOA:	ACCOA(#AB) ⇌ ACCOA[mit](#AB)
Transporter CO2:	CO2(#A) → CO2[mit](#A)
BM_AAA:	AAA(#ABCDEF) →
BM_ACCOA:	ACCOA(#AB) →
BM_ALA:	ALA[mit](#ABC) →
BM_ARAB:	Ru5P(#ABCDE) →
BM_ARG:	ARG(#ABCDEF) →
BM_ASN:	ASN(#ABCD) →
BM_ASP:	ASP(#ABCD) →
BM_CIT:	CIT(#ABCDEF) →
BM_CO2:	CO2(#A) →
BM_CYS:	CYS(#ABC) →
BM _CYST:	CYST(#ABCDEF) →
BM_E4P:	E4P(#ABCD) →
BM_EC1:	EC1(#A) →
BM_F6P:	F6P(#ABCDEF) →
BM_FUM:	FUM[mit](#ABCD) →
BM_GAL:	G6P(#ABCDEF) →
BM_GAP:	GAP(#ABC) →
BM_GLN:	GLN[mit](#ABCDE) →
BM_GLU:	GLU[mit](#ABCDE) →
BM_GLUC:	G6P(#ABCDEF) →
BM_GLY:	GLY(#AB) →
BM_HIS:	HIS(#ABCDEF) →
BM_AKG:	AKG[mit](#ABCDE) →
BM_ILE:	ILE(#ABCDEF) →
BM_LEU:	LEU(#ABCDEF) →
BM_LYS:	LYS(#ABCDEF) →
BM_MAN:	F6P(#ABCDEF) →
BM_MANNOSE:	F6P(#ABCDEF) →
BM_MET:	MET(#ABCDE) →
BM_ORN:	ORN[mit] (#ABCDE) →
BM_PHE:	PHE(#ABCDEFGHI) →
BM_PRO:	PRO[mit] (#ABCDE) →
BM_R5P:	R5P(#ABCDE) →

Reaction	Stoichiometry and carbon atom mappings
BM_SER:	SER(#ABC) $\rightarrow$
BM_THR:	THR(#ABCD) $\rightarrow$
BM_TRP:	TRP(#ABCDEFGHIJK) $\rightarrow$
BM_TYR:	TYR(#ABCDEFGHI) $\rightarrow$
BM_VAL:	VAL(#ABCDE) $\rightarrow$
CO2_EX:	CO2(#A) $\rightarrow$
bisACV_IPN_EX:I:	PN(#ABCDEF) $\rightarrow$
PEN_EX:	PENV() $\rightarrow$
BYPROD_EX:	BYPROD() $\rightarrow$
BIOM_EX:	BIOMass() $\rightarrow$
BIOM_IN:	0.0299*AAA+0.8743*ACCOA+0.3005*ALA+0.0095*ARAB+ 0.2130*ARG+0.1958*ASN+0.2837*ASP+0.0011*CIT+ 0.1550*CO2+0.0039*Cyst+0.0157*E4P+0.1344*EC1+0.0711*F6P+ 0.0007*FUM+0.2966*GAL+0.1427*GAP+0.2819*GLN+0.0950*GLUC +0.5656*GLU+0.4224*GLY+0.0798*HIS+0.2102*ILE+0.3143*LEU+ 0.1900*LYS+0.3581*MAN+0.1956*MANNOSE+0.0665*MET+ 0.0044*ORN+0.1617*PHE+0.1273*PRO+0.1550*R5P+0.2841*SER+ 0.2349*THR+0.0398*TRP+0.1247*TYR+0.3103*VAL $\rightarrow$ BIOMass

Table B. Equality constraints for net (.n) and exchange (.x) fluxes in [mmol/(g_{CDW}·h)].

BM_CYS.n = 0.000001
Help_Ru5P_X5P.n = 0.0001
Pen_Syn_VAL.n = Pen2.n
Pen_Syn_CYS.n = Pen2.n
Tca5a.n - Tca5b.n = 0
Tca6a.n - Tca6b.n = 0
Tca7a.n - Tca7b.n = 0
Tca5a.x - Tca5b.x = 0
Tca6a.x - Tca6b.x = 0
Tca7a.x - Tca7b.x = 0

Table C. Inequality constraints for net (.n) and exchange (.x) fluxes in [mmol/(g_{CDW}·h)].

Ac3.n $\geq$ 0
Ana1.n $\geq$ 0
Ana1.n $\leq$ gly6.n
Ana2.n $\geq$ 0
Ana3.n $\leq$ 0
gly7.n $\leq$ gly6.n
Gly1.n $\geq$ 0
Ppp1.n $\geq$ 0
Thr2.n $\leq$ 0
Transporter_ACCOA.n $\geq$ 0

Table D. Flux values of independent (free) fluxes (.n – net flux, .x – exchange flux).

Free fluxes	Flux value [mmol/(g_{CDW}·h)]	GC-MS	LC-MS	LC-MS/MS	^{13}C-NMR	^{1}H-NMR	GC-C-IRMS
ByProd_ex.n	0.0049	×	×	×	×	×	×
Ana1.n	0.0125	×	×	×	×	×	×
Ana1.x	140.6222	×	×	×	×	×	×
Ana2.x	5.0e-07	×	×	×	×	×	×
Ana3.x	5.0e-07						×
Asp1b.n	0.0272					×	×
CO2_ex.n	1.3014						
gly2.x	700.5319	×	×	×	×		×
Gly1.n	0.4530						
Gly1.x	0.0279						×
Gly2.x	0.0056					×	×
gly4.x	0.4530	×	(×)	(×)	×	×	×
gly4b.x	2.4203	×	(×)	(×)	×	×	×
gly5.x	0.0623	(×)	(×)	(×)	×	×	×
gly6.x	0.5386	(×)	(×)	(×)	×	×	×
gly7.n	0.5543						
Help_Ru5P.n	5.0e-07					×	×
Ppp2.n	0.2881						×
Ppp3.x	2.5896	(×)	(×)	(×)	×	×	×
Ppp4.x	0.1570					×	×
Ppp5.x	0.0490						×
Ppp6.x	0.5402					×	×
Ppp7.x	5.0e-07						×
Tca1.n	0.3805					×	
Tca5b.x	0.0370					×	×
Tca6a.x	5.0e-07					×	×
Tca7a.x	1.2058					×	×
Tca8.n	0.2919						
Tca8.x	202.9183	×	×	×	×	×	×
Thr2.x	104.9220	×	×	×	×	×	×
Transporter_ACCOA.x	5.0e-07						×
Transporter_CO2.n	1.0419					×	×
Transporter_OAA.x	0.2046	×	×	×	(×)	×	×
Transporter_PYR.n	0.4688					×	×

Table E. Flux values of independent (free) fluxes (.n – net flux, .x – exchange flux).

Free fluxes	GC-MS	LC-MS	LC-MS/MS	^{13}C-NMR	^{1}H-NMR	GC-C-IRMS
# statistically identifiable	24	26	26	22	11	5
# statistically non-identifiable	10	8	8	12	23	29

Out of the in total 34 free fluxes, several fluxes had to be constrained to ensure comparability among different analytical platforms. Fluxes marked with "×" were statistically non-identifiable. The symbol "(×)" indicates fluxes which were statistically identifiable but are set to constant values to guarantee comparability between models for different measurement setups, i.e., equal nominal model dimensionality (s. a. the main text or further explanation). The statistical flux (non-)identifiability study was performed with 60% [1-^{13}C]-glucose, 20% [U-^{13}C]-glucose and 20% [^{12}C]-glucose as substrate mixture.

Table F. Extracellular rate measurements with associated standard deviations in [mmol/(g_{CDW}·h)].

gly1.n = 0.1169 ± 0.00865

POA_IN.n = 0.0160 ± 0.00739

PEN2.n = 0.0107 ± 0.00259

BIOM_EX = 0.0085 ± 0.00207

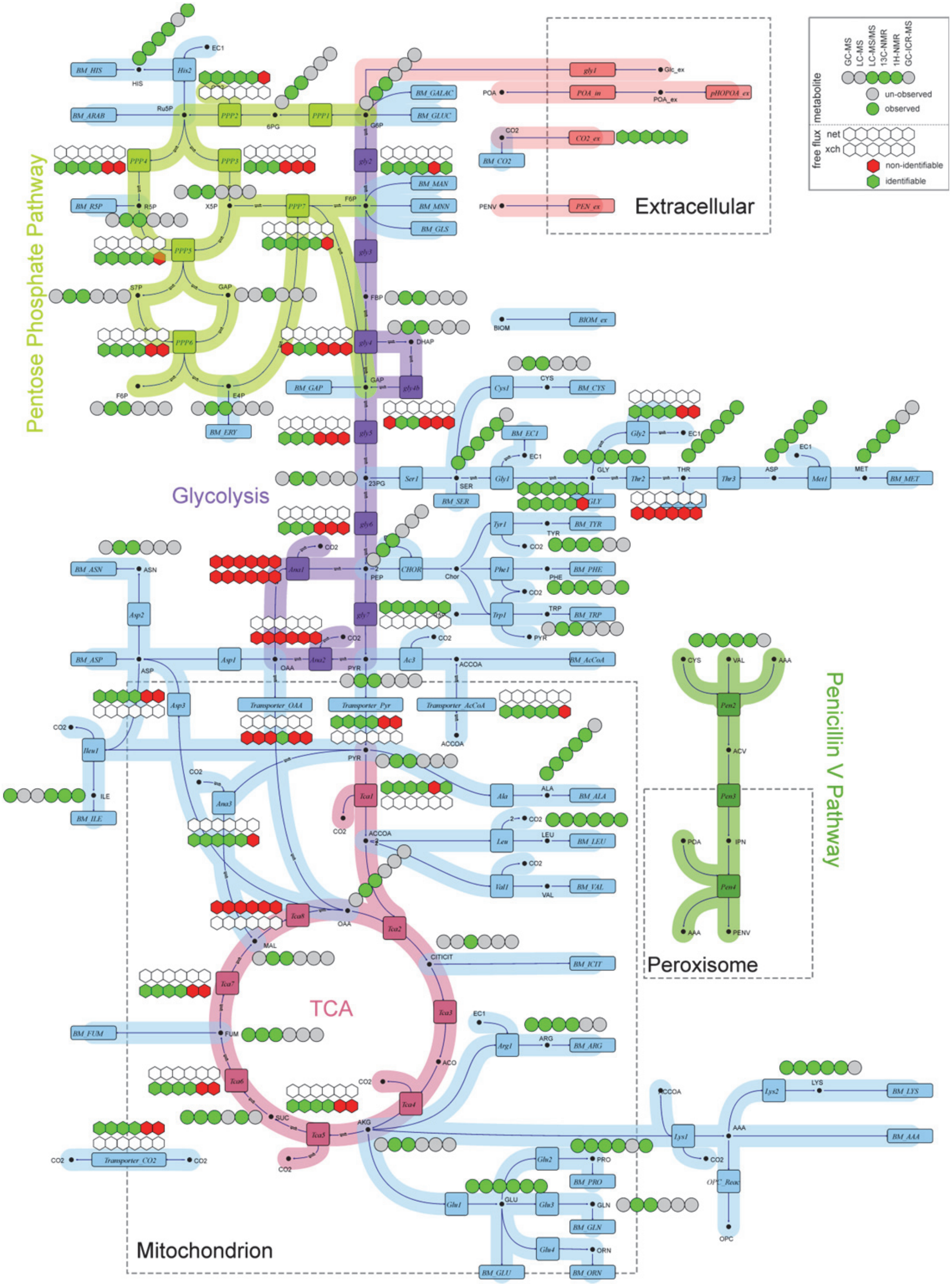

Fig A. Metabolic map indicating labeling measurements and statistically identifiable and non-identifiable free fluxes (green and red hexagons, respectively) resolved by analytical platforms GC-MS, LC-MS, LC-MS/MS, ^{1}H-NMR, and ^{13}C-NMR (green cycles: observed, grey cycle un-observed).
