# Supplementary material for: A Pareto approach to resolve the conflict between information gain and experimental costs: Multiple-criteria design of carbon labeling experiments
Source: PLoS Comput Biol. 2018 Oct 31;14(10):e1006533. doi: 10.1371/journal.pcbi.1006533 (PMC6209137; doi:10.1371/journal.pcbi.1006533)
Supplement: S4 Text — Documentation of results for the analytical platforms GC-MS, LC-MS, LC-MS/MS, 13C-NMR and platform combinations. (PDF) [file pcbi.1006533.s004.pdf]

Supplementary Appendix 4 for

A Pareto approach to resolve the conflict between information gain and experimental costs: Multiple-criteria design of carbon labeling experiments

Multi-objective experimental design: Results for the 3D scenario

Katharina Nöh, Sebastian Niedenführ, Martin Beyß, Wolfgang Wiechert

k.noeh@fz-juelich.de

Contents

1. GC-MS 2
2. LC-MS 10
3. LC-MS/MS 18
4. ^{13}C-NMR 26
5. Objective space ranges 35
6. Multi-platform applications 39
   6.1. ^{1}H-NMR + ^{13}C-NMR 39
   6.2. GC-MS + LC-MS 41
   6.3. LC-MS/MS + ^{13}C-NMR 45

1. GC-MS

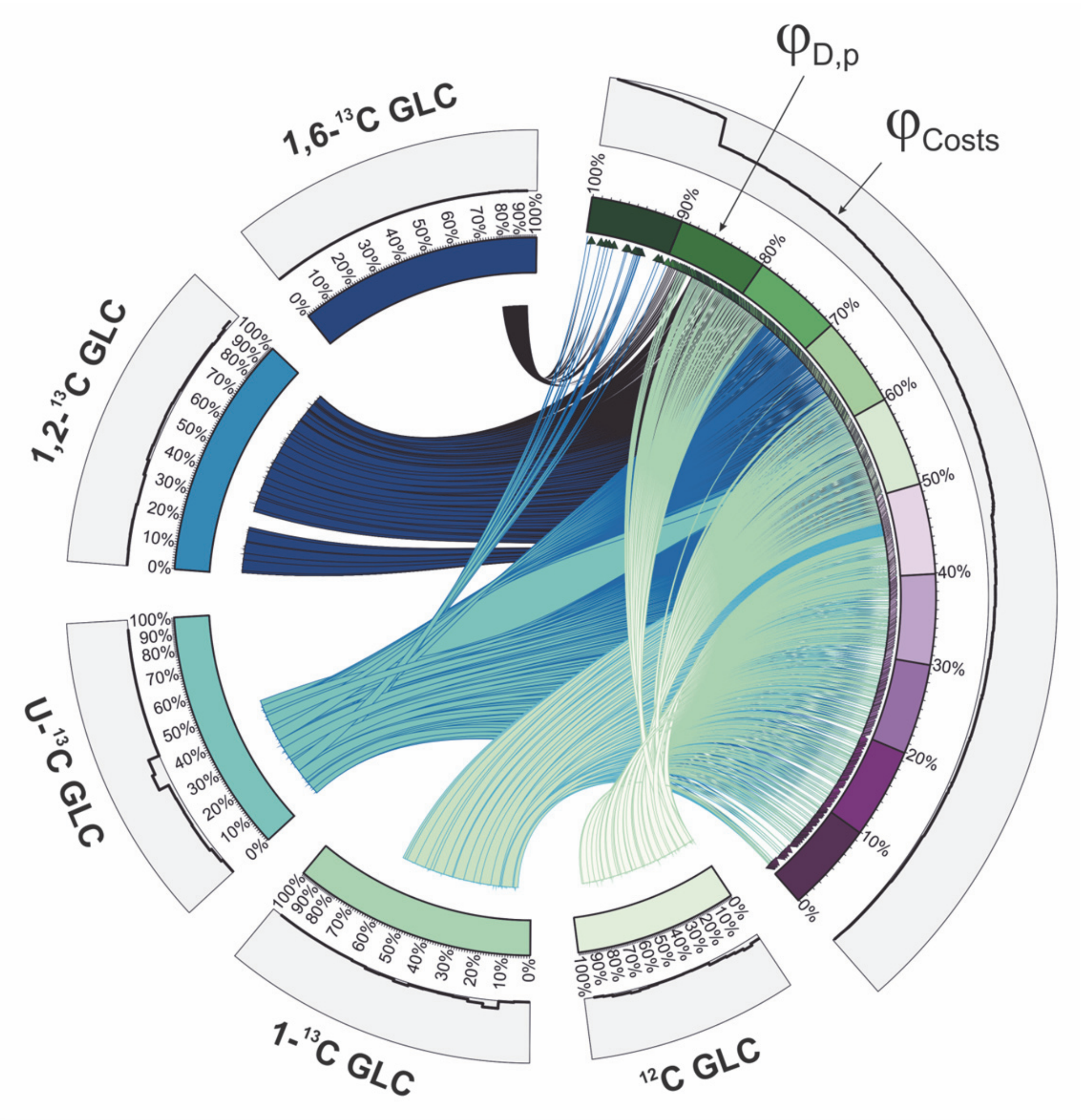

Fig A. 3D-MO-ED ^{13}C-MFA design space for GC-MS (p=21). Enlarged version of cord diagram shown in Fig 7 of the main text.

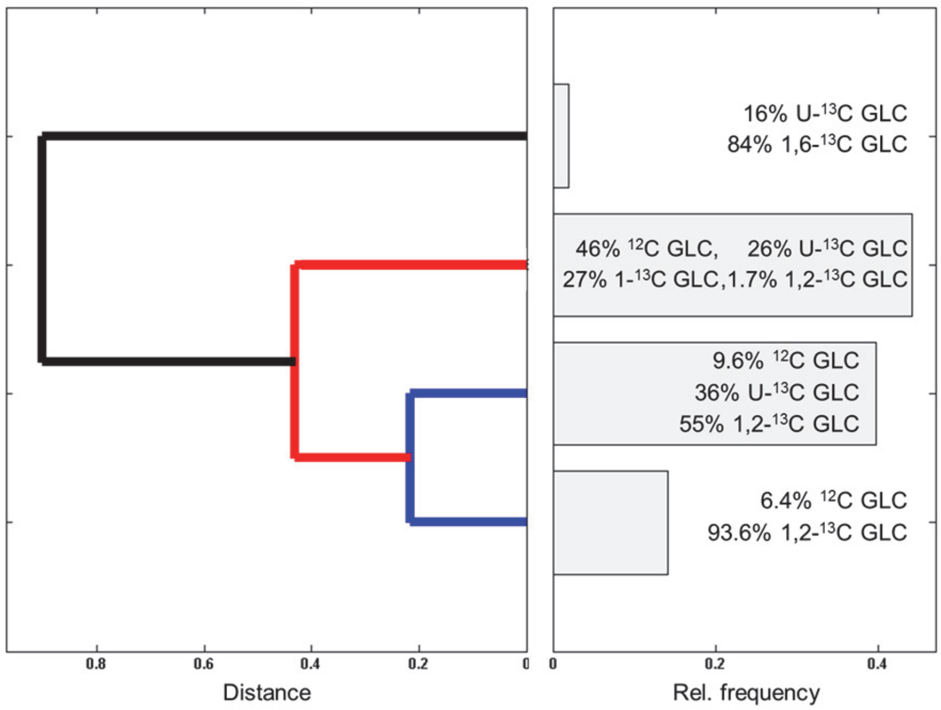

Fig B. Dendrogram of substrate mixture clusters for GC-MS (p=21). The best known Pareto set of the 3D-MO-ED problem was hierarchically clustered by the minimal Euclidean distance of the labeling fraction composition. Values below 1% were omitted. The length of the edges (distance) represents the dissimilarities of the mixtures. Four different mixture clusters were determined for which composition average values along with their relative frequency. The corresponding number of measurement groups and replicates resolved by the four clusters are given in S4 Fig C–F.

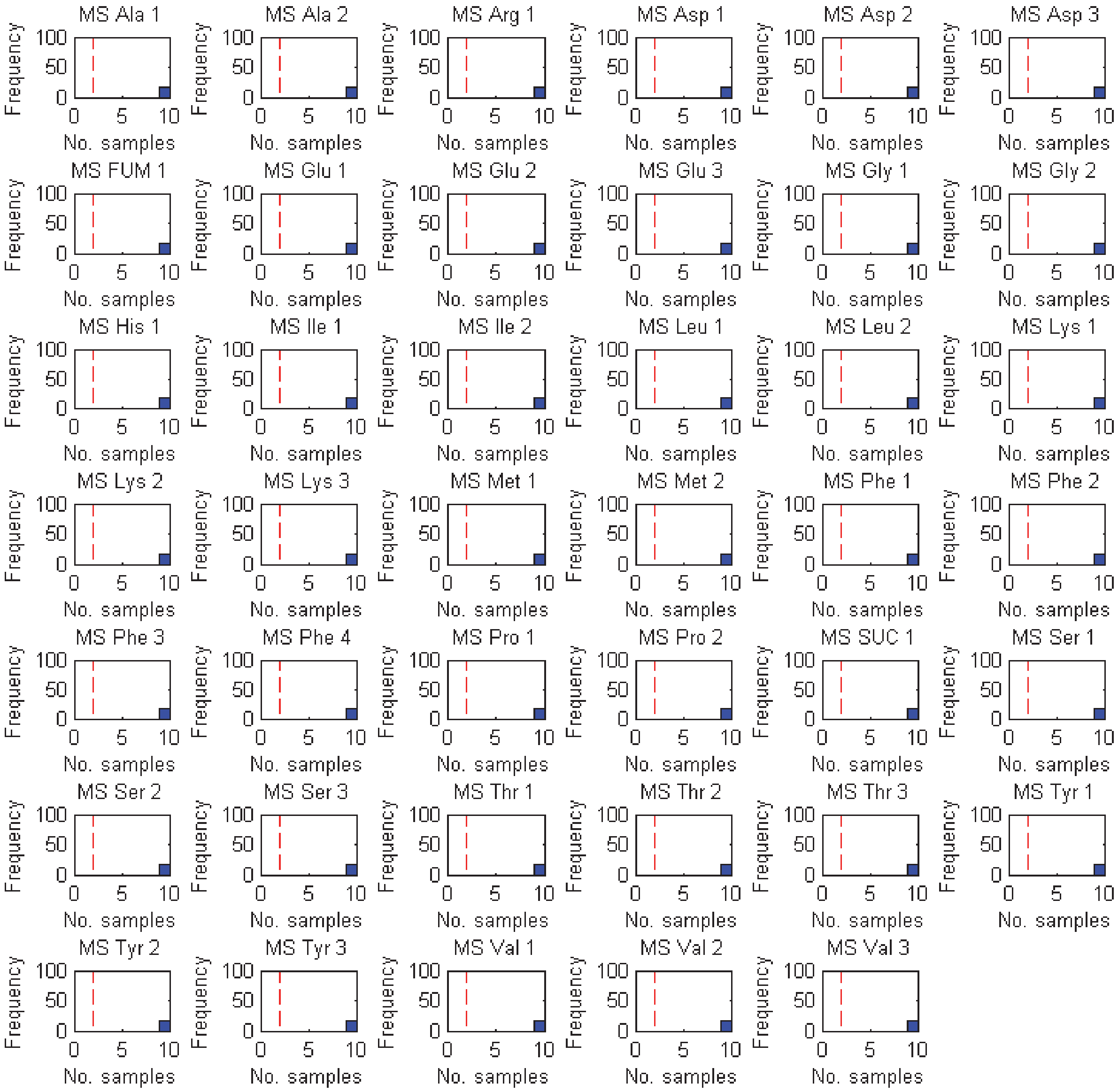

Fig C. Measurement groups and replicate numbers for the 3D-MO-ED solution for GC-MS and substrate mixture cluster #1: 15.69% [U-^{13}C]-glucose, 84.13% [1,2-^{13}C]-glucose (mean values). Measurement groups with less than two replicates (red line) were not considered in the analysis.

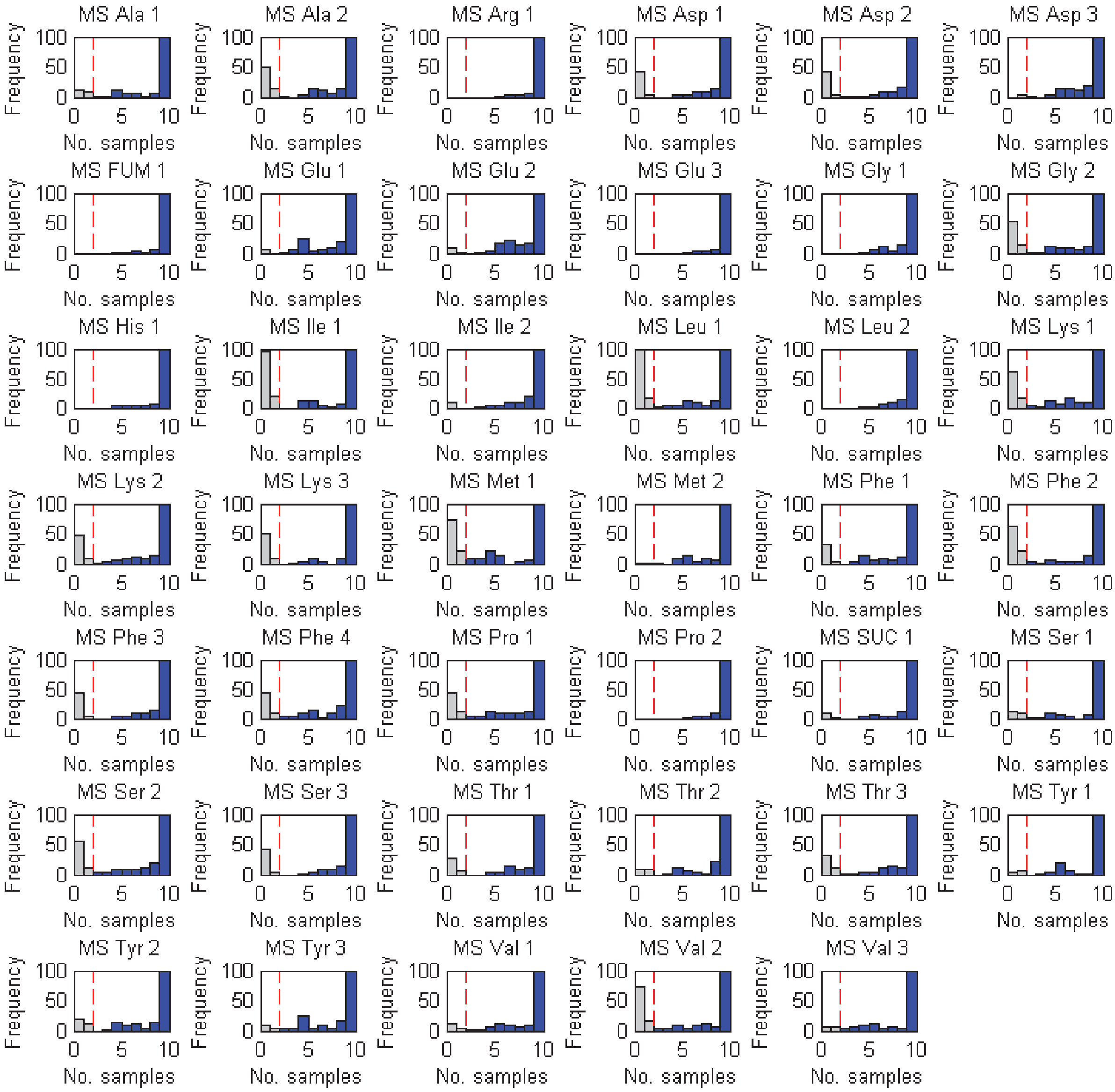

Fig D. Measurement groups and replicate numbers for the 3D-MO-ED solution for GC-MS and substrate mixture cluster #2: 45.53% [^{12}C]-glucose, 27.14% [1-^{13}C]-glucose, 25.61% [U-^{13}C]-glucose, 1.68% [1,2-^{13}C]-glucose (mean values). Measurement groups with less than two replicates (red line) were not considered in the analysis.

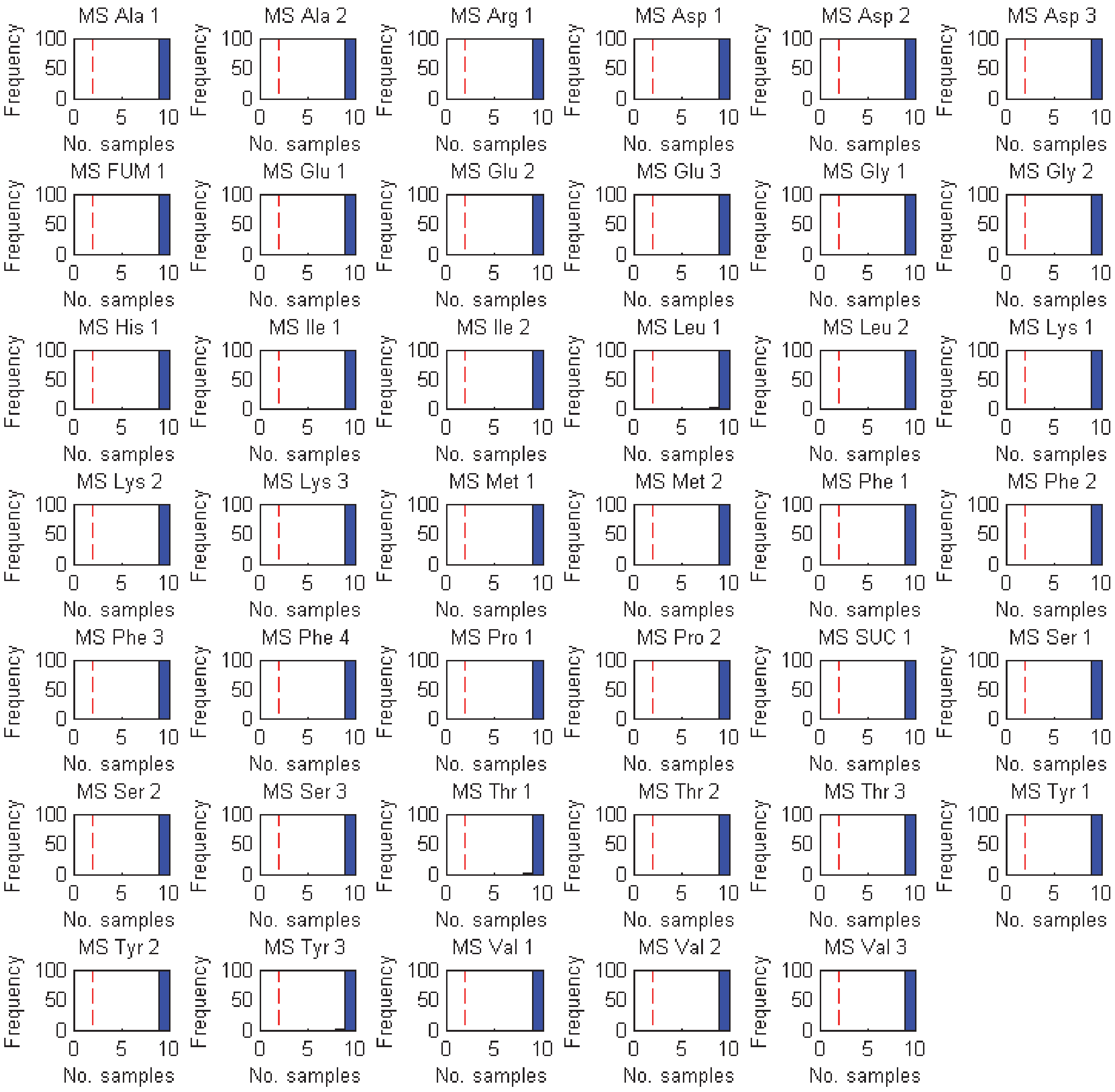

Fig E. Measurement groups and replicate numbers for the 3D-MO-ED solution for GC-MS and substrate mixture cluster #3: 9.59% [^{12}C]-glucose, 35.61% [U-^{13}C]-glucose, 54.76% [1,2-^{13}C]-glucose (mean values). Measurement groups with less than two replicates (red line) were not considered in the analysis.

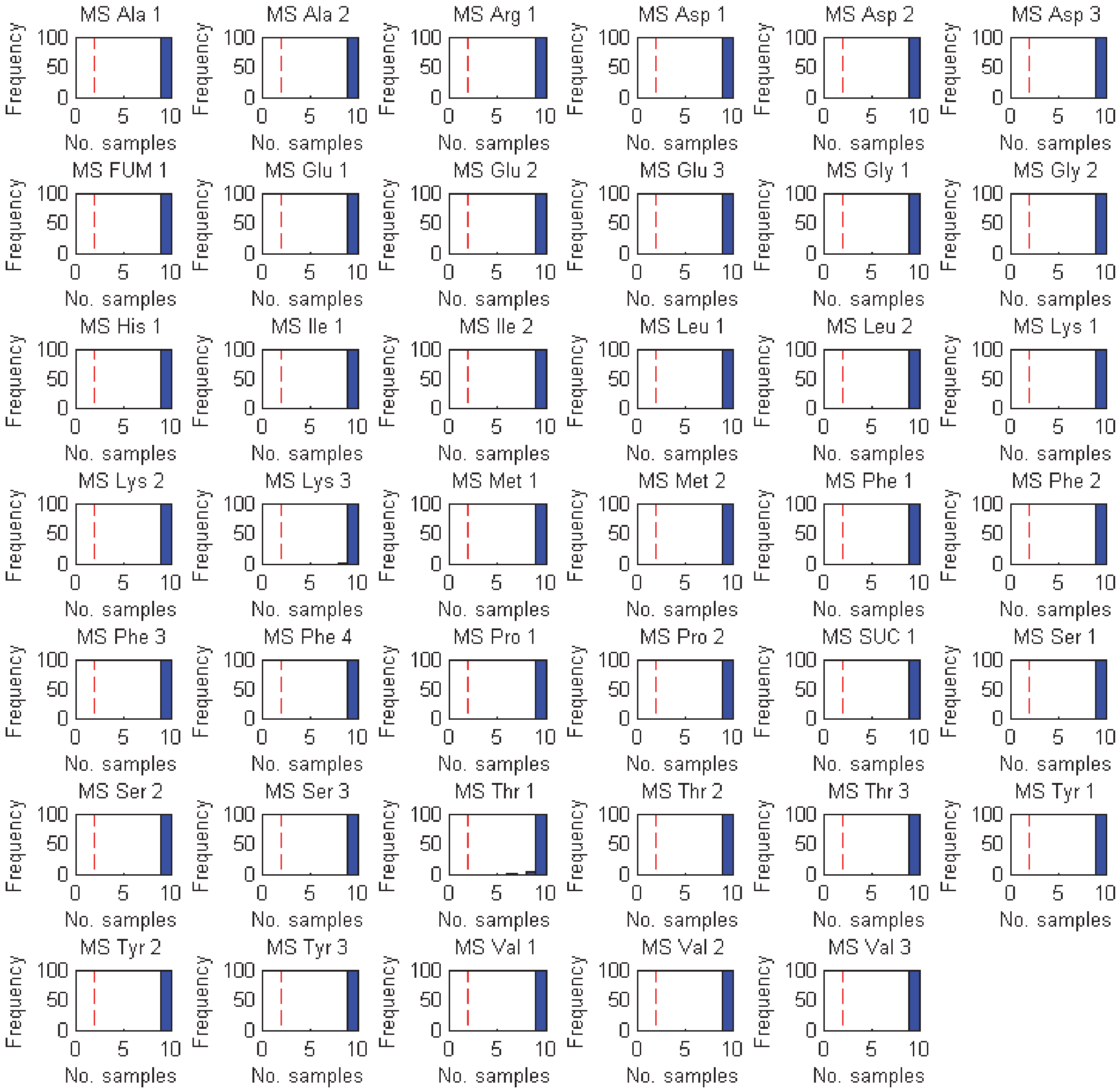

Fig F. Measurement groups and replicate numbers for the 3D-MO-ED solution for GC-MS and substrate mixture cluster #4: 6.41% [^{12}C]-glucose, 93.49% [1,2-^{13}C]-glucose (mean values). Measurement groups with less than two replicates (red line) were not considered in the analysis.

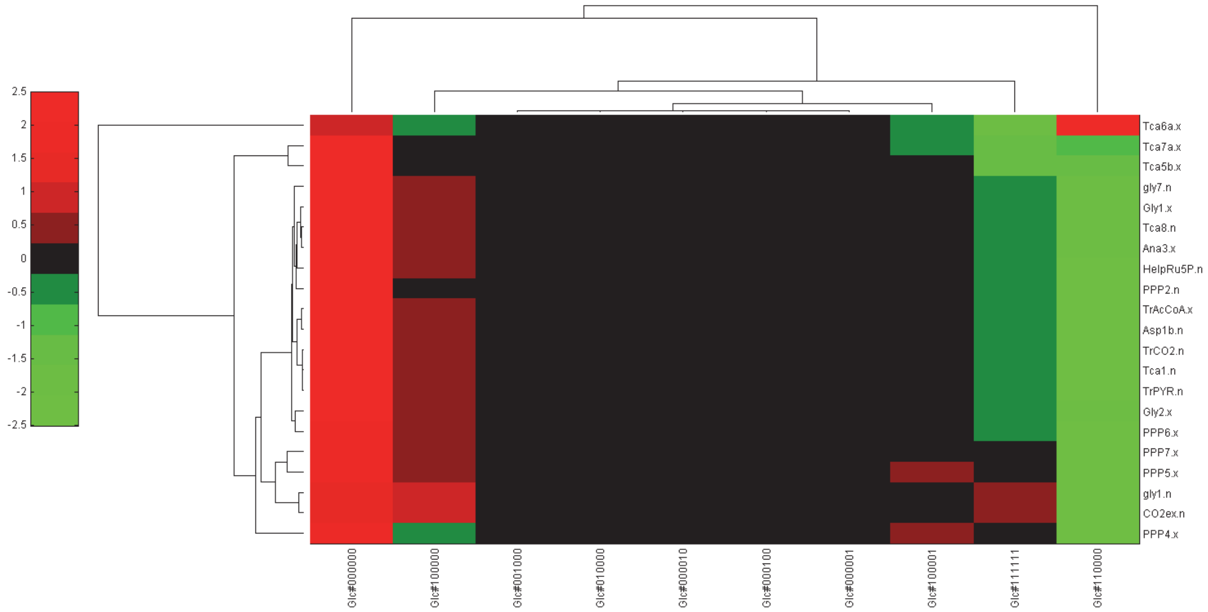

Fig G. Clustergram of Pareto-optimal 3D-MO-ED correlations between flux STDs and input substrates for GC-MS (p=21).

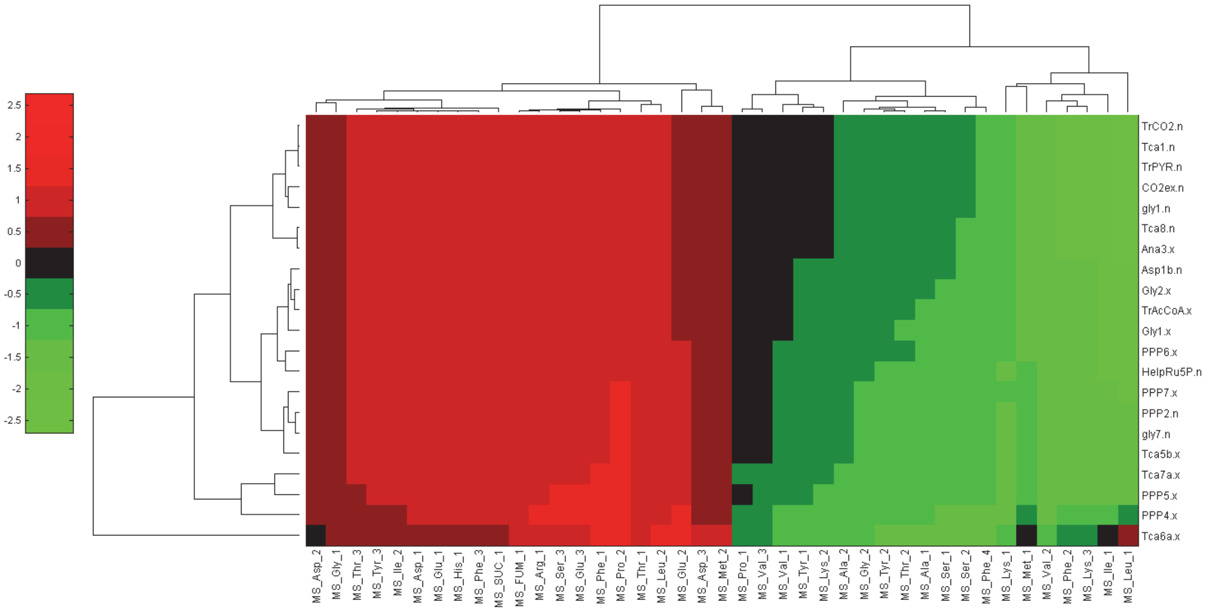

Fig H. Clustergram of Pareto-optimal 3D-MO-ED correlations between flux STDs and measurement groups for GC-MS (p=21).

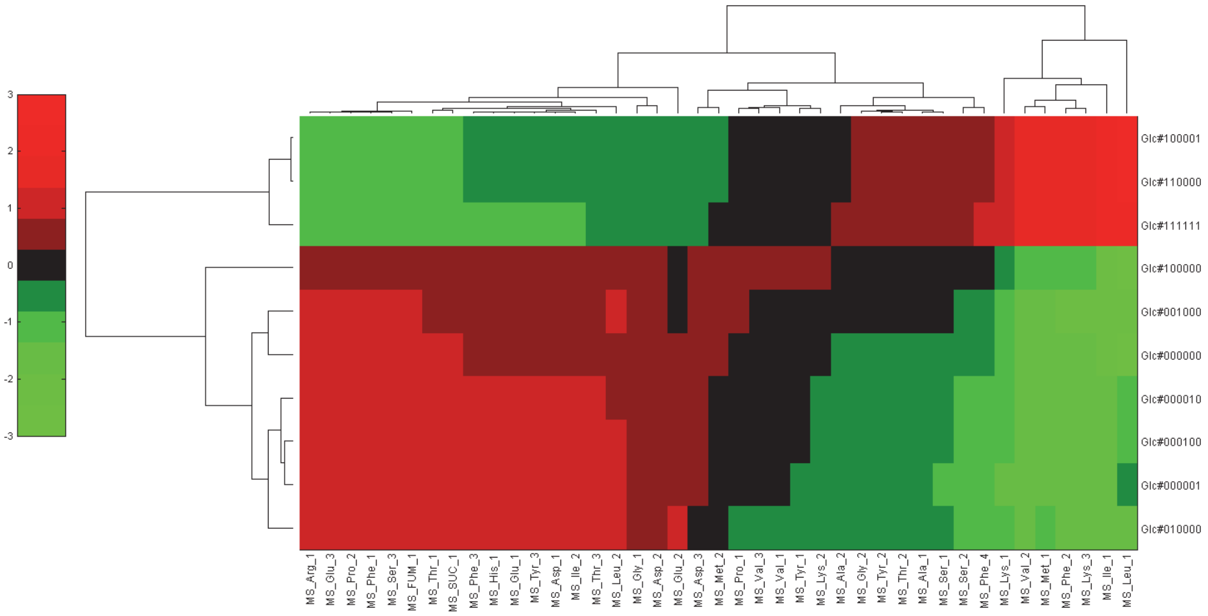

Fig I. Clustergram of Pareto-optimal 3D-MO-ED correlations between input species and measurement groups for GC-MS (p=21).

2. LC-MS

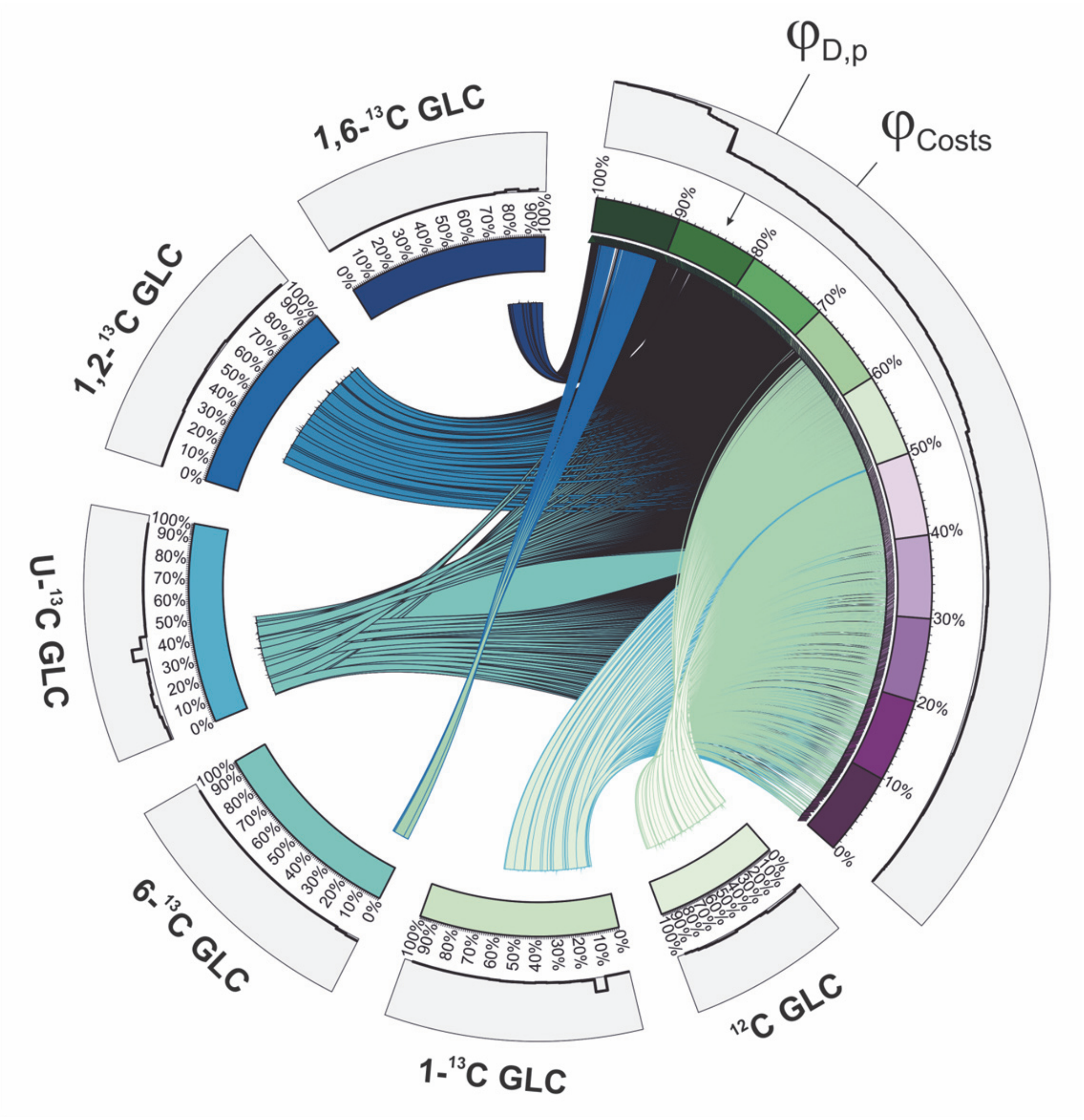

Fig J. 3D-MO-ED ^{13}C-MFA design space for LC-MS (p=21). Enlarged version of cord diagram shown in Fig 7 of the main text.

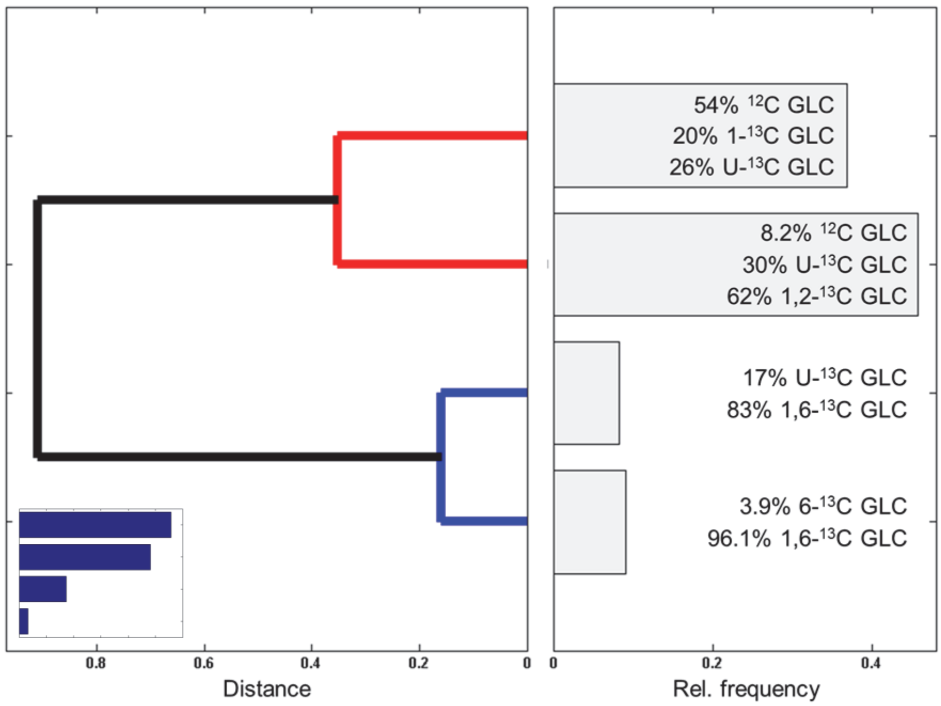

Fig K. Dendrogram of the substrate mixture clusters for LC-MS (*p*=21). The best known Pareto set of the 3D-MO-ED problem was hierarchically clustered by the minimal Euclidean distance of the labeling fraction composition. Values below 1% were omitted. The length of the edges (distance) represents the dissimilarities of the mixtures. Four different mixture clusters were determined for which average composition values are given along with their relative frequency. The clusters' cost proportions are shown in the inset. The corresponding number of measurement groups and replicates resolved by the four clusters are given in S4 Fig L–O.

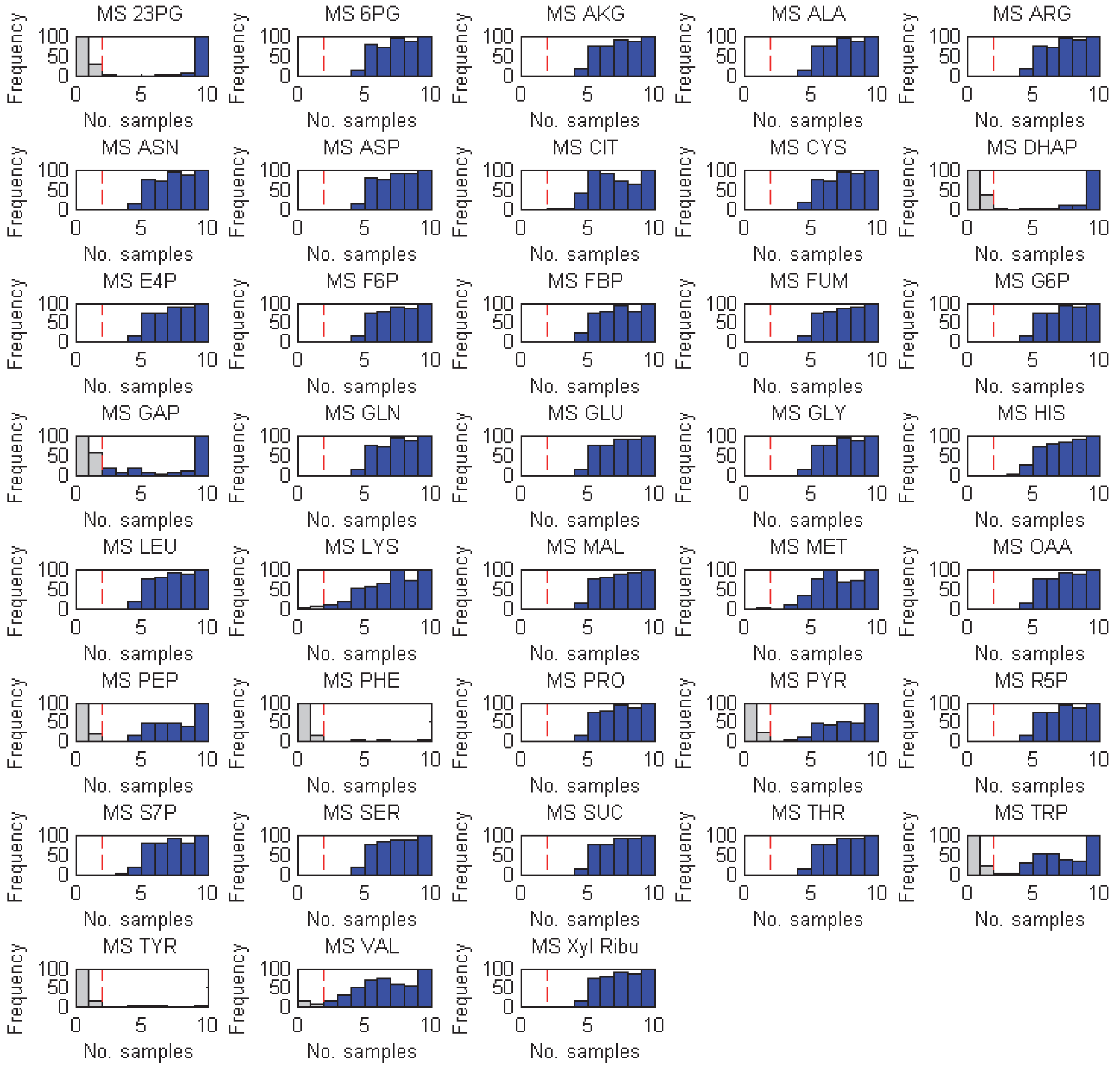

Fig L. Measurement groups and replicate numbers of the 3D-MO-ED solution for LC-MS and substrate mixture cluster #1: 53.71% [^{12}C]-glucose, 19.80% [1-^{13}C]-glucose, 26.44% [U-^{13}C]-glucose (mean values). Measurement groups with less than two replicates (red line) were not considered in the analysis.

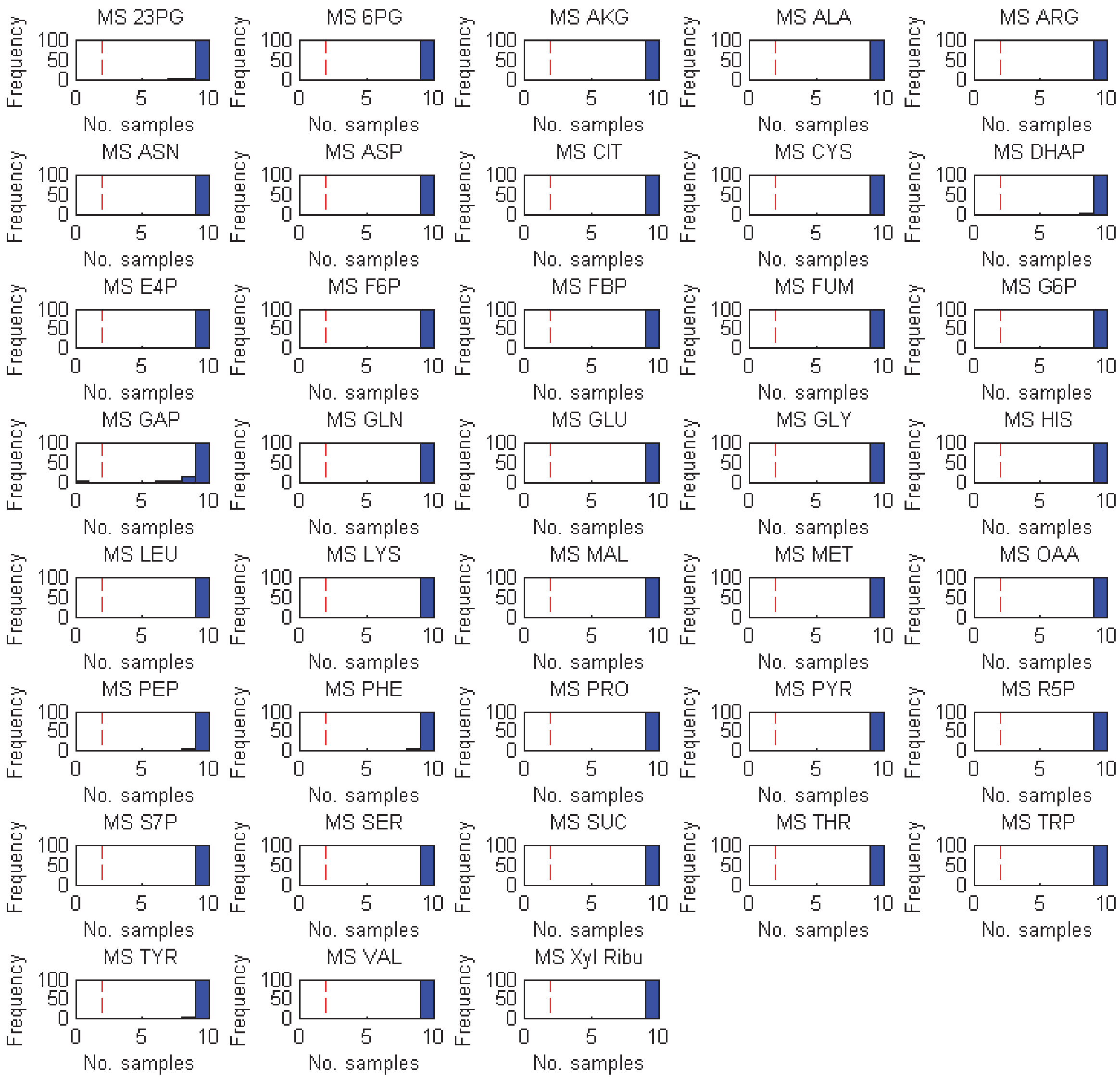

Fig M. Measurement groups and replicate numbers of the 3D-MO-ED solution for LC-MS and substrate mixture cluster #2: 8.16% [^{12}C]-glucose, 29.77% [U-^{13}C]-glucose, 62.03% [1,2-^{13}C]-glucose (mean values). Measurement groups with less than two replicates (red line) were not considered in the analysis.

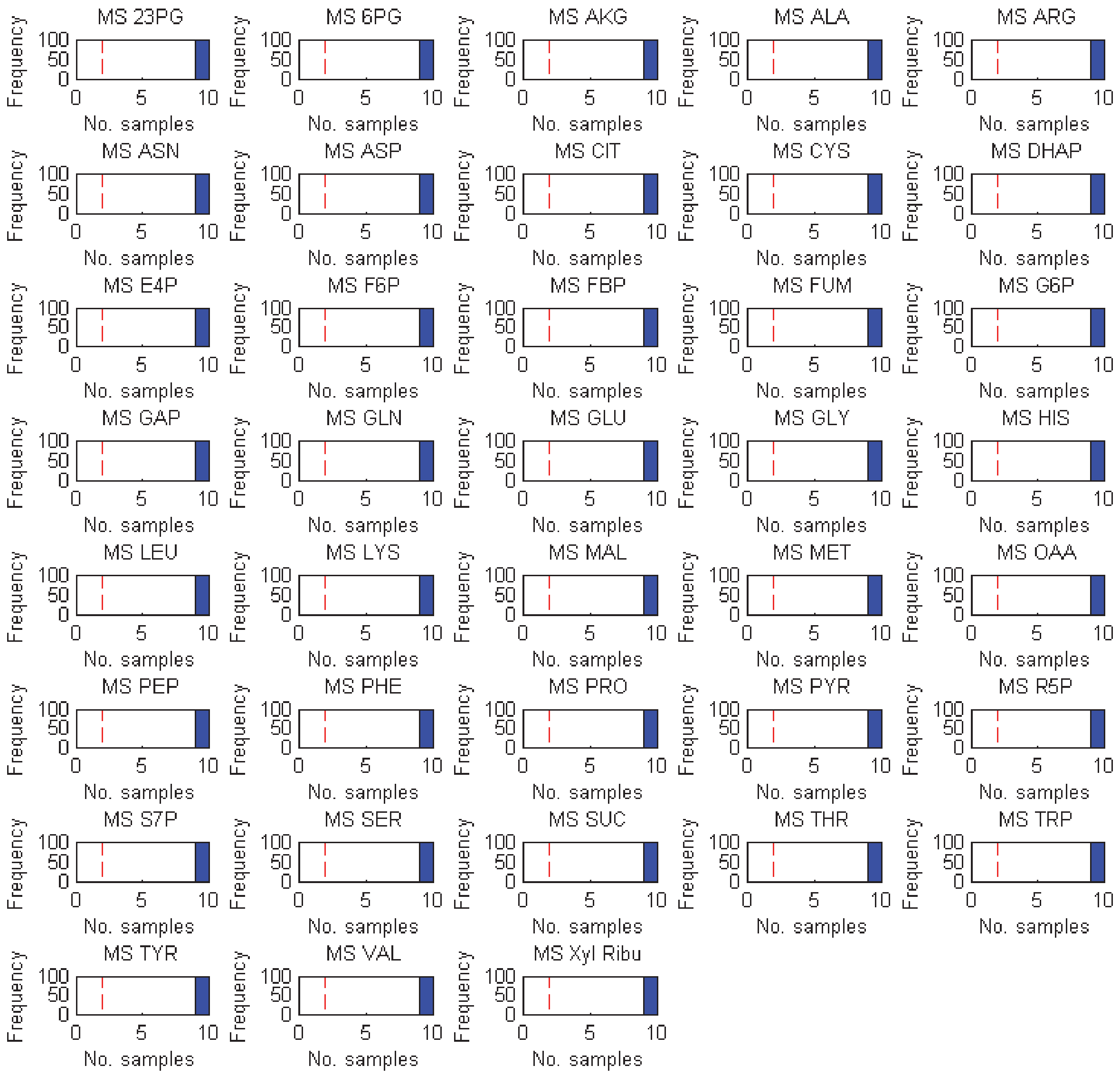

Fig N. Measurement groups and replicate numbers of the 3D-MO-ED solution for LC-MS and substrate mixture cluster #3: 17.47% [U-^{13}C]-glucose, 82.47% [1,6-^{13}C]-glucose (mean values). Measurement groups with less than two replicates (red line) were not considered in the analysis.

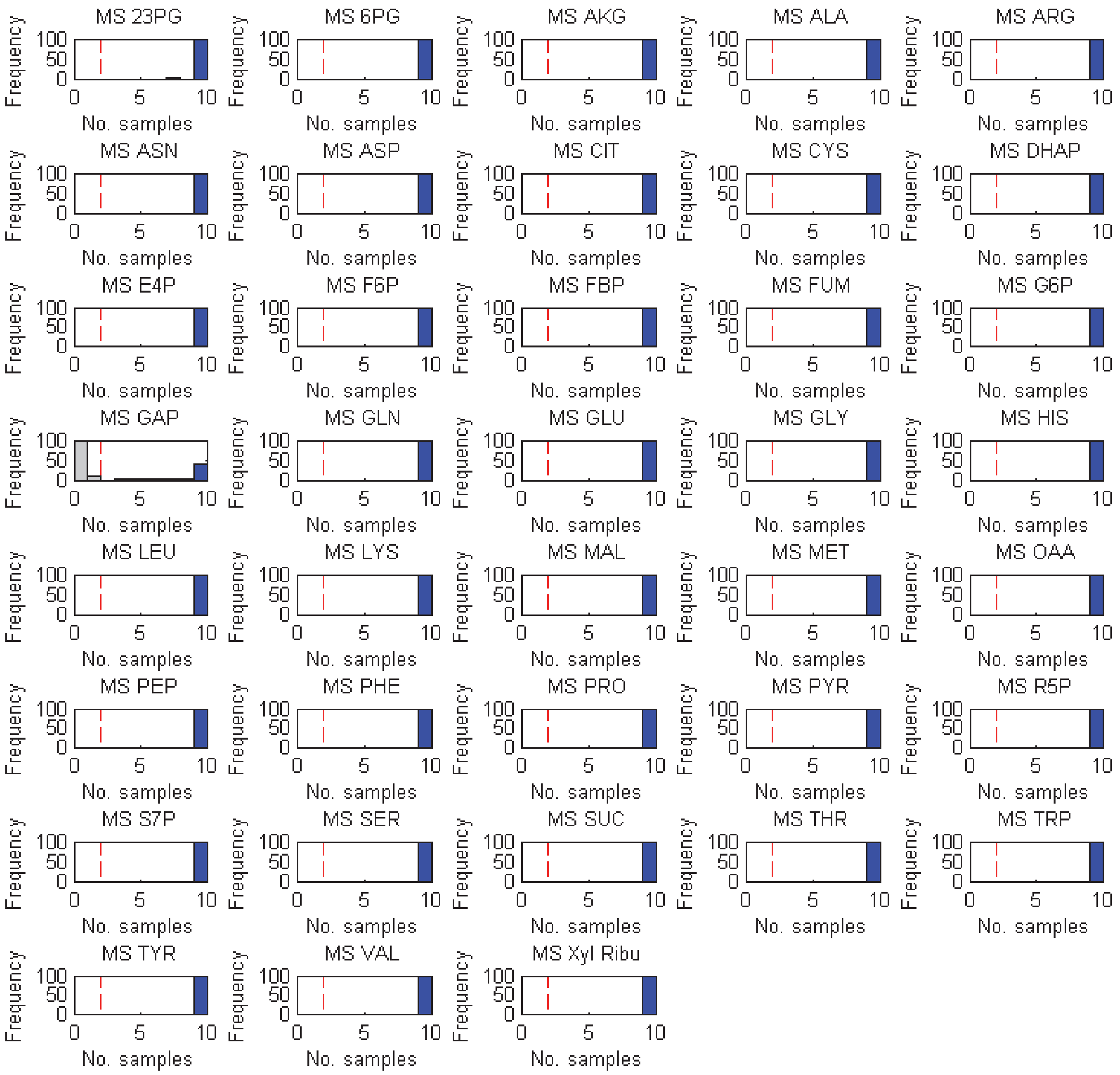

Fig O. Measurement groups and replicate numbers of the 3D-MO-ED solution for LC-MS and substrate mixture cluster #4: 3.86% [6-^{13}C]-glucose, 96.06% [1,6-^{13}C]-glucose (mean values). Measurement groups with less than two replicates (red line) were not considered in the analysis.

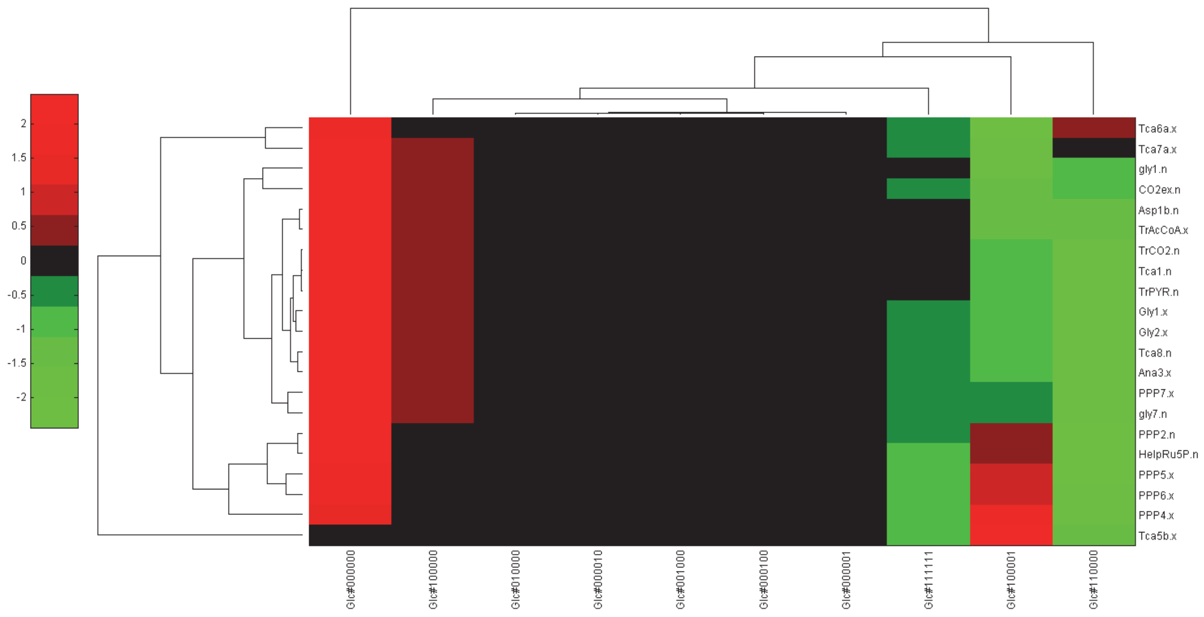

Fig P. Clustergram of Pareto-optimal 3D-MO-ED correlations between flux STDs and input substrates for LC-MS (*p*=21).

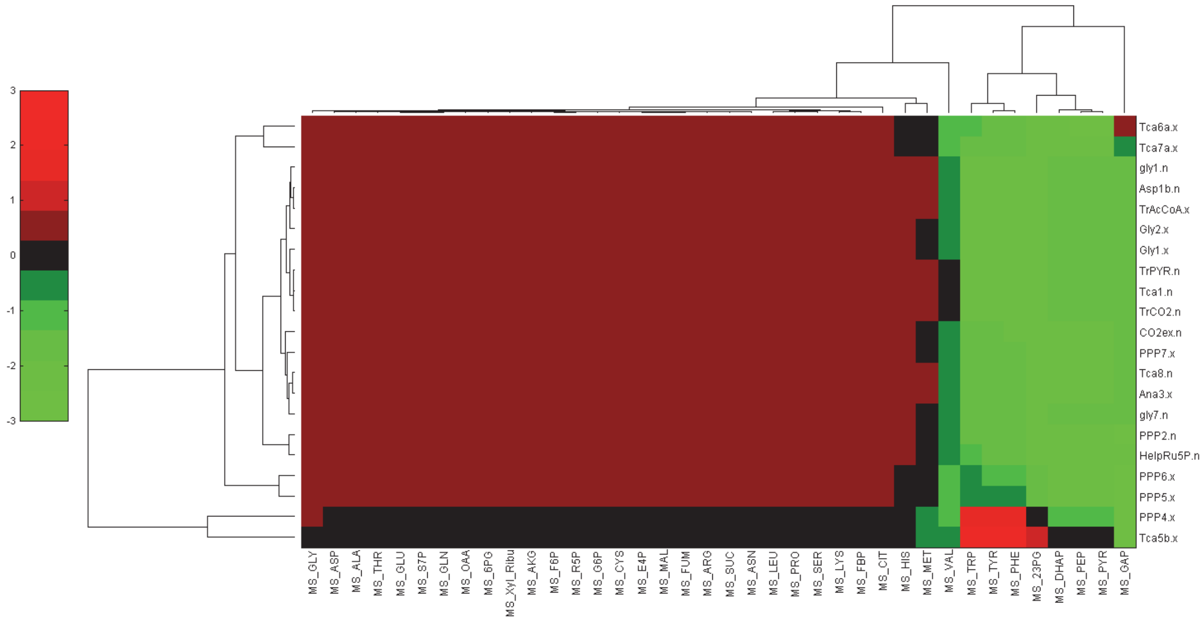

Fig Q. Clustergram of Pareto-optimal 3D-MO-ED correlations between flux STDs and measurement groups for LC-MS (*p*=21).

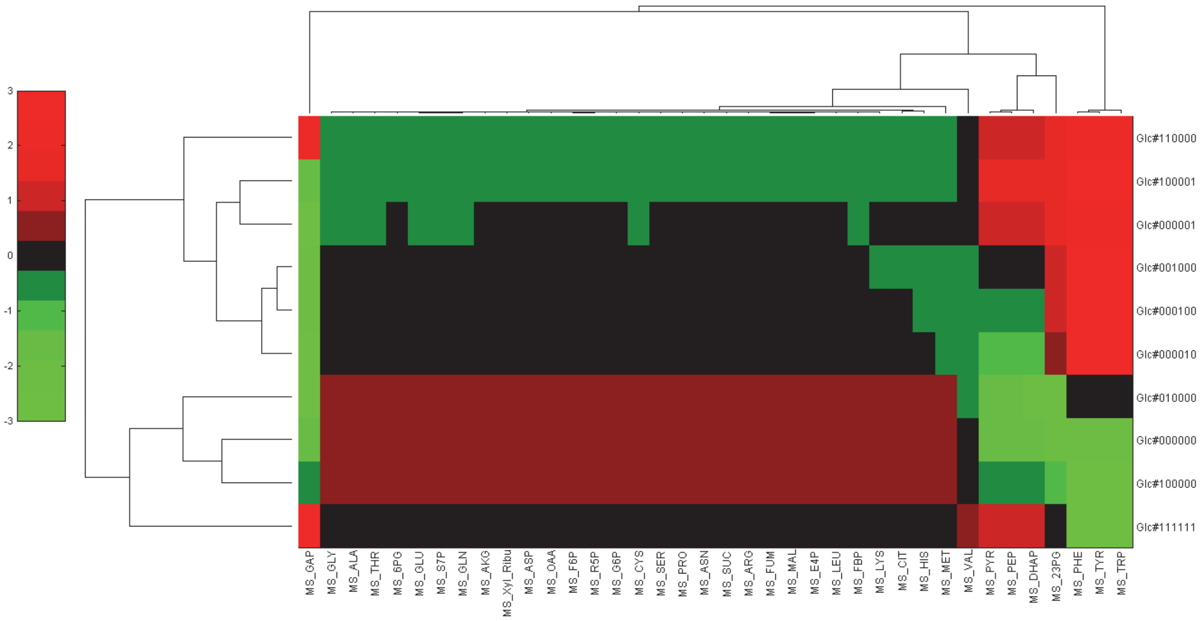

Fig R. Clustergram of Pareto-optimal 3D-MO-ED correlations between input species and measurement groups for LC-MS (p=21).

3. LC-MS/MS

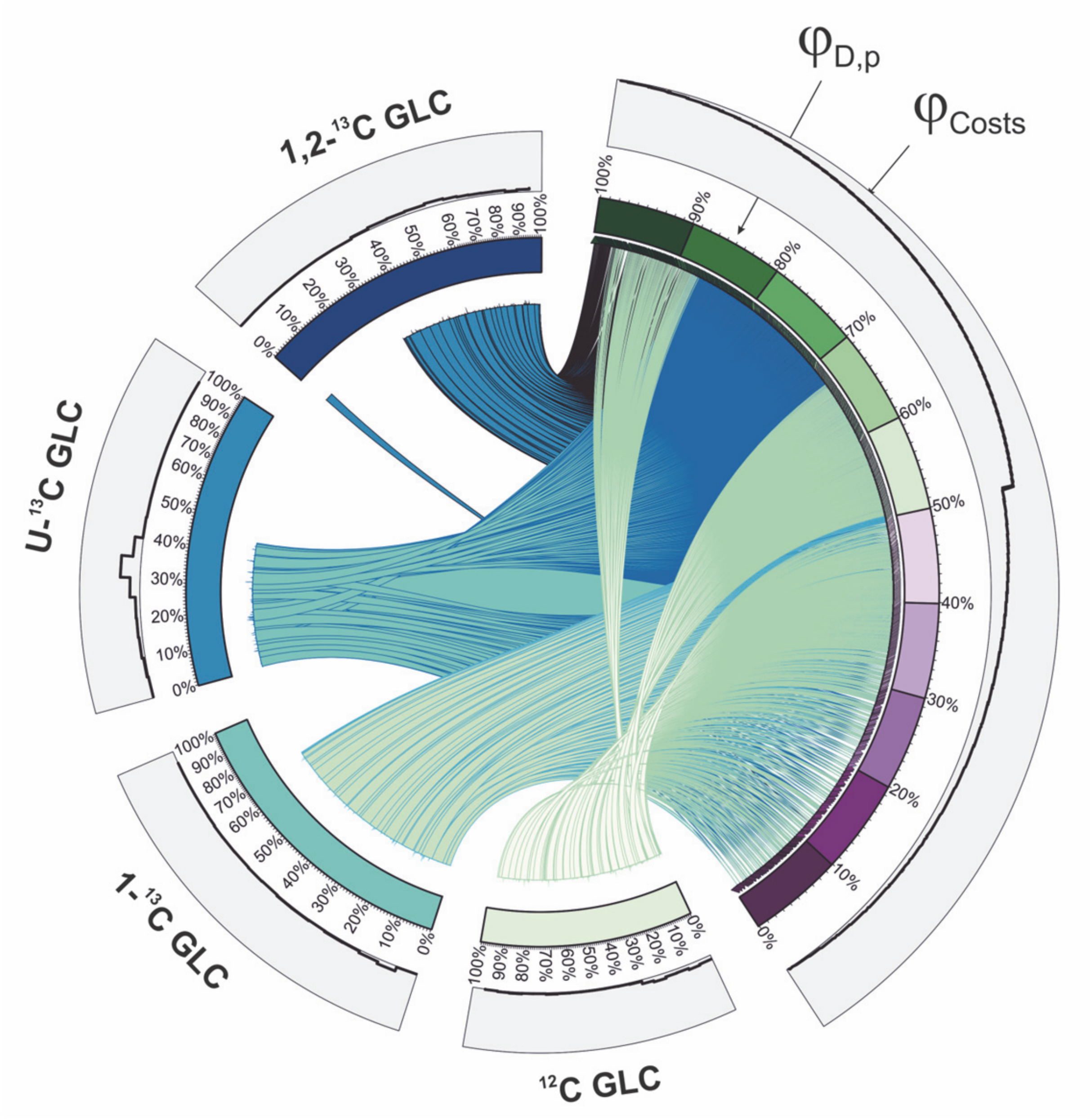

Fig S. 3D-MO-ED ^{13}C-MFA design space for LC-MS/MS (*p*=21). Enlarged version of cord diagram shown in Fig 7 of the main text.

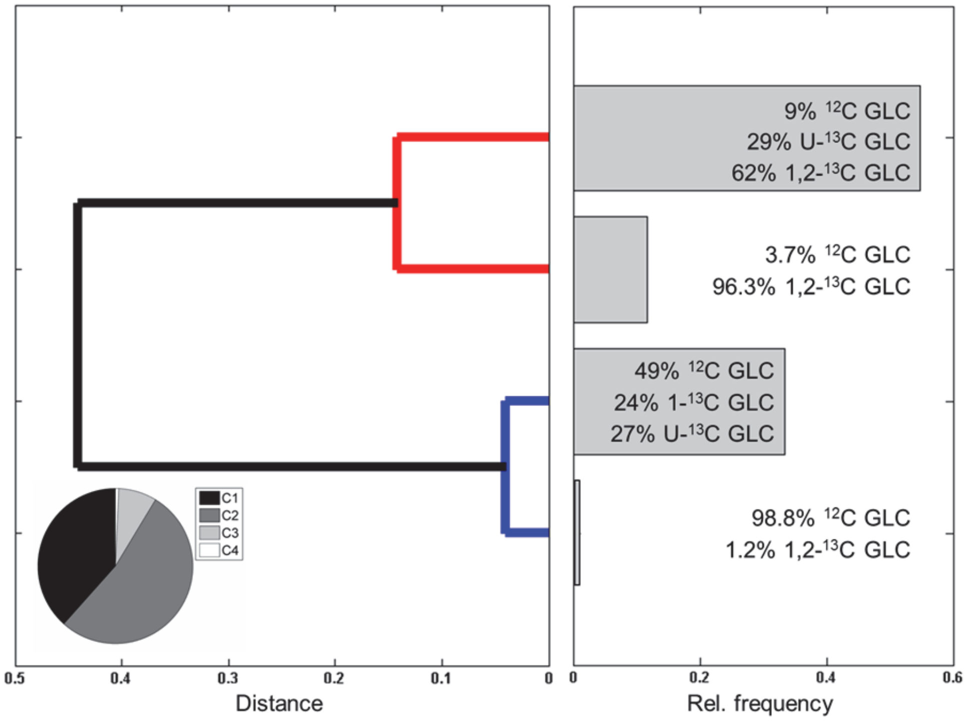

Fig T. Dendrogram of the substrate mixture clusters for LC-MS/MS (p=21). The best known Pareto set of the 3D-MO-ED problem was hierarchically clustered by the minimal Euclidean distance of the labeling fraction composition. Values below 1% were omitted. The length of the edges (distance) represents the dissimilarities of the mixtures. Four different mixture clusters were determined for which composition average values are given along with their relative frequency. The clusters' cost proportions are shown in the pie chart. The corresponding number of measurement groups and replicates resolved by the four clusters are given in S4 Fig U–X.

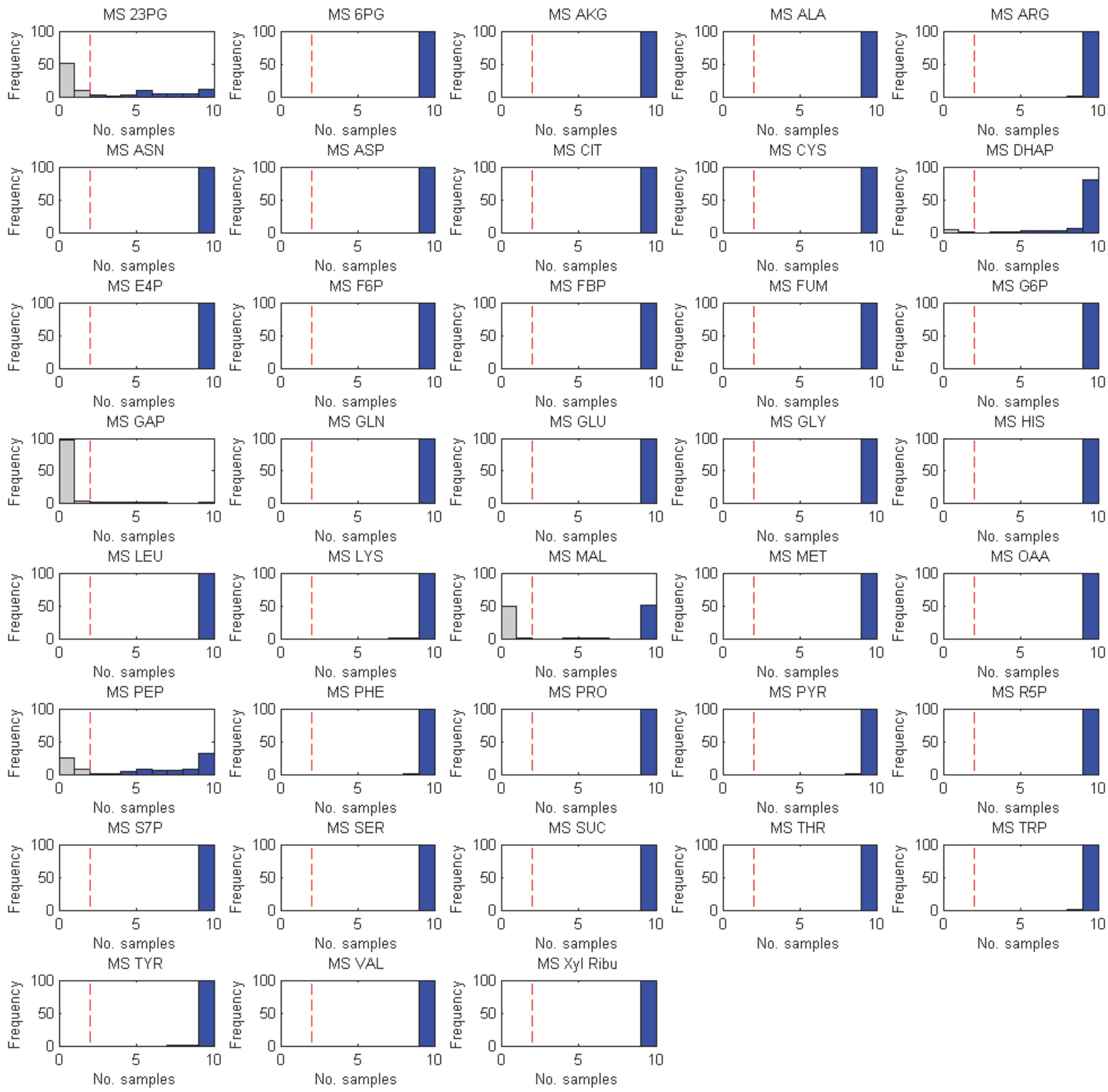

Fig U. Measurement groups and replicate numbers of the 3D-MO-ED solution for LC-MS/MS and substrate mixture cluster #1: 9% [^{12}C]-glucose, 29% [U-^{13}C]-glucose, 62% [1,2-^{13}C]-glucose. Measurement groups with less than two replicates (red line) were not considered in the analysis.

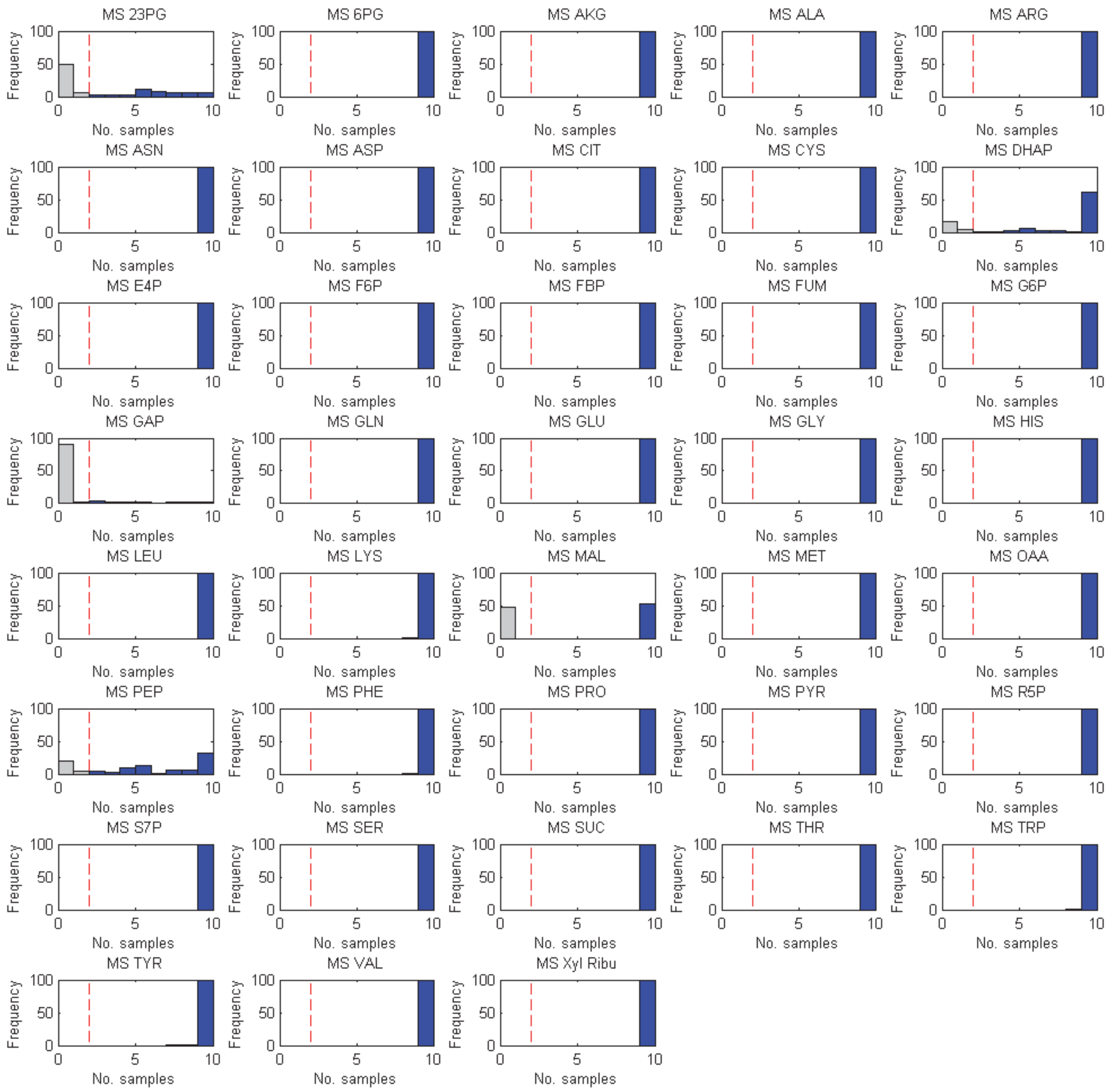

Fig V. Measurement groups and replicate numbers of the 3D-MO-ED solution for LC-MS/MS and substrate mixture cluster #2: 3.7% [^{12}C]-glucose, 96.3% [1,2-^{13}C]-glucose. Measurement groups with less than two replicates (red line) were not considered in the analysis.

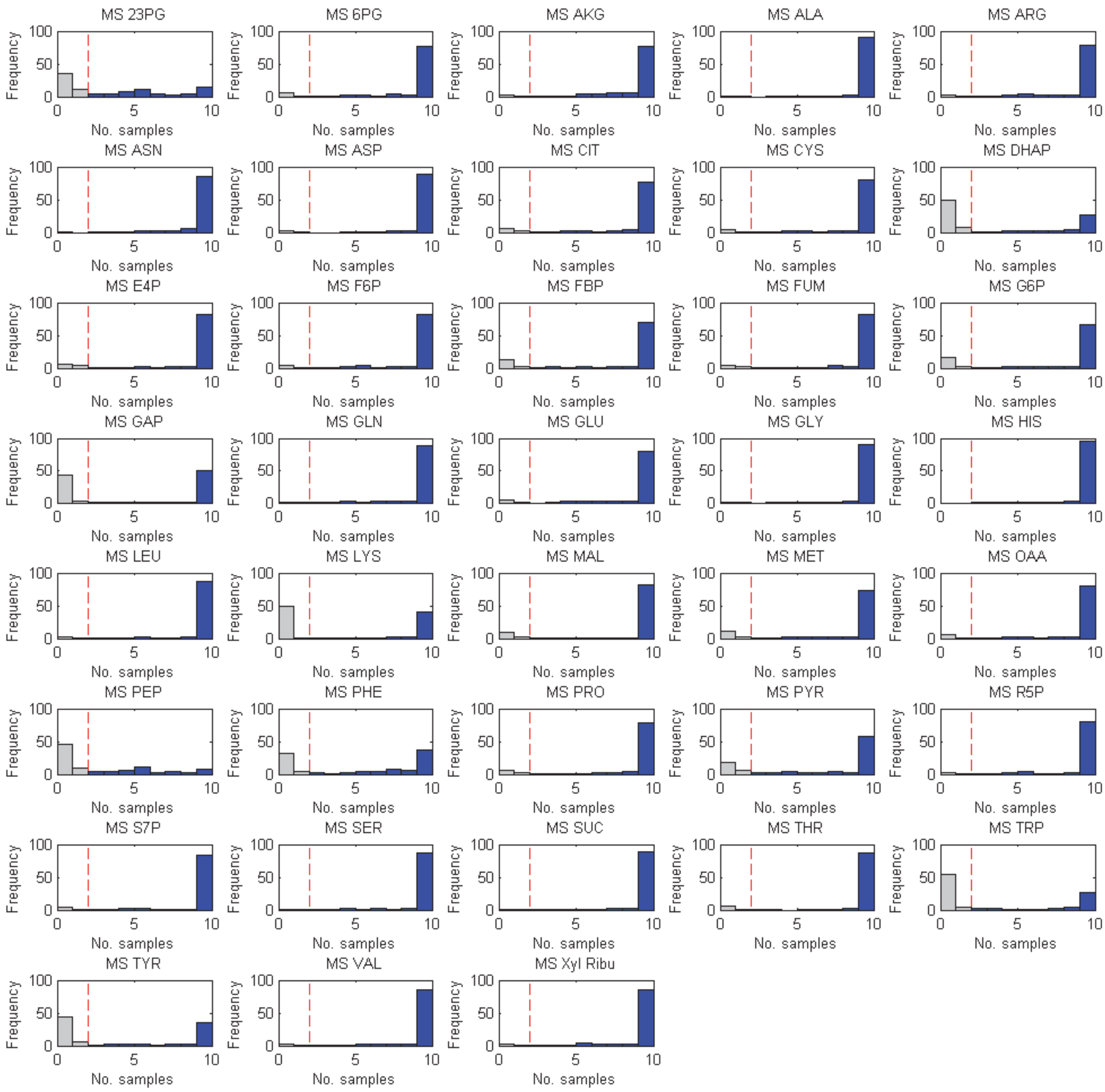

Fig W. Measurement groups and replicate numbers of the 3D-MO-ED solution for LC-MS/MS and substrate mixture cluster #3: 49% [^{12}C]-glucose, 24% [1-^{13}C]-glucose, 27% [U-^{13}C]-glucose. Measurement groups with less than two replicates (red line) were not considered in the analysis.

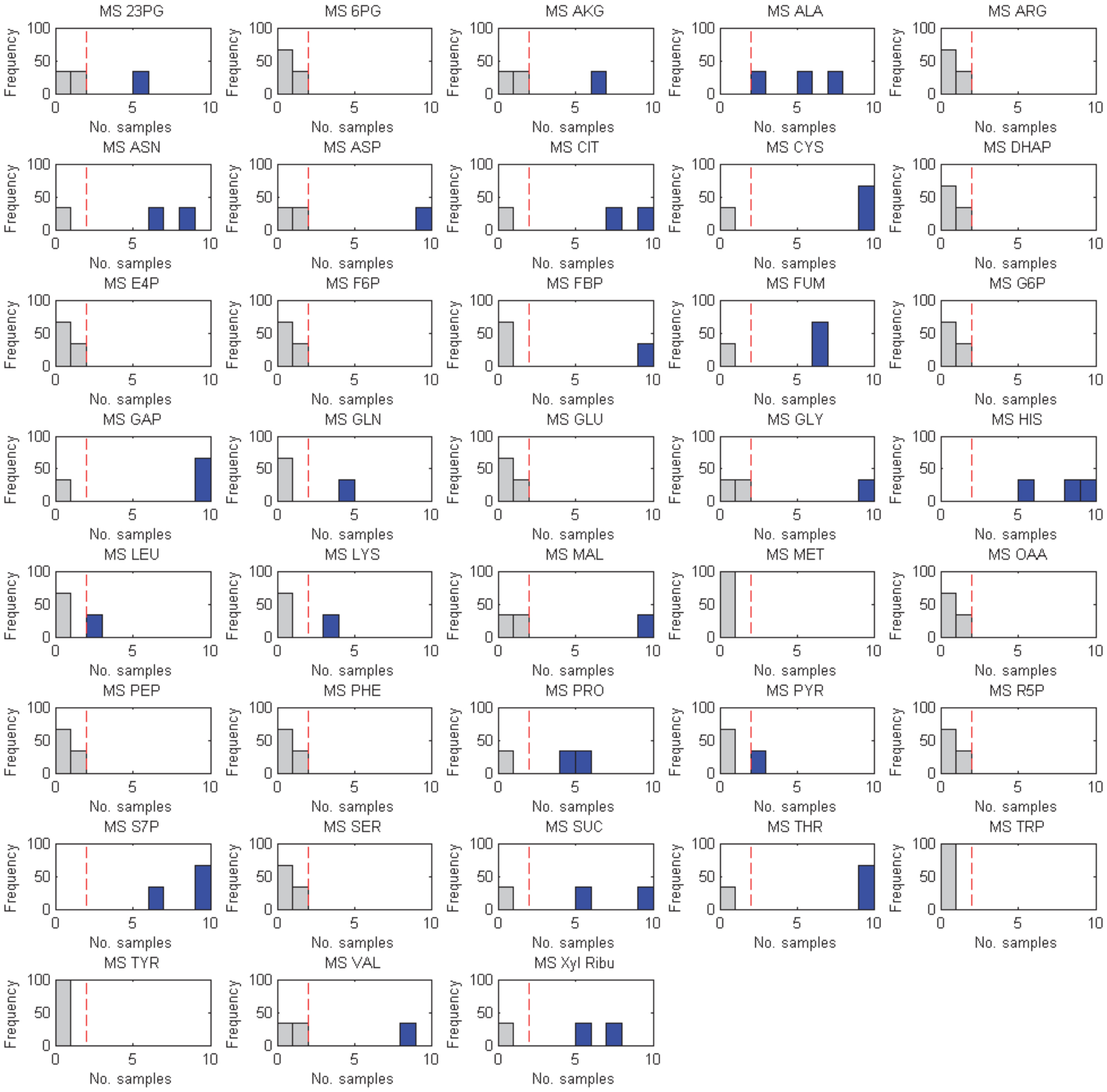

Fig X. Measurement groups and replicate numbers of the 3D-MO-ED solution for LC-MS/MS and substrate mixture cluster #4: 98.8% [^{12}C]-glucose, 1.2% [1,2-^{13}C]-glucose. Measurement groups with less than two replicates (red line) were not considered in the analysis.

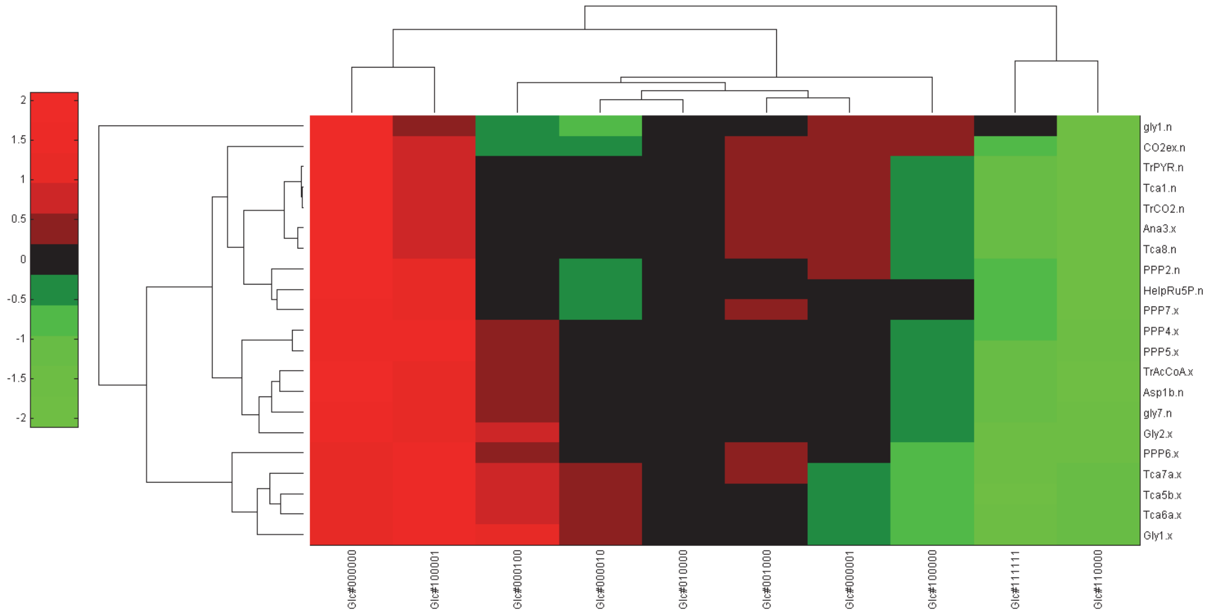

Fig Y. Clustergram of Pareto-optimal 3D-MO-ED correlations between flux STDs and input substrates for LC-MS/MS (*p*=21).

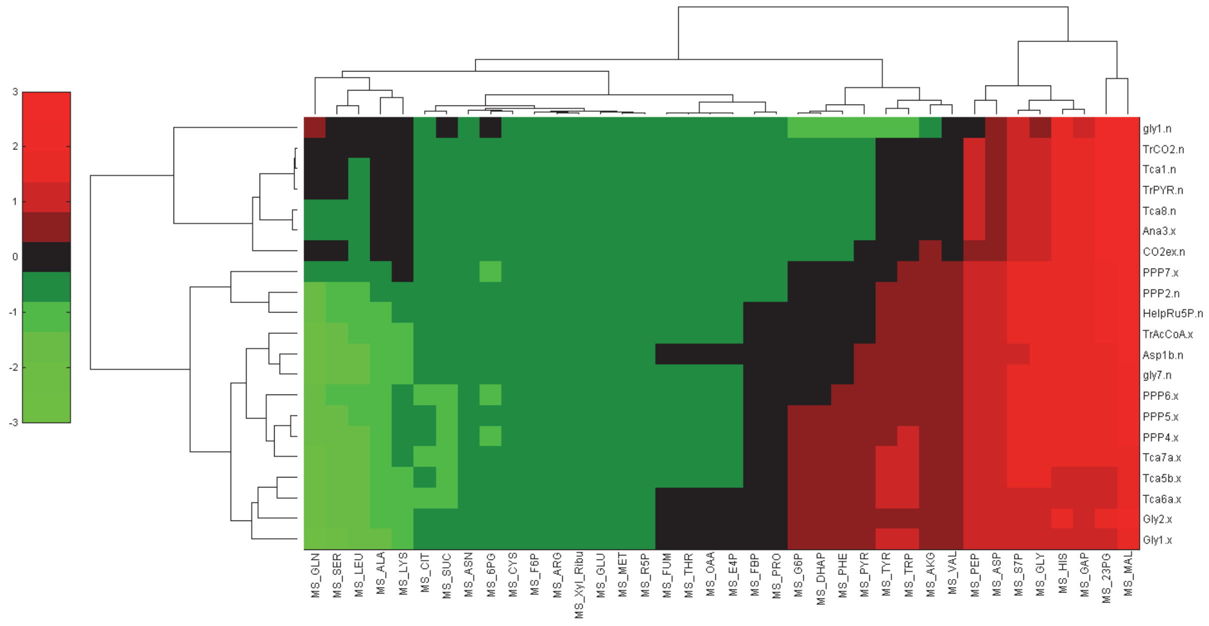

Fig Z. Clustergram of Pareto-optimal 3D-MO-ED correlations between flux STDs and measurement groups and LC-MS/MS (*p*=21).

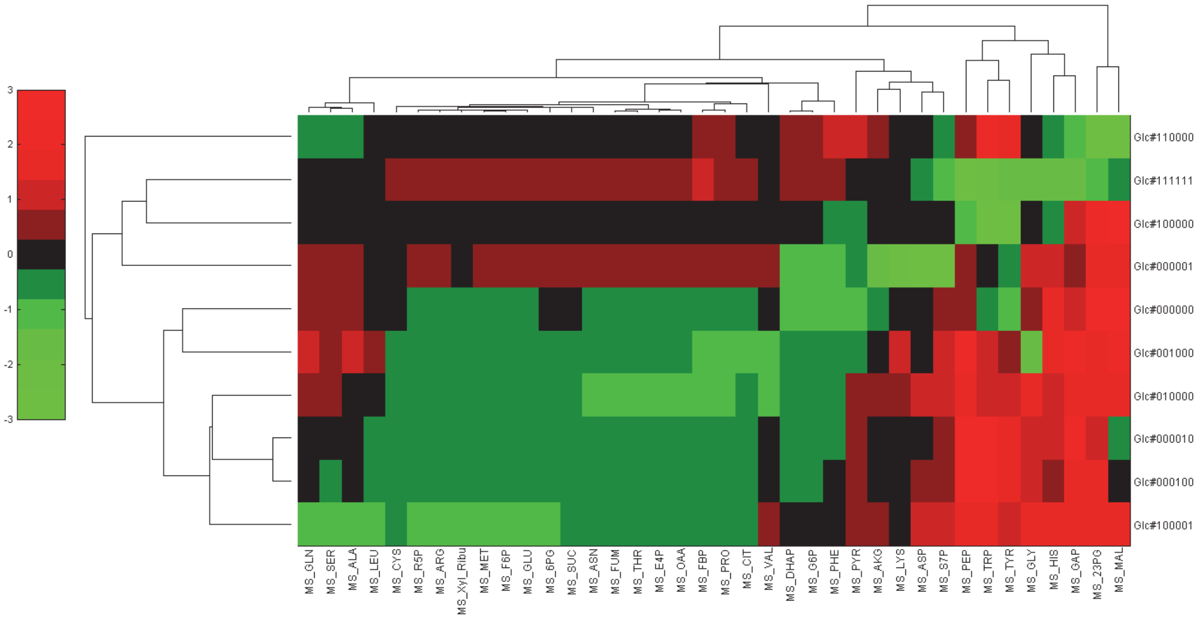

Fig AA. Clustergram of Pareto-optimal 3D-MO-ED correlations between input species and measurement groups for LC-MS/MS (*p*=21).

4. ^{13}C-NMR

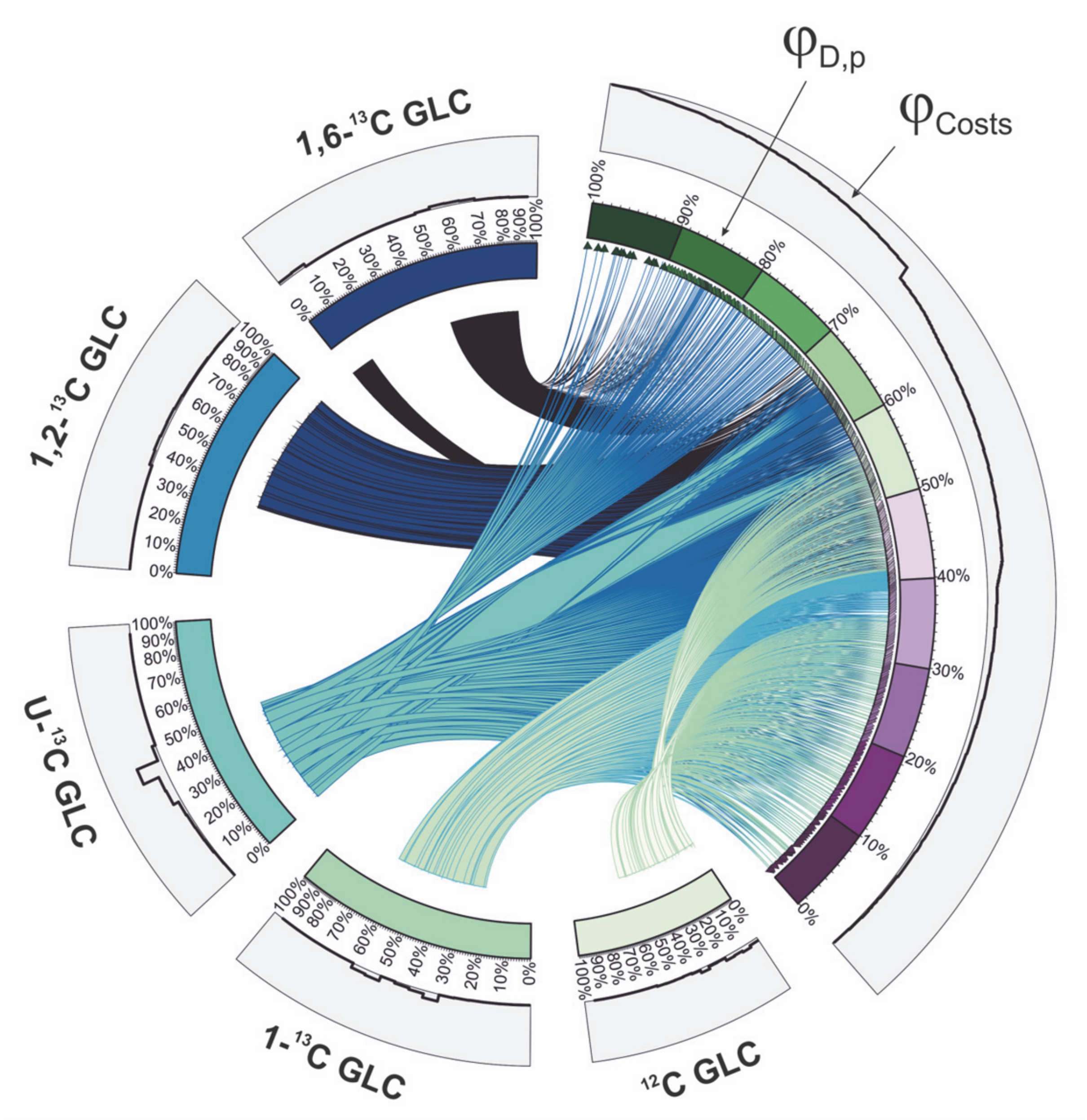

Fig AB. 3D-MO-ED ^{13}C-MFA design space for ^{13}C-NMR (p=21). Enlarged version of cord diagram shown in Fig 7 of the main text.

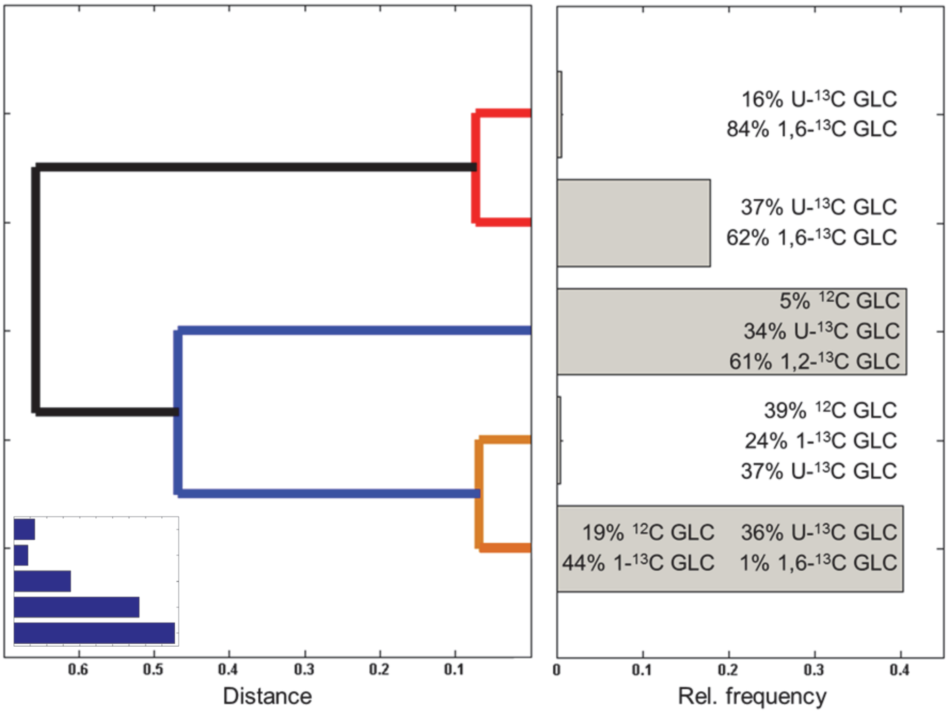

Fig AC. Dendrogram of the substrate mixture clusters for ^{13}C-NMR (p=21). The best known Pareto set of the 3D-MO-ED problem was hierarchically clustered by the minimal Euclidean distance of the labeling fraction composition. Values below 1% were omitted. The length of the edges (distance) represents the dissimilarities of the mixtures. Five mixture clusters were determined for which composition average values are given along with their relative frequency. The clusters' mean cost proportions are shown in the inset. The corresponding number of measurement groups and replicates resolved by the four clusters are given in S4 Fig AD–AH.

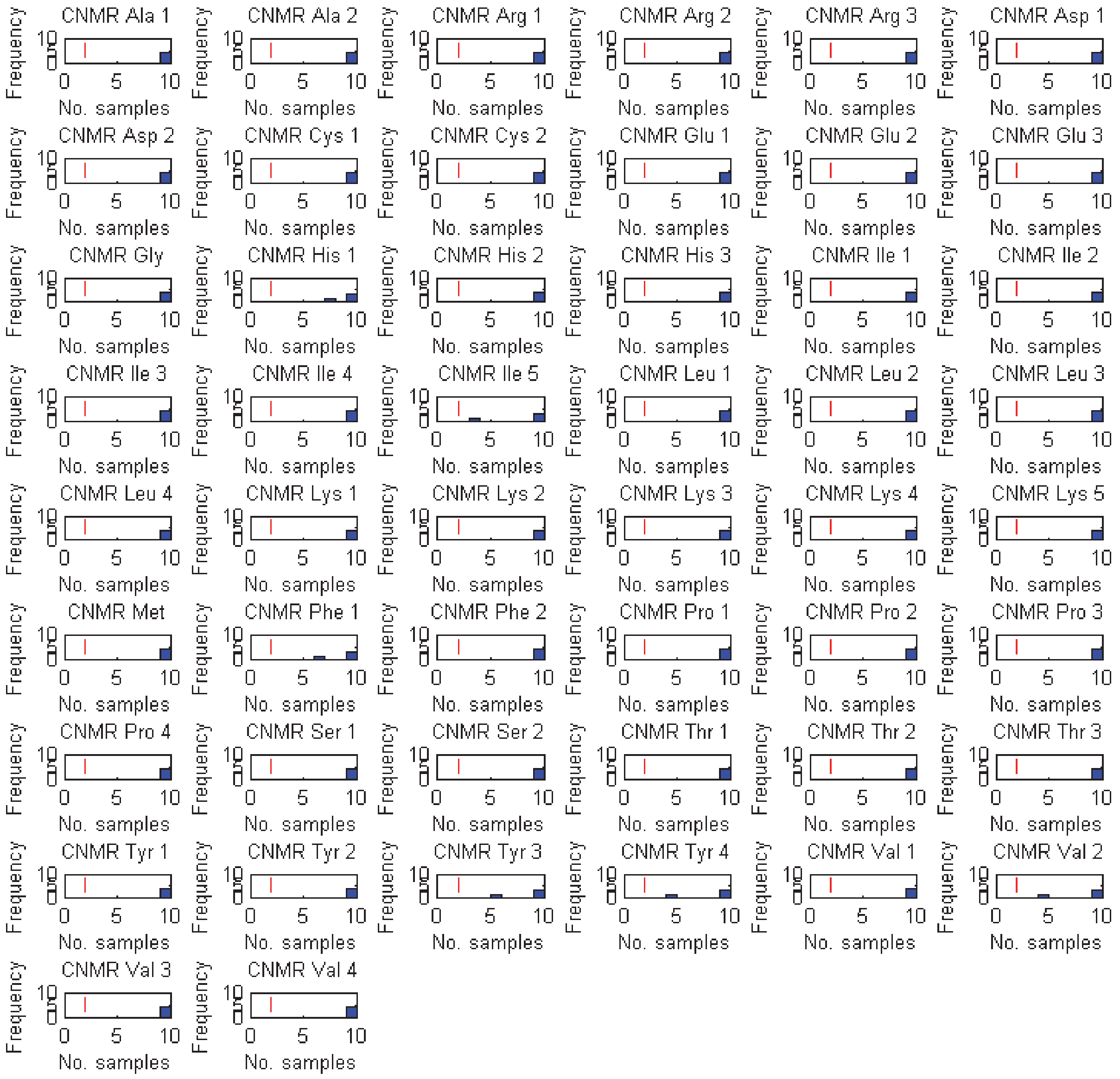

Fig AD. Measurement groups and replicate numbers of the 3D-MO-ED solution for ^{13}C-NMR and substrate mixture cluster #1: 15.78% [U-^{13}C]-glucose, 83.92% [1,6-^{13}C]-glucose (mean values). Measurement groups with less than two replicates (red line) were not considered in the analysis.

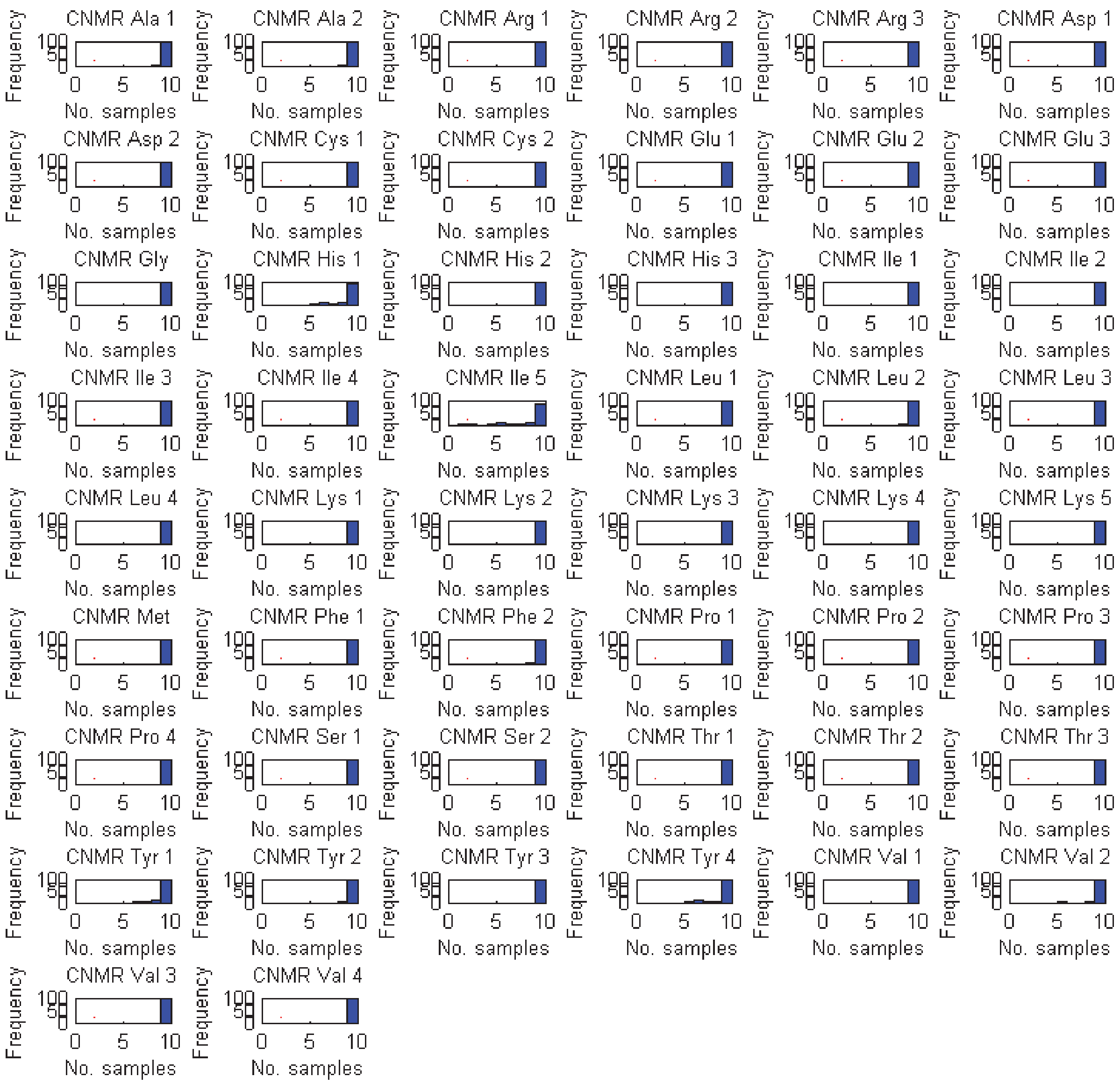

Fig AE. Measurement groups and replicate numbers of the 3D-MO-ED solution for ^{13}C-NMR and substrate mixture cluster #2: 37.45% [U-^{13}C]-glucose, 62.48% [1,6-^{13}C]-glucose (mean values). Measurement groups with less than two replicates (red line) were not considered in the analysis.

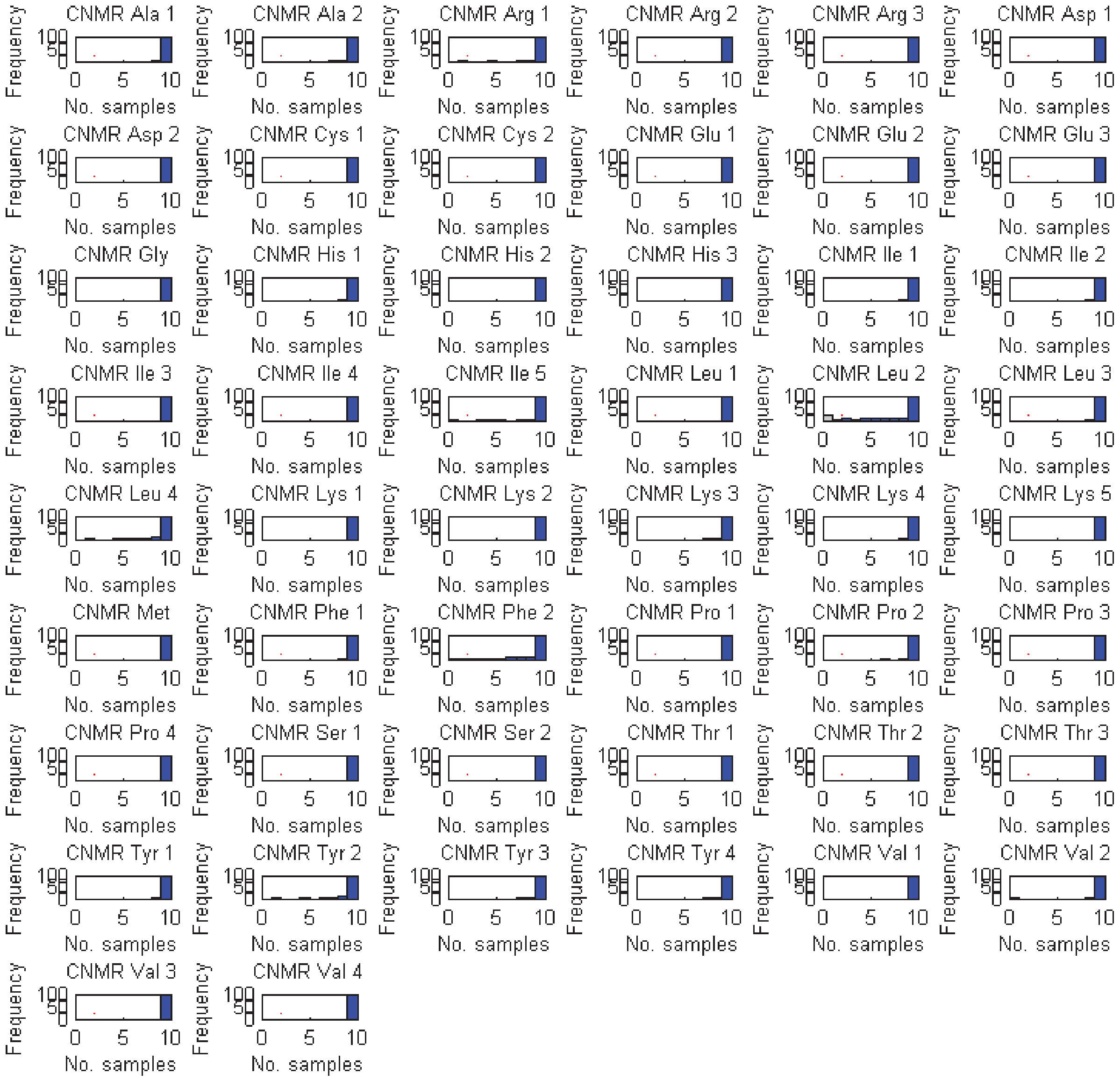

Fig AF. Measurement groups and replicate numbers of the 3D-MO-ED solution for ^{13}C-NMR and substrate mixture cluster #3: 5.31% [^{12}C]-glucose, 33.91% [U-^{13}C]-glucose, 60.74% [1,2-^{13}C]-glucose (mean values). Measurement groups with less than two replicates (red line) were not considered in the analysis.

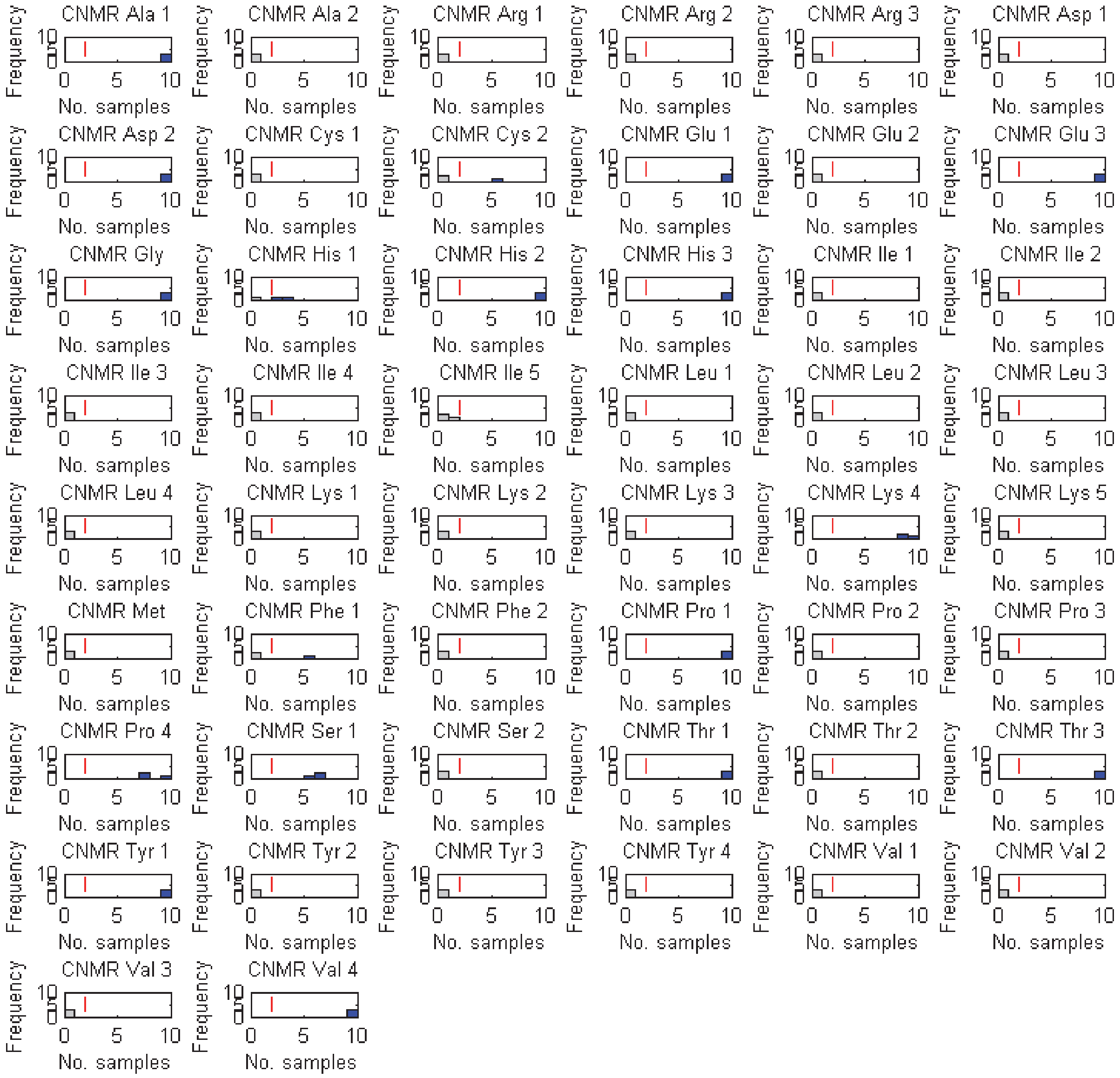

Fig AG. Measurement groups and replicate numbers of the 3D-MO-ED solution for ^{13}C-NMR and substrate mixture cluster #4: 38.63% [^{12}C]-glucose, 23.85% [1-^{13}C]-glucose, 37.48% [U-^{13}C]-glucose (mean values). Measurement groups with less than two replicates (red line) were not considered in the analysis.

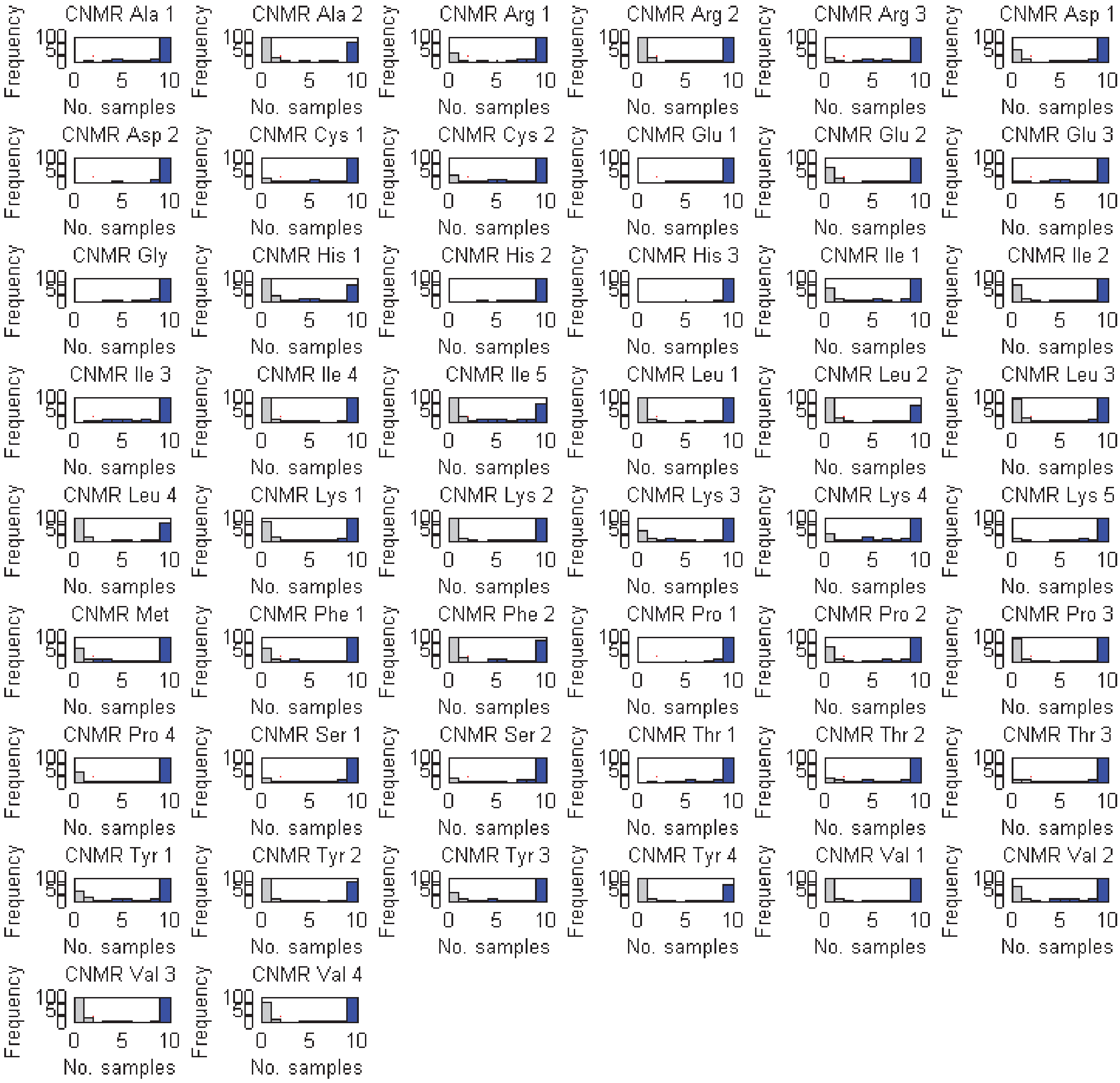

Fig AH. Measurement groups and replicate numbers of the 3D-MO-ED solution for ^{13}C-NMR and substrate mixture cluster #5: 18.62% [^{12}C]-glucose, 43.77% [1-^{13}C]-glucose, 36.28% [U-^{13}C]-glucose, 1.27% [1,6-^{13}C]-glucose (mean values). Measurement groups with less than two replicates (red line) were not considered in the analysis.

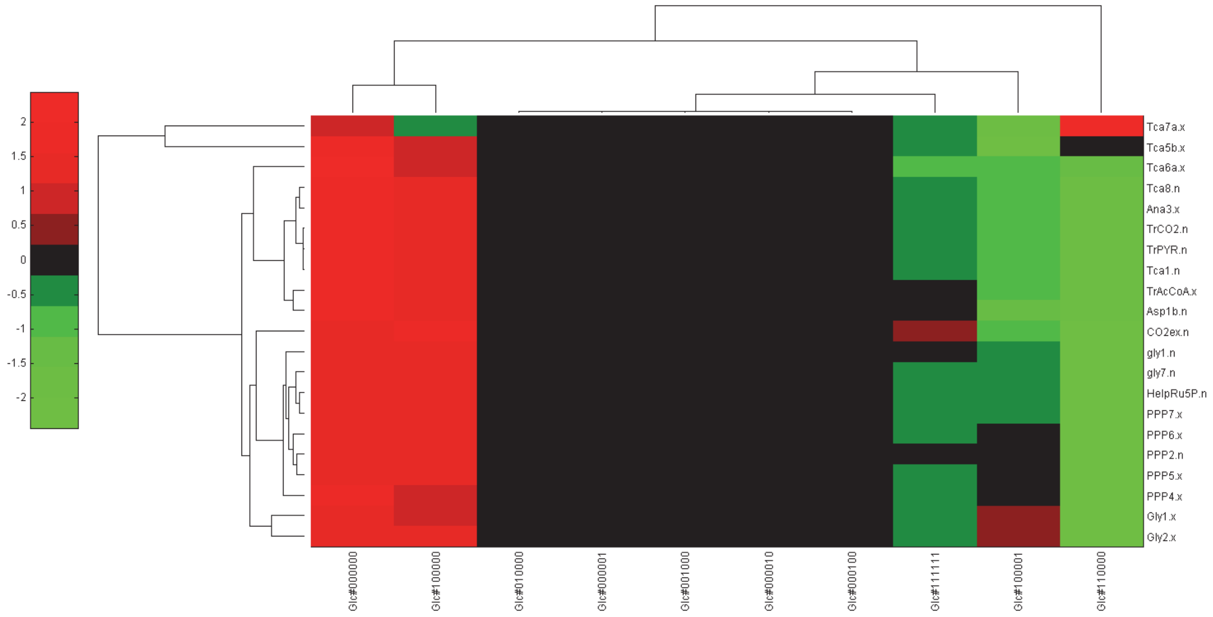

Fig AI. Clustergram of Pareto-optimal 3D-MO-ED correlations between flux STDs and input substrates for ^{13}C-NMR (p=21).

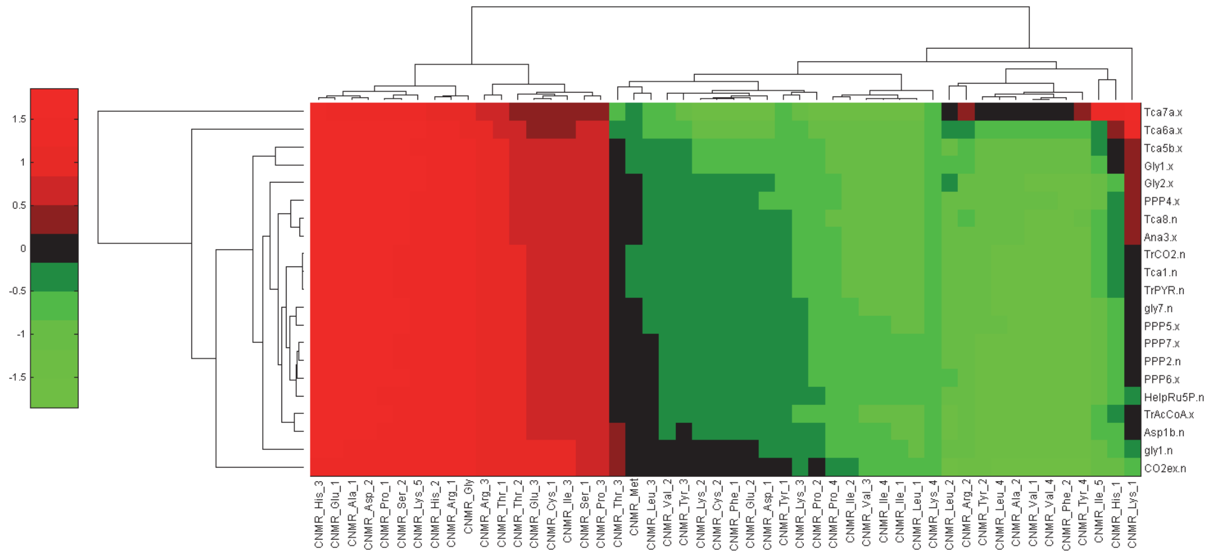

Fig AJ. Clustergram of Pareto-optimal 3D-MO-ED correlations between flux STDs and measurement groups for ^{13}C-NMR (p=21).

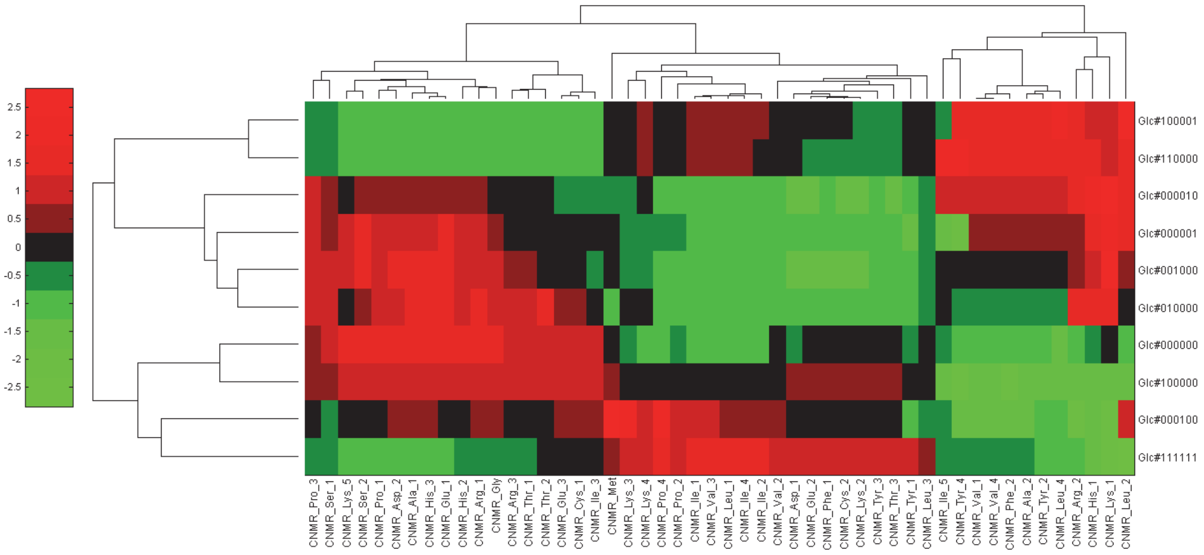

Fig AK. Clustergram of Pareto-optimal 3D-MO-ED correlations between input species and measurement groups for ^{13}C-NMR (p=21).

5. Objective space ranges

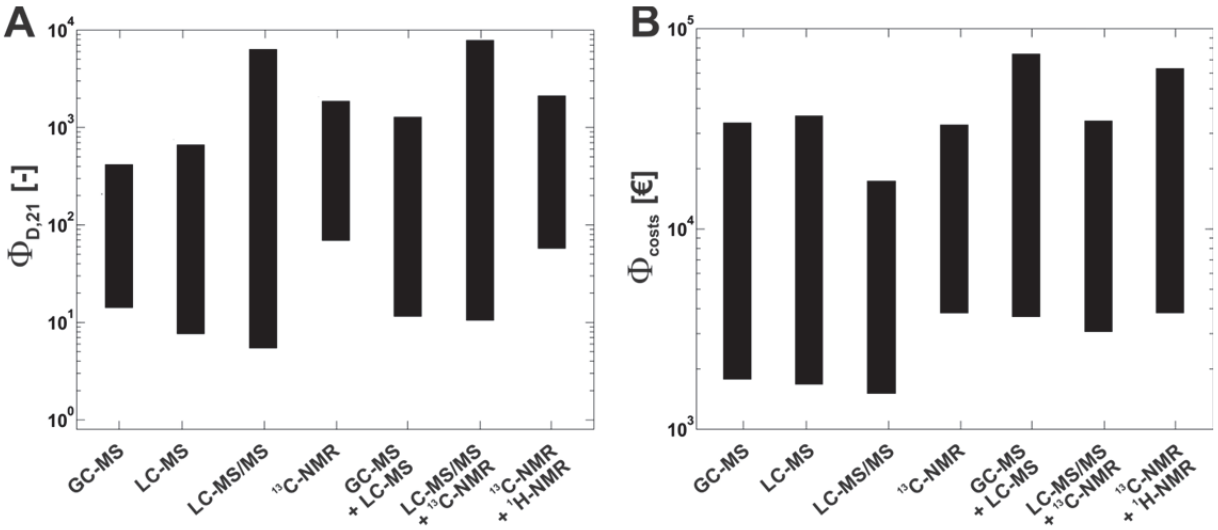

Fig AL. Ranges of Pareto-optimal D-criterion values (A) and costs (B) of the 3D-MO-ED design problem for different analytical platforms and platform combinations filtered for solutions with full model dimensionality. See also S4 Fig AM-AO for results for p<21.

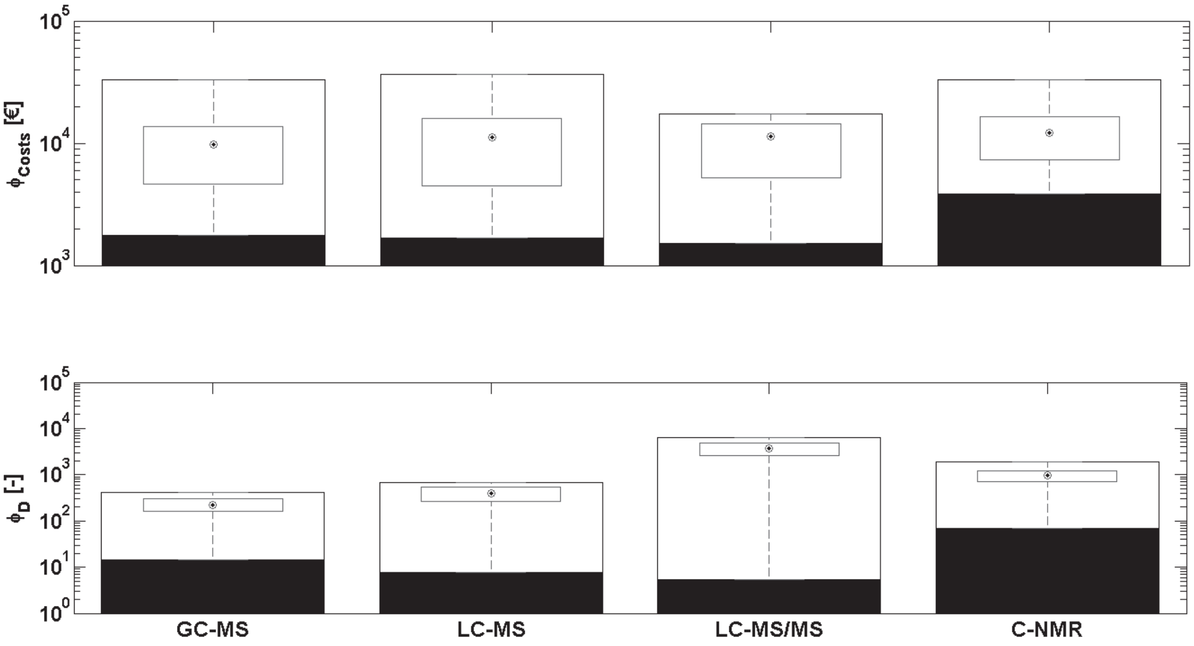

Fig AM. Ranges of 3D-MO-ED Pareto-optimal cost- and D-criterion values ($p = 21$) and different analytical platforms and platform combinations. Y-axes are log-scaled. Big white boxes indicate the minimal and maximal criterion values. Inner box plots show 25th, 75th percentiles and the median criterion values. Cardinality of Pareto-solutions: GC-MS – 431, LC-MS – 897, LC-MS/MS – 933, ^{13}C-NMR – 323. See also Fig 6 in the main text.

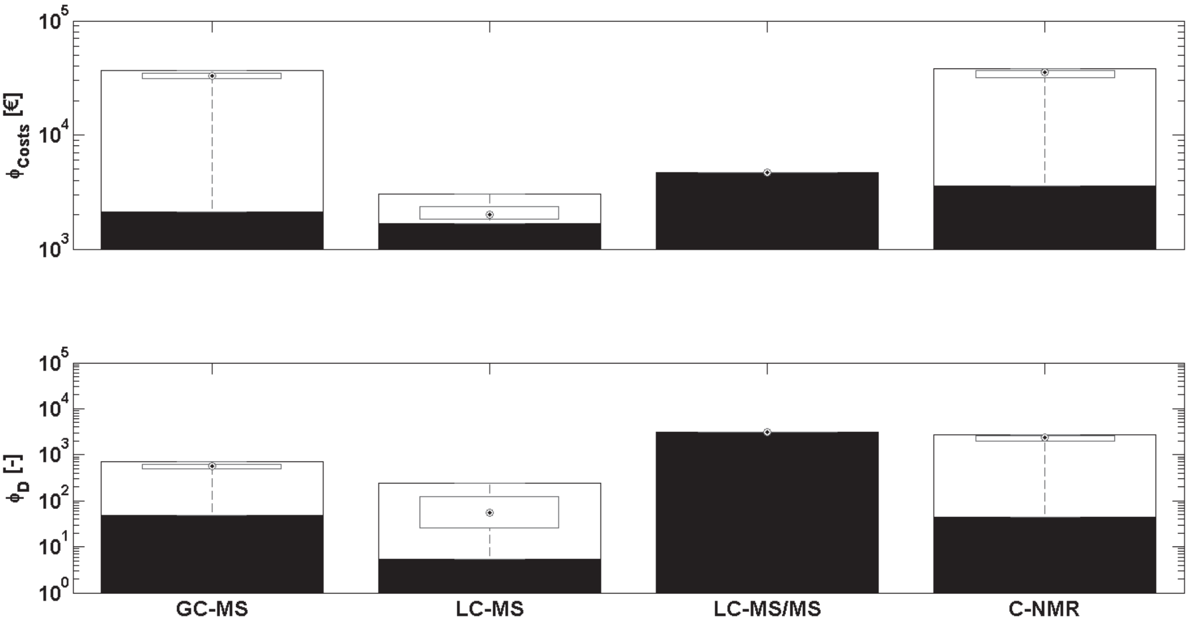

Fig AN. Ranges of 3D-MO-ED Pareto-optimal cost- and D-criterion values (p = 20) and different analytical platforms and platform combinations. Y-axes are log-scaled. Big white boxes indicate the minimal and maximal criterion values. Inner box plots show 25th, 75th percentiles and the median criterion values. Cardinality of Pareto-solutions: GC-MS – 198, LC-MS – 19, LC-MS/MS – 1, ^{13}C-NMR – 74.

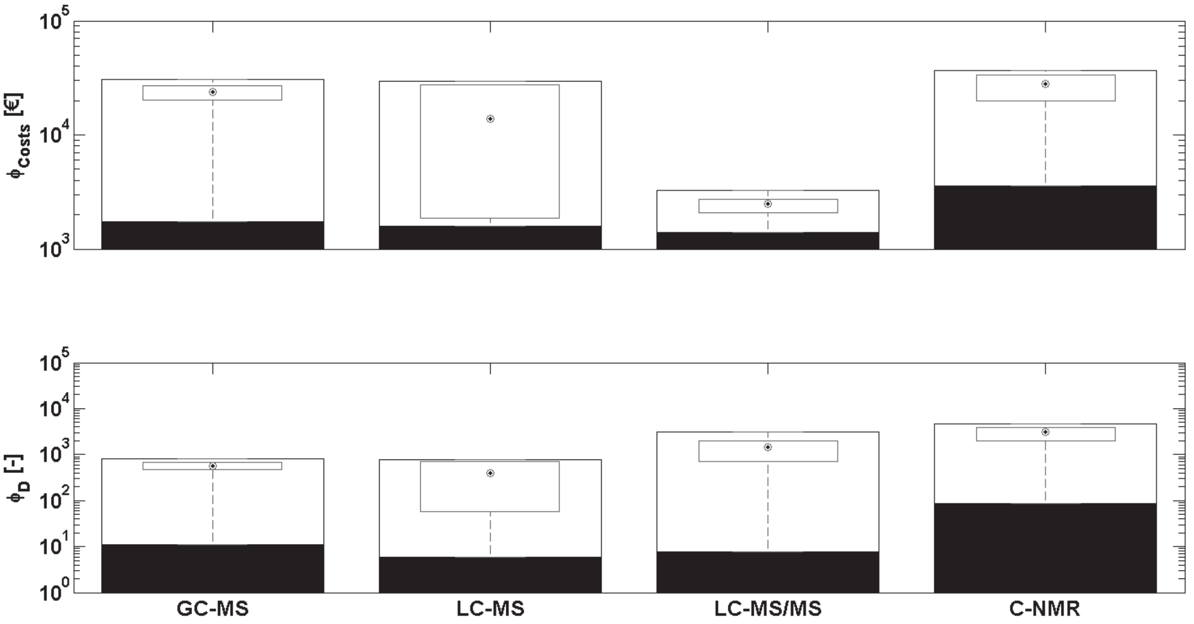

Fig AO. Ranges of 3D-MO-ED Pareto-optimal cost- and D-criterion values (p = 19) and different analytical platforms and platform combinations. Y-axes are log-scaled. Big white boxes indicate the minimal and maximal criterion values. Inner box plots show 25th, 75th percentiles and the median criterion values. Cardinality of Pareto-solutions: GC-MS – 352, LC-MS – 26, LC-MS/MS – 13, ^{13}C-NMR – 205.

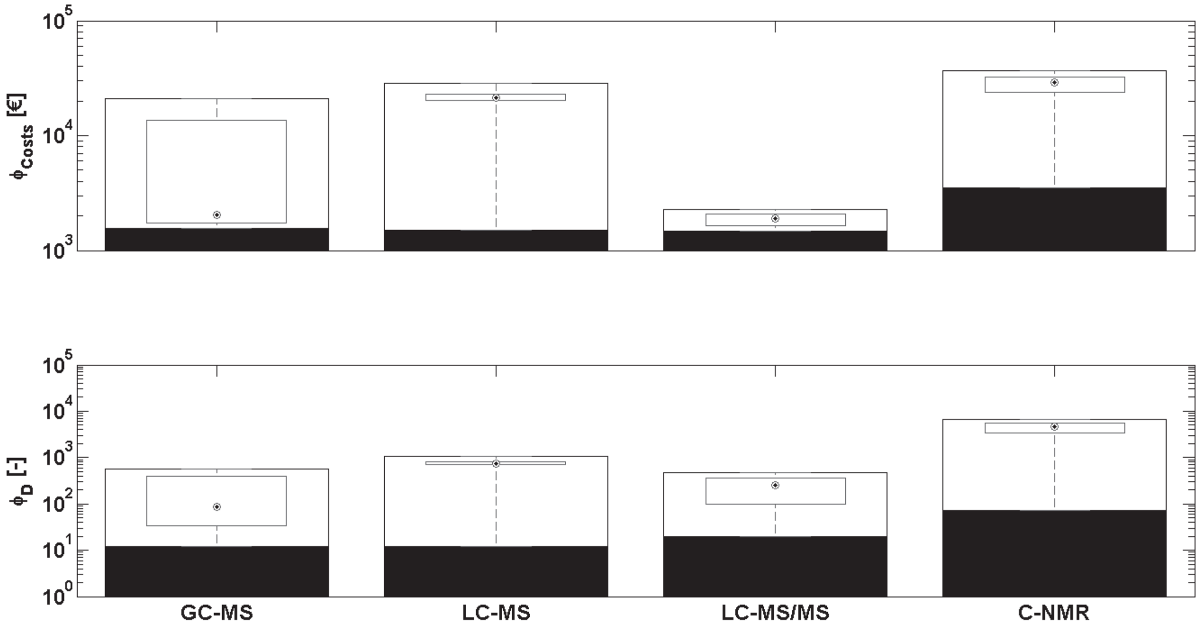

Fig AP. Ranges of 3D-MO-ED Pareto-optimal cost- and D-criterion values ($p = 18$) and different analytical platforms and platform combinations. Y-axes are log-scaled. Big white boxes indicate the minimal and maximal criterion values. Inner box plots show 25th, 75th percentiles and the median criterion values. Cardinality of Pareto-solutions: GC-MS – 19, LC-MS – 58, LC-MS/MS – 51, ^{13}C-NMR – 398.

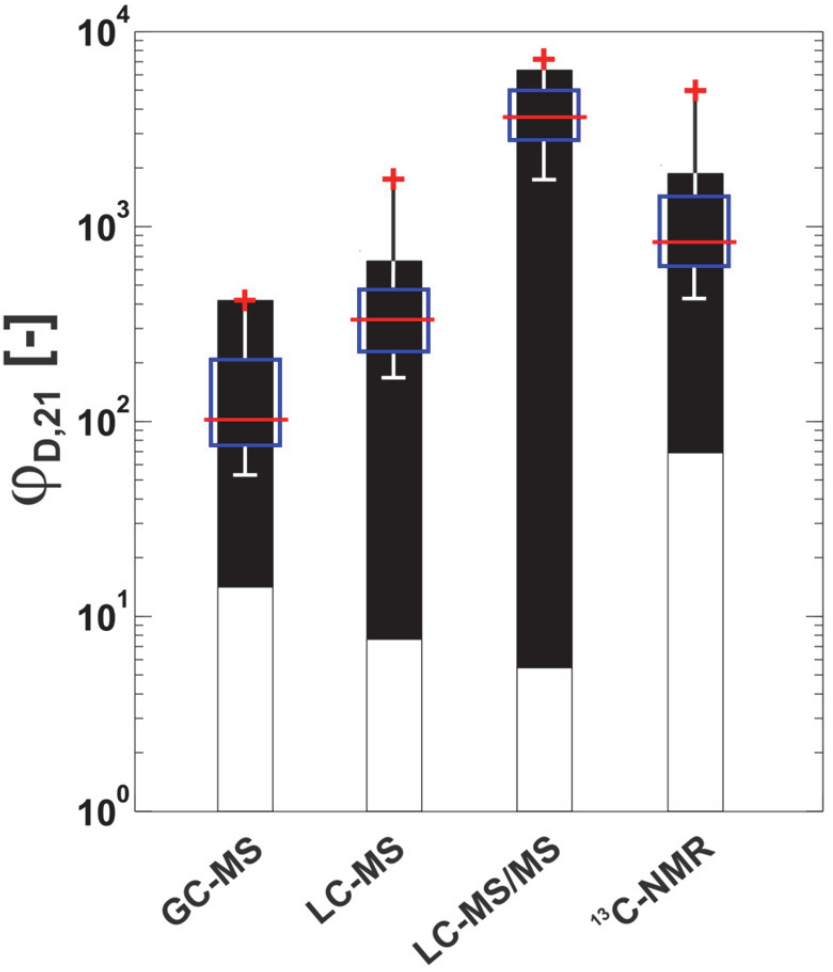

Fig AQ. Box plots of the ranges of D-criterion values emerging from 10,000 randomly sampled flux distributions. Samples are taken in the vicinity of the reference flux distribution in the bounding box of the confidence ellipsoid of the in each case maximally informative substrate composition: GC-MS - 11.2% [U-^{13}C]-glucose, 88.7% [1,6-^{13}C]-glucose, 0.1% [2-^{13}C]-glucose, LC-MS - 100% [1,6-^{13}C]-glucose, LC-MS/MS - 100% [1,2-^{13}C]-glucose, ^{13}C-NMR - 16.5% [U-^{13}C]-glucose, 83.5% [1,6-^{13}C]-glucose. Black bars show ranges of Pareto-optimal D criterion values of the 3D-MO-ED problem with models of dimensionality *p*=21 (see also Fig 6 in the main text). Shown are 25th, 75th percentiles (blue) and the median criterion values. Whiskers denote lowest and highest D-criterion values of the samples. The y-axis is log-scaled.

6. Multi-platform applications

6.1. ^{1}H-NMR + ^{13}C-NMR

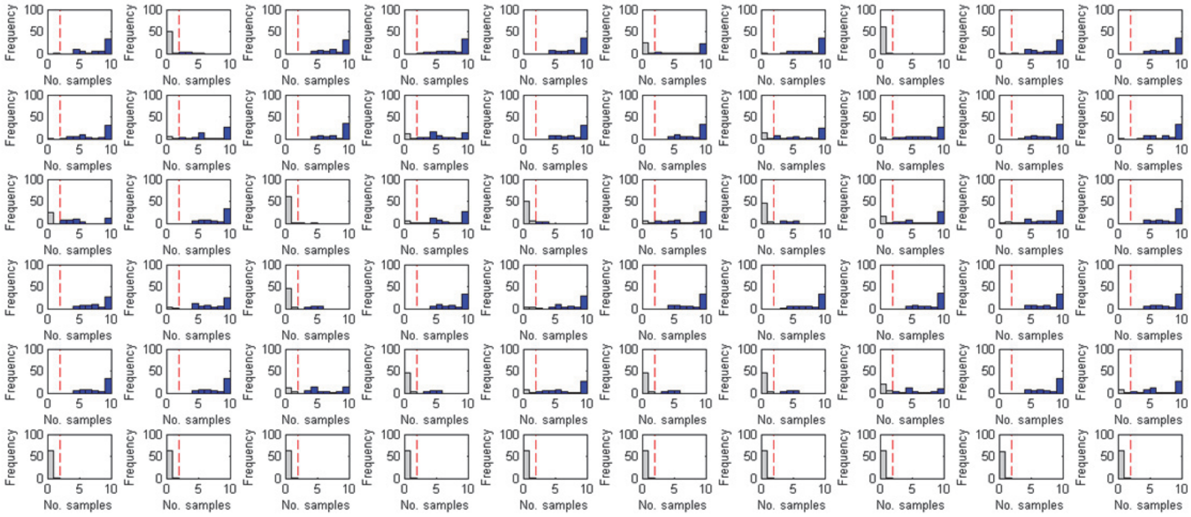

Fig AR. Measurement groups and replicate numbers of the 3D-MO-ED solution for combined ^{1}H-NMR and ^{13}C-NMR analyses. Substrate mixture cluster #1: 5% [^{12}C]-glucose, 33% [U-^{13}C]-glucose, 62% [1,2-^{13}C]-glucose (mean values). Measurement groups 1-50 and 51-60 refer to ^{13}C-NMR and ^{1}H-NMR, respectively.

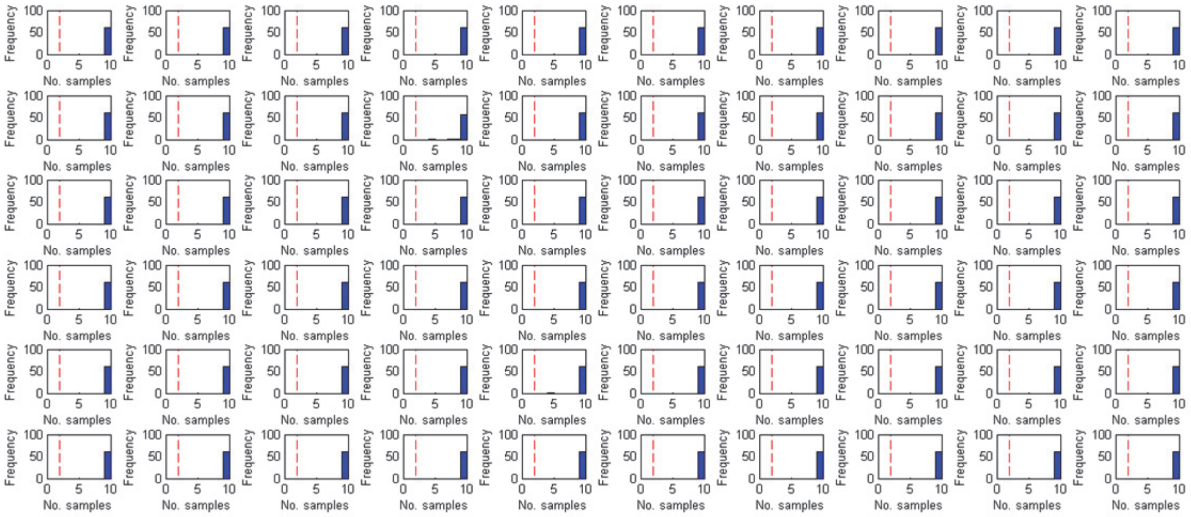

Fig AS. Measurement groups and replicate numbers of the 3D-MO-ED solution for combined ^{1}H-NMR and ^{13}C-NMR analyses. Substrate mixture cluster #2: 29% [U-^{13}C]-glucose, 71% [1,6-^{13}C]-glucose (mean values). Measurement groups 1-50 and 51-60 refer to ^{13}C-NMR and ^{1}H-NMR, respectively.

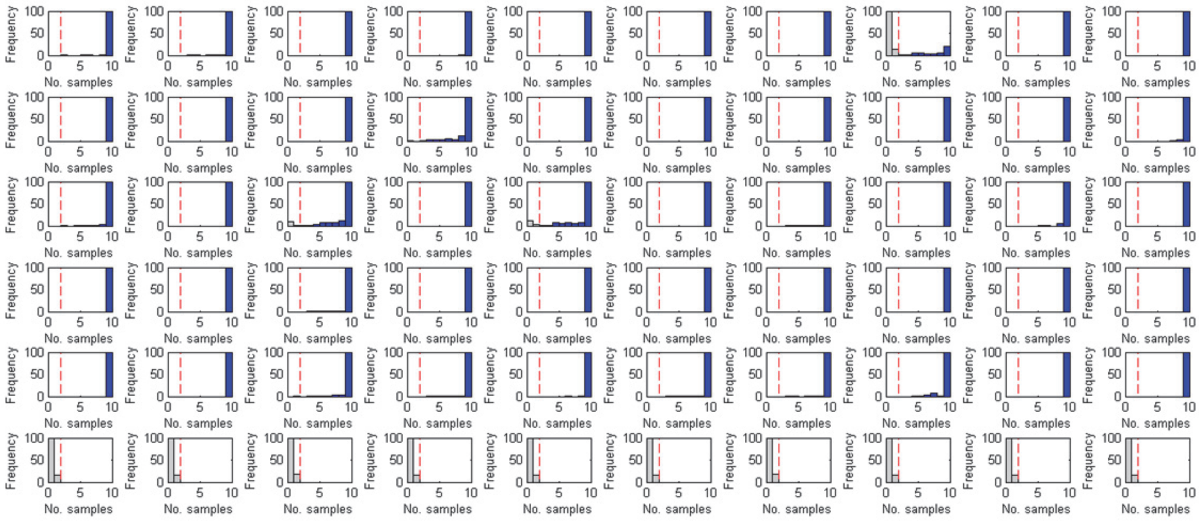

Fig AT. Measurement groups and replicate numbers of the 3D-MO-ED solution for combined ^{1}H-NMR and ^{13}C-NMR analyses. Substrate mixture cluster #3: 21% [^{12}C]-glucose, 43% [1-^{13}C]-glucose, 35% [U-^{13}C]-glucose, 1% [1,6-^{13}C]-glucose (mean values). Measurement groups 1-50 and 51-60 refer to ^{13}C-NMR and ^{1}H-NMR, respectively.

6.2. GC-MS + LC-MS

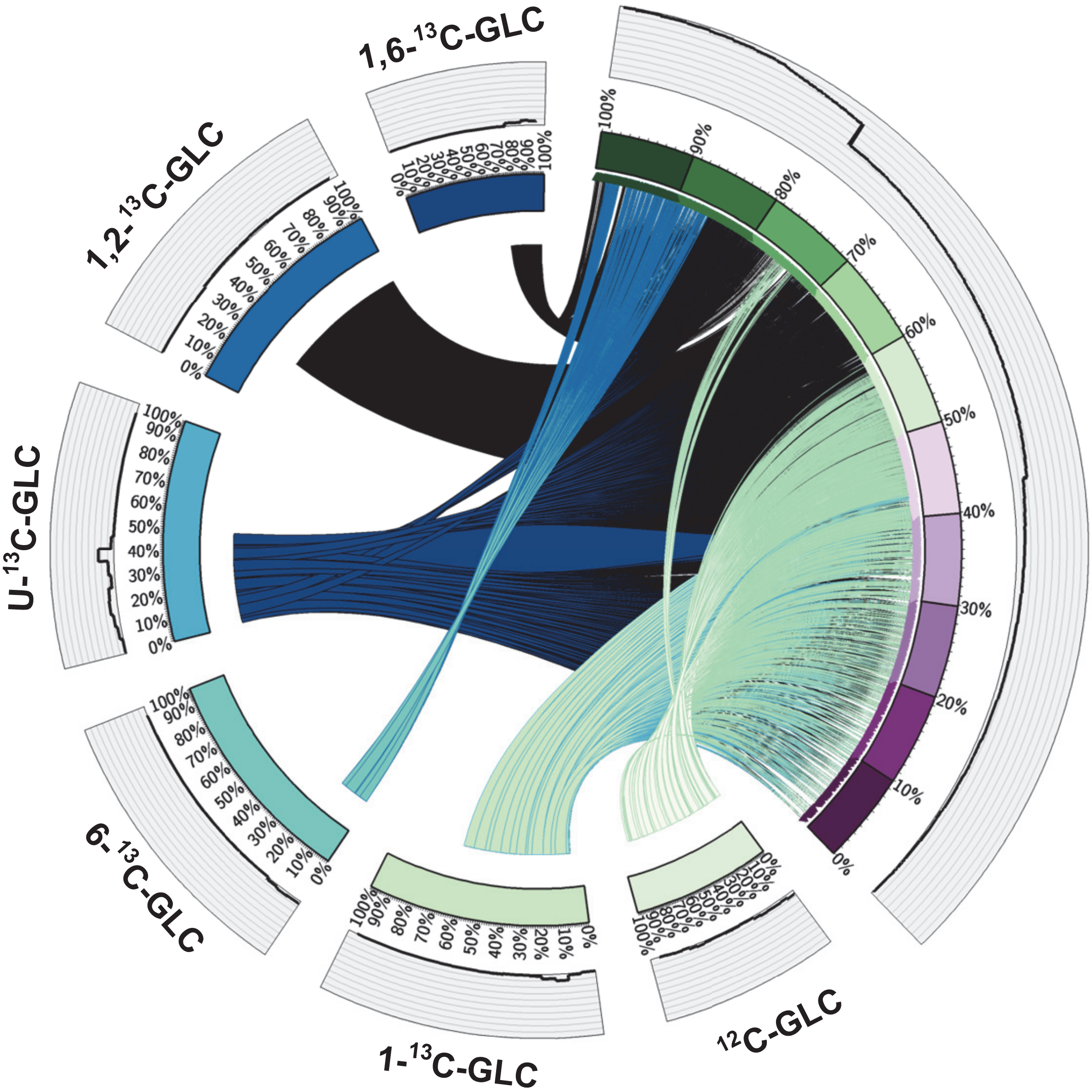

Fig. AU. Chord diagram of 3D-MO-ED ^{13}C-MFA design and objective spaces for LC-MS and GC-MS (p=21).

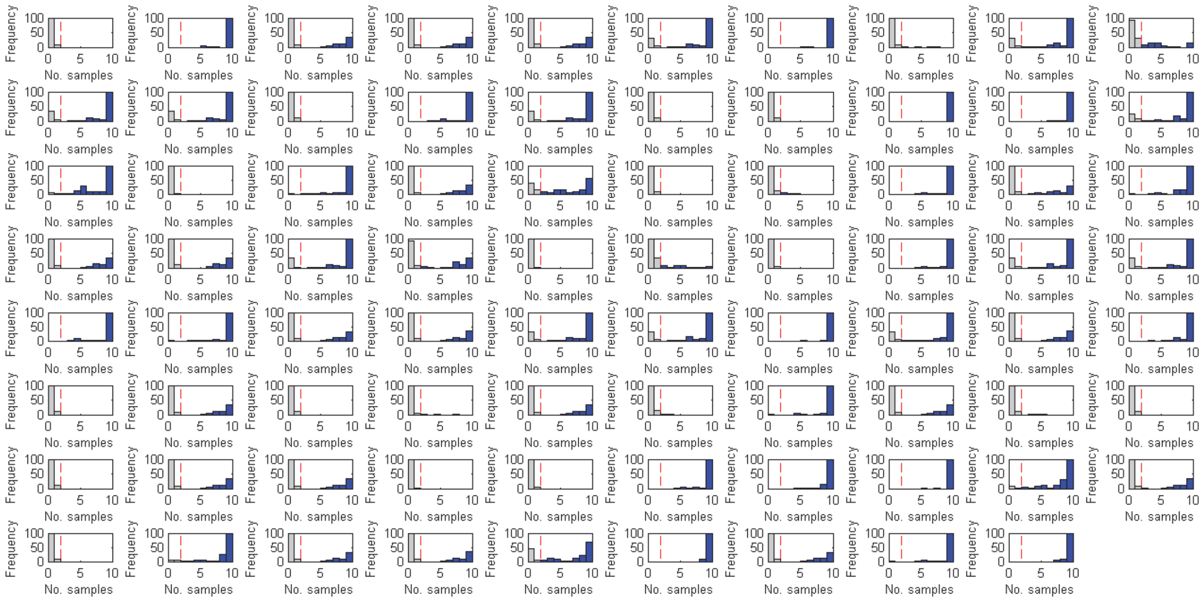

Fig AV. Measurement groups and replicate numbers of the 3D-MO-ED solution for combined GC-MS and LC-MS analyses. Substrate mixture cluster #1: 51% [^{12}C]-glucose, 21% [1-^{13}C]-glucose, 28% [U-^{13}C]-glucose (mean values).

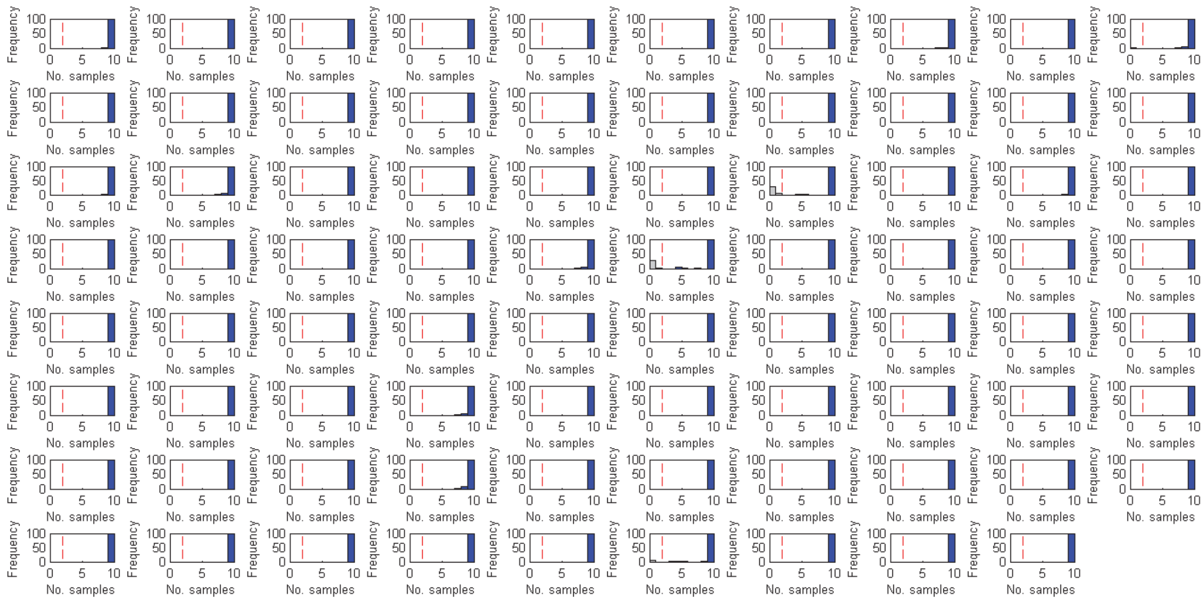

Fig AW. Measurement groups and replicate numbers of the 3D-MO-ED solution for combined GC-MS and LC-MS analyses. Substrate mixture cluster #2: 7% [6-^{13}C]-glucose, 93% [1,6-^{13}C]-glucose (mean values).

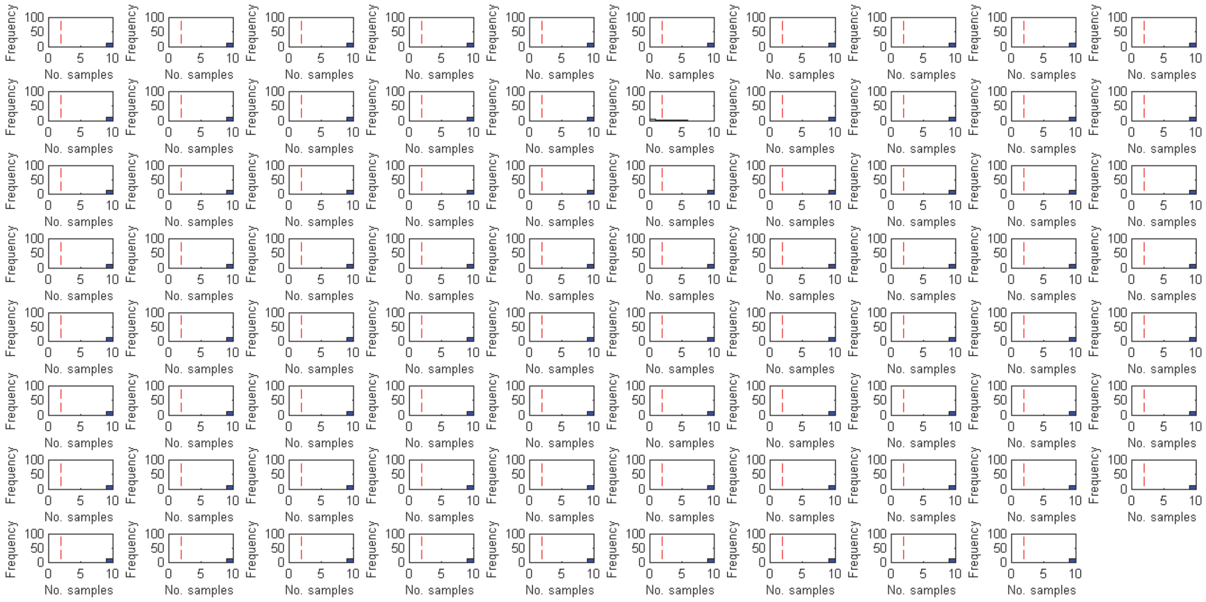

Fig AX. Measurement groups and replicate numbers of the 3D-MO-ED solution for combined GC-MS and LC-MS analyses. Substrate mixture cluster #3: 8% [^{12}C]-glucose, 31% [U-^{13}C]-glucose, 61% [1,2-^{13}C]-glucose (mean values).

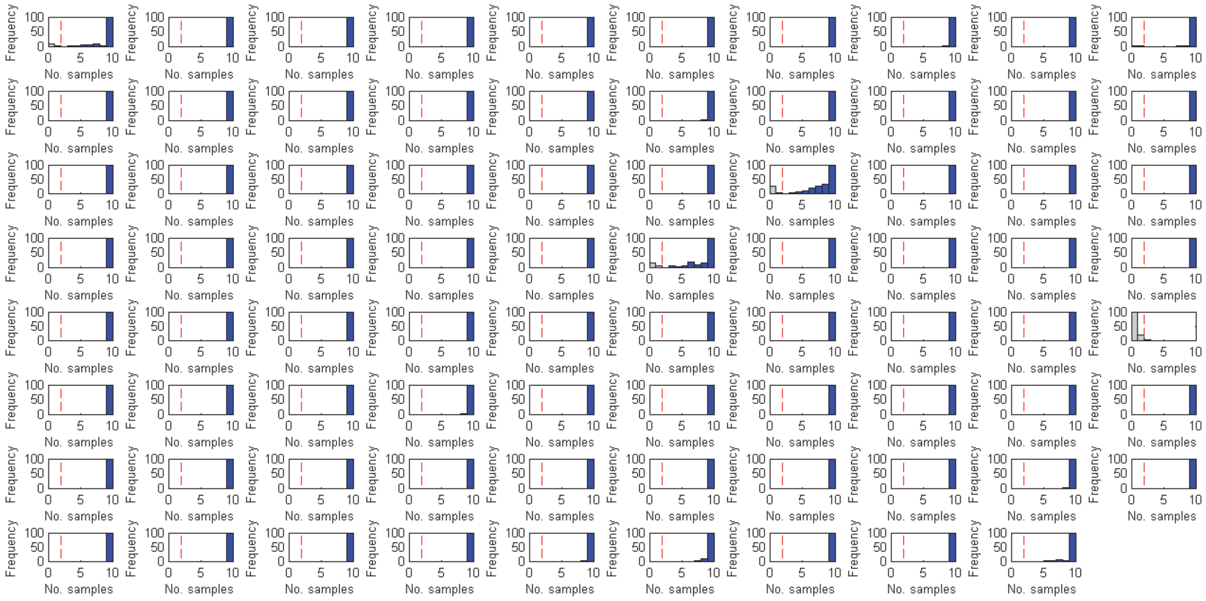

Fig AY. Measurement groups and replicate numbers of the 3D-MO-ED solution for combined GC-MS and LC-MS analyses. Substrate mixture cluster #4: 17% [U-^{13}C]-glucose, 83% [1,6-^{13}C]-glucose (mean values).

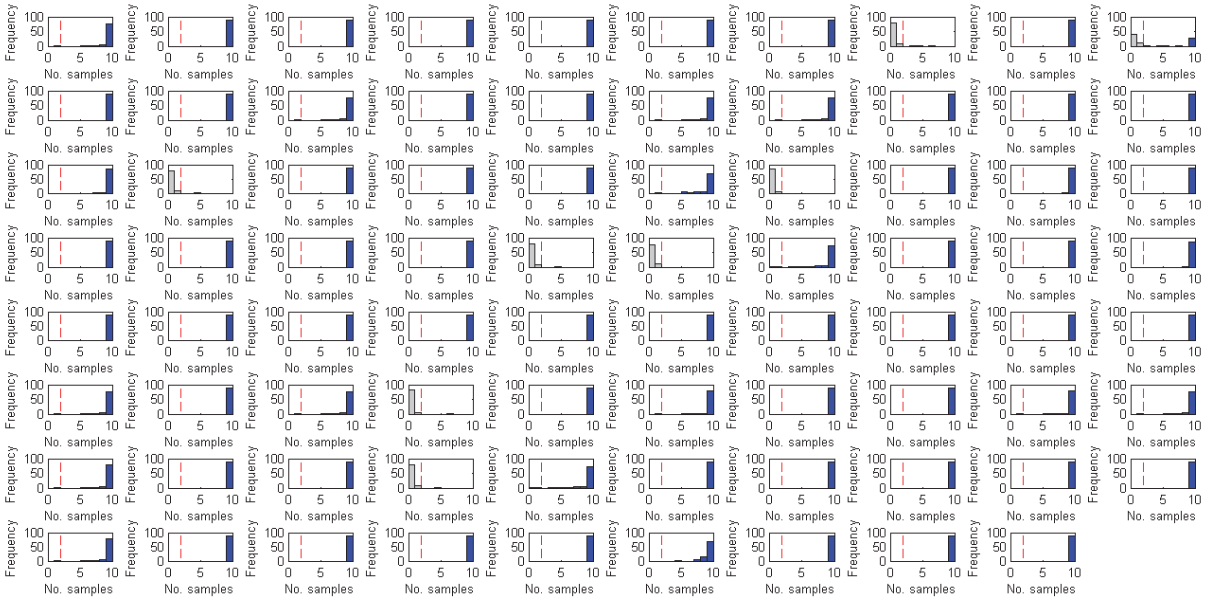

Fig AZ. Measurement groups and replicate numbers of the 3D-MO-ED solution for combined GC-MS and LC-MS analyses. Substrate mixture cluster #5: 2% [^{12}C]-glucose, 98% [1,2-^{13}C]-glucose (mean values).

6.3. LC-MS/MS + ^{13}C-NMR

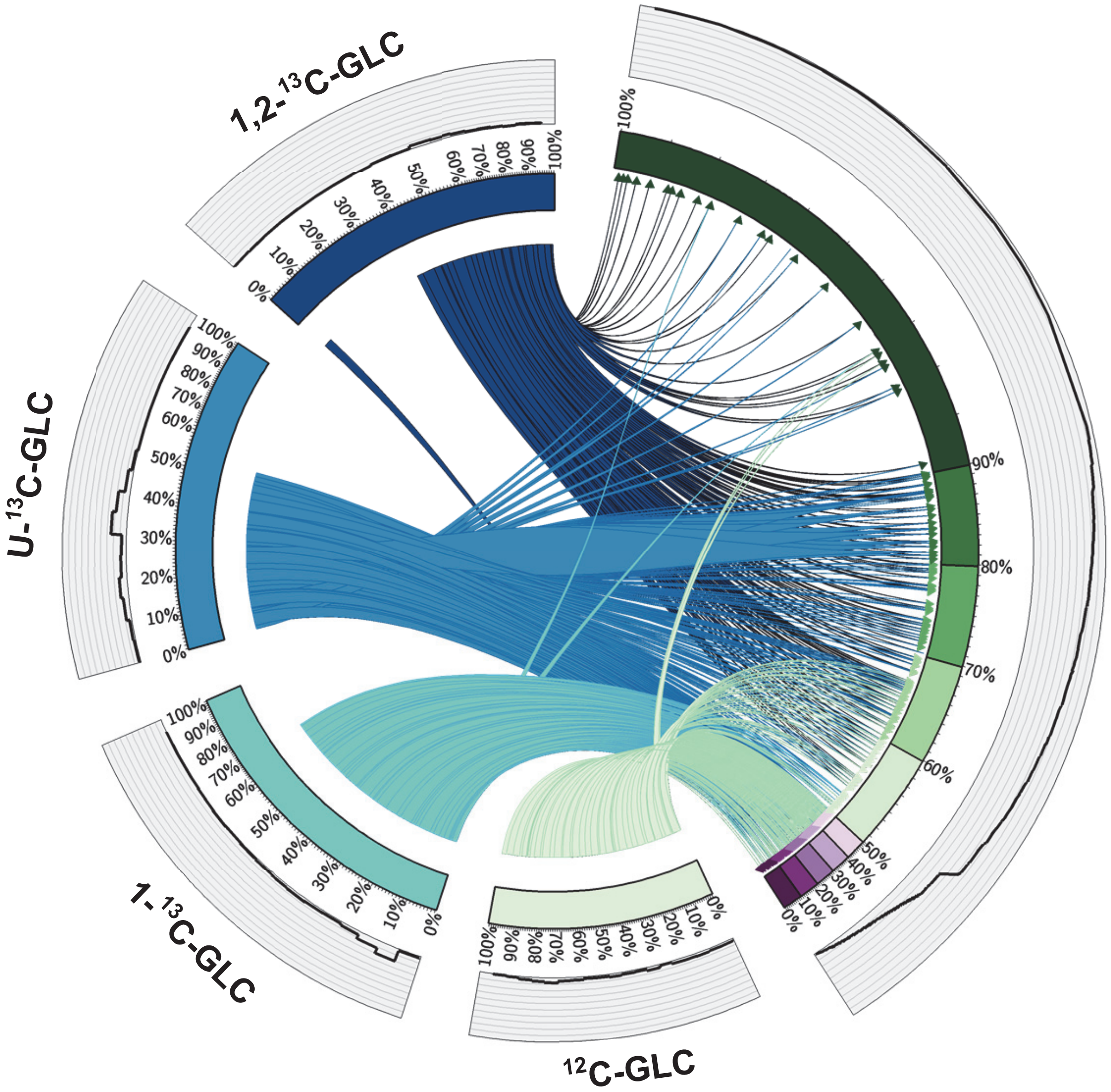

Fig BA. Chord diagram of 3D-MO-ED ^{13}C-MFA design and objective spaces for LC-MS/MS and ^{13}C-NMR (p=21).

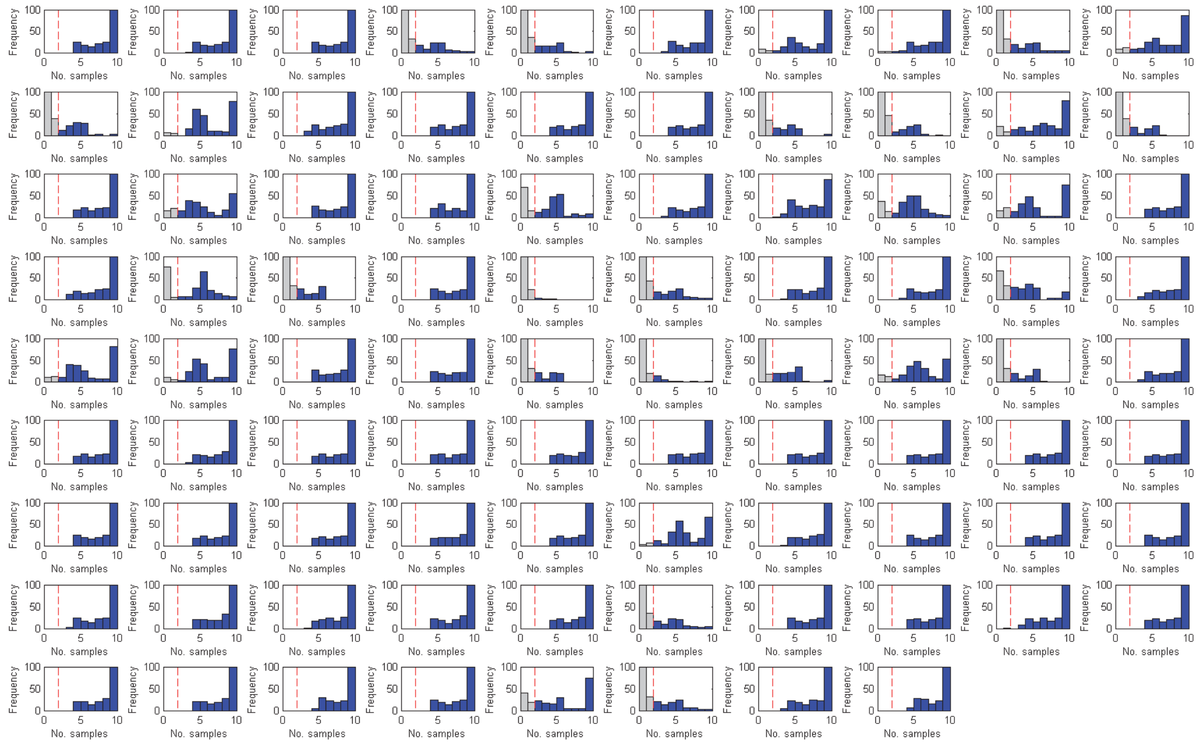

Fig BB. Measurement groups and replicate numbers of the 3D-MO-ED solution for combined LC-MS/MS and ^{13}C-NMR analyses. Substrate mixture cluster #1: 52% [^{12}C]-glucose, 21% [1-^{13}C]-glucose, 27% [U-^{13}C]-glucose (mean values).

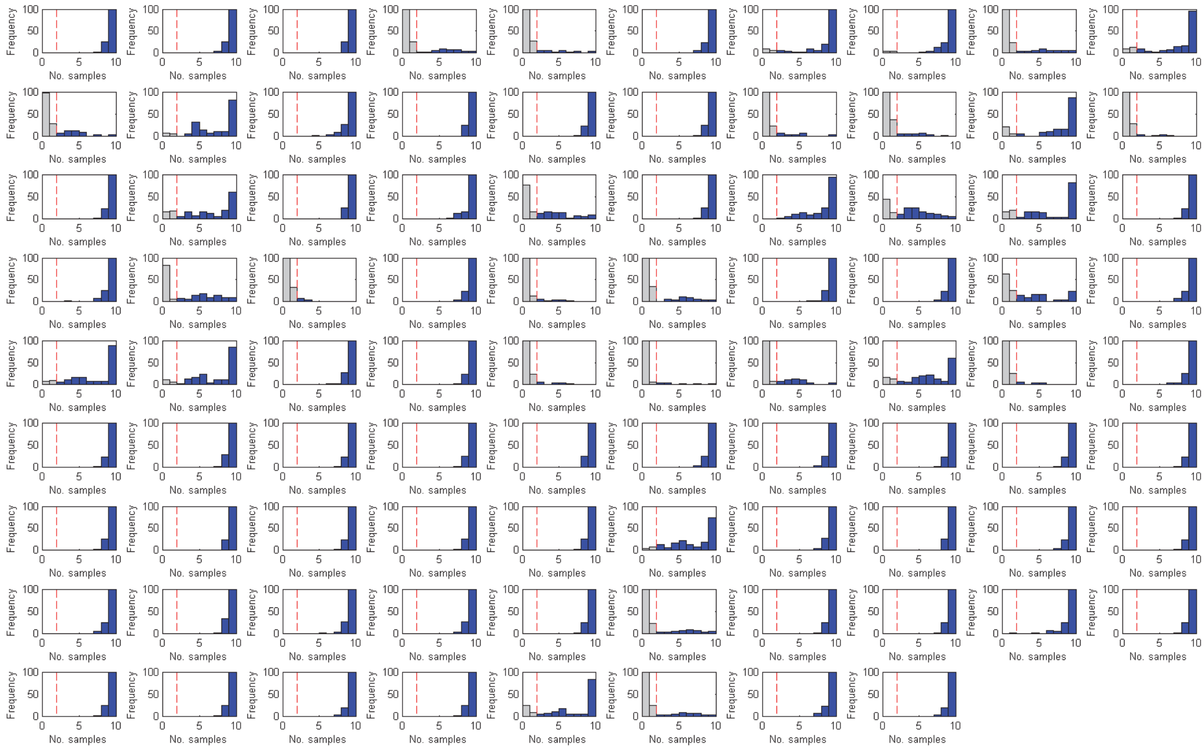

Fig BC. Measurement groups and replicate numbers of the 3D-MO-ED solution for combined LC-MS/MS and ^{13}C-NMR analyses. Substrate mixture cluster #2: 7% [^{12}C]-glucose, 30% [U-^{13}C]-glucose, 63% [1,2-^{13}C]-glucose (mean values).

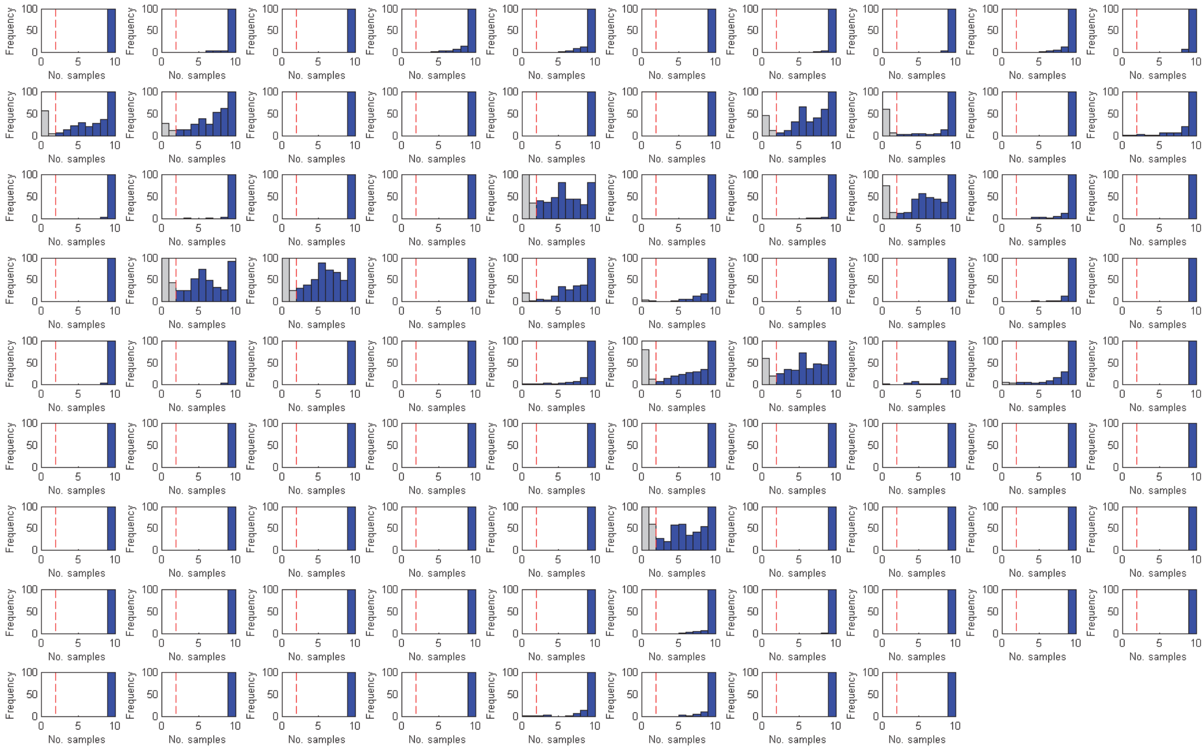

Fig BD Measurement groups and replicate numbers of the 3D-MO-ED solution for combined LC-MS/MS and ^{13}C-NMR analyses. Substrate mixture cluster #3: 2% [^{12}C]-glucose, 98% [1,2-^{13}C]-glucose (mean values).
