# Supplementary material for: A Pareto approach to resolve the conflict between information gain and experimental costs: Multiple-criteria design of carbon labeling experiments
Source: PLoS Comput Biol. 2018 Oct 31;14(10):e1006533. doi: 10.1371/journal.pcbi.1006533 (PMC6209137; doi:10.1371/journal.pcbi.1006533)
Supplement: S5 Text — Documentation of results for the analytical platforms GC-MS, LC-MS, LC-MS/MS, 13C-NMR. (PDF) [file pcbi.1006533.s005.pdf]

Supplementary Appendix 5 for

A Pareto approach to resolve the conflict between information gain and experimental costs: Multiple-criteria design of carbon labeling experiments

Multi-objective experimental design: Results for the 5D scenario

Katharina Nöh, Sebastian Niedenführ, Martin Beyß, Wolfgang Wiechert

k.noeh@fz-juelich.de

Contents

1. GC-MS 2
2. LC-MS 4
3. LC-MS/MS 6
4. ^{13}C-NMR 14

1. GC-MS

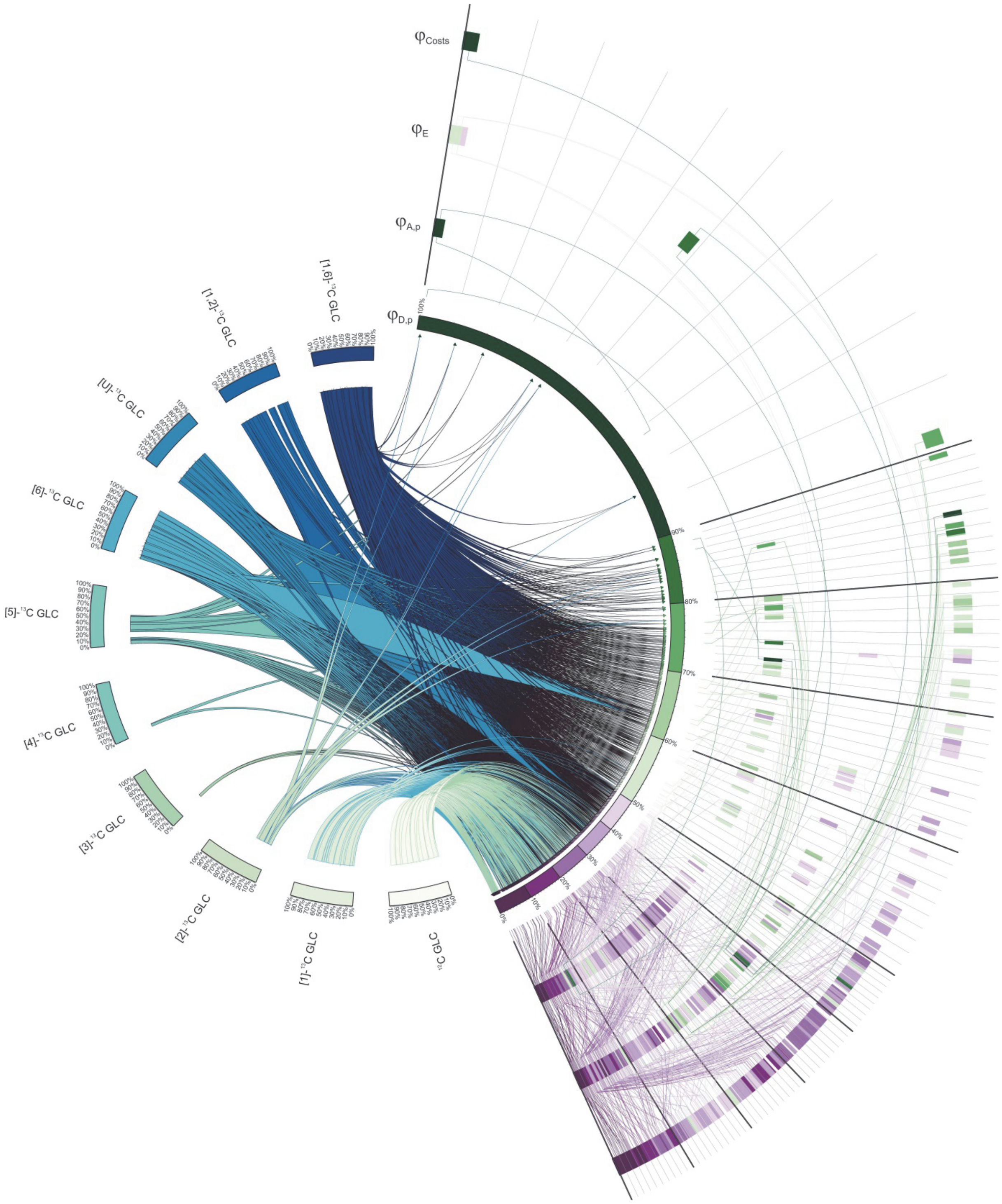

Fig A. 5D multi-objective optimization results for GC-MS (*p*=21). Cord diagram showing design and objective spaces.

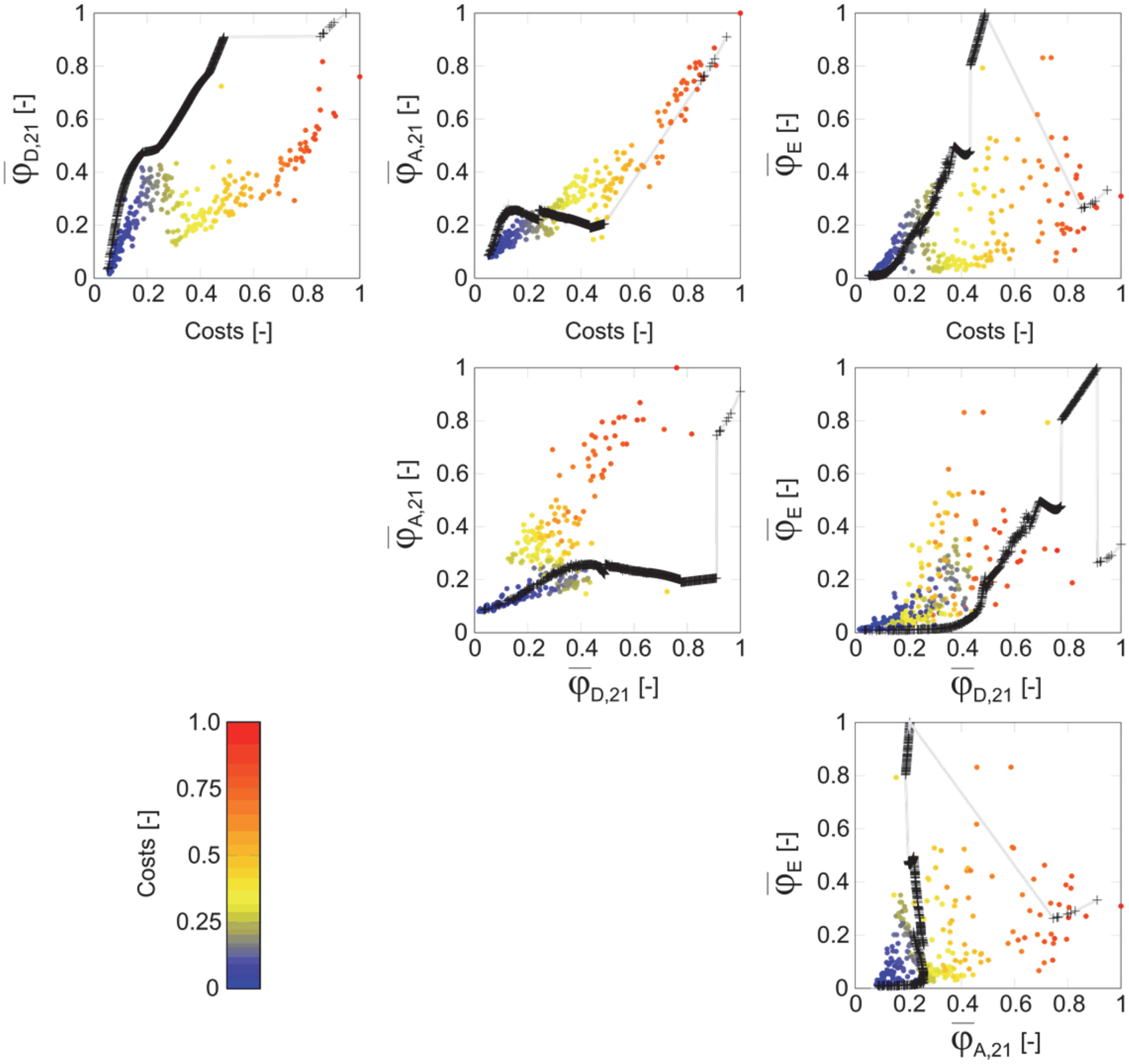

Fig B. Multi-objective optimization results for GC-MS (*p*=21). Calculated Pareto front for the 5D-MO-ED problem (objectives: D-, A-, E-criteria, dimensions, and costs). Color codes for costs of design point. Black plus signs indicate the results of the 3D-MO-ED (objectives: D-criterion, dimensions, and costs). All criteria are scaled to [0,1].

2. LC-MS

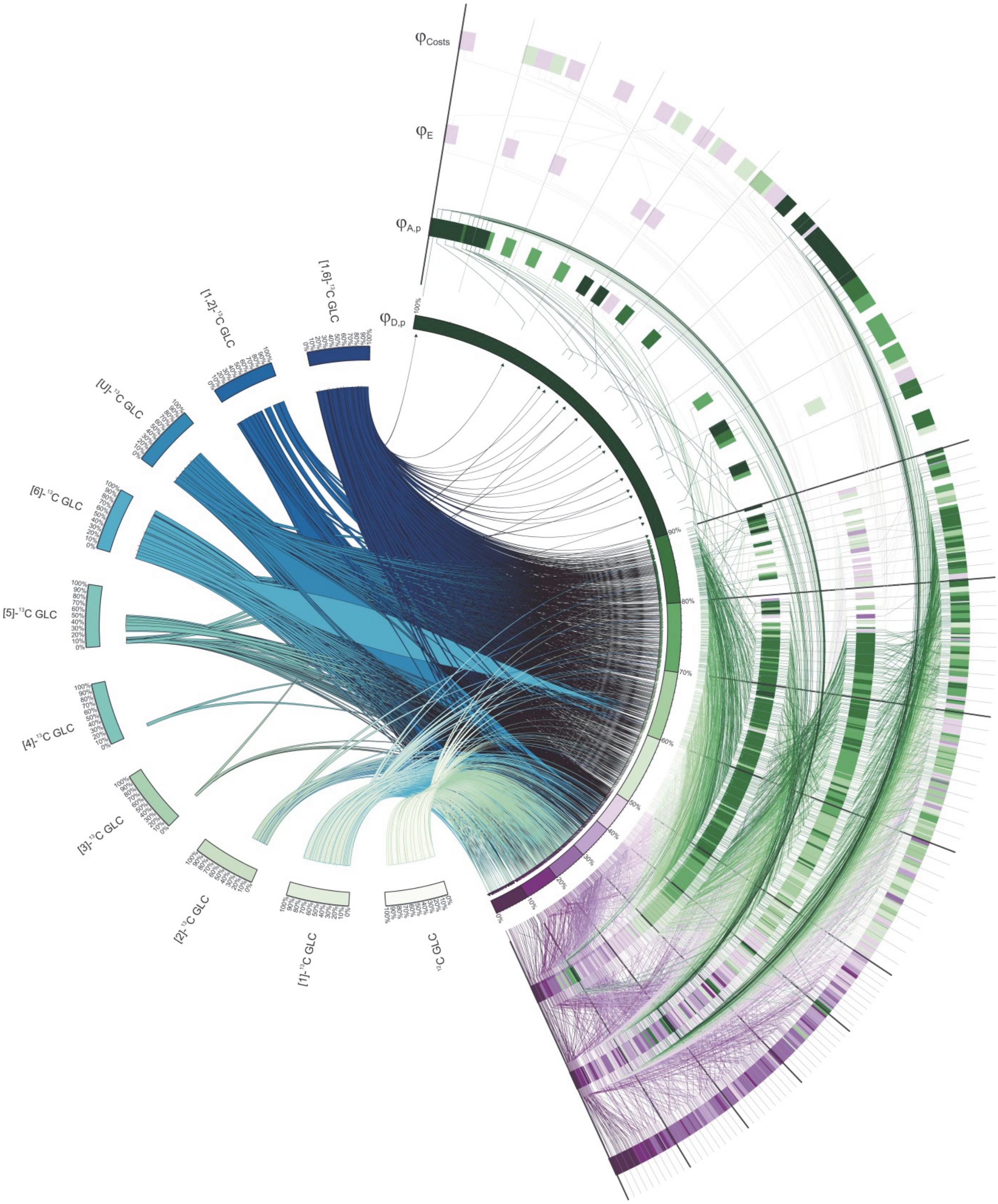

Fig C. 5D multi-objective optimization results for LC-MS (p=21). Cord diagram showing design and objective spaces.

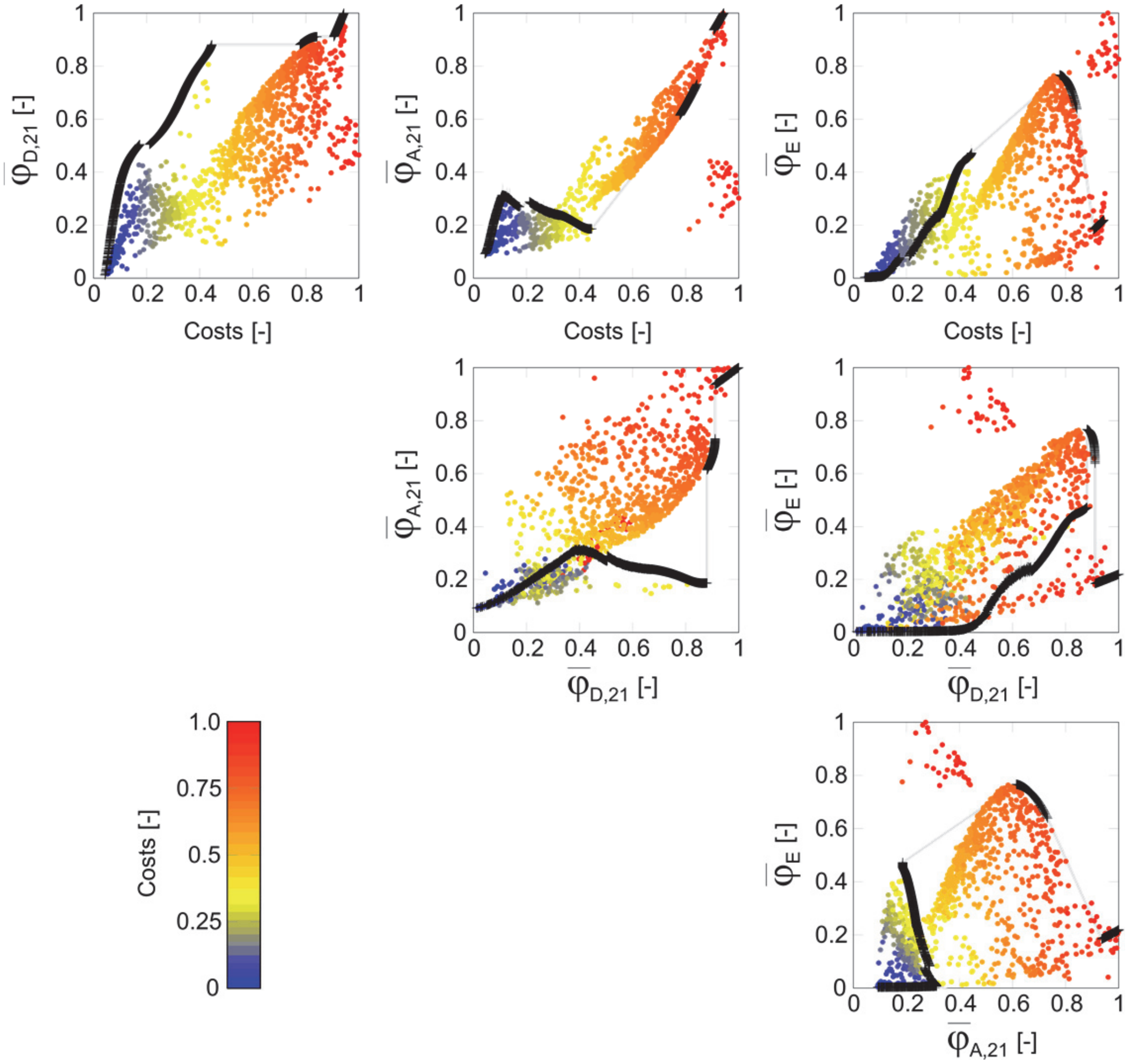

Fig D. Multi-objective optimization results for LC-MS (p=21). Calculated Pareto front for the 5D-MO-ED problem (objectives: D-, A-, E-criteria, dimensions and costs). Color codes for costs of design point. Black plus signs indicate the results of the 3D-MO-ED (objectives: D-criterion, dimensions and costs). All criteria are scaled to [0,1].

3. LC-MS/MS

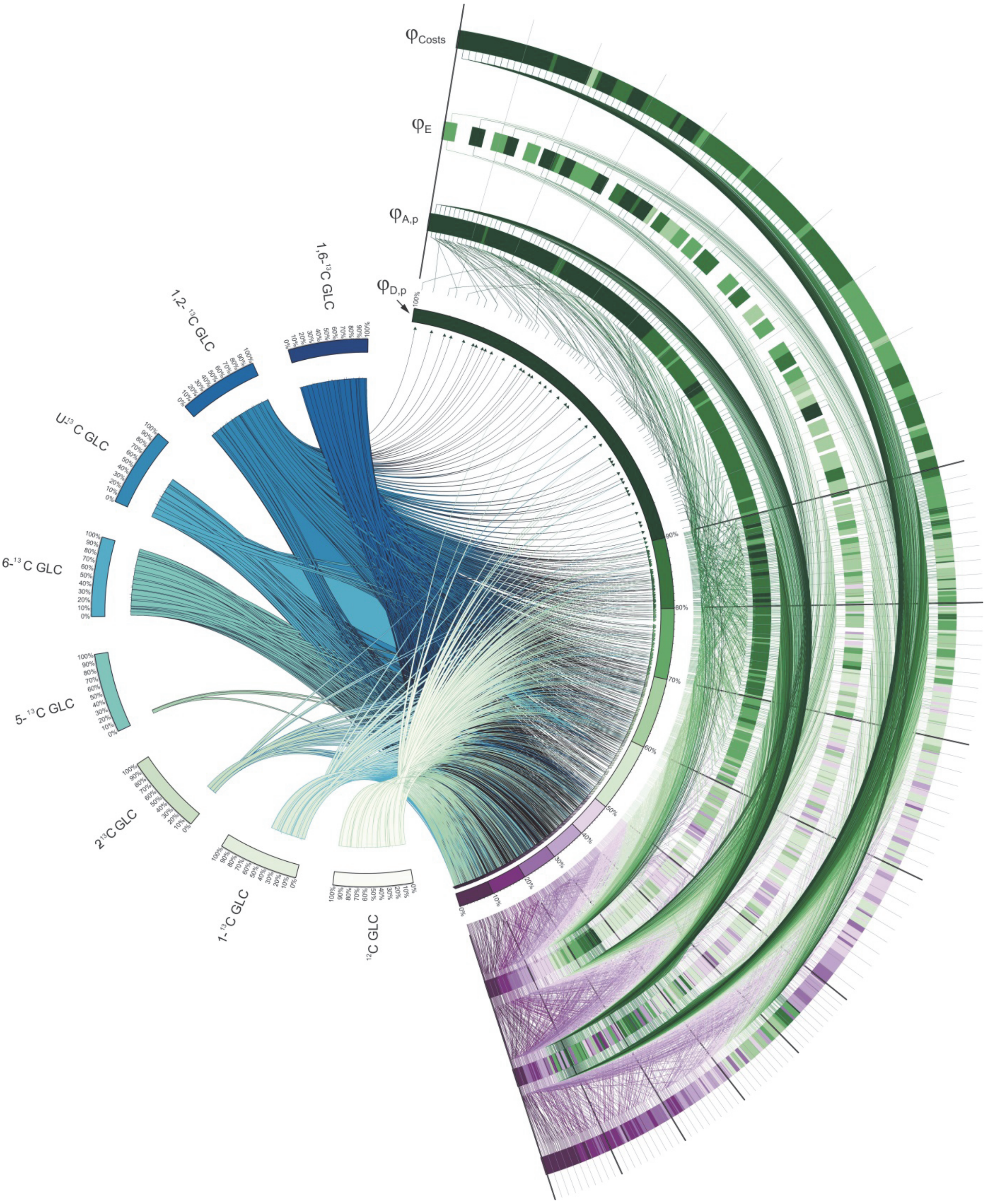

Fig E. 5D multi-objective optimization results for LC-MS/MS (p=21). Cord diagram showing design and objective spaces.

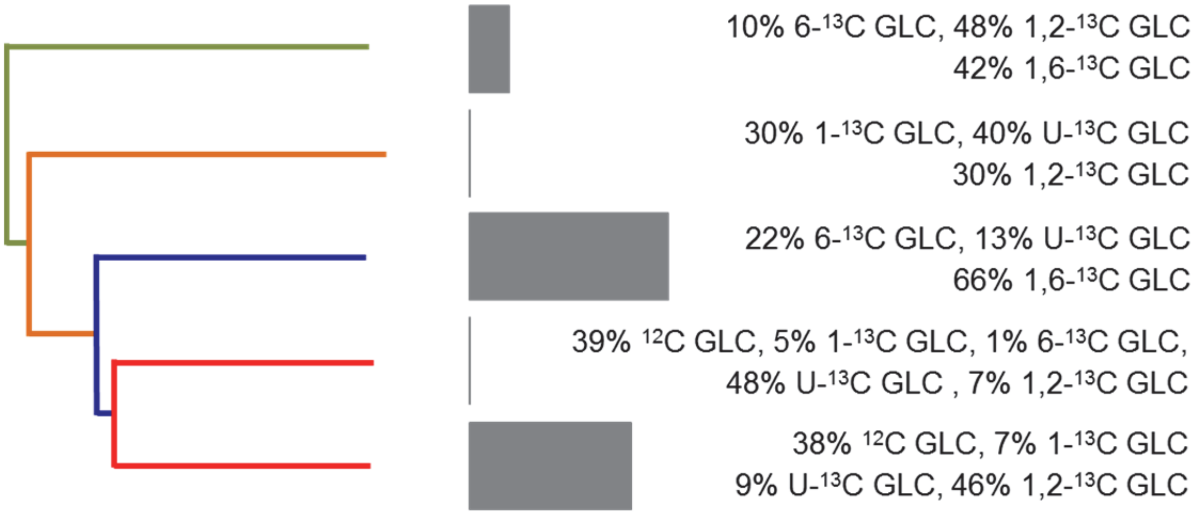

Fig F. Dendrogram of 5D-MO-ED some substrate mixture clusters for LC-MS/MS derived by hierarchical clustering (minimal Euclidean distance of the labeling fraction composition, p=21). Values below 1% were omitted. The length of the edges (distance) represents the dissimilarities of the mixtures. Five mixture clusters were determined for which composition average values are given along with their relative frequency. The corresponding number of measurement groups and replicates resolved by the five clusters are given in S5 Fig G-K.

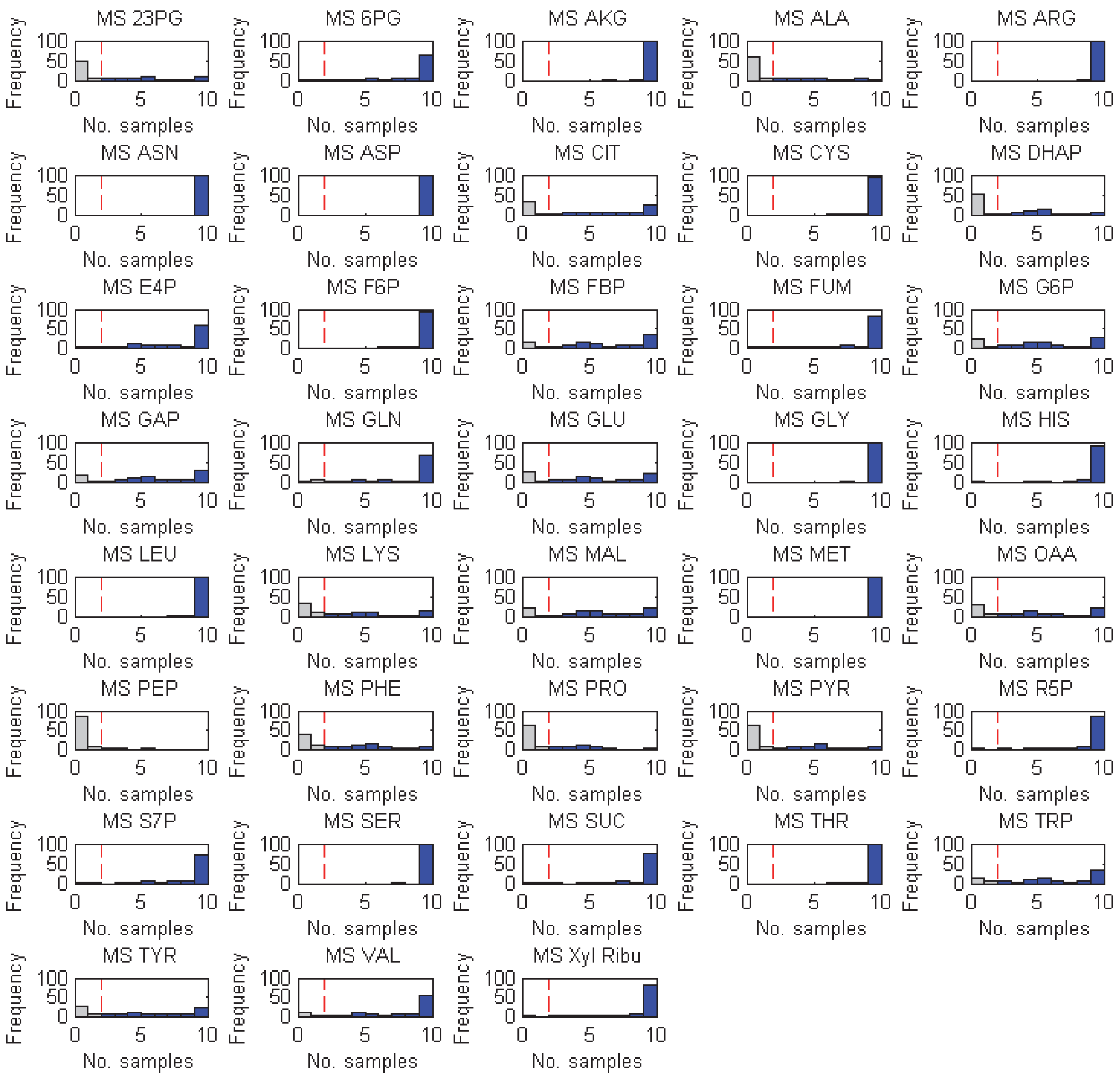

Fig G. Measurement groups and replicate numbers of the 5D-MO-ED solution for LC-MS/MS and substrate mixture cluster #1: 10% [6-^{13}C]-glucose, 48% [1,2-^{13}C]-glucose, 42% [1,6-^{13}C]-glucose. Measurement groups with less than two replicates (red line) were not considered in the analysis.

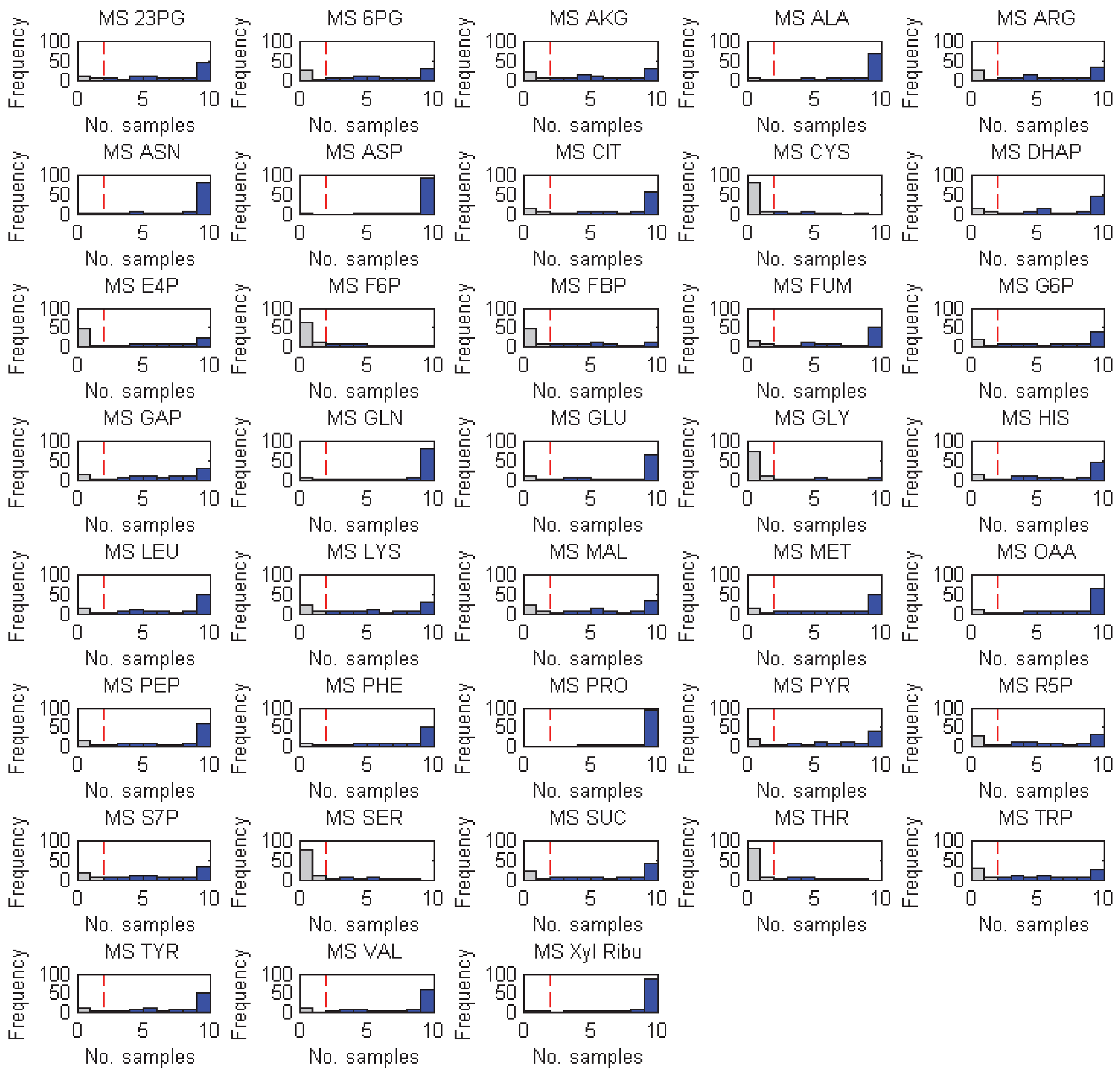

Fig H. Measurement groups and replicate numbers of the 5D-MO-ED solution for LC-MS/MS and substrate mixture cluster #2: 30% [1-^{13}C]- glucose, 40% [U-^{13}C]- glucose, 30% [1,2-^{13}C]-glucose. Measurement groups with less than two replicates (red line) were not considered in the analysis.

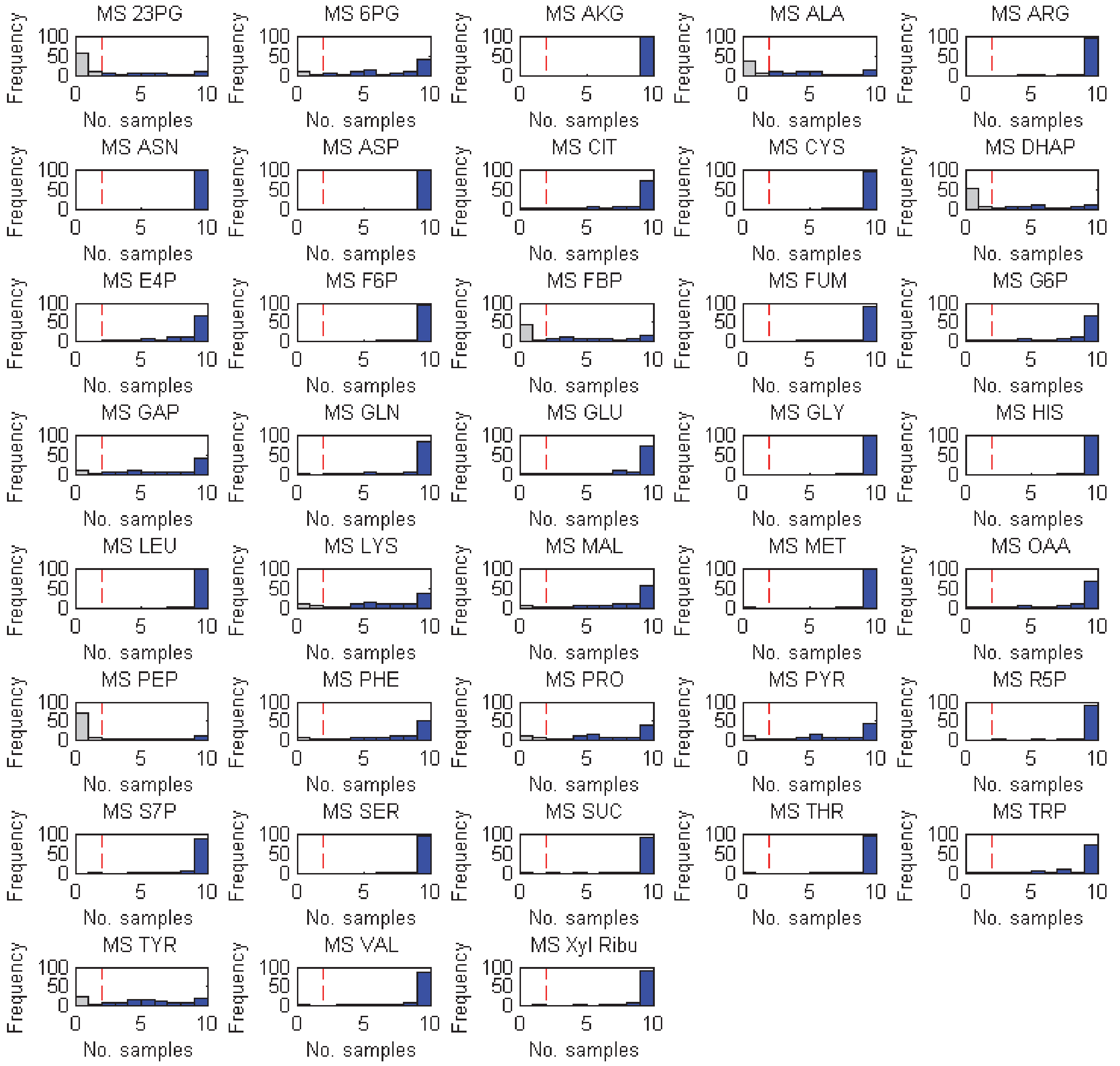

Fig I. Measurement groups and replicate numbers of the 5D-MO-ED solution for LC-MS/MS and substrate mixture cluster #3: 22% [6-^{13}C]-glucose, 13% [U-^{13}C]-glucose, 66% [1,6-^{13}C]-glucose. Measurement groups with less than two replicates (red line) were not considered in the analysis.

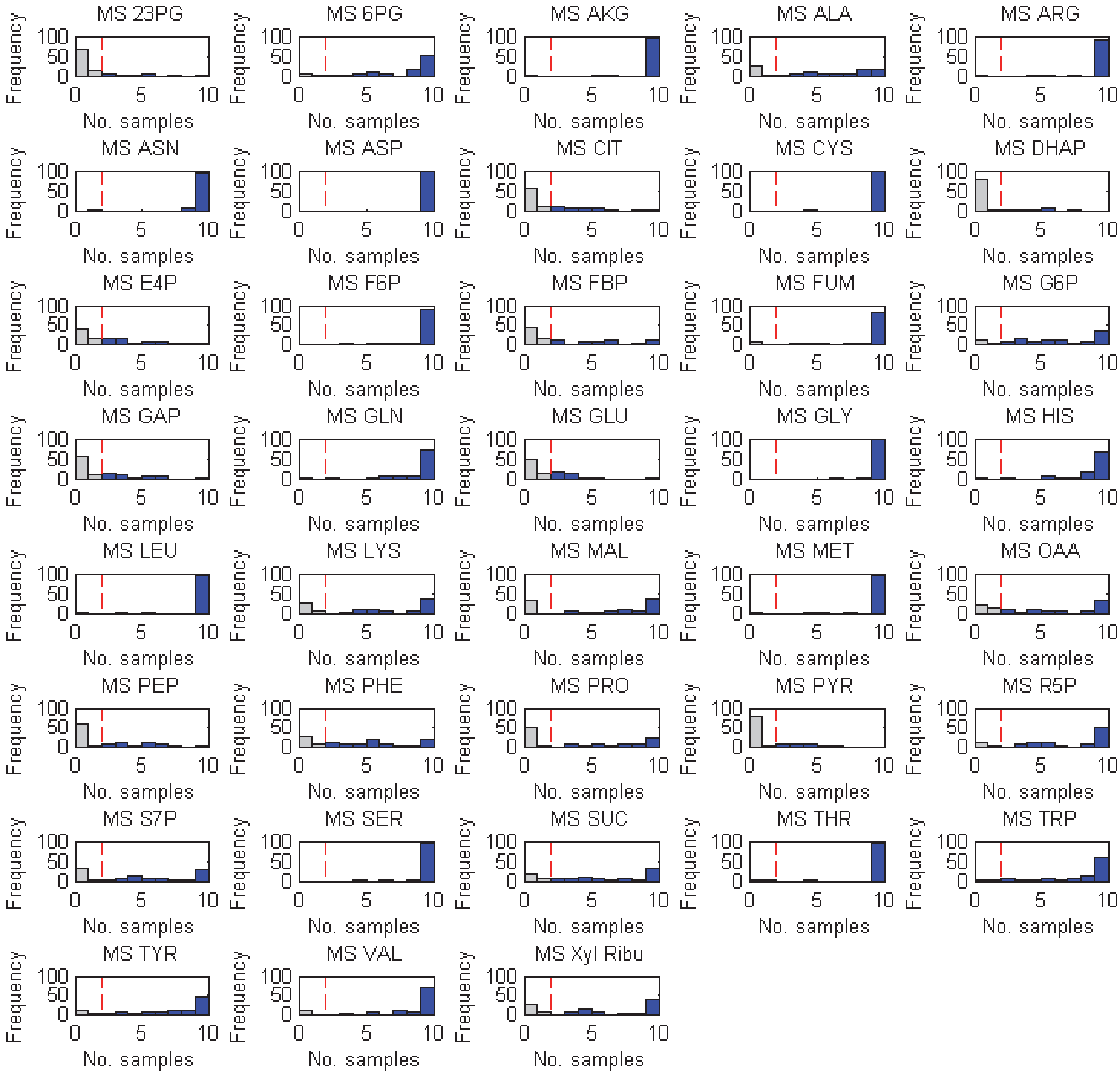

Fig J. Measurement groups and replicate numbers of the 5D-MO-ED solution for LC-MS/MS and substrate mixture cluster #4: 39% [^{12}C]-glucose, 5% [1-^{13}C]-glucose, 1% [6-^{13}C]-glucose, 48% [U-^{13}C]-glucose, 7% [1,2-^{13}C]-glucose. Measurement groups with less than two replicates (red line) were not considered in the analysis.

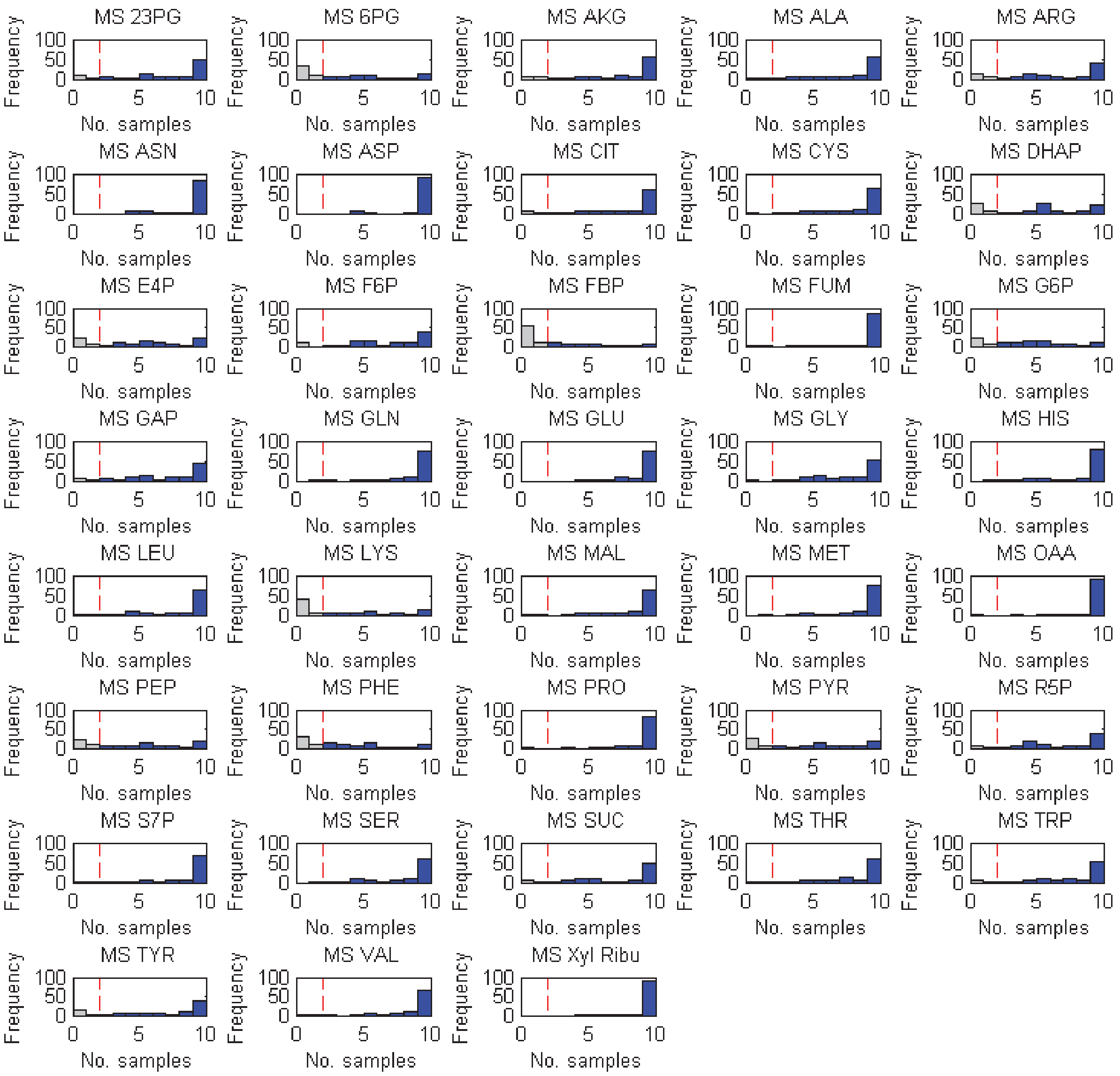

Fig K. Measurement groups and replicate numbers of the 5D-MO-ED solution for LC-MS/MS and substrate mixture cluster #5: 38% [^{12}C]-glucose, 7% [1-^{13}C]-glucose, 9% [U-^{13}C]-glucose, 46% [1,2-^{13}C]-glucose. Measurement groups with less than two replicates (red line) were not considered in the analysis.

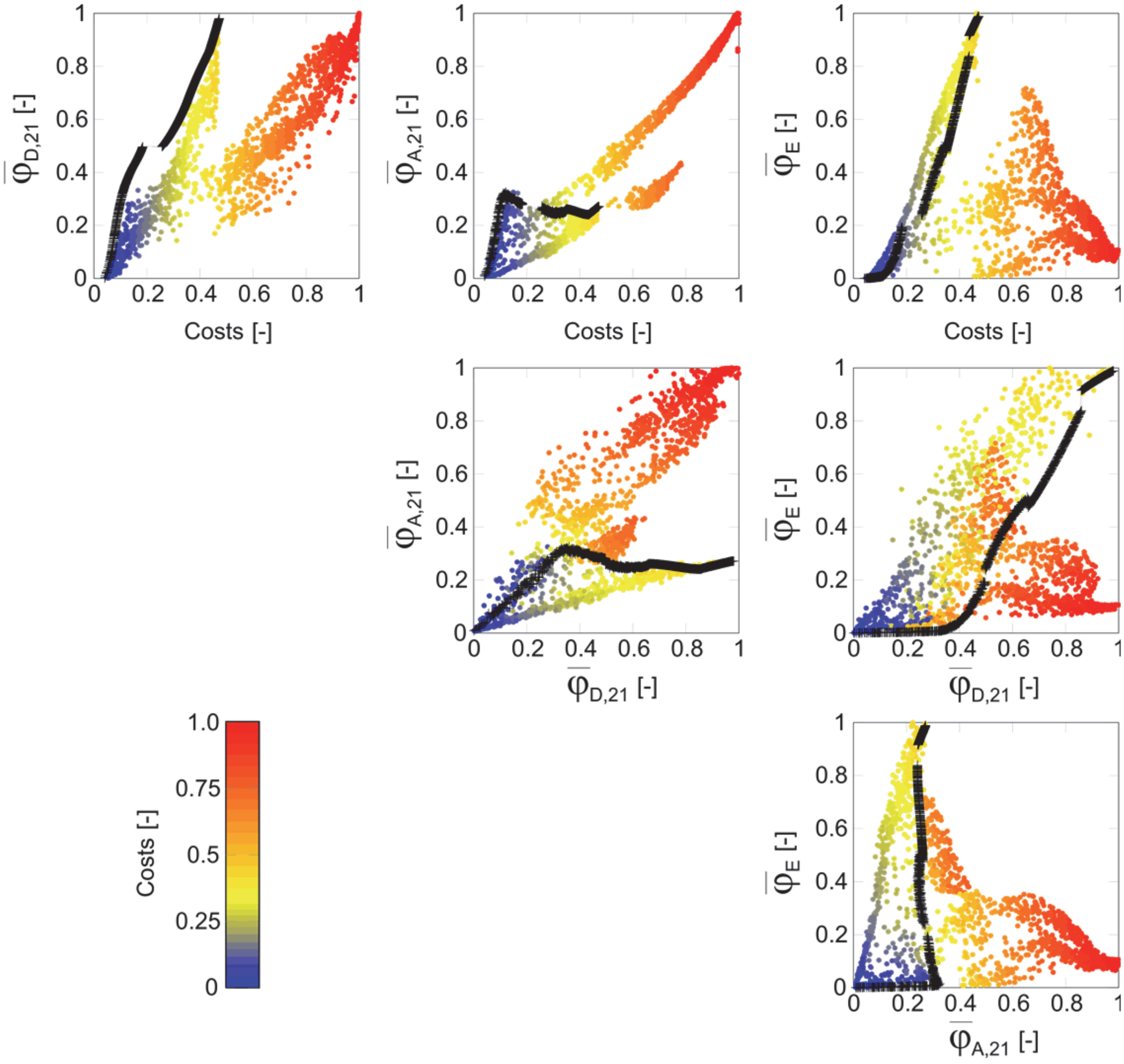

Fig L. Multi-objective optimization results for LC-MS/MS (p=21). Calculated Pareto front for the 5D-MO-ED problem (objectives: D-, A-, E-criteria, dimensions and costs). Color codes for costs of design point. Black plus signs indicate the results of the 3D-MO-ED (objectives: D-criterion, dimensions and costs). All criteria are scaled to [0,1] (enlarged version of Fig 8 in the main text).

4. ^{13}C-NMR

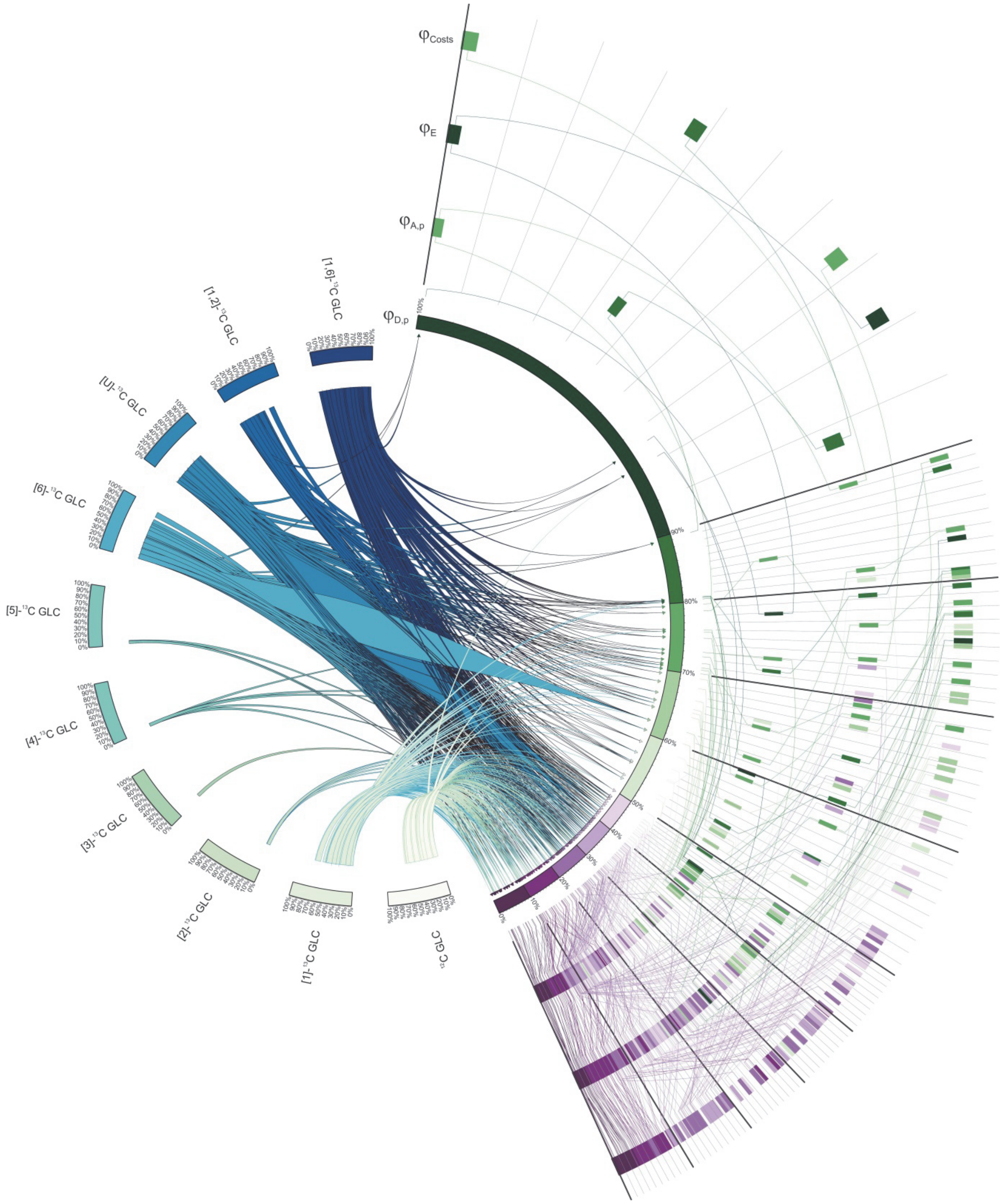

Fig M. 5D multi-objective optimization results for ^{13}C-NMR (p=21). Cord diagram showing design and objective spaces.

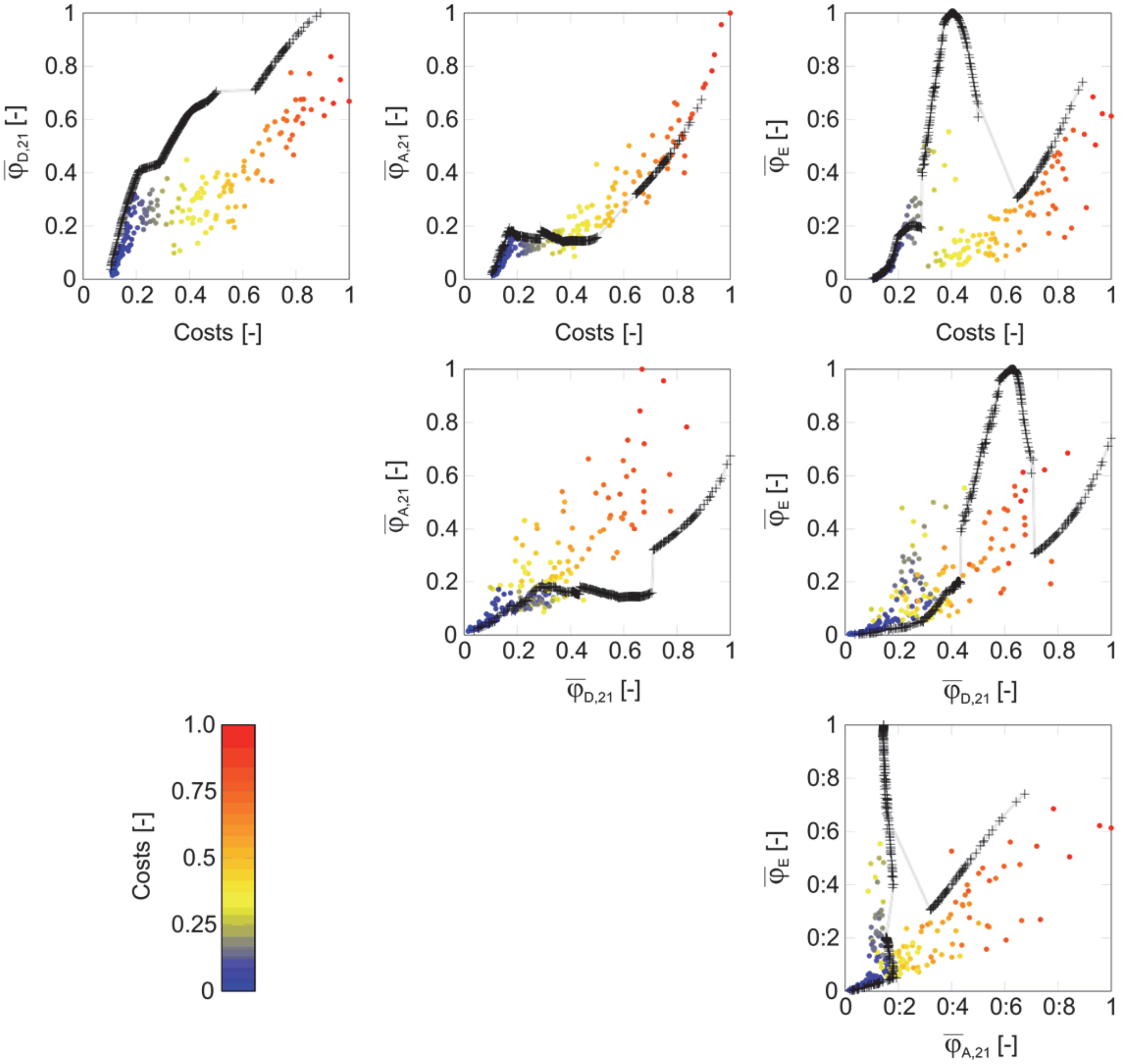

Fig N. Multi-objective optimization results for ^{13}C-NMR (p=21). Pareto front of the 5D-MO-ED problem (objectives: D-, A-, E-criteria, dimensions and costs). Color codes for costs of design point. Black plus signs indicate the results of the 3D-MO-ED problem (objectives: D-criterion, dimensions and costs). All criteria are scaled to [0,1].
