# Supplementary figures and images for: A Pareto approach to resolve the conflict between information gain and experimental costs: Multiple-criteria design of carbon labeling experiments
Source: PLoS Comput Biol. 2018 Oct 31;14(10):e1006533. doi: 10.1371/journal.pcbi.1006533 (PMC6209137; doi:10.1371/journal.pcbi.1006533)

LC-MS/MS

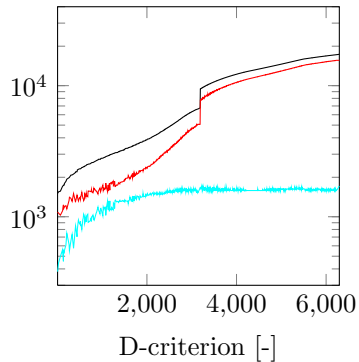

GC-MS

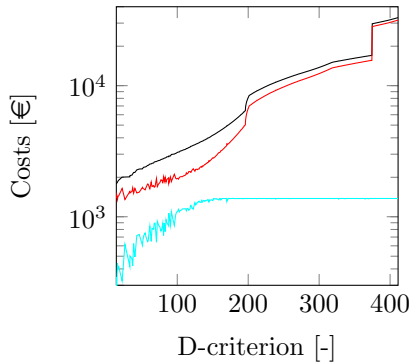

C-NMR

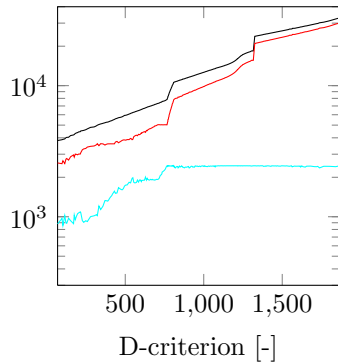

LC-MS

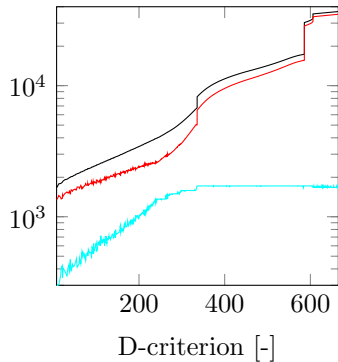

Supplement: S1 File — ZIP archive containing Pareto scatterplots for 3D- and 5D-MO-ED scenarios and scripts to reproduce Figs 7 and 8. (ZIP) [file pcbi.1006533.s007.zip › Scatter/3D_Comparison_Fig7/CostsDcrit.pdf]

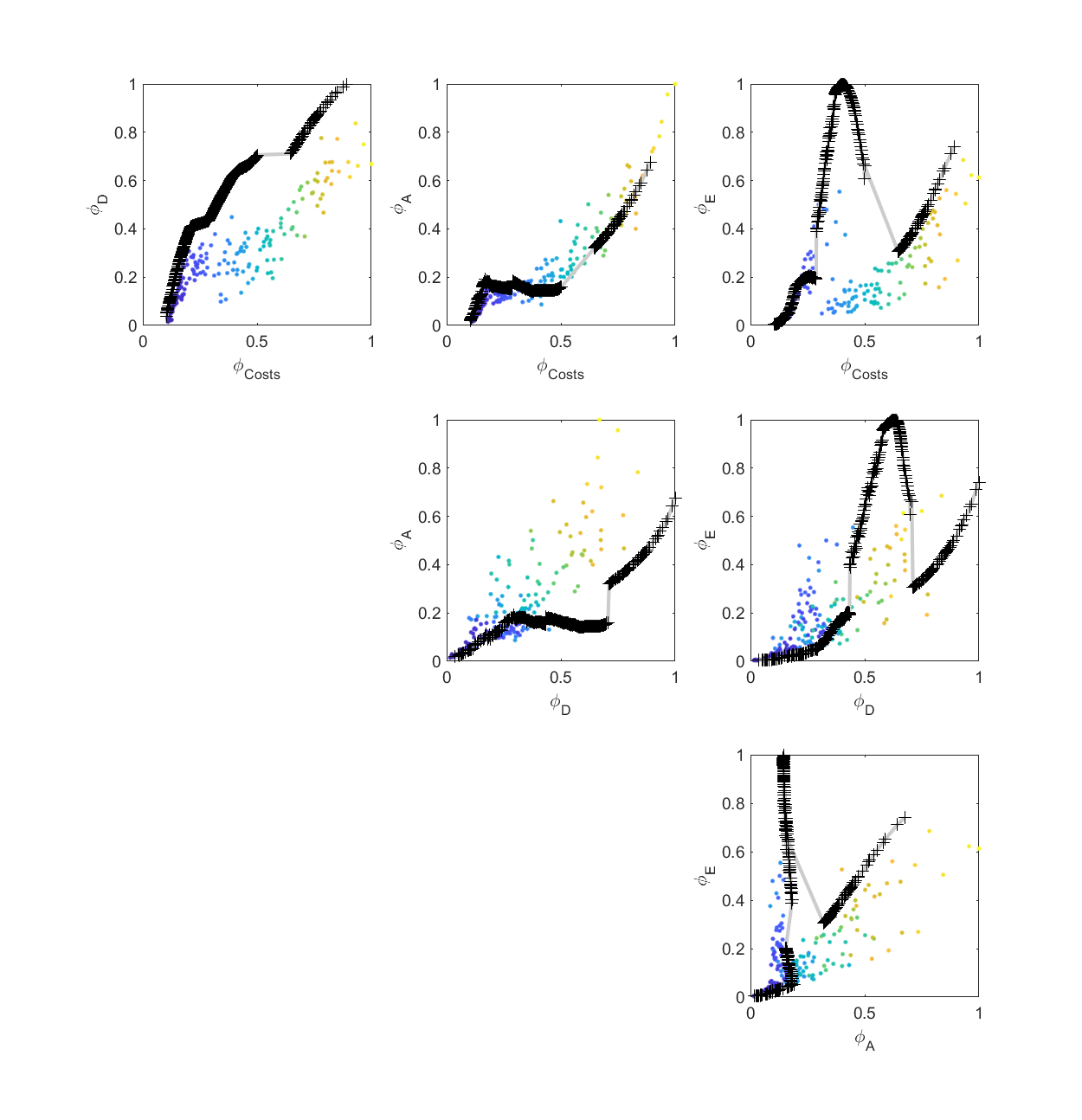

Supplement: S1 File — ZIP archive containing Pareto scatterplots for 3D- and 5D-MO-ED scenarios and scripts to reproduce Figs 7 and 8. (ZIP) [file pcbi.1006533.s007.zip › Scatter/Scatter_5D_CNMR/Pareto_5D_CNMR_2D_21.png]

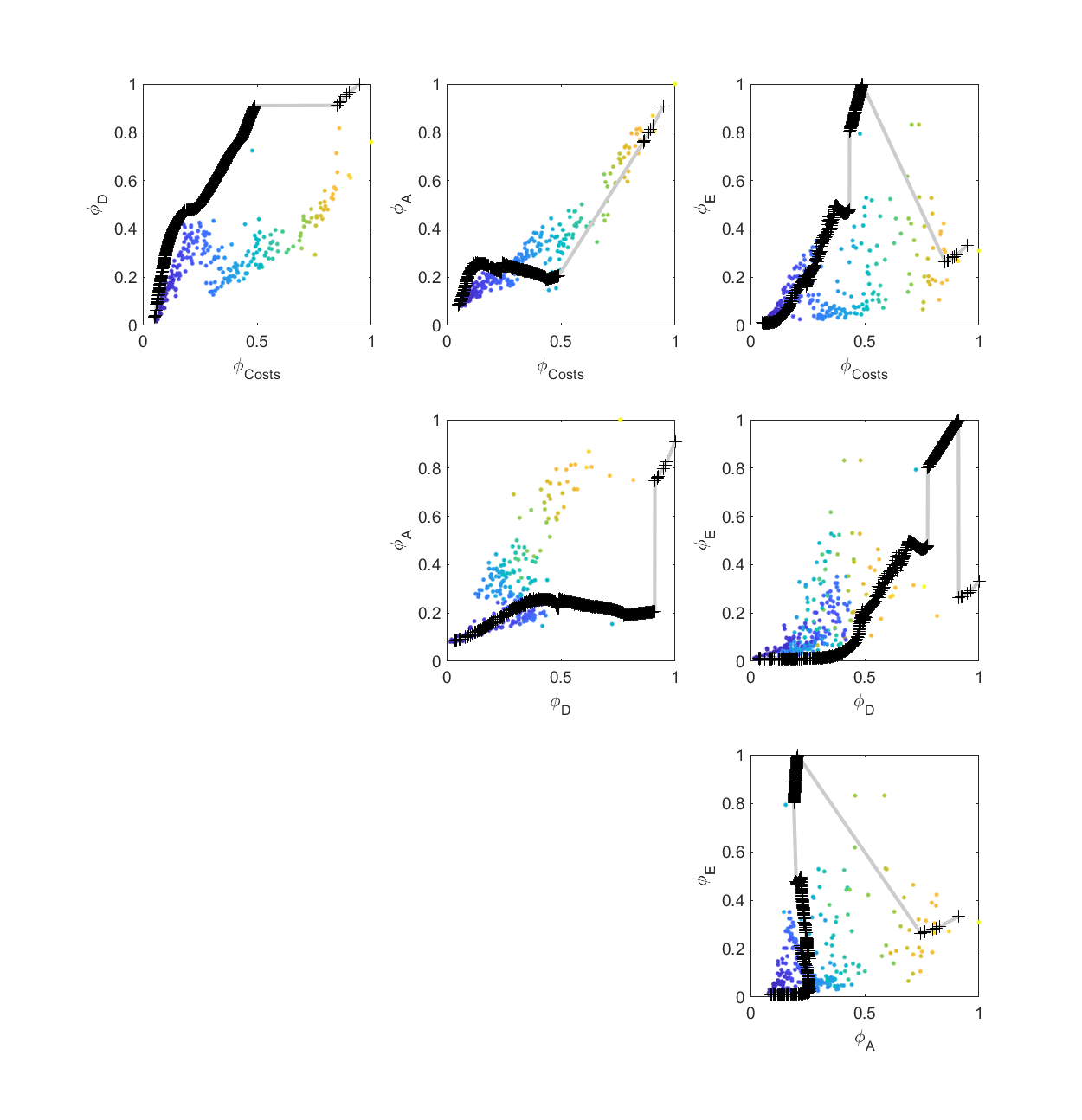

Supplement: S1 File — ZIP archive containing Pareto scatterplots for 3D- and 5D-MO-ED scenarios and scripts to reproduce Figs 7 and 8. (ZIP) [file pcbi.1006533.s007.zip › Scatter/Scatter_5D_GCMS/Pareto_5D_GCMS_2D_21.png]

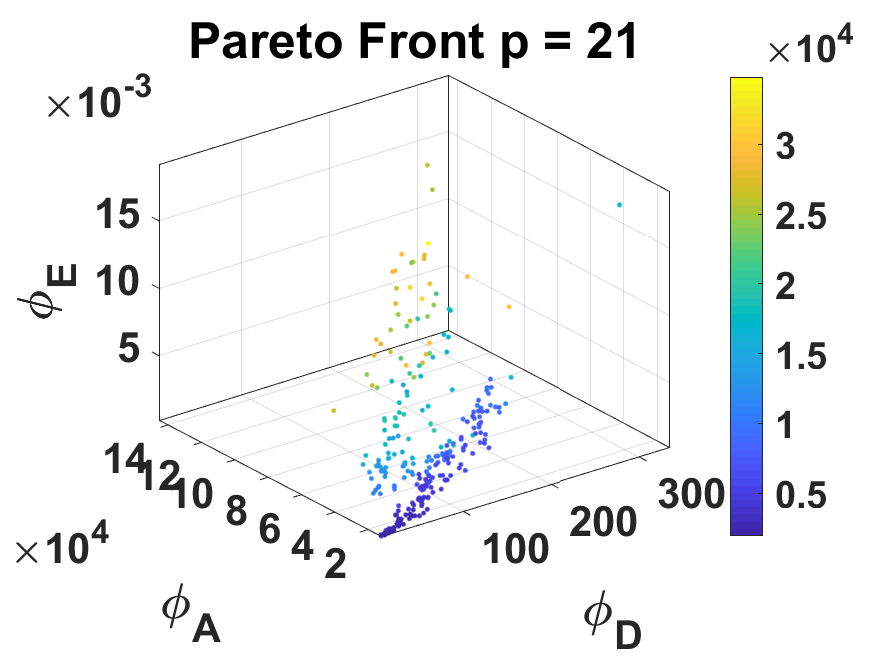

Supplement: S1 File — ZIP archive containing Pareto scatterplots for 3D- and 5D-MO-ED scenarios and scripts to reproduce Figs 7 and 8. (ZIP) [file pcbi.1006533.s007.zip › Scatter/Scatter_5D_GCMS/Pareto_5D_GCMS_3D_p_21.png]

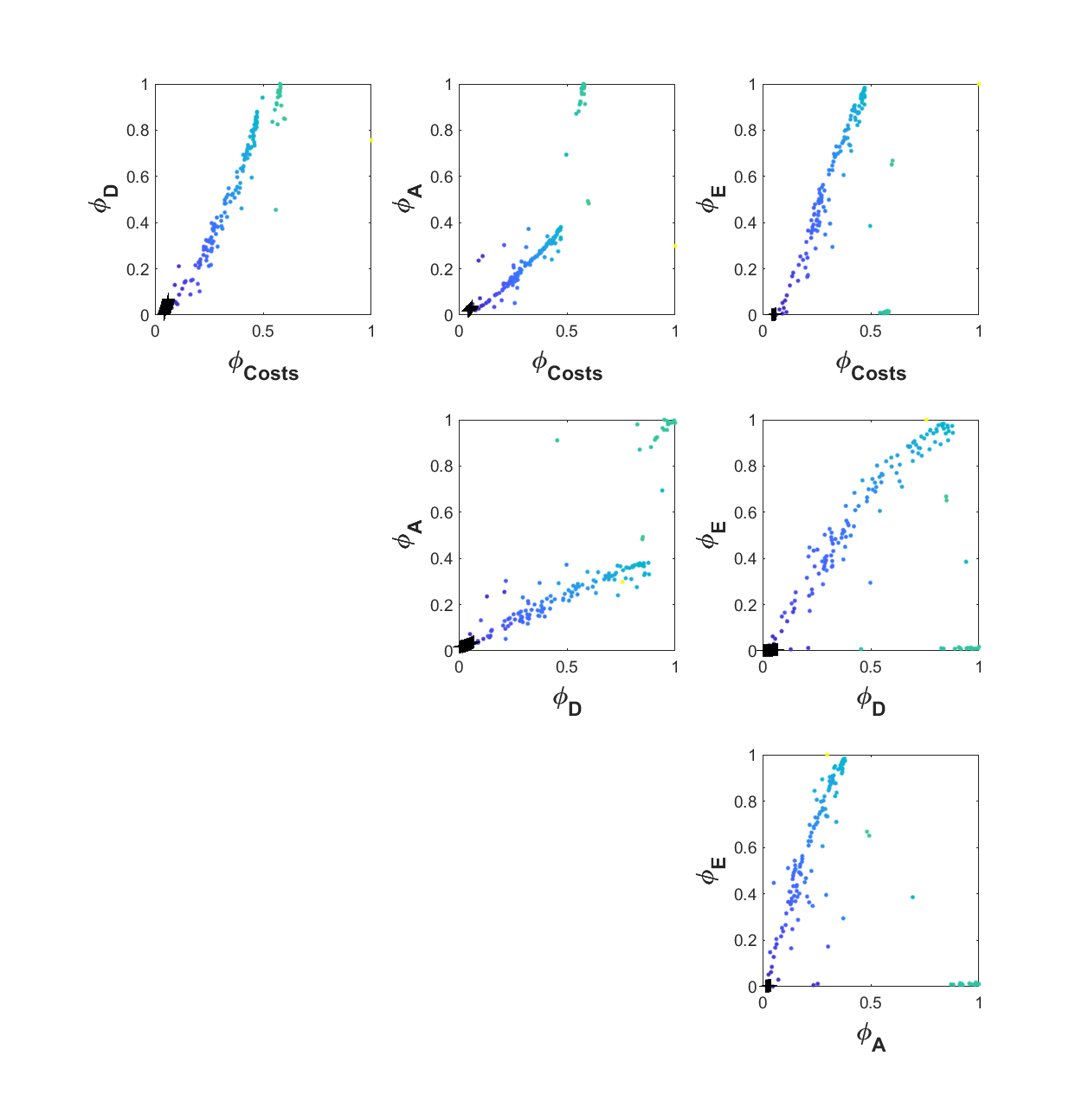

Supplement: S1 File — ZIP archive containing Pareto scatterplots for 3D- and 5D-MO-ED scenarios and scripts to reproduce Figs 7 and 8. (ZIP) [file pcbi.1006533.s007.zip › Scatter/Scatter_5D_LCMSMS/Pareto_5D_LCMSMS_2D_18.png]

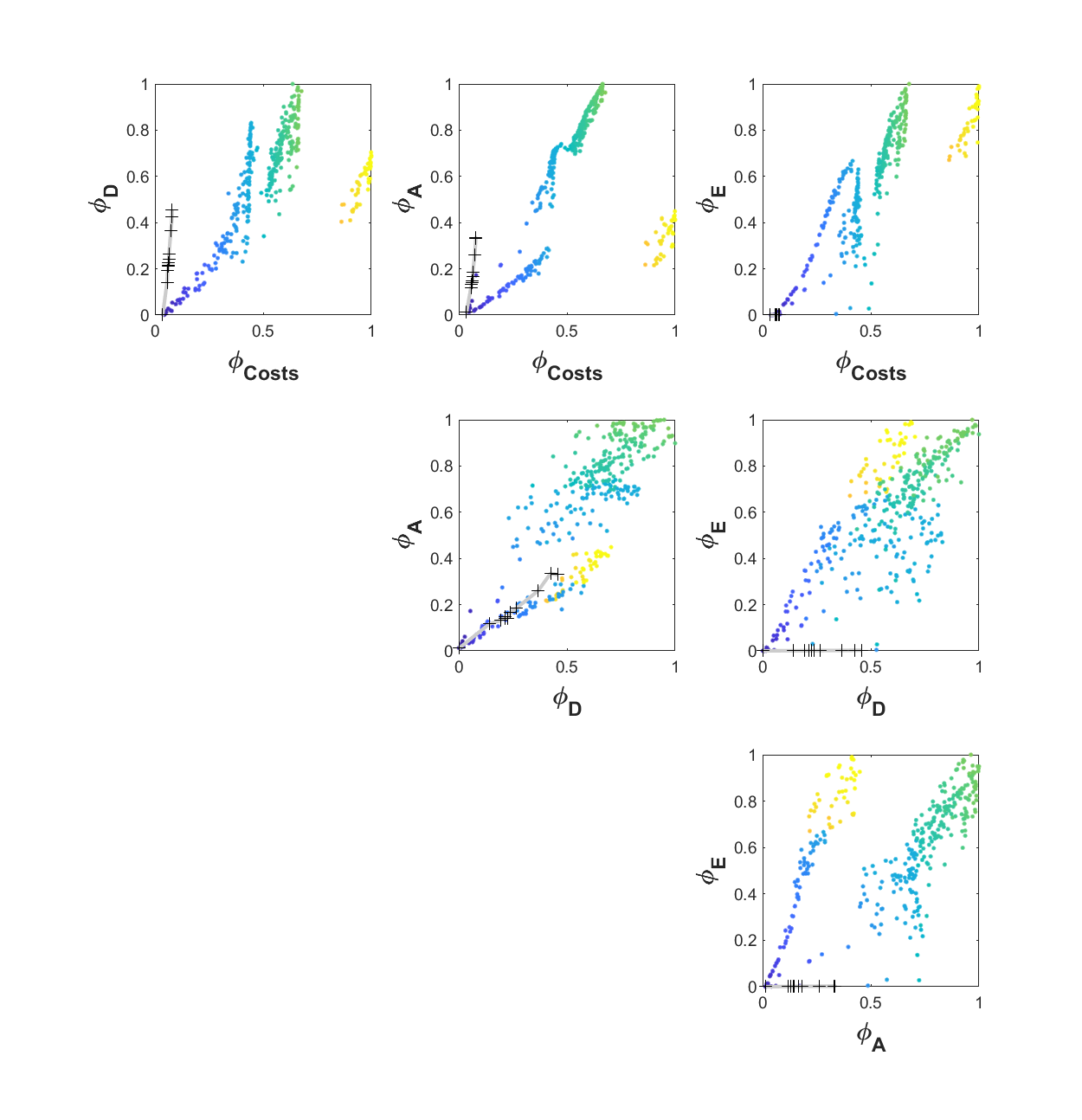

Supplement: S1 File — ZIP archive containing Pareto scatterplots for 3D- and 5D-MO-ED scenarios and scripts to reproduce Figs 7 and 8. (ZIP) [file pcbi.1006533.s007.zip › Scatter/Scatter_5D_LCMSMS/Pareto_5D_LCMSMS_2D_19.png]

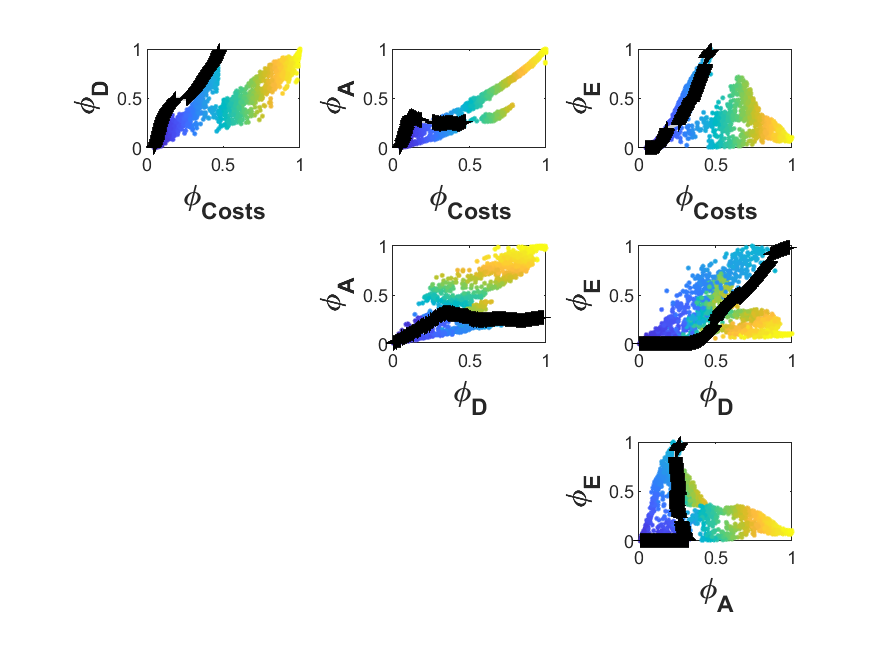

Supplement: S1 File — ZIP archive containing Pareto scatterplots for 3D- and 5D-MO-ED scenarios and scripts to reproduce Figs 7 and 8. (ZIP) [file pcbi.1006533.s007.zip › Scatter/Scatter_5D_LCMSMS/Pareto_5D_LCMSMS_2D_21.png]

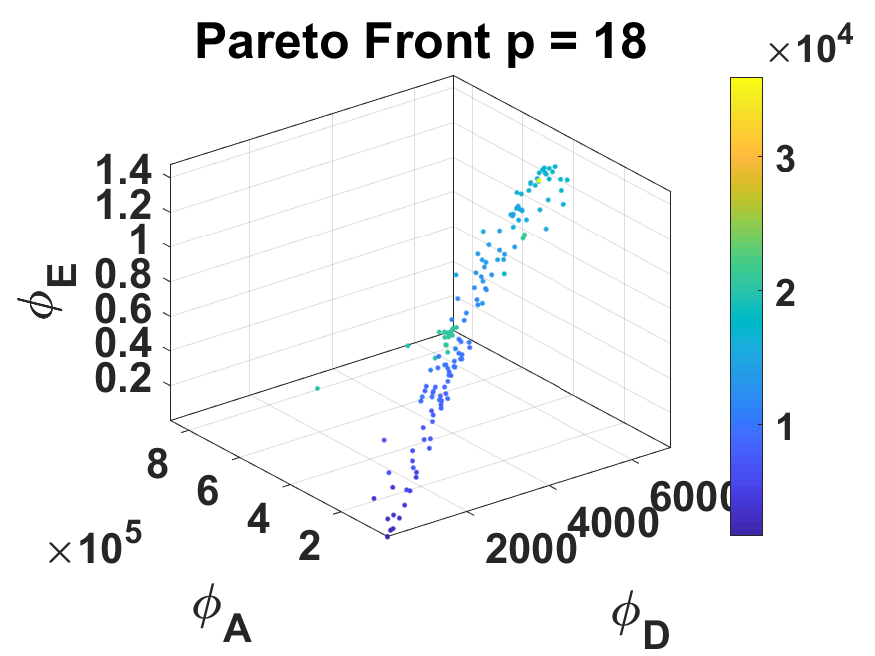

Supplement: S1 File — ZIP archive containing Pareto scatterplots for 3D- and 5D-MO-ED scenarios and scripts to reproduce Figs 7 and 8. (ZIP) [file pcbi.1006533.s007.zip › Scatter/Scatter_5D_LCMSMS/Pareto_5D_LCMSMS_3D_p_18.png]

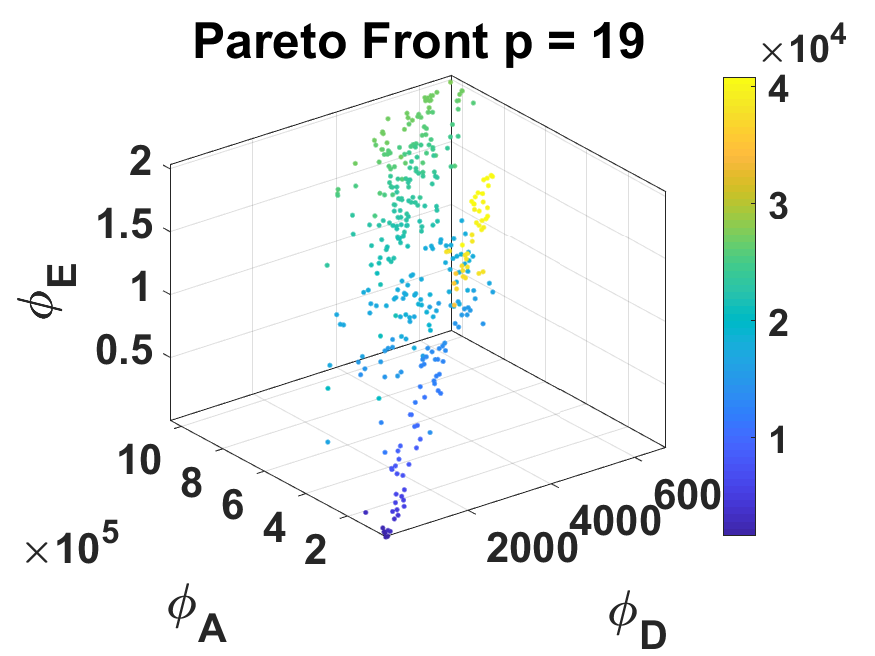

Supplement: S1 File — ZIP archive containing Pareto scatterplots for 3D- and 5D-MO-ED scenarios and scripts to reproduce Figs 7 and 8. (ZIP) [file pcbi.1006533.s007.zip › Scatter/Scatter_5D_LCMSMS/Pareto_5D_LCMSMS_3D_p_19.png]

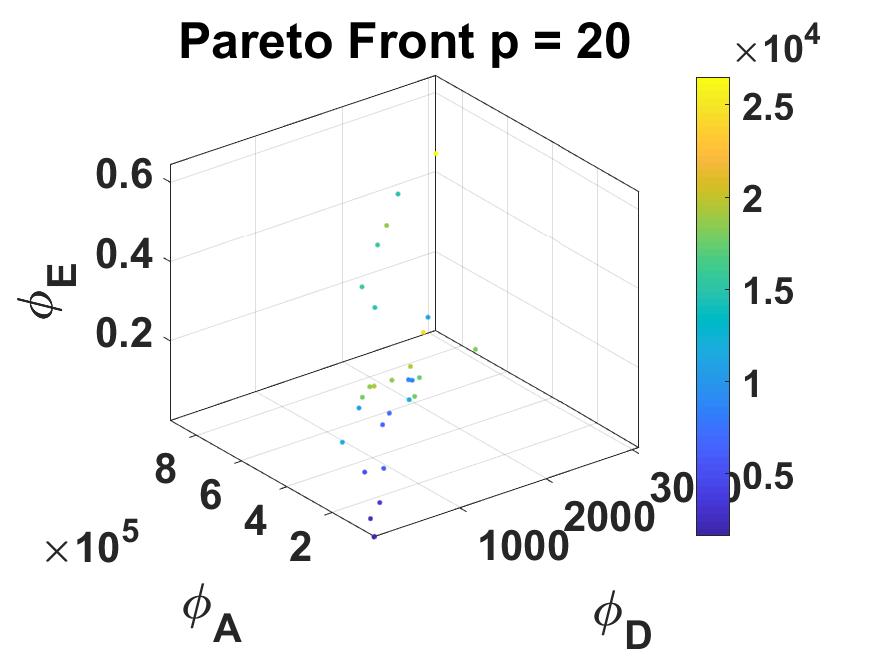

Supplement: S1 File — ZIP archive containing Pareto scatterplots for 3D- and 5D-MO-ED scenarios and scripts to reproduce Figs 7 and 8. (ZIP) [file pcbi.1006533.s007.zip › Scatter/Scatter_5D_LCMSMS/Pareto_5D_LCMSMS_3D_p_20.png]

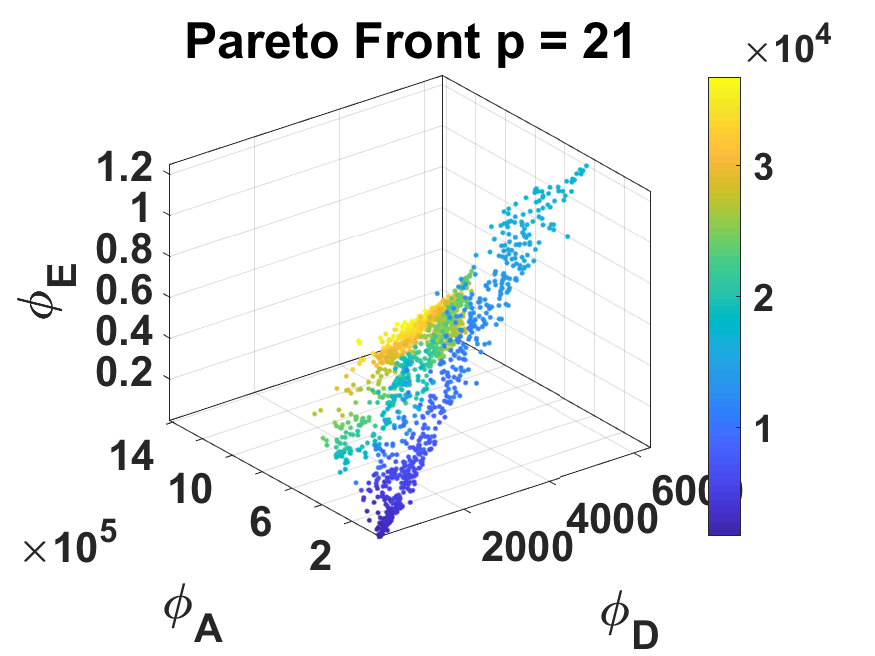

Supplement: S1 File — ZIP archive containing Pareto scatterplots for 3D- and 5D-MO-ED scenarios and scripts to reproduce Figs 7 and 8. (ZIP) [file pcbi.1006533.s007.zip › Scatter/Scatter_5D_LCMSMS/Pareto_5D_LCMSMS_3D_p_21.png]

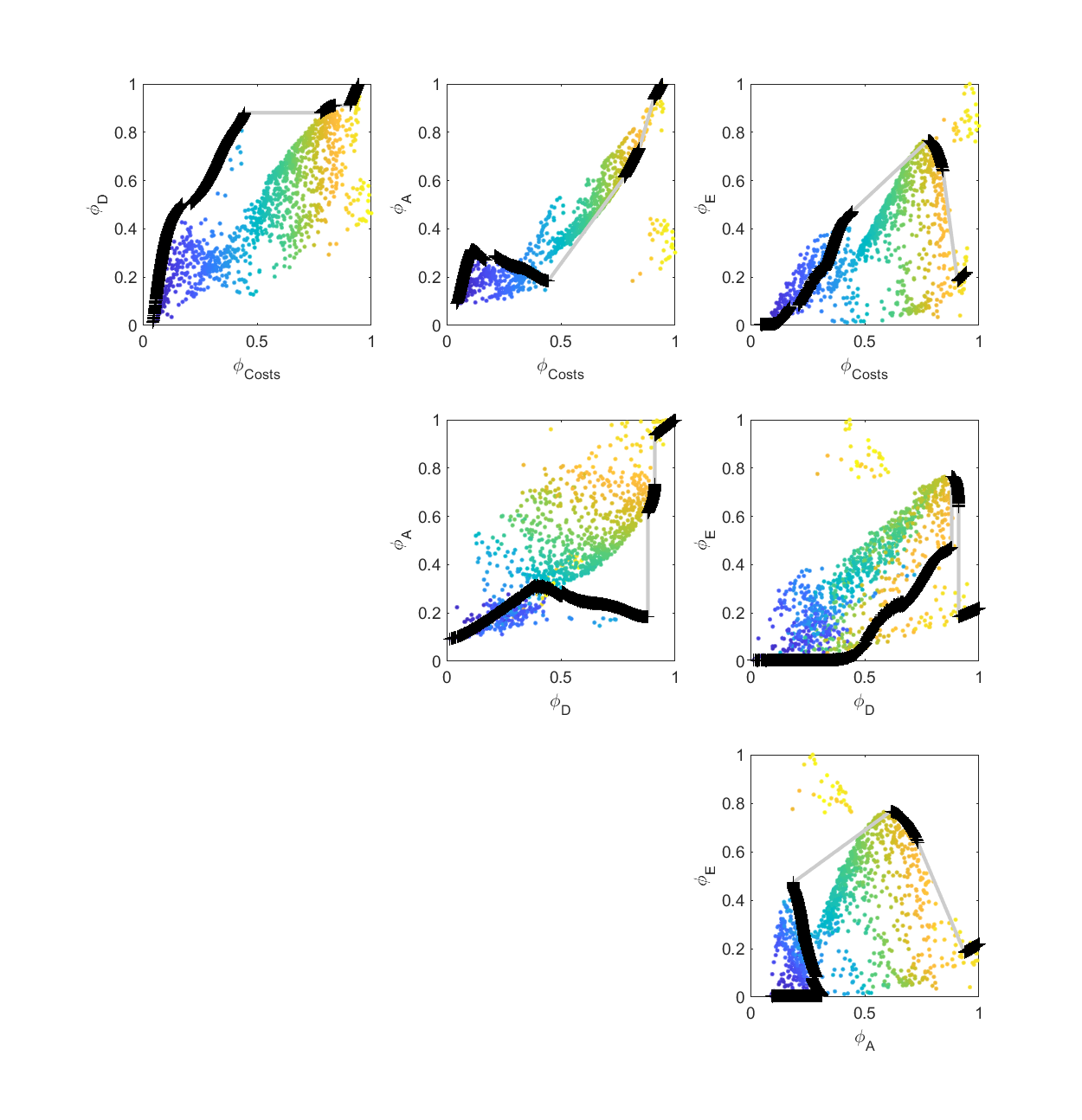

Supplement: S1 File — ZIP archive containing Pareto scatterplots for 3D- and 5D-MO-ED scenarios and scripts to reproduce Figs 7 and 8. (ZIP) [file pcbi.1006533.s007.zip › Scatter/Scatter_5D_LCMS/Pareto_5D_LCMS_2D_21.png]

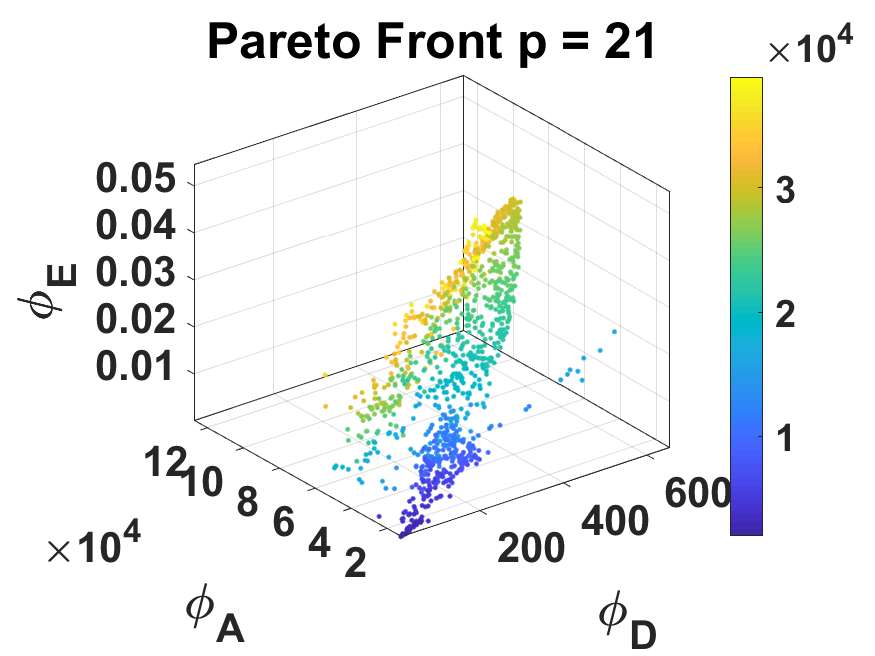

Supplement: S1 File — ZIP archive containing Pareto scatterplots for 3D- and 5D-MO-ED scenarios and scripts to reproduce Figs 7 and 8. (ZIP) [file pcbi.1006533.s007.zip › Scatter/Scatter_5D_LCMS/Pareto_5D_LCMS_3D_p_21.png]

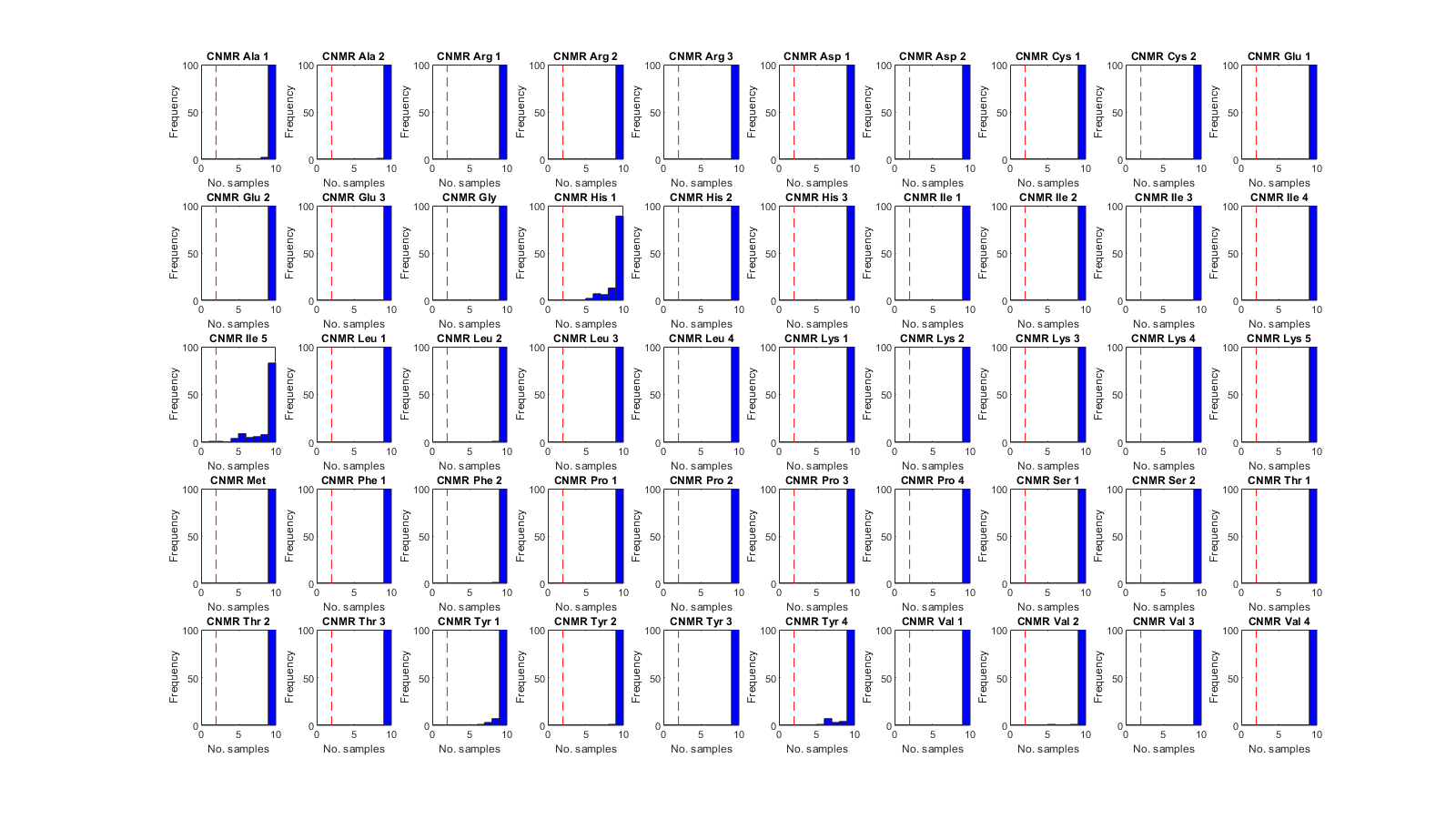

Supplement: S2 File — ZIP archive containing Matlab scripts to produce the substrate clusters and replicate evaluations (S4 and S5 Text). (ZIP) [file pcbi.1006533.s008.zip › Cluster/Cluster_3D_CNMR/Ausgabe_1.png]

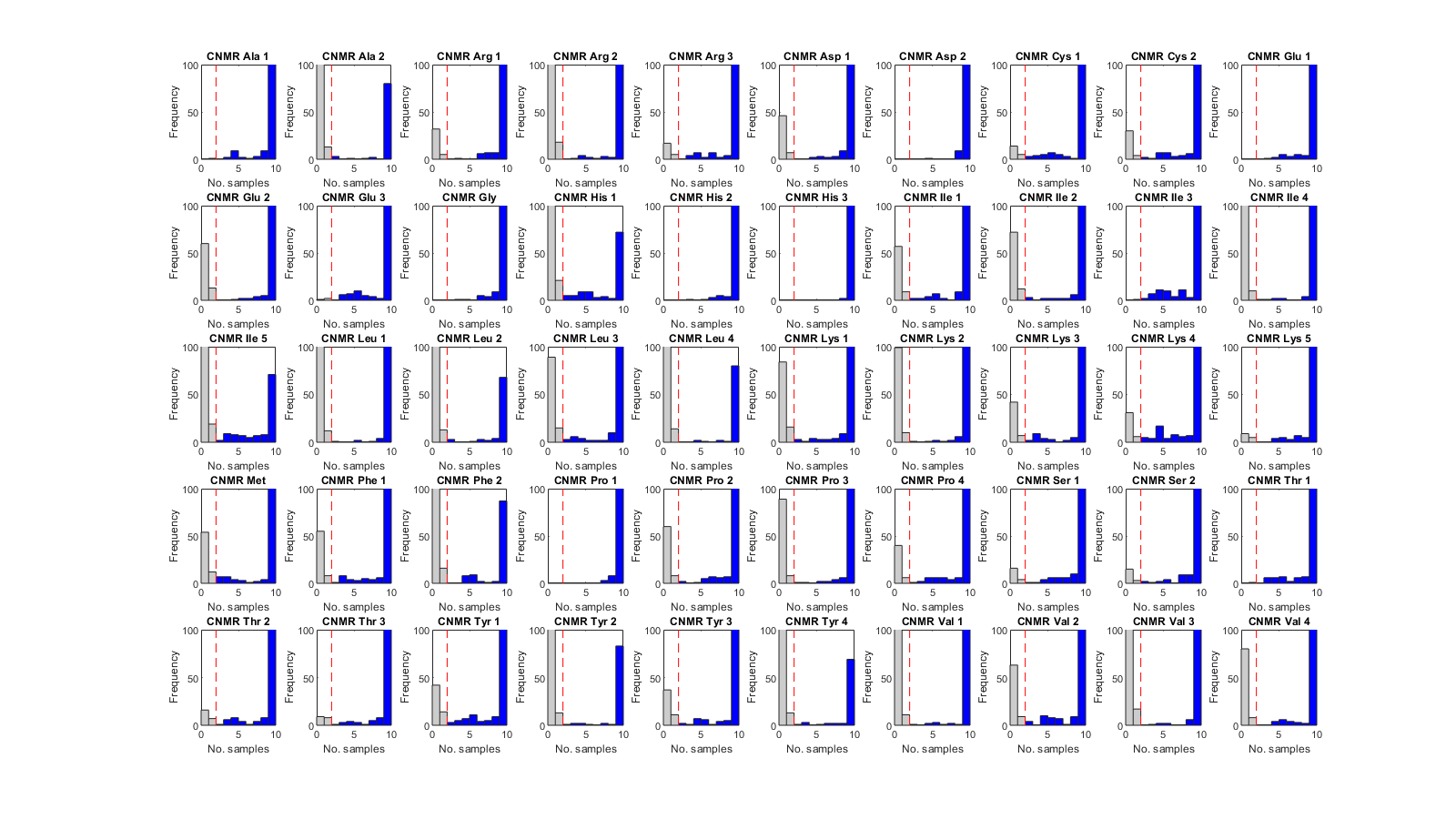

Supplement: S2 File — ZIP archive containing Matlab scripts to produce the substrate clusters and replicate evaluations (S4 and S5 Text). (ZIP) [file pcbi.1006533.s008.zip › Cluster/Cluster_3D_CNMR/Ausgabe_2.png]

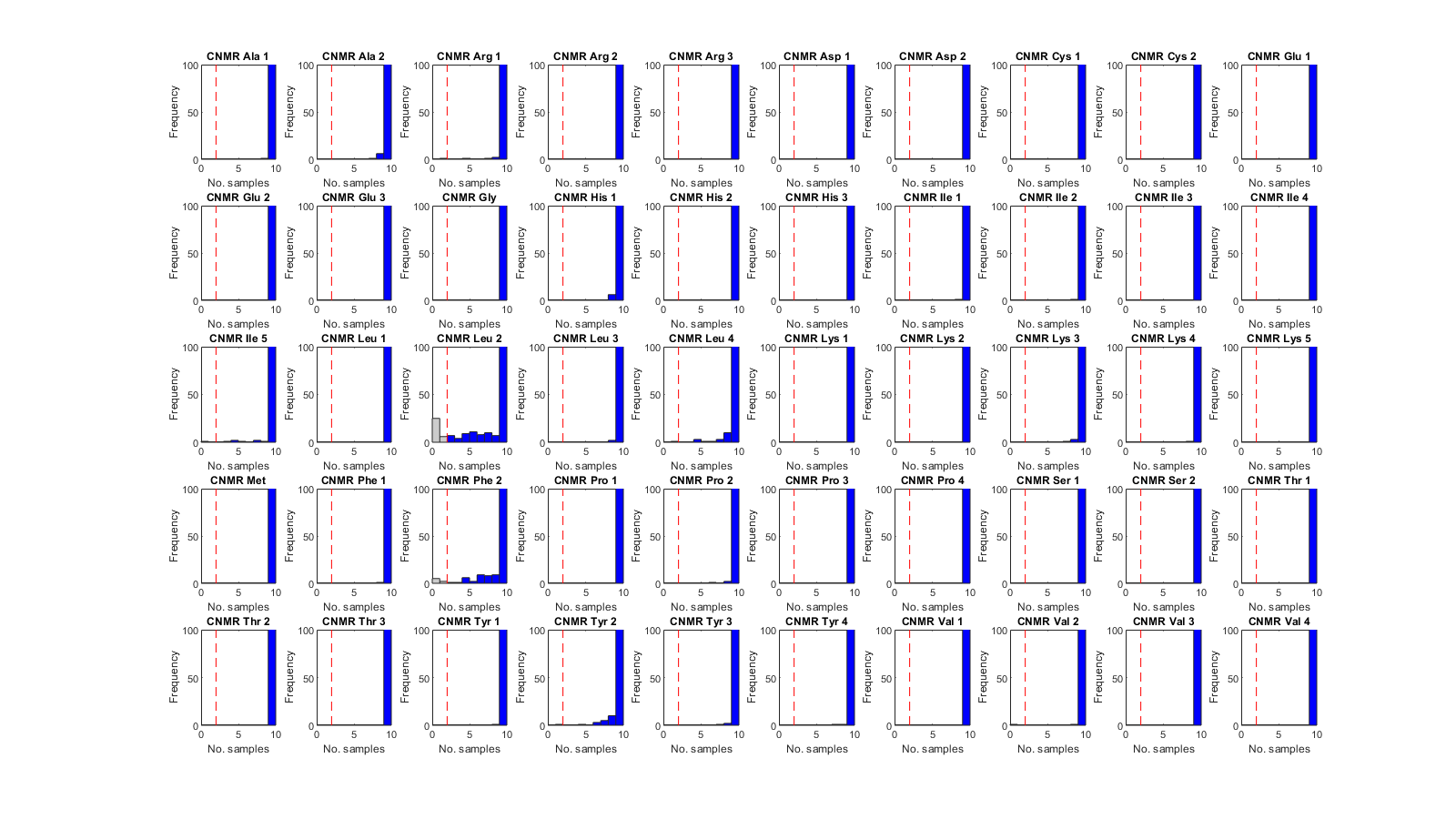

Supplement: S2 File — ZIP archive containing Matlab scripts to produce the substrate clusters and replicate evaluations (S4 and S5 Text). (ZIP) [file pcbi.1006533.s008.zip › Cluster/Cluster_3D_CNMR/Ausgabe_3.png]

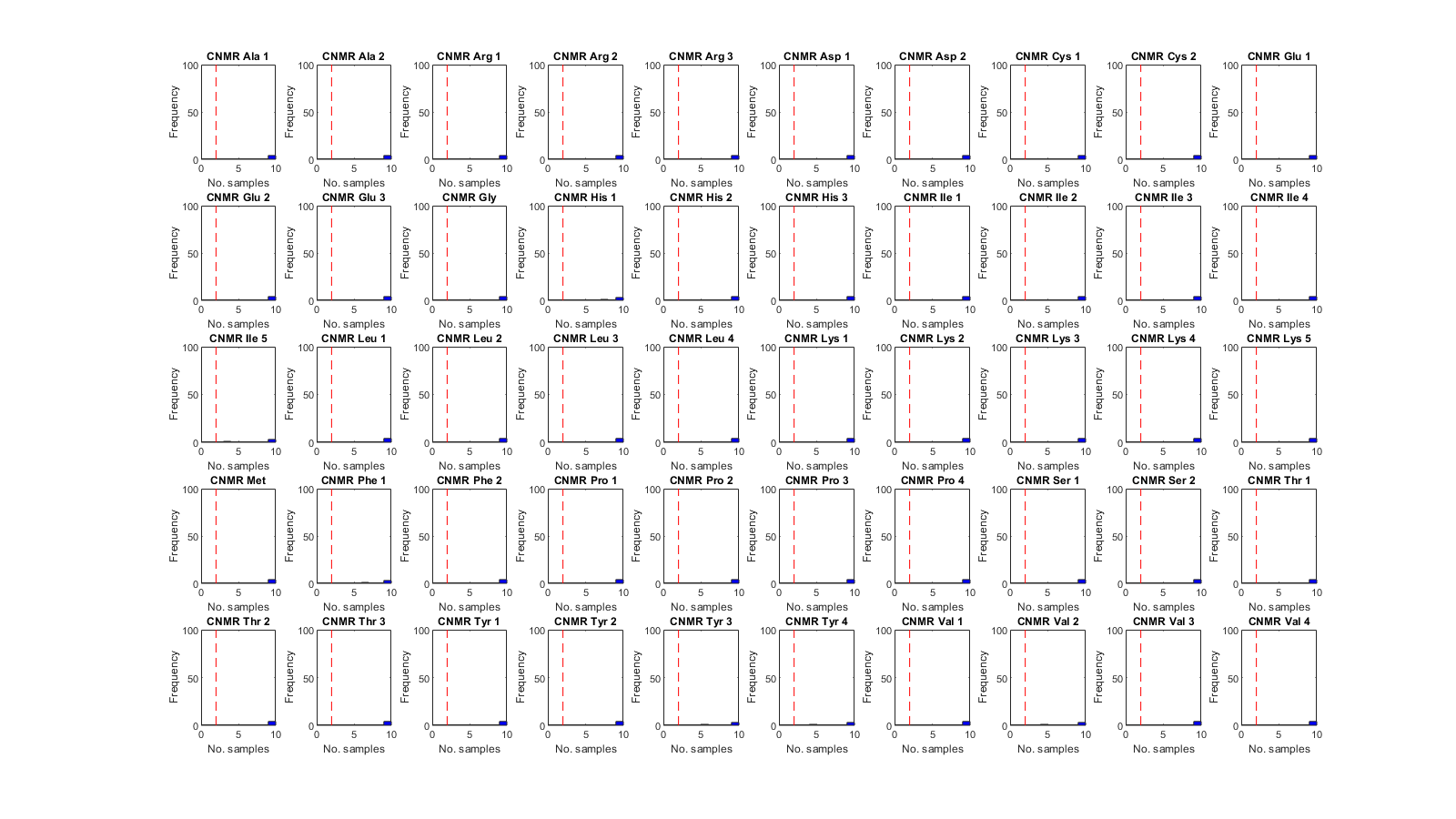

Supplement: S2 File — ZIP archive containing Matlab scripts to produce the substrate clusters and replicate evaluations (S4 and S5 Text). (ZIP) [file pcbi.1006533.s008.zip › Cluster/Cluster_3D_CNMR/Ausgabe_4.png]

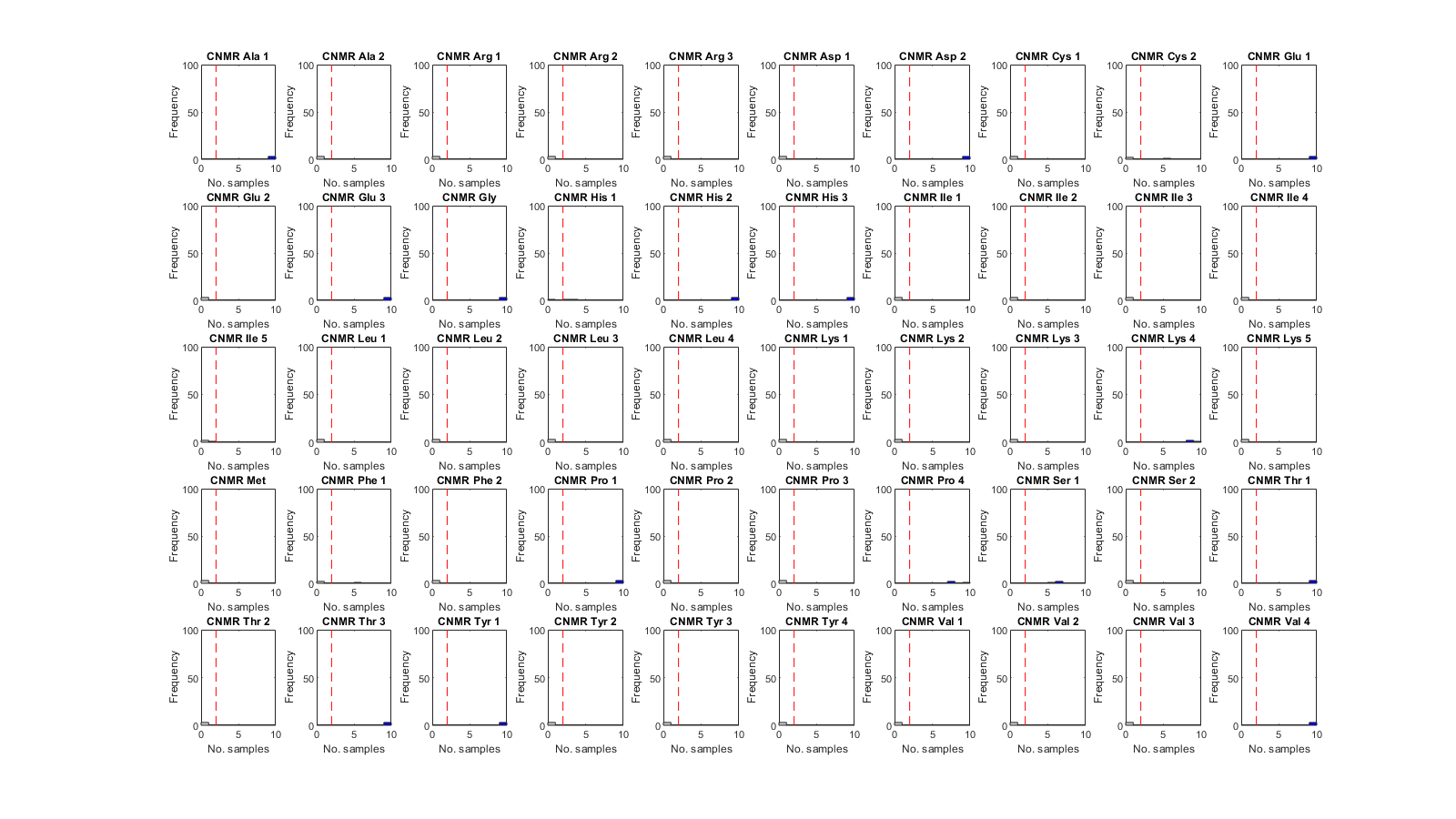

Supplement: S2 File — ZIP archive containing Matlab scripts to produce the substrate clusters and replicate evaluations (S4 and S5 Text). (ZIP) [file pcbi.1006533.s008.zip › Cluster/Cluster_3D_CNMR/Ausgabe_5.png]

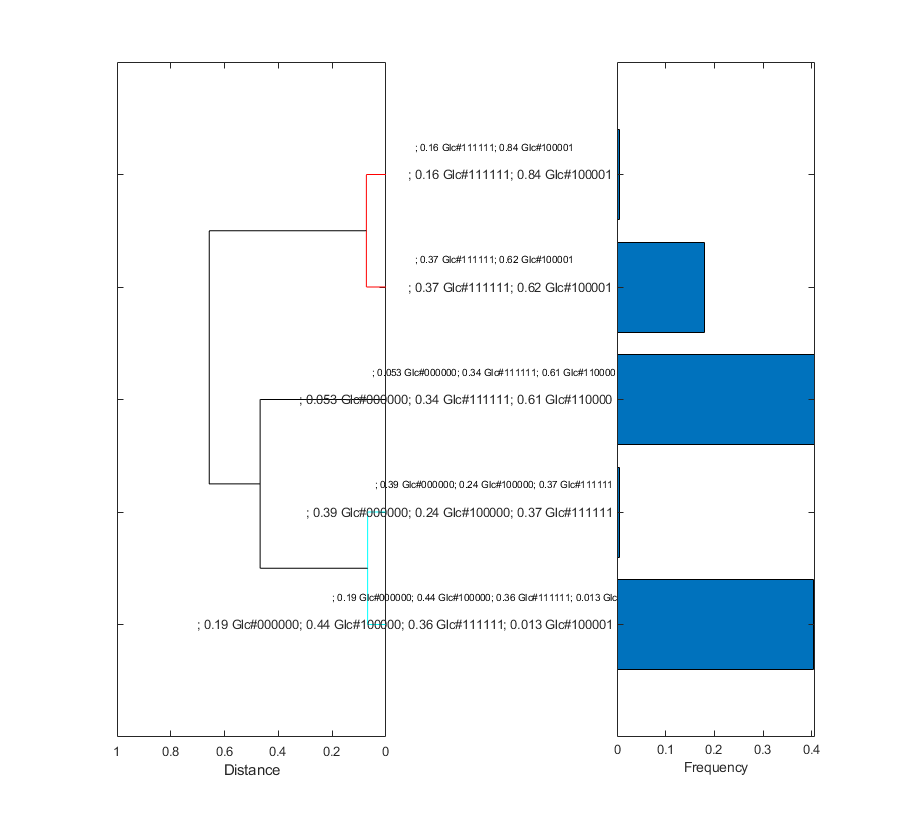

Supplement: S2 File — ZIP archive containing Matlab scripts to produce the substrate clusters and replicate evaluations (S4 and S5 Text). (ZIP) [file pcbi.1006533.s008.zip › Cluster/Cluster_3D_CNMR/Ausgabe_Dendro.png]

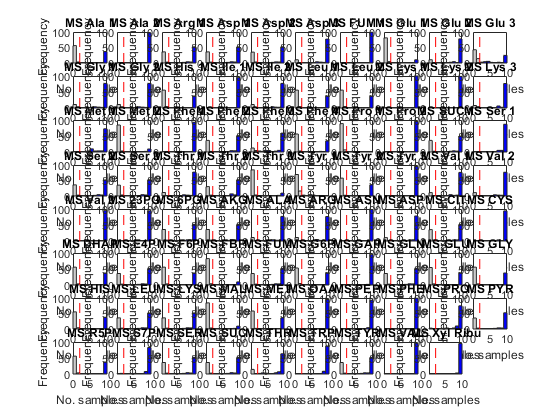

Supplement: S2 File — ZIP archive containing Matlab scripts to produce the substrate clusters and replicate evaluations (S4 and S5 Text). (ZIP) [file pcbi.1006533.s008.zip › Cluster/Cluster_3D_GCMSLCMS/Ausgabe_1.png]

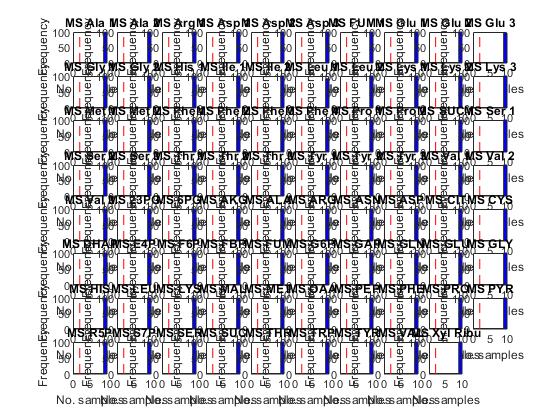

Supplement: S2 File — ZIP archive containing Matlab scripts to produce the substrate clusters and replicate evaluations (S4 and S5 Text). (ZIP) [file pcbi.1006533.s008.zip › Cluster/Cluster_3D_GCMSLCMS/Ausgabe_2.png]

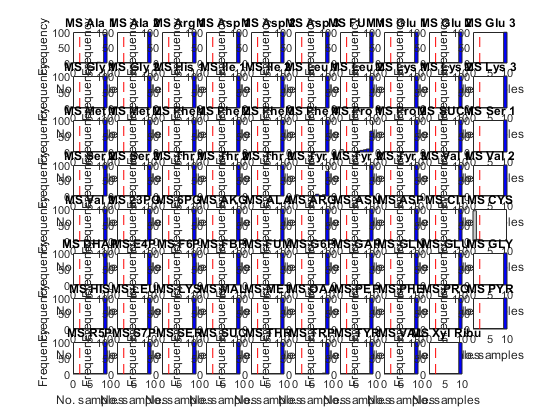

Supplement: S2 File — ZIP archive containing Matlab scripts to produce the substrate clusters and replicate evaluations (S4 and S5 Text). (ZIP) [file pcbi.1006533.s008.zip › Cluster/Cluster_3D_GCMSLCMS/Ausgabe_3.png]

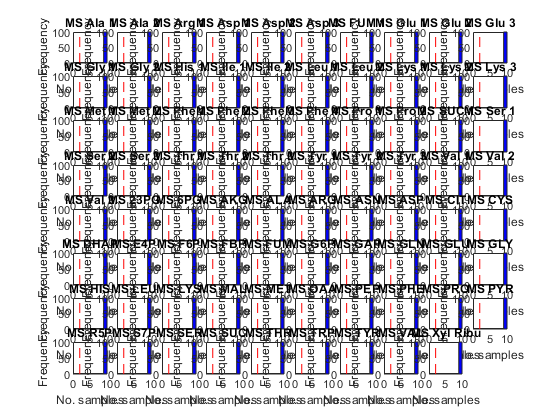

Supplement: S2 File — ZIP archive containing Matlab scripts to produce the substrate clusters and replicate evaluations (S4 and S5 Text). (ZIP) [file pcbi.1006533.s008.zip › Cluster/Cluster_3D_GCMSLCMS/Ausgabe_4.png]

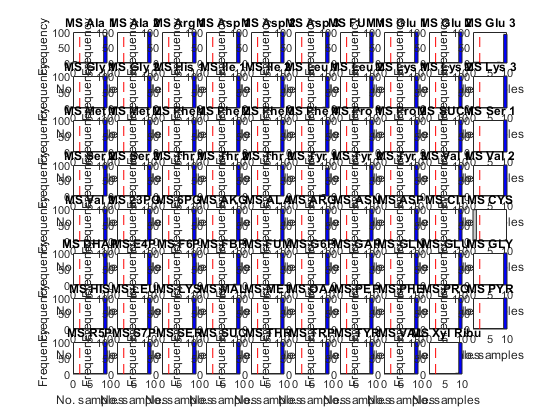

Supplement: S2 File — ZIP archive containing Matlab scripts to produce the substrate clusters and replicate evaluations (S4 and S5 Text). (ZIP) [file pcbi.1006533.s008.zip › Cluster/Cluster_3D_GCMSLCMS/Ausgabe_5.png]

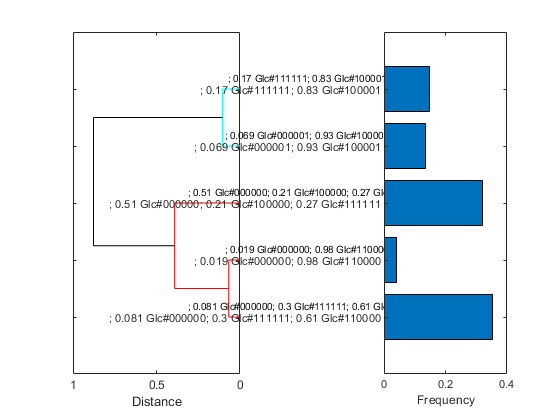

Supplement: S2 File — ZIP archive containing Matlab scripts to produce the substrate clusters and replicate evaluations (S4 and S5 Text). (ZIP) [file pcbi.1006533.s008.zip › Cluster/Cluster_3D_GCMSLCMS/Ausgabe_Dendro.png]

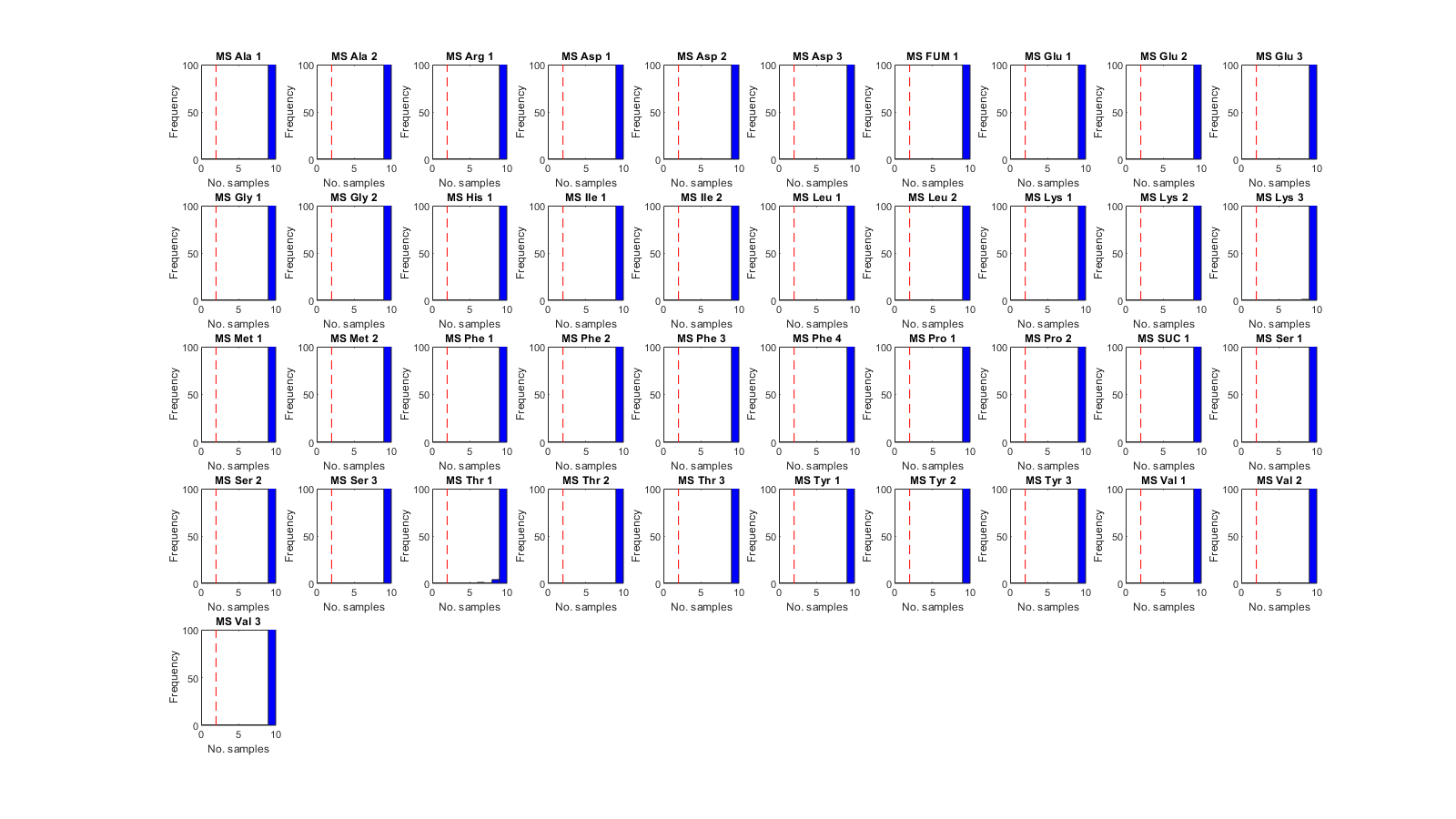

Supplement: S2 File — ZIP archive containing Matlab scripts to produce the substrate clusters and replicate evaluations (S4 and S5 Text). (ZIP) [file pcbi.1006533.s008.zip › Cluster/Cluster_3D_GCMS/Ausgabe_1.png]

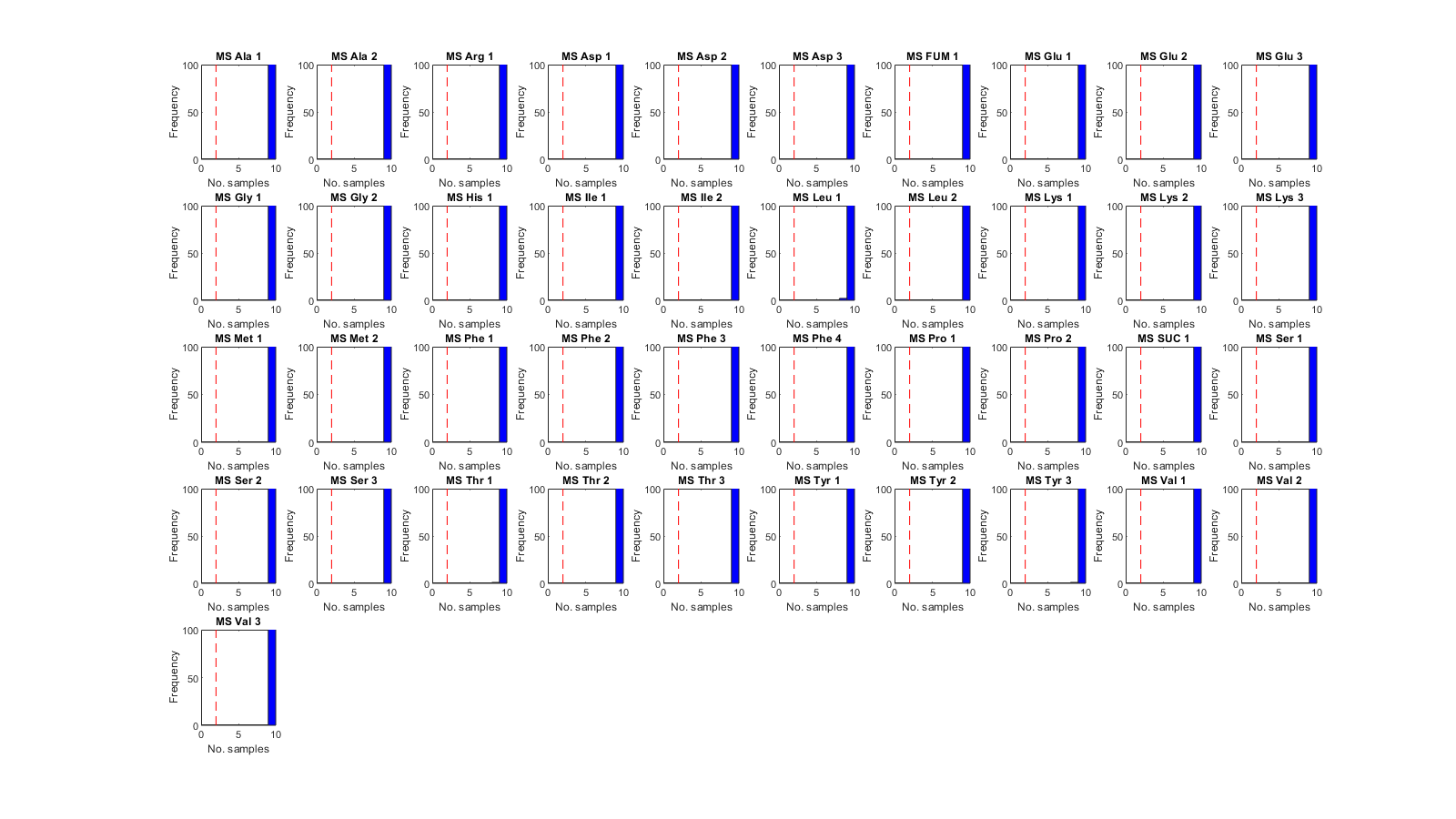

Supplement: S2 File — ZIP archive containing Matlab scripts to produce the substrate clusters and replicate evaluations (S4 and S5 Text). (ZIP) [file pcbi.1006533.s008.zip › Cluster/Cluster_3D_GCMS/Ausgabe_2.png]

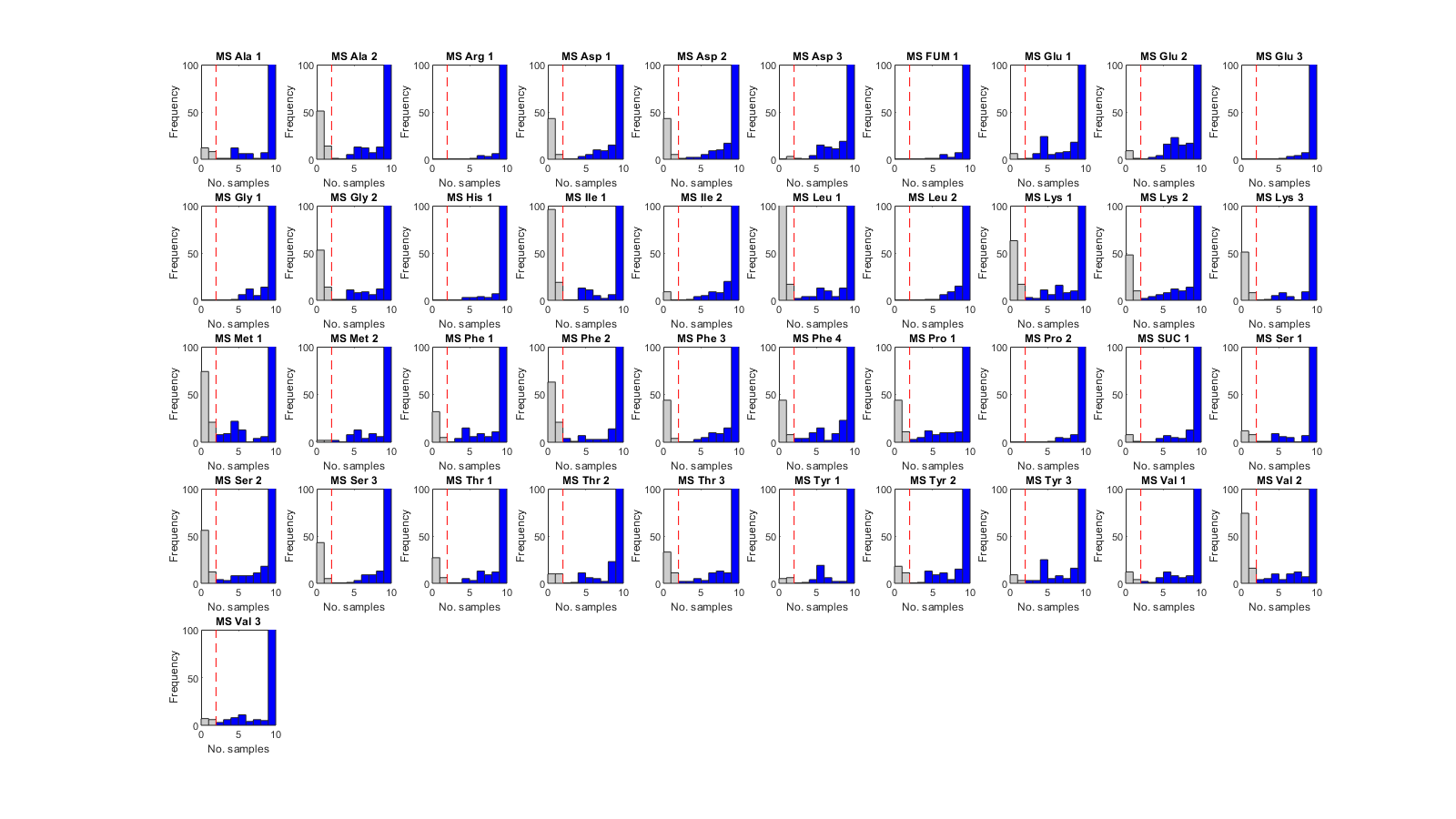

Supplement: S2 File — ZIP archive containing Matlab scripts to produce the substrate clusters and replicate evaluations (S4 and S5 Text). (ZIP) [file pcbi.1006533.s008.zip › Cluster/Cluster_3D_GCMS/Ausgabe_3.png]

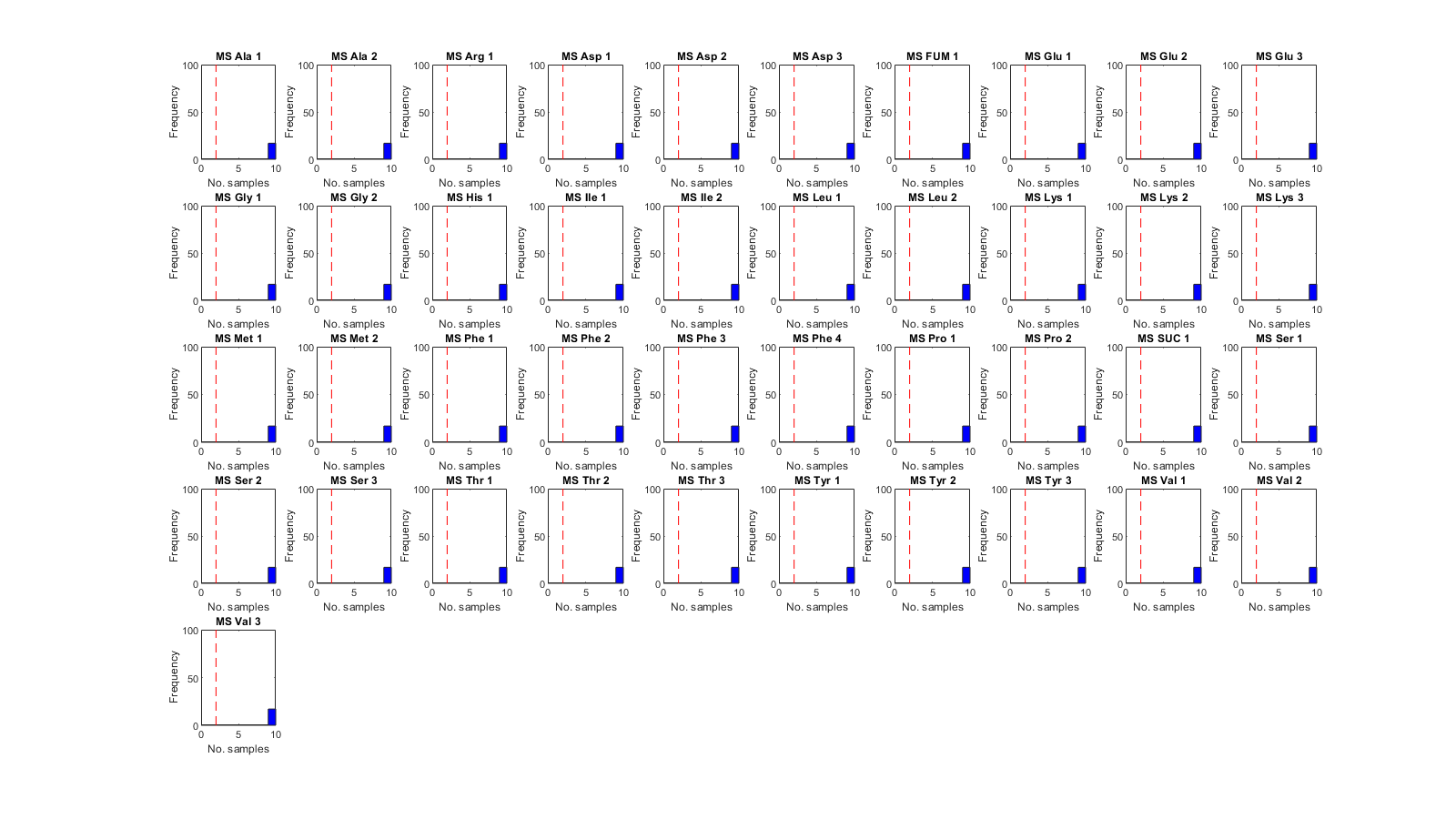

Supplement: S2 File — ZIP archive containing Matlab scripts to produce the substrate clusters and replicate evaluations (S4 and S5 Text). (ZIP) [file pcbi.1006533.s008.zip › Cluster/Cluster_3D_GCMS/Ausgabe_4.png]

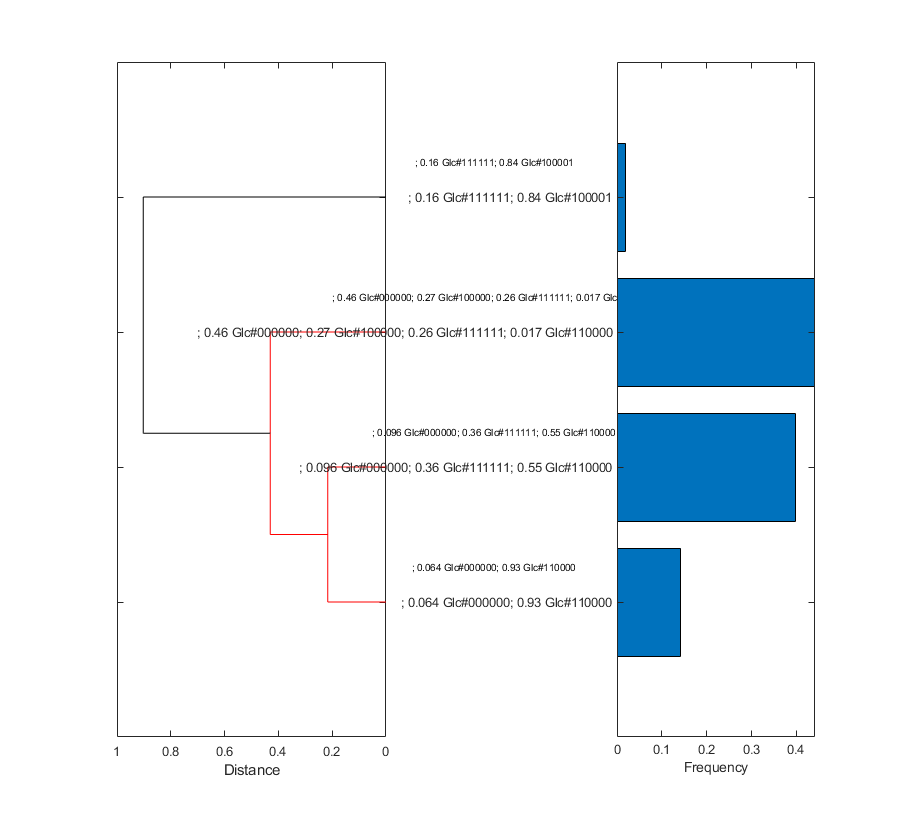

Supplement: S2 File — ZIP archive containing Matlab scripts to produce the substrate clusters and replicate evaluations (S4 and S5 Text). (ZIP) [file pcbi.1006533.s008.zip › Cluster/Cluster_3D_GCMS/Ausgabe_Dendro.png]

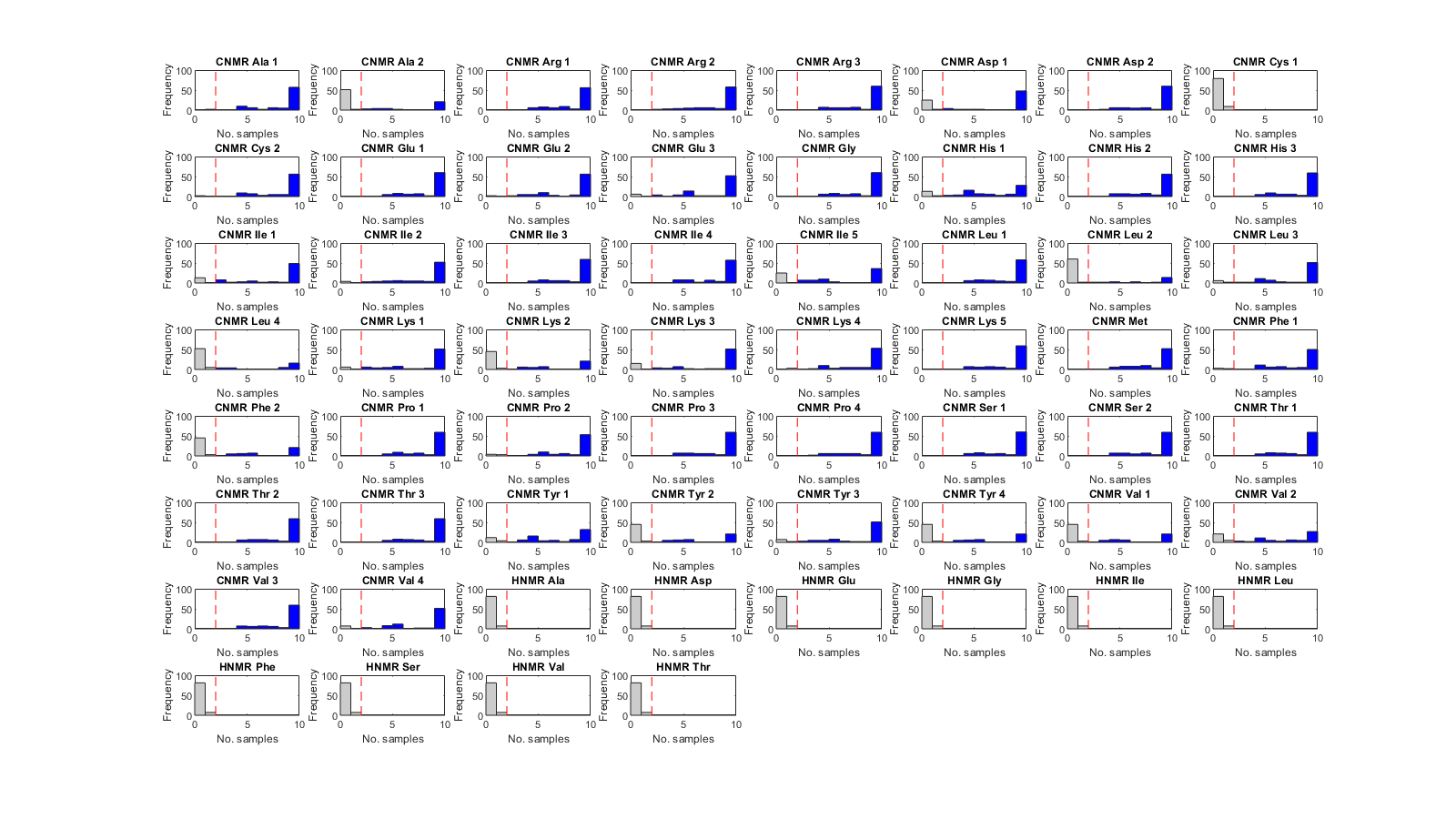

Supplement: S2 File — ZIP archive containing Matlab scripts to produce the substrate clusters and replicate evaluations (S4 and S5 Text). (ZIP) [file pcbi.1006533.s008.zip › Cluster/Cluster_3D_HNMRCNMR/Ausgabe_1.png]

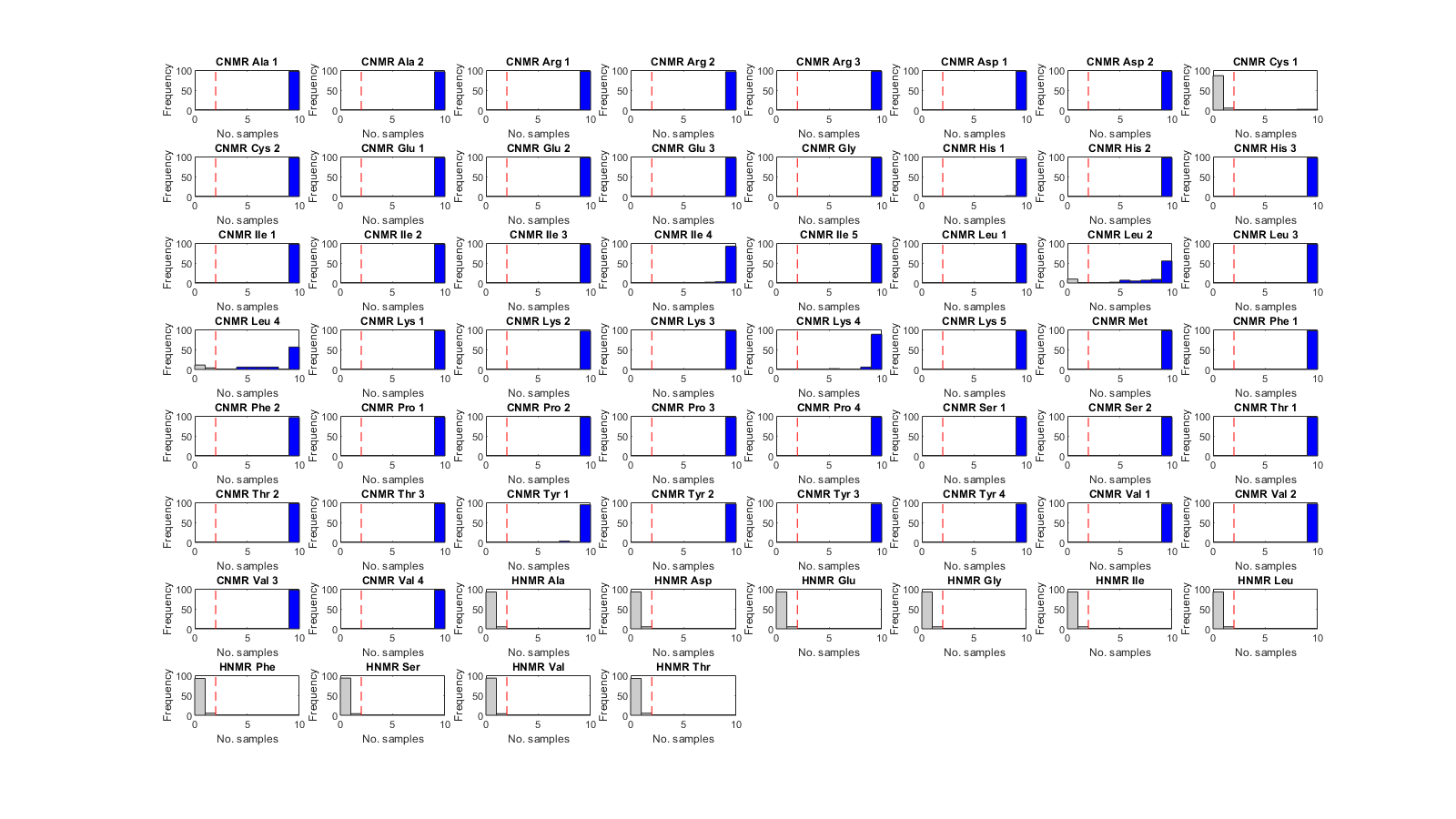

Supplement: S2 File — ZIP archive containing Matlab scripts to produce the substrate clusters and replicate evaluations (S4 and S5 Text). (ZIP) [file pcbi.1006533.s008.zip › Cluster/Cluster_3D_HNMRCNMR/Ausgabe_2.png]

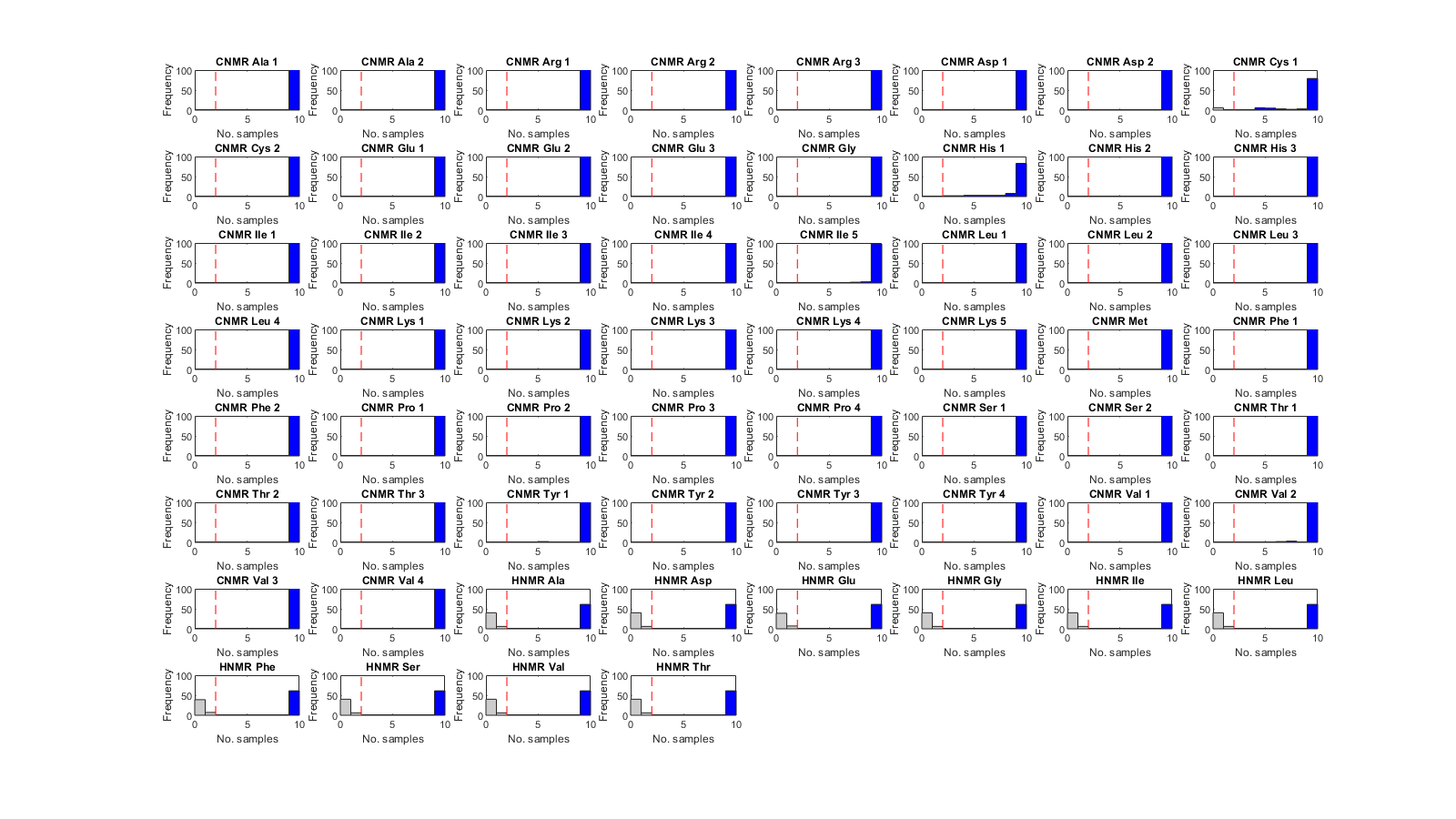

Supplement: S2 File — ZIP archive containing Matlab scripts to produce the substrate clusters and replicate evaluations (S4 and S5 Text). (ZIP) [file pcbi.1006533.s008.zip › Cluster/Cluster_3D_HNMRCNMR/Ausgabe_3.png]

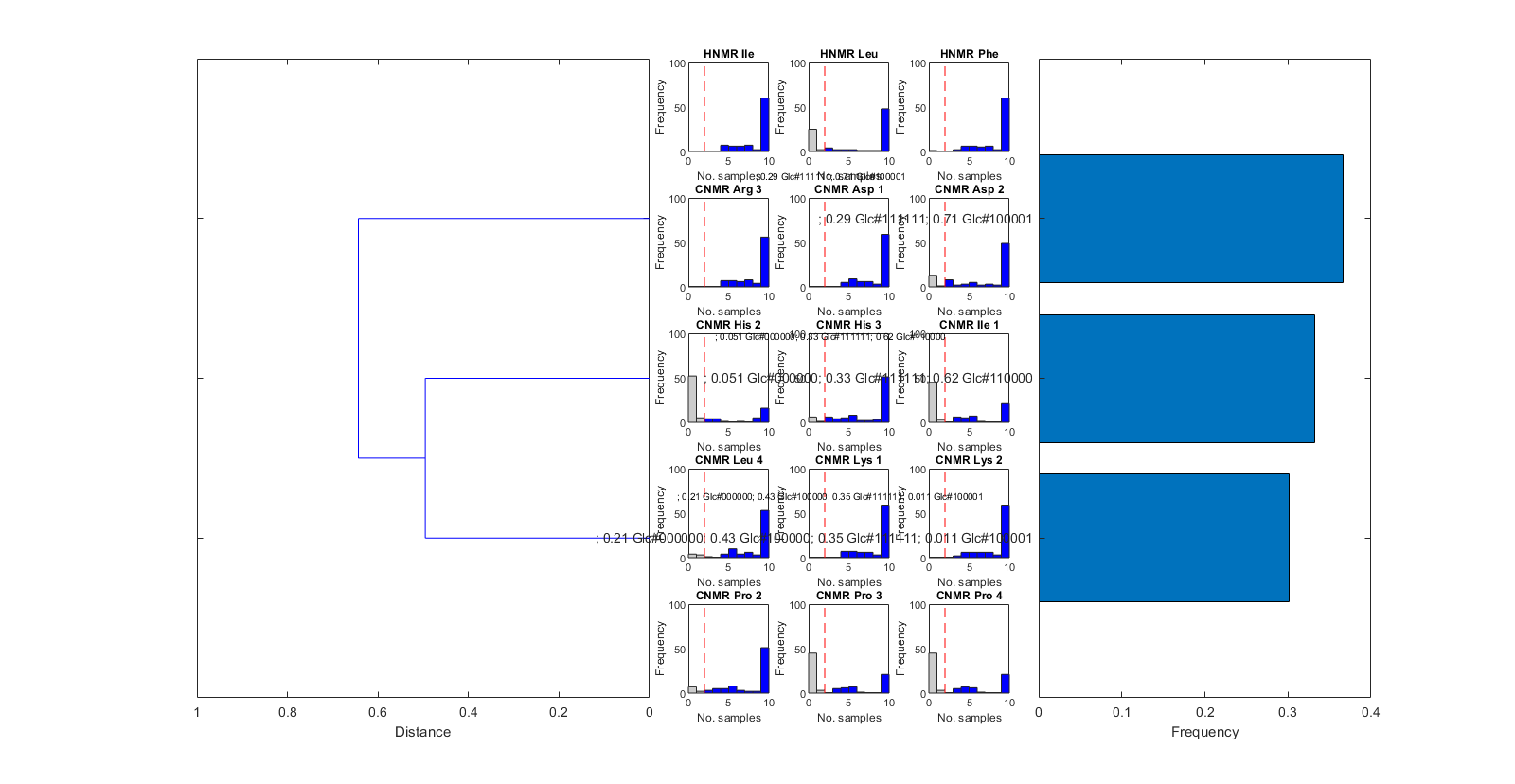

Supplement: S2 File — ZIP archive containing Matlab scripts to produce the substrate clusters and replicate evaluations (S4 and S5 Text). (ZIP) [file pcbi.1006533.s008.zip › Cluster/Cluster_3D_HNMRCNMR/Ausgabe_Dendro.png]

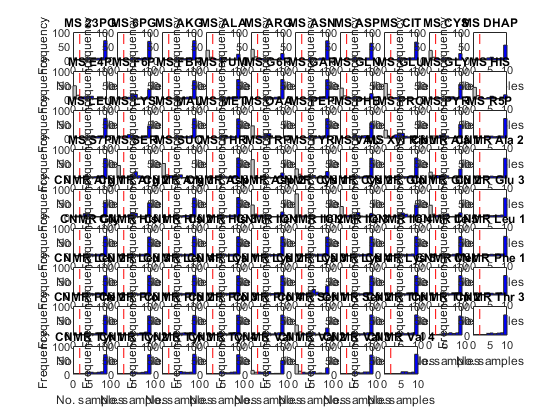

Supplement: S2 File — ZIP archive containing Matlab scripts to produce the substrate clusters and replicate evaluations (S4 and S5 Text). (ZIP) [file pcbi.1006533.s008.zip › Cluster/Cluster_3D_LCMSMSCNMR/Ausgabe_1.png]

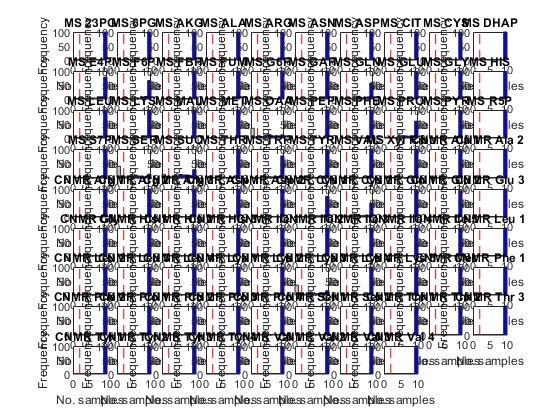

Supplement: S2 File — ZIP archive containing Matlab scripts to produce the substrate clusters and replicate evaluations (S4 and S5 Text). (ZIP) [file pcbi.1006533.s008.zip › Cluster/Cluster_3D_LCMSMSCNMR/Ausgabe_2.png]

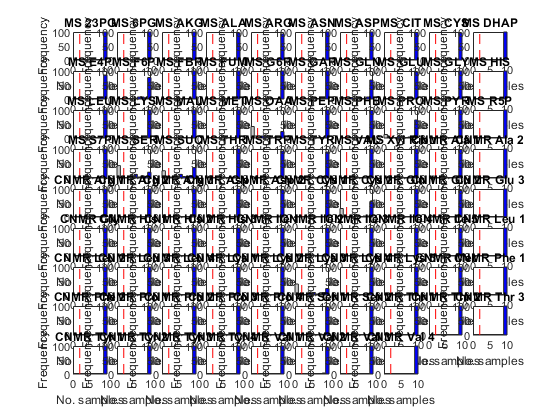

Supplement: S2 File — ZIP archive containing Matlab scripts to produce the substrate clusters and replicate evaluations (S4 and S5 Text). (ZIP) [file pcbi.1006533.s008.zip › Cluster/Cluster_3D_LCMSMSCNMR/Ausgabe_3.png]

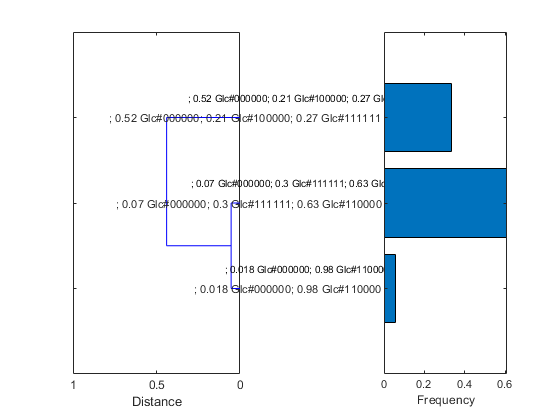

Supplement: S2 File — ZIP archive containing Matlab scripts to produce the substrate clusters and replicate evaluations (S4 and S5 Text). (ZIP) [file pcbi.1006533.s008.zip › Cluster/Cluster_3D_LCMSMSCNMR/Ausgabe_Dendro.png]

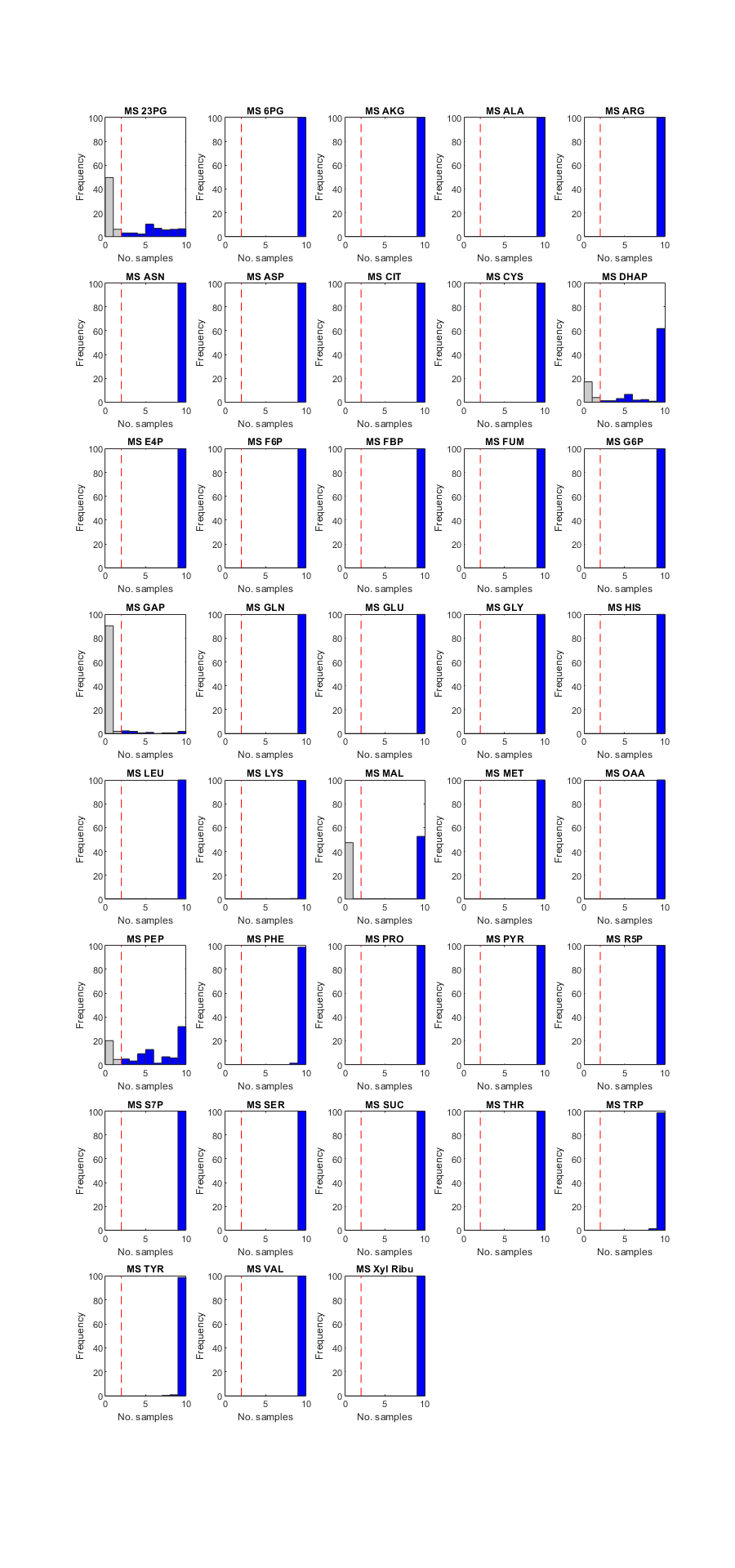

Supplement: S2 File — ZIP archive containing Matlab scripts to produce the substrate clusters and replicate evaluations (S4 and S5 Text). (ZIP) [file pcbi.1006533.s008.zip › Cluster/Cluster_3D_LCMSMS/Ausgabe_1.png]

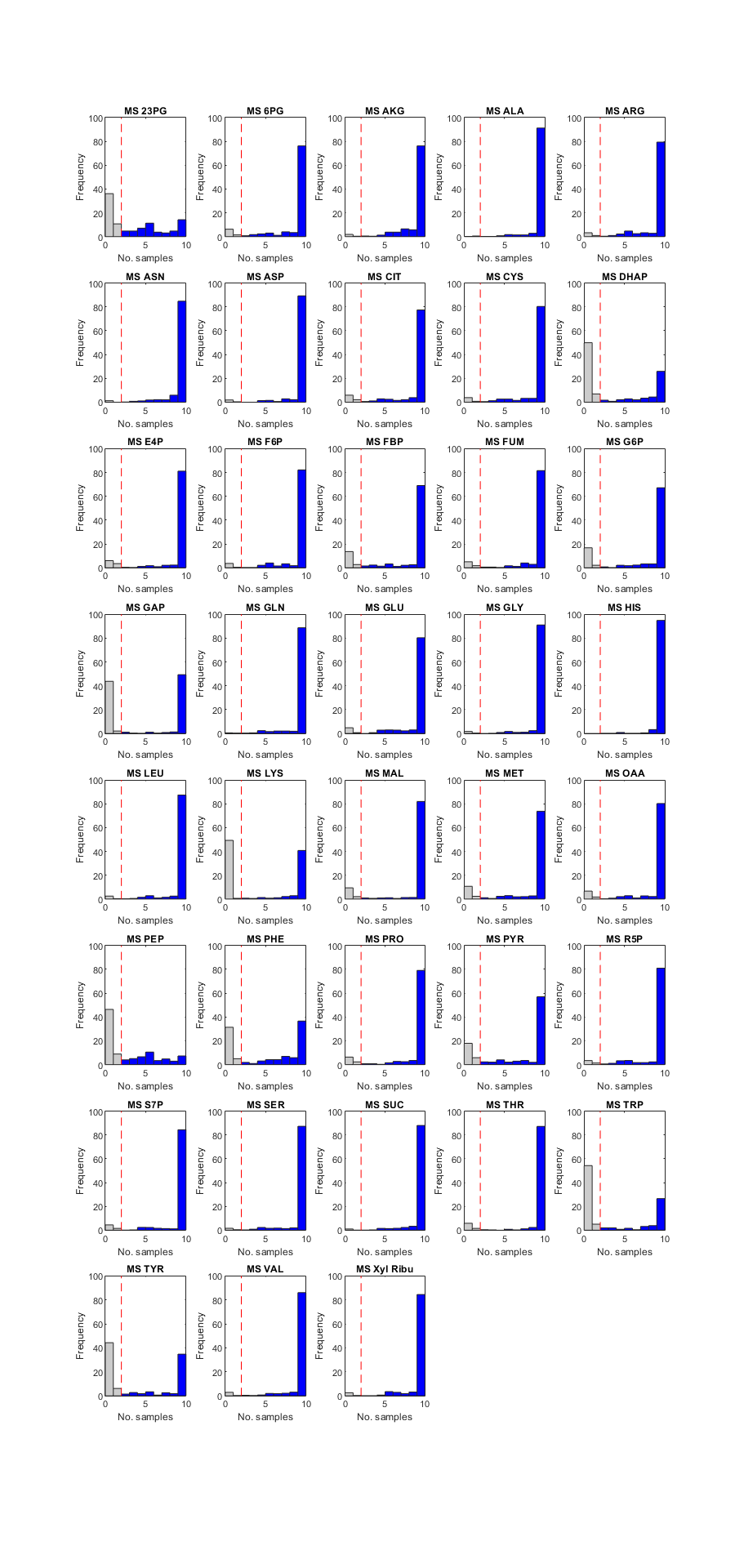

Supplement: S2 File — ZIP archive containing Matlab scripts to produce the substrate clusters and replicate evaluations (S4 and S5 Text). (ZIP) [file pcbi.1006533.s008.zip › Cluster/Cluster_3D_LCMSMS/Ausgabe_2.png]

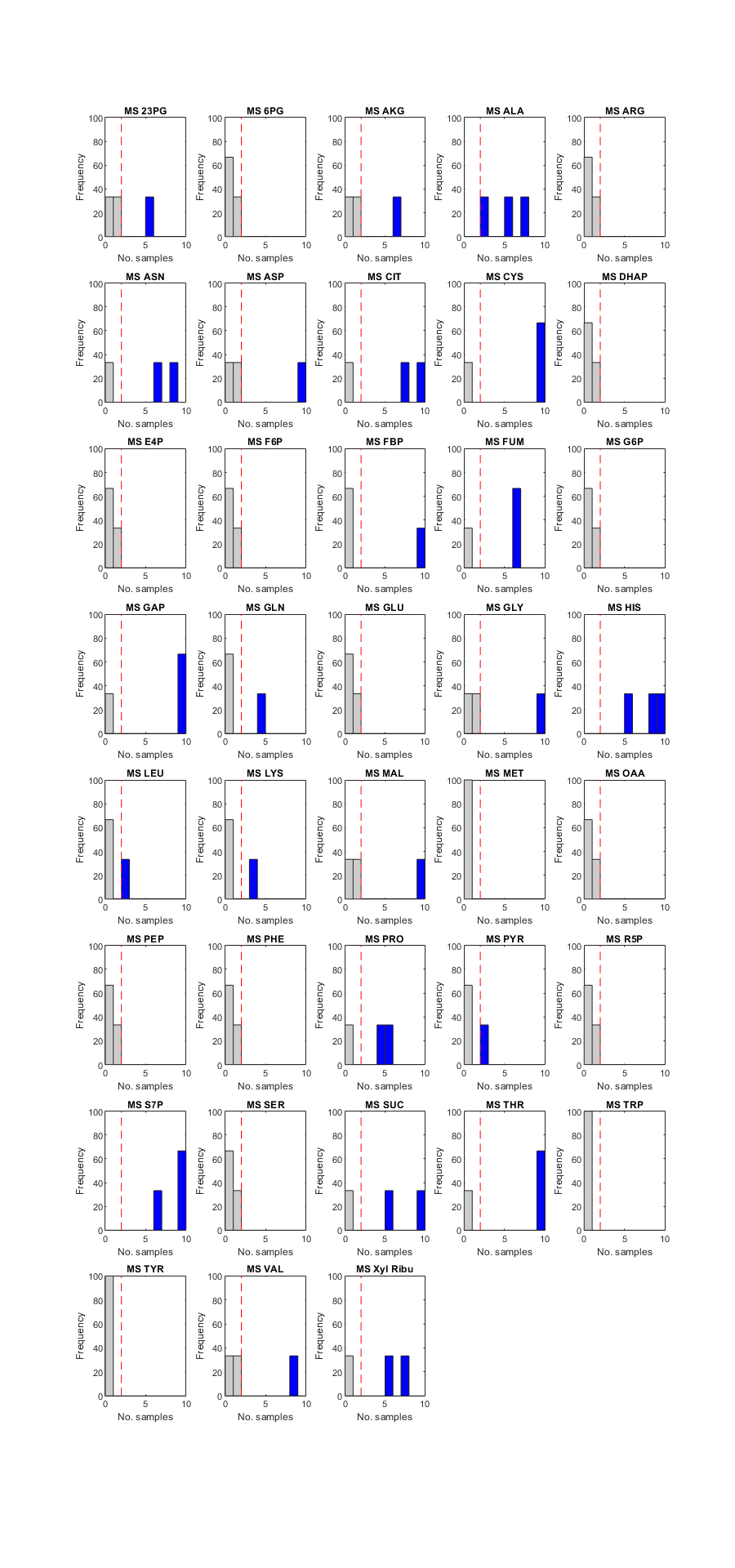

Supplement: S2 File — ZIP archive containing Matlab scripts to produce the substrate clusters and replicate evaluations (S4 and S5 Text). (ZIP) [file pcbi.1006533.s008.zip › Cluster/Cluster_3D_LCMSMS/Ausgabe_3.png]

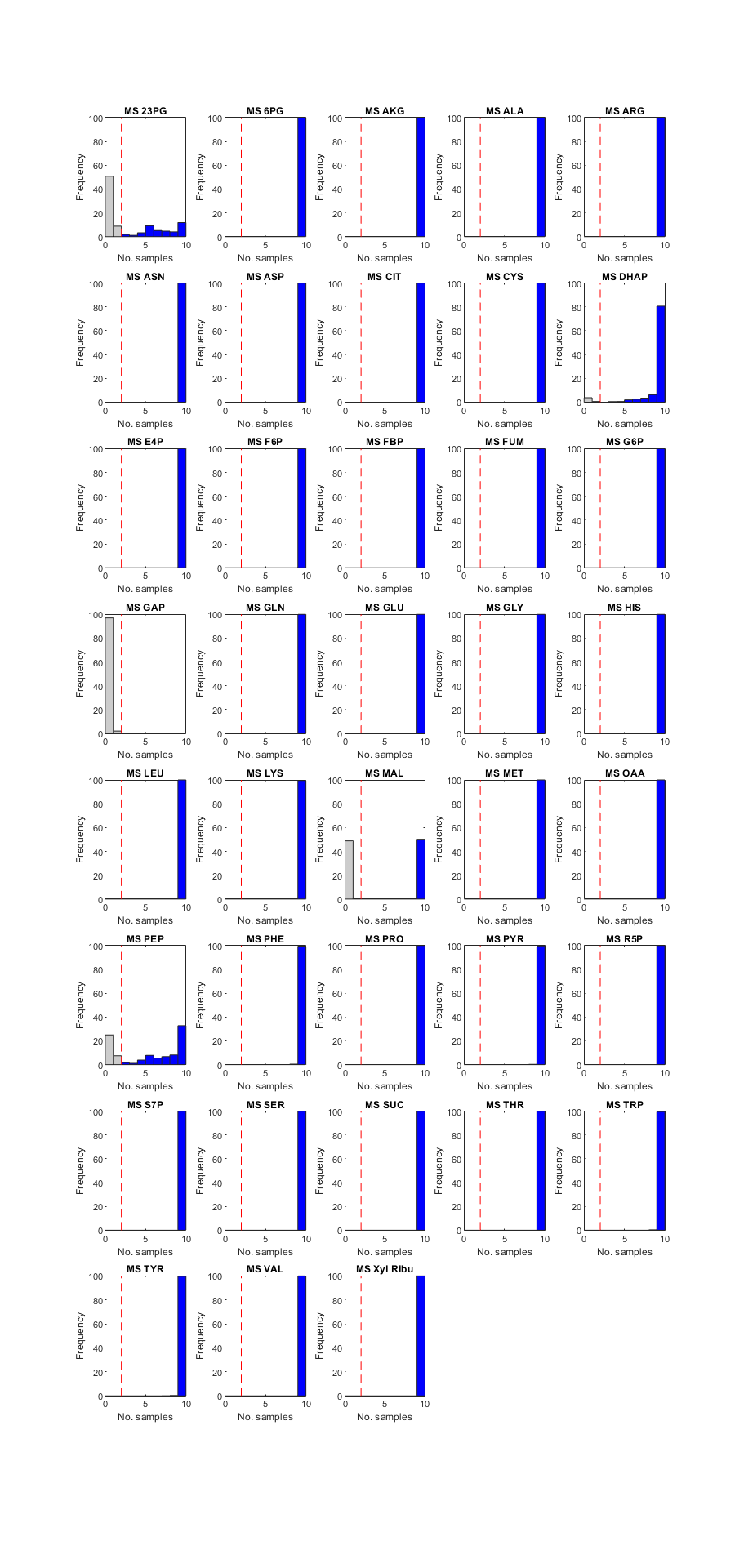

Supplement: S2 File — ZIP archive containing Matlab scripts to produce the substrate clusters and replicate evaluations (S4 and S5 Text). (ZIP) [file pcbi.1006533.s008.zip › Cluster/Cluster_3D_LCMSMS/Ausgabe_4.png]

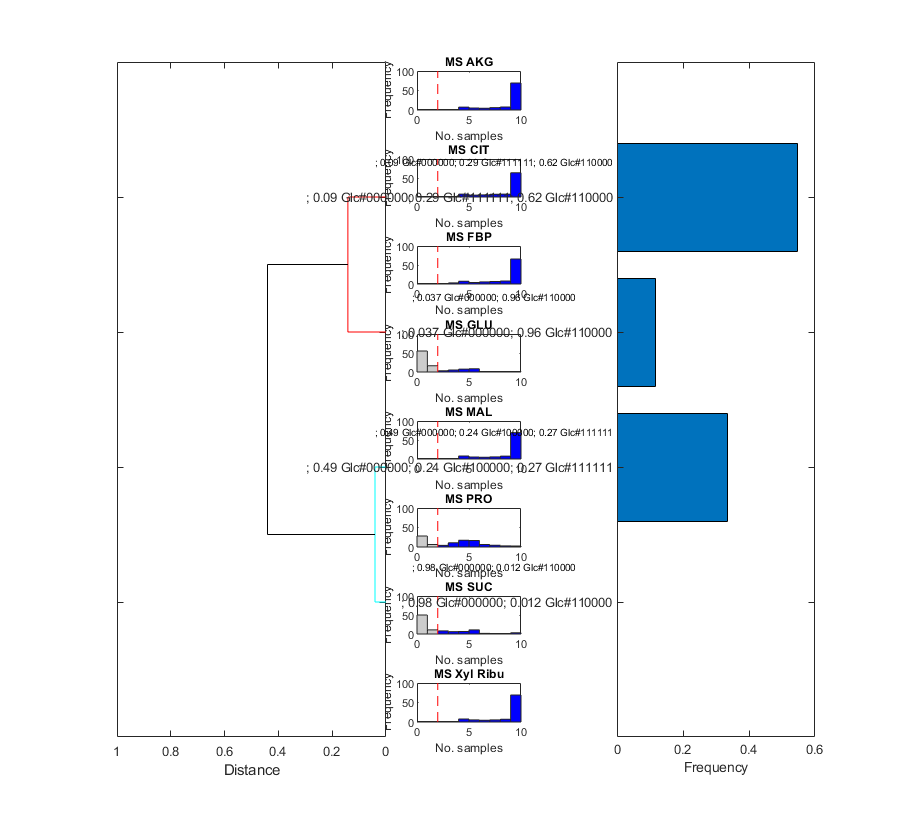

Supplement: S2 File — ZIP archive containing Matlab scripts to produce the substrate clusters and replicate evaluations (S4 and S5 Text). (ZIP) [file pcbi.1006533.s008.zip › Cluster/Cluster_3D_LCMSMS/Ausgabe_Dendro.png]

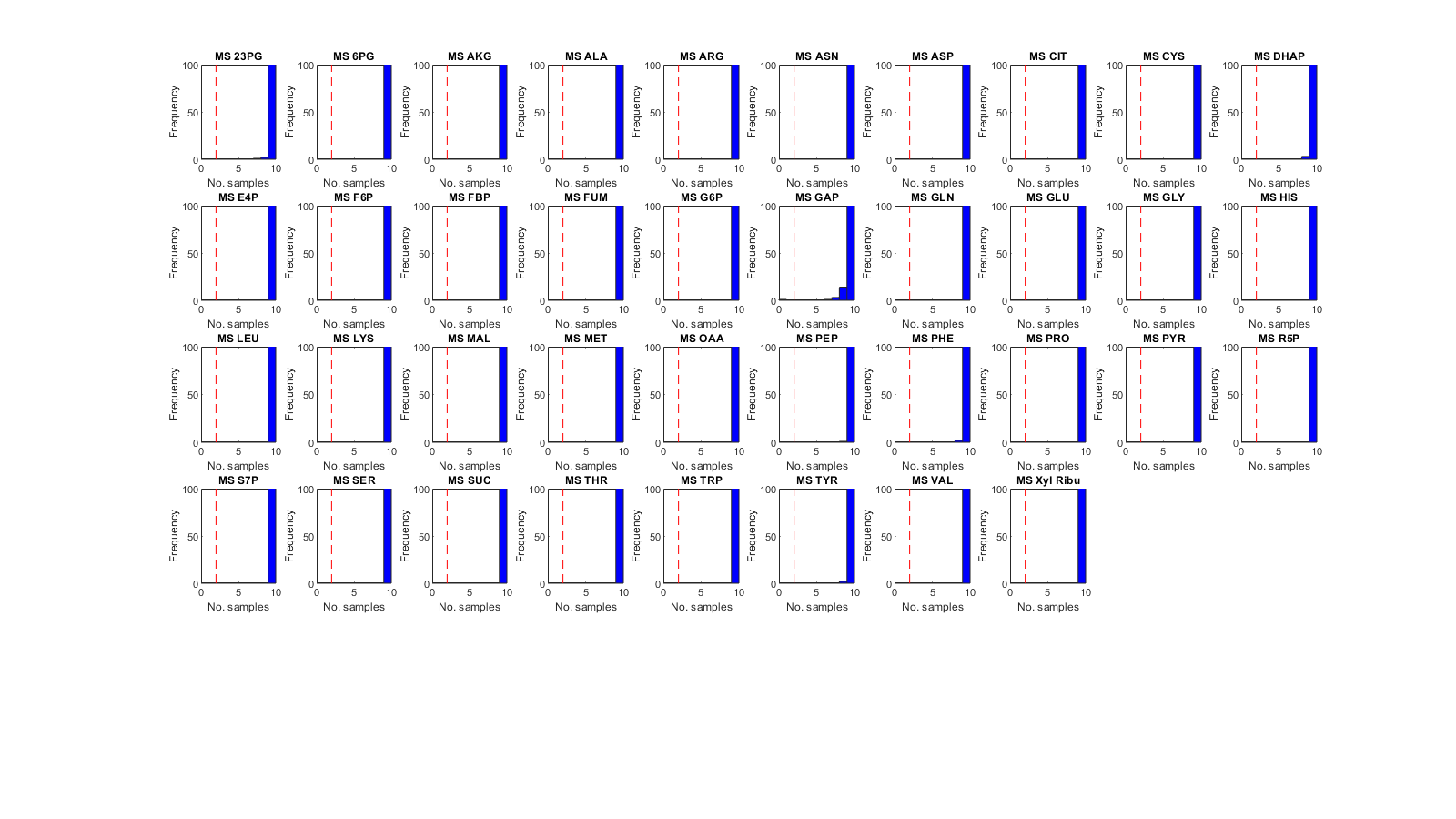

Supplement: S2 File — ZIP archive containing Matlab scripts to produce the substrate clusters and replicate evaluations (S4 and S5 Text). (ZIP) [file pcbi.1006533.s008.zip › Cluster/Cluster_3D_LCMS/Ausgabe_1.png]

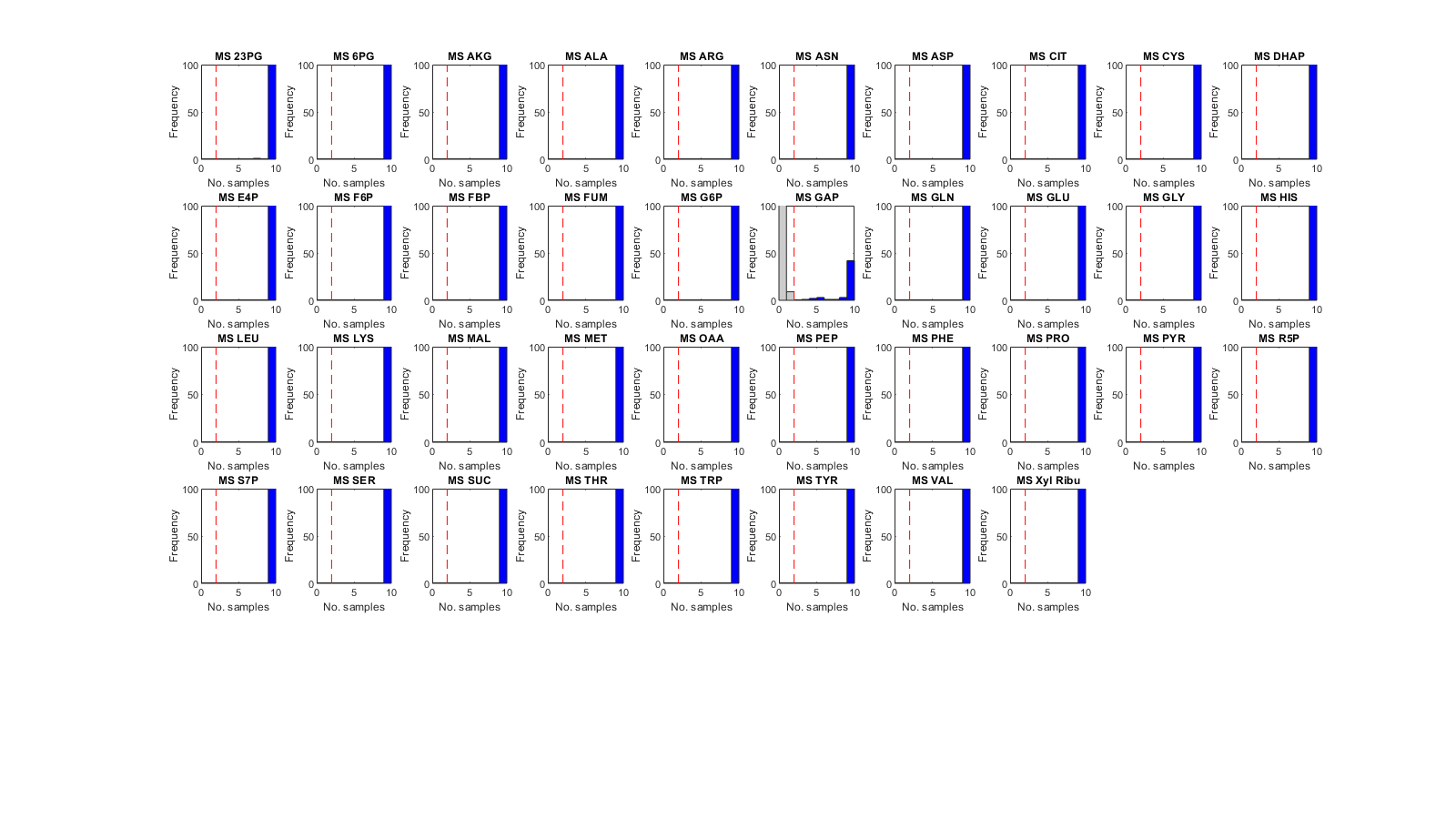

Supplement: S2 File — ZIP archive containing Matlab scripts to produce the substrate clusters and replicate evaluations (S4 and S5 Text). (ZIP) [file pcbi.1006533.s008.zip › Cluster/Cluster_3D_LCMS/Ausgabe_2.png]

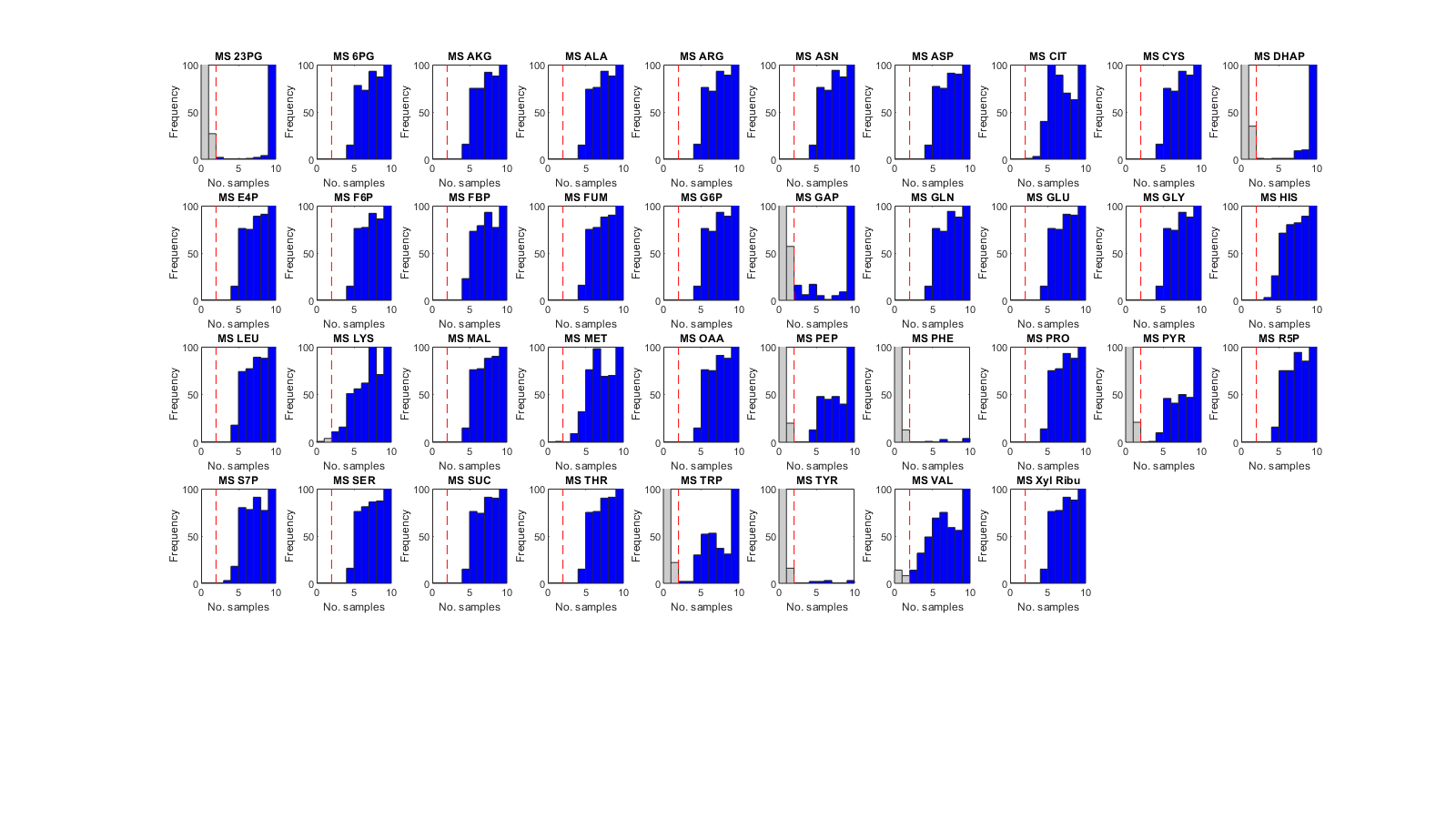

Supplement: S2 File — ZIP archive containing Matlab scripts to produce the substrate clusters and replicate evaluations (S4 and S5 Text). (ZIP) [file pcbi.1006533.s008.zip › Cluster/Cluster_3D_LCMS/Ausgabe_3.png]

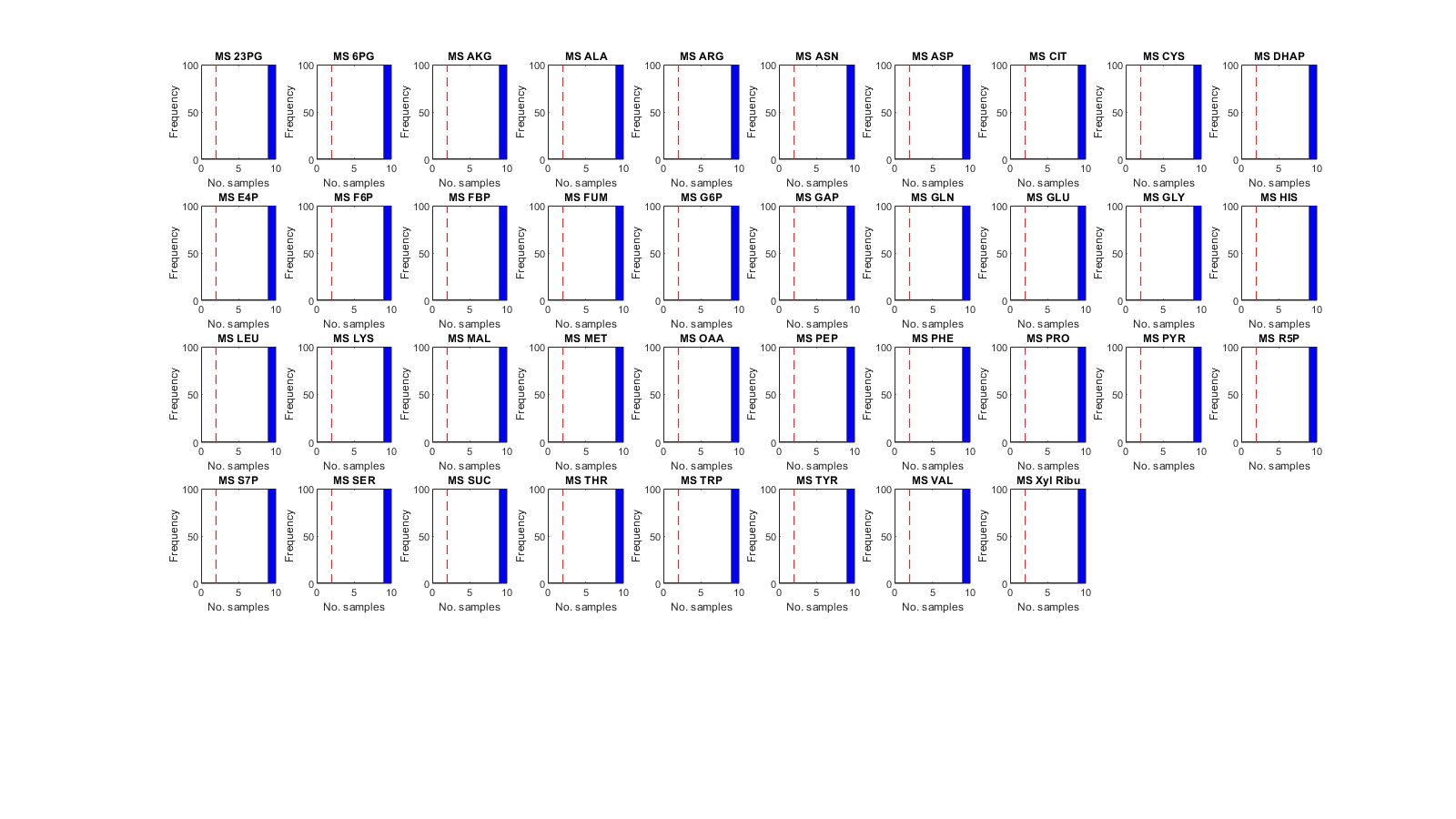

Supplement: S2 File — ZIP archive containing Matlab scripts to produce the substrate clusters and replicate evaluations (S4 and S5 Text). (ZIP) [file pcbi.1006533.s008.zip › Cluster/Cluster_3D_LCMS/Ausgabe_4.png]

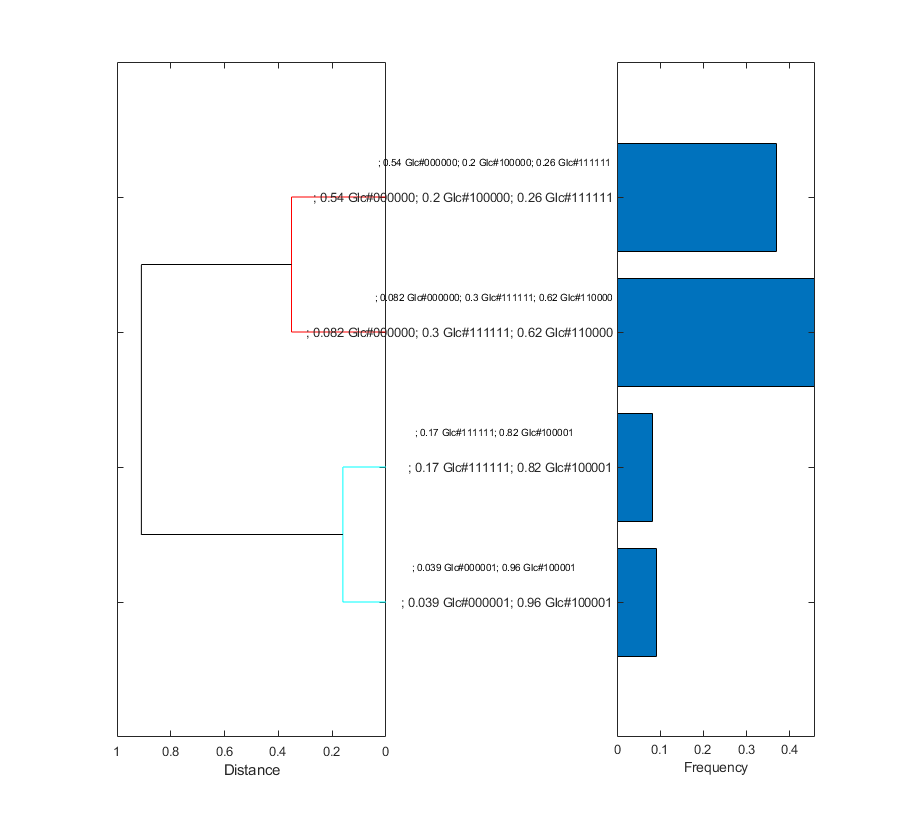

Supplement: S2 File — ZIP archive containing Matlab scripts to produce the substrate clusters and replicate evaluations (S4 and S5 Text). (ZIP) [file pcbi.1006533.s008.zip › Cluster/Cluster_3D_LCMS/Ausgabe_Dendro.png]

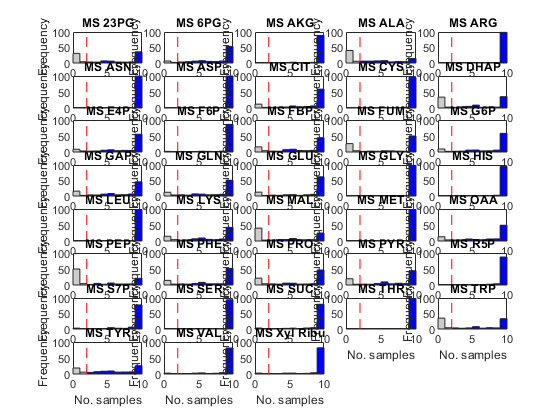

Supplement: S2 File — ZIP archive containing Matlab scripts to produce the substrate clusters and replicate evaluations (S4 and S5 Text). (ZIP) [file pcbi.1006533.s008.zip › Cluster/Cluster_5D_LCMSMS/Ausgabe_1.png]

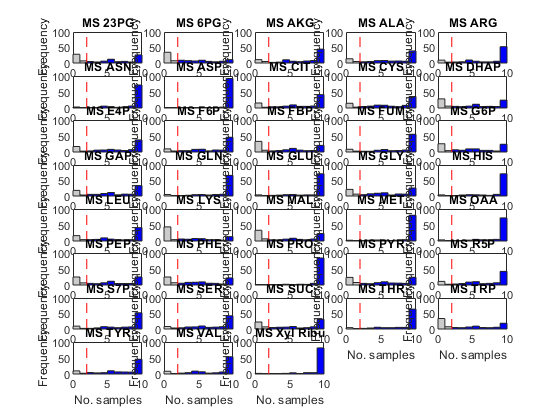

Supplement: S2 File — ZIP archive containing Matlab scripts to produce the substrate clusters and replicate evaluations (S4 and S5 Text). (ZIP) [file pcbi.1006533.s008.zip › Cluster/Cluster_5D_LCMSMS/Ausgabe_2.png]

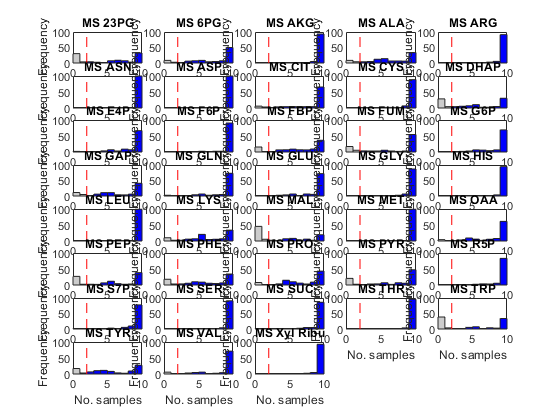

Supplement: S2 File — ZIP archive containing Matlab scripts to produce the substrate clusters and replicate evaluations (S4 and S5 Text). (ZIP) [file pcbi.1006533.s008.zip › Cluster/Cluster_5D_LCMSMS/Ausgabe_3.png]

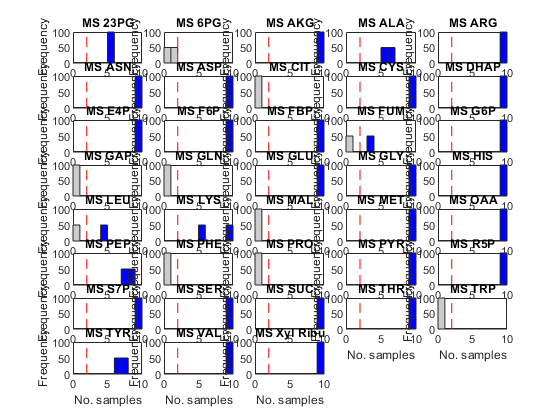

Supplement: S2 File — ZIP archive containing Matlab scripts to produce the substrate clusters and replicate evaluations (S4 and S5 Text). (ZIP) [file pcbi.1006533.s008.zip › Cluster/Cluster_5D_LCMSMS/Ausgabe_4.png]

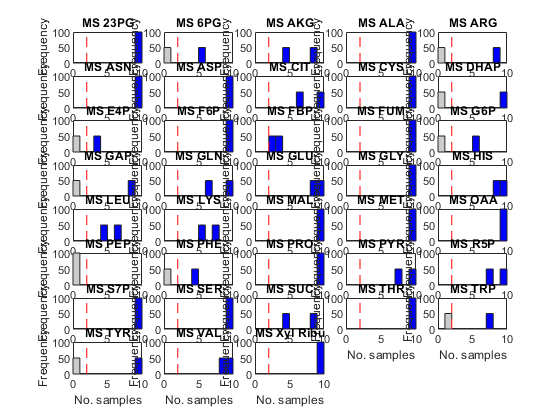

Supplement: S2 File — ZIP archive containing Matlab scripts to produce the substrate clusters and replicate evaluations (S4 and S5 Text). (ZIP) [file pcbi.1006533.s008.zip › Cluster/Cluster_5D_LCMSMS/Ausgabe_5.png]

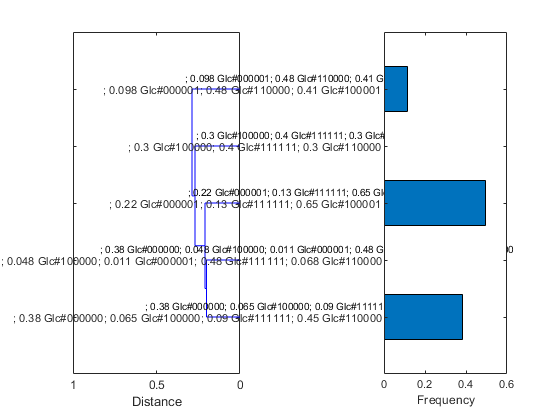

Supplement: S2 File — ZIP archive containing Matlab scripts to produce the substrate clusters and replicate evaluations (S4 and S5 Text). (ZIP) [file pcbi.1006533.s008.zip › Cluster/Cluster_5D_LCMSMS/Ausgabe_Dendro.png]

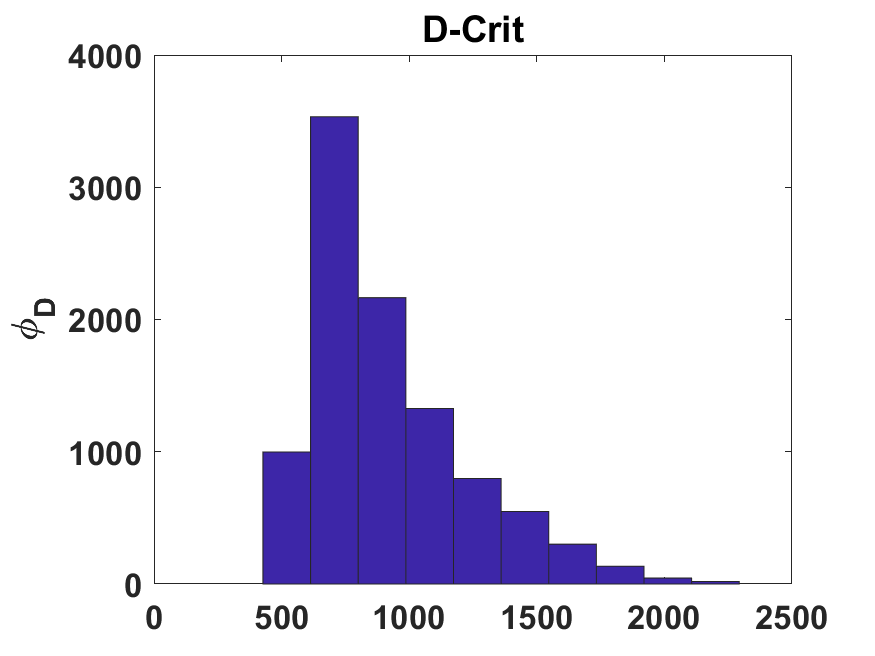

Supplement: S3 File — ZIP archive containing information criteria for flux space samples and Matlab scripts to evaluate variations. (ZIP) [file pcbi.1006533.s009.zip › Sensitivity/Sensitivity_3D_CNMR/3D-MOED-CNMR-D_Sampling.png]
